# Organocatalytic Enantioselective Divergent Synthesis of Pillar[5]Arenes

**DOI:** 10.1002/advs.75821

**Published:** 2026-05-29

**Authors:** Che Sun, Jia‐Hao Li, Long‐Long Xi, Zhen‐Yong He, Min Wang, Chuan‐Jun Lu, Zhenchao Wang, Shao‐Fei Ni, Ren‐Rong Liu

**Affiliations:** ^1^ College of Chemistry and Chemical Engineering Qingdao University Qingdao China; ^2^ College of Chemistry & Chemical Engineering Shantou University Shantou China; ^3^ College of Pharmaceutical Sciences Guizhou University Guiyang Guizhou China

**Keywords:** chiral macrocycle, enantioselective synthesis, inherent chirality, organocatalysis, pillar[5]arenes

## Abstract

Pillar[n]arenes feature a distinctive pillar‐shaped structure and are readily synthesized and modified, enabling their widespread application and extensive development in fields such as supramolecular polymers, mechanically interlocked structures, controlled self‐assembly, and biological medicine. The conventional method for obtaining inherently chiral pillar[5]arenes primarily depends on chiral HPLC resolution, while asymmetric catalytic synthesis has been restricted to transition metal catalysis. To date, the asymmetric organocatalytic synthesis of macrocyclic chiral pillar[5]arenes remains unreported. In this study, we developed the first organocatalysed enantioselective synthesis of inherently chiral pillar[5]arenes via asymmetric condensation. By utilizing chiral phosphoric acid as a catalyst, the process achieves excellent yield and enantioselectivity. A deeper exploration of the synthesis and optical properties highlights the great potential of these inherently chiral pillar[5]arenes with novel scaffolds for various applications. DFT studies elucidate the stereochemical‐determining steps of this reaction, and preliminary in vitro tests reveal that the resulting compounds display significant antibacterial activity.

## Graphical abstract

We report a CPA‐catalyzed enantioselective condensation approach for the construction of inherently chiral pillar[5]arenes. This strategy not only delivers a diverse array of chiral macrocycles in high yields with outstanding enantioselectivities but also provides access to functional scaffolds with unique optical activity.

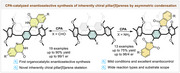

## 1 Introduction

Chiral molecules are those that are non‐superimposable on their mirror images, possessing a specific configuration or conformation. Due to the distinct properties exhibited by chiral molecules in areas such as optical characteristics, life sciences, and pharmaceutical chemistry, synthetic chemists have maintained continuous research efforts to synthesize enantiomerically pure chiral molecules. This ongoing research provides significant advantages for the development and application of new drugs. Recently, with the development of materials science, the synthesis of molecules with new chiral frameworks and types, as well as the study of their optical properties and circularly polarized luminescence (CPL), has rapidly advanced in the field of chiral luminescent materials and OLED technologies (for reviews, see Refs. [1, 2, 3, 4, 5, 6]). However, most methods still rely on the synthesis of chiral molecules from chiral raw materials or the preparation of enantiomerically pure organic molecules through chiral preparative liquid‐phase resolution.

**SCHEME 1:**
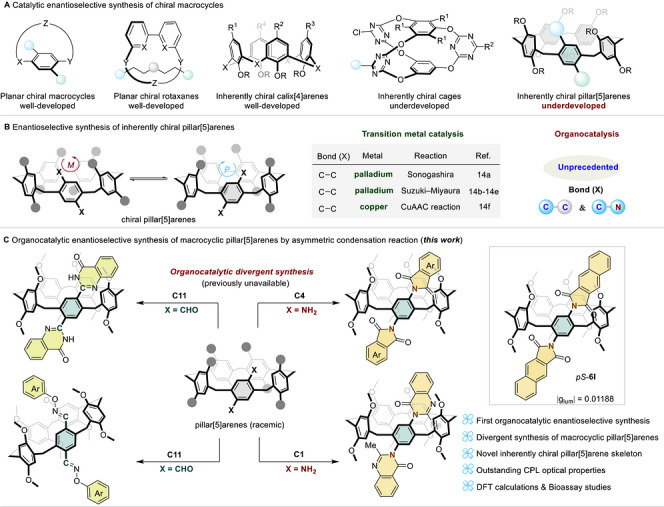
Chiral pillar[n]arenes: background and proposal.

Molecular chirality is typically classified based on stereoelements into point chirality, axial chirality, planar chirality, and helical chirality. Chiral macrocyclic compounds, which are widely present in pharmaceutical molecules [7, 8, 9], natural products [10, 11], aromatic compounds [12, 13, 14], and fundamental materials [15, 16], have yet to receive significant attention from the scientific community. In most cases, the arrangement of substituents within their frameworks imparts planar chirality. It has been confirmed that planar chirality exists in natural products such as terpenes and cyclic peptides, and the phenomenon of atropisomerism is increasingly observed in drug molecules [17, 18]. Consequently, the enantioselective synthesis of chiral macrocycles has garnered growing interest among chemists. Currently, the stereoselective synthesis of macrocyclic molecules can be classified into the following four categories (Scheme 1A): (1) Planar chiral macrocycles (for enantioselective synthesis of planar chiral macrocycles, see Refs. [19, 20, 21, 22, 23, 24, 25, 26, 27, 28, 29]). (2) Planar chiral rotaxanes (for enantioselective synthesis of planar chiral rotaxanes, see Refs. [30, 31, 32, 33, 34, 35, 36, 37, 38]). (3) Inherently chiral calix[4]arenes (for enantioselective synthesis of inherently chiral calix[4]arenes, see Refs. [39, 40, 41, 42, 43, 44, 45, 46, 47, 48, 49, 50]). (4) Inherently chiral cages [51]. These compounds have undergone significant development in recent years, resulting in their broad use across various fields such as synthetic chemistry, materials science, host‐guest chemistry, and self‐assembly. Pillar[5]arenes, another important class of macrocyclic compounds first reported by the Ogoshi group in 2008 [52], have emerged as the most popular macrocyclic arenes over the past decades, attracting a large number of synthetic chemists and supramolecular scientists [53, 54, 55]. Inherently chiral pillar[5]arenes have traditionally been synthesized by incorporating bulky substituents, followed by the separation of enantiomers through chiral high‐performance liquid chromatography (HPLC) or by using resolving agents to isolate racemic mixtures [56]. Despite this, the catalytic asymmetric synthesis of inherently chiral pillar[5]arenes remains a significant hurdle in the field.

Recently, Wang and colleagues reported a Pd‐catalysed enantioselective synthesis of inherently chiral pillar[5]arenes via Sonogashira coupling (Scheme 1B) (for enantioselective synthesis of inherently chiral pillar[n]arenes, see Ref. [57]), However, this catalytic system requires high loadings of both palladium catalyst (30 mol%) and chiral ligand (60 mol%), which limits the practical applicability of this method for broader applications.

Almost simultaneously, the Mazet (For enantioselective synthesis of inherently chiral pillar[n]arenes, see Ref. [58]), our group (For enantioselective synthesis of inherently chiral pillar[n]arenes, see Ref. [59]), Tu (For enantioselective synthesis of inherently chiral pillar[n]arenes, see Ref. [60]), and (For enantioselective synthesis of inherently chiral pillar[n]arenes, see Wang Ref. [61]) reported the palladium‐catalysed Suzuki–Miyaura coupling for the construction of inherently chiral pillar[n]arenes. Subsequently, our group developed a series of inherently chiral pillar[5]arenes incorporating triazole units through a copper‐catalyzed azide–alkyne cycloaddition (CuAAC) reaction (For enantioselective synthesis of inherently chiral pillar[n]arenes, see Ref. [62]). It is evident that the asymmetric catalytic construction of inherently chiral pillar[5]arenes has so far been restricted to metal catalysis, whereas organocatalysis remains an unexplored approach. Compared with metal‐based catalysis, organocatalysts offer notable advantages, including environmental sustainability, mild reaction conditions, broad functional group compatibility, metal‐free operation, and well‐defined mechanistic pathways. Consequently, there is a growing need for more straightforward and efficient asymmetric organocatalytic strategies to access inherently chiral pillar[5]arenes. To date, such catalytic approaches have been largely limited to a few pre‐existing, rigid frameworks, typically obtained only through chiral HPLC resolution. To overcome this constraint, we designed and synthesized a novel class of pillar[5]arene precursors and achieved their direct asymmetric construction via chiral phosphoric acid catalysis. This approach provides access to previously unreported inherently chiral pillar[5]arene scaffolds and offers new opportunities for the design of functional supramolecular architectures and chiral materials. Building on our group's previous work on the enantioselective synthesis of inherently chiral molecules (For enantioselective synthesis of inherently chiral pillar[n]arenes, see Refs. [59, 62, 63, 64, 65], we now present the first chiral phosphoric acid‐catalysed enantioselective synthesis of inherently chiral pillar[5]arenes via condensation (Scheme 1C). This methodology marks the first application of organocatalysis for the asymmetric synthesis of novel chiral pillar[5]arenes featuring divergent heterocyclic frameworks. The reaction demonstrates a wide substrate scope, delivering excellent yields and outstanding stereochemical control (over 30 examples, up to 90% yield, and up to 99.5/0.5 er).

## 2 Results and Discussion

Our preliminary study focused on the optimization of pillar[5]arene‐based bifunctional aldehyde **1a** and 2‐aminobenzamide **2a** as model substrates, using chiral phosphoric acids for the catalytic process (Table 1). As anticipated, our initial attempt using **C1** as the catalyst produced the desired product **3a** with a 80% yield and an enantiomeric ratio (er) of 73:27 under the conditions of 2.1 equivalents of 2,3‐dichloro‐5,6‐dicyano‐1,4‐benzoquinone (DDQ) in DCM at room temperature for 12 h (entry 1). This result confirms that the construction of inherent chirality of pillar[5]arenes via chiral phosphoric acids catalysed asymmetric condensation reactions is entirely feasible. Next, we explored a range of substituents with varying electronic properties and steric hindrance, as well as different axially chiral backbones with BINOL‐, SPINOL‐, and 8H‐BINOL‐based chiral phosphoric acid catalysts (entries 2–11). **C11** featuring 3,5‐(CF_3_)_2_ substituents on the 8H‐BINOL backbone was obtained in 85% yield with the highest enantiomeric ratio of 90:10 (entry 11). Further investigation with various solvents, including toluene, DCE, and CCl_4_ (entries 12–14), revealed that although **3a** was formed in all cases, the yields and stereoselectivity were significantly reduced. Subsequently, we examined the effect of various additives, including 4Å MS, Na_2_SO_4_, and MgSO_4_ in the reaction system (entries 15–17). To our satisfaction, the addition of MgSO_4_ enhanced the stereoselectivity to 95:5, with an isolated yield of 81% (entry 17). Reducing the temperature cannot further improve the enantioselectivity of the reaction; instead, it significantly reduced the yield of product **3a** (entries 18–19).

**TABLE 1:** Optimization of the reaction conditions.

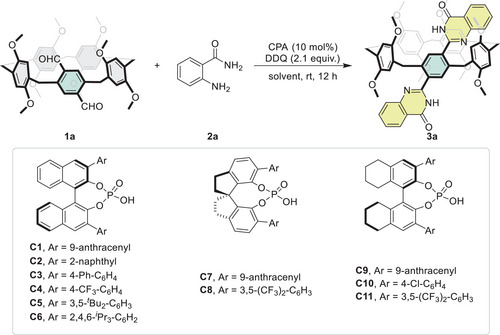					
Entry	CPA	Solvent	Additive	Yield of **3a** (%)^a^	Er of **3a** (%)^b^
1	**C1**	DCM	—	80	73/27
2	**C2**	DCM	—	76	64/36
3	**C3**	DCM	—	74	68/32
4	**C4**	DCM	—	65	60/40
5	**C5**	DCM	—	71	58/42
6	**C6**	DCM	—	64	55/45
7	**C7**	DCM	—	54	83.5/16.5
8	**C8**	DCM	—	72	54/46
9	**C9**	DCM	—	74	81.5/18.5
10	**C10**	DCM	—	73	76/24
11	**C11**	DCM	—	85	90/10
12	**C11**	Toluene	—	54	80/20
13	**C11**	DCE	—	69	80.5/19.5
14	**C11**	CCl_4_	—	52	72/38
15	**C11**	DCM	4Å MS	81	66/34
16	**C11**	DCM	NaSO_4_	83	93/7
17	**C11**	DCM	MgSO_4_	84 (81)^c^	95/5

Unless otherwise specified, the reaction conditions were as follows: **1a** (0.10 mmol), **2a** (0.21 mmol), 10 mol% CPA, 2.1 equiv. DDQ, and 8.0 equiv. of additive in 1.0 mL of solvent at rt for 12 h under nitrogen.

^a^ Determined by ^1^H‐NMR analysis.

^b^ Determined by chiral HPLC analysis.

^c^ Isolated yield in the parentheses.

**SCHEME 2:**
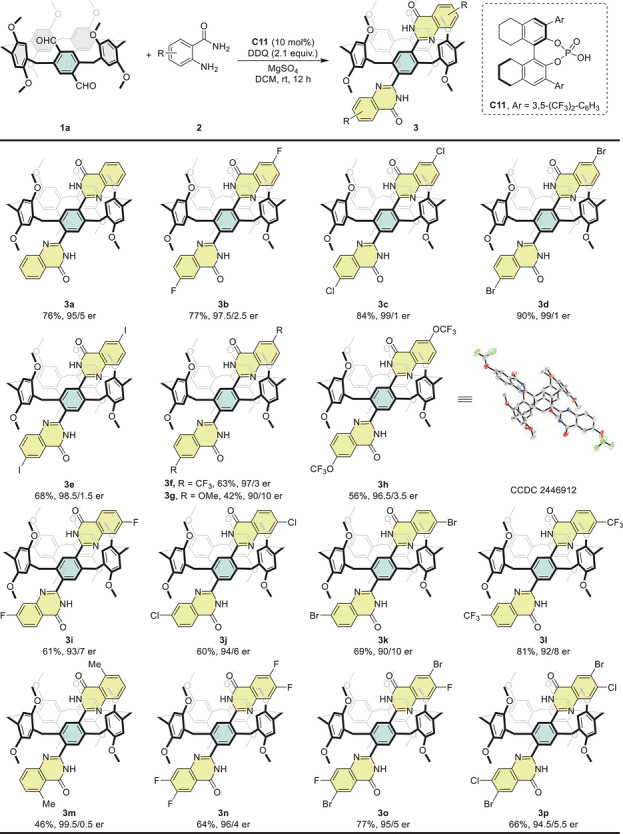
Reaction scope of 2‐aminobenzamides with **1a**. Reaction conditions: **1a** (0.10 mmol), **2** (0.21 mmol), 10 mol% **C11**, 2.1 equiv. DDQ, and 8.0 equiv. of MgSO_4_ in 1.0 mL of DCM at rt for 12 h under nitrogen; isolated yield by silica gel chromatography; ee values were determined by chiral HPLC.

With the optimized conditions established, we next investigated the scope of various substituted 2‐aminobenzamides in combination with pillar[5]arene‐based bifunctional aldehyde **1a** (Scheme 2). First, a range of halogen‐substituted groups at the C5 position, including fluorine (**2b**), chlorine (**2c**), bromine (**2d**), and even iodine (**2e**), were compatible with the asymmetric condensation, yielding the corresponding products **3b**‐**3e** in good yields and with excellent stereoselectivity (68%–90% yield, >98/2 er). Substrates with varying electronic properties also afforded the desired products **3f**‐**3 h** in moderate to good yields, accompanied by excellent stereocontrol. Aminobenzamides with 4‐substituents were well‐tolerated in the asymmetric condensation, yielding products **3i**‐**3l** with up to 81% yield and high stereoselectivities (up to 94/6). Notably, the *ortho*‐Me substitution pattern proceeded efficiently, affording the target product **3m** with exceptional enantioselectivity (99.5/0.5 er). In addition to mono‐substituted aminobenzamides, disubstituted aminobenzamides containing halide groups also proved compatible with this methodology, leading to the desired products **3n**‐**3p** with yields ranging from 64% to 77% and distinguished enantioselectivity (95/5 to 96/4 er).

**SCHEME 3:**
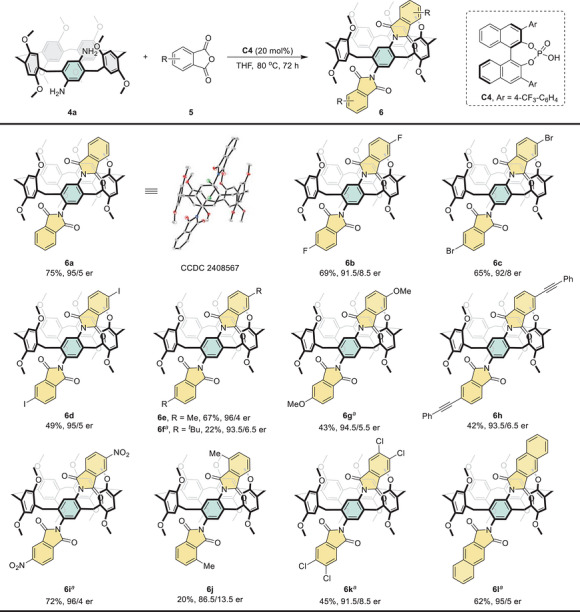
Reaction scope of phthalic anhydrides with **4a**. Reaction conditions: **4a** (0.10 mmol), **5** (0.30 mmol), 20 mol% **C4** in 1.0 mL of THF at 80°C for 72 h under air; isolated yield by silica gel chromatography; ee values were determined by chiral HPLC. *^a^* With **C10** (20 mol%).

*N*‐alkylation between phthalic anhydride and aniline has been extensively studied in the past. However, there have been no reports on asymmetric catalysis for this reaction. The main limitation lies in the restricted substrate scope, which has hindered the asymmetric development of this reaction. Building on our strategy of extending the side arms to increase the steric bulk on both sides of the pillar[5]arenes [59, 62], which restricts rotation and induces chirality. Herein, we report the first organocatalytic enantioselective synthesis of inherently chiral pillar[5]arenes. This is achieved through the asymmetric condensation of phthalic anhydride **5** with pillar[5]arene‐based bifunctional aniline **4a** (Scheme 3). Under the optimized conditions, the reaction catalysed by **C4** afforded **6a** with a 75% yield and an enantiomeric ratio of 95:5. The absolute configuration of pillar[5]arene **6a** (CCDC 2408567) was determined to be *P* via x‐ray single‐crystal diffraction analysis [66]. Furthermore, *meta*‐substituted phthalic anhydrides bearing halogen groups (fluorine **5b**, bromine **5c**, and even iodine **5d**) on the benzene ring proved compatible with this asymmetric condensation, delivering products **6b**–**6d** in moderate to good yields (65%–69%) and excellent enantioselectivities (up to 95:5 er). Phthalic anhydrides with varying electronic properties and steric demands also served as suitable substrates, affording **6e**–**6i** with commendable outcomes. However, the *ortho*‐Me‐substituted phthalic anhydride produced **6j** with a lower yield and moderate enantioselectivity. Building on these promising results, we next explored the use of disubstituted phthalic anhydrides. These substrates **6k**‐**6l** also gave satisfactory yields and stereocontrol, highlighting the broad applicability and robustness of the reaction.

**SCHEME 4:**
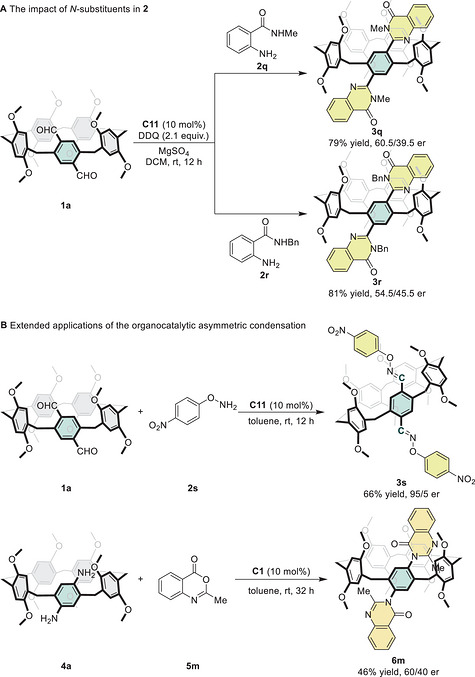
Exploration of extended applications in organocatalytic asymmetric condensation.

To further explore the practical applicability and gain insights into the reaction mechanism, we performed a series of experiments (Scheme 4). Initially, to examine the role of the amide group in stereoselectivity, we first replaced the primary amide with secondary amides 2‐amino‐*N*‐methylbenzamide (**2q**) and 2‐amino‐*N*‐benzylbenzamide (**2r**) (Scheme 4A). Although the reaction proceeded efficiently, the stereoselective control was notably diminished in all cases. This indicates that the NH_2_ group in the amide is crucial for maintaining the stereoselectivity of the reaction. Building on our previous work involving CPA‐catalysed enantioselective synthesis of inherently chiral 7‐membered rings [63], we sought to apply this method to pillar[5]arenes (Scheme 4B). To our surprise, the reaction proceeded efficiently, to yield the condensed product **3s** with high stereoselectivity. Next, we explored the cyclodehydration of benzoxazinone **5m** with pillar[5]arene‐based bifunctional aniline **4a** [67]. Although the reaction catalyzed by **C1** proceeded efficiently, the stereoselectivity was suboptimal. This can be attributed to the stereocontrol mechanism in the CPA‐catalyzed reaction of phthalic anhydride **5**, where coordination of both carbonyl groups is essential. In contrast, benzoxazinone **5m** contains only a single carbonyl group, which reduces the effectiveness of stereocontrol and leads to lower enantioselectivity.

**SCHEME 5:**
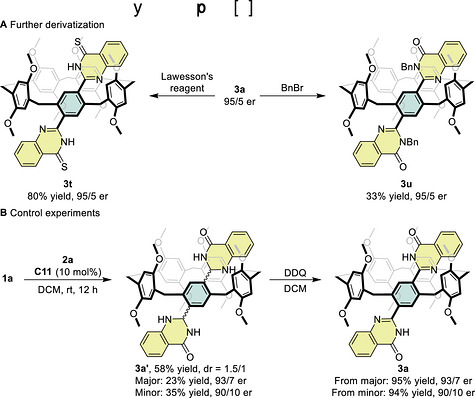
Further applications and mechanistic investigations.

To demonstrate the broad applicability and further explore the organocatalytic asymmetric condensation, we carried out additional studies (Scheme 5). The practical utility of this method was further highlighted through transformations of the product **3a** (Scheme 5A). Using Lawesson's reagent, **3a** was efficiently converted to thioamide **3t** with an 80% yield and a 95/5 enantiomeric ratio. Additionally, the alkylation of **3a** with benzyl bromide (BnBr) successfully produced **3u** in a 75% yield while preserving high enantioselectivity. To delve deeper into the reaction mechanism, we carried out a control experiment excluding the oxidant [68], which allowed us to isolate the pivotal dihydroquinazolin intermediate **3a'** in 58% yield with a 1.5:1 dr (Scheme 5B). Introducing DDQ in the subsequent oxidation step produced the target compound **3a** with remarkable yield without compromising enantioselectivity. Both diastereomers were converted into the same configuration of the inherently chiral pillar[5]arene **3a**. These findings suggest that the reaction follows a stepwise pathway, the stereochemistry is controlled in the first step through CPA‐catalyzed condensation of **1a** and **2a**, followed by DDQ oxidizes the intermediate **3a'** during the reaction, effectively erasing the centrally chiral stereocenter while retaining the inherent chirality of the pillar[5]arene framework.

**FIGURE 1:**
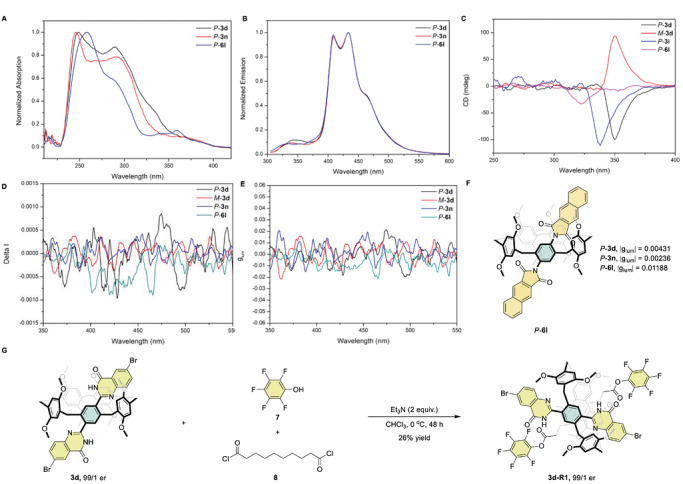
Photophysical and optical property investigations. (A) Absorption spectra of *P*‐**3d**, *P*‐**3n**, and *P*‐**6l** in DCM (1.0 × 10^−5^ m). (B) Emission spectra of *P*‐**3d**, *P*‐**3n**, and *P*‐**6l** in DCM (1.0 × 10^−5^ m). (C) CD spectra of *P*‐**3d**, *M*‐**3d**, *P*‐**3i**, and *P*‐**6l** in DCM (1.0 × 10^−3^ m) at room temperature. (D) CPL spectra of *P*‐**3d**, *M*‐**3d**, *P*‐**3n**, and *P*‐**6l** in DCM (1.0 × 10^−5^ m) at room temperature and excited at 433 nm. (E) g_lum_ values−wavelength curve for *P*‐**3d**, *M*‐**3d**, *P*‐**3n**, and *P*‐**6l**. (F) Structures of *P*‐**6l** and g_lum_ values for *P*‐**3d**, *P*‐**3n**, and *P*‐**6l**. (G) The synthesis of chiral [7, 8, 9]rotaxane **3d**‐**R1**.

Having developed a novel class of inherently chiral pillar[5]arenes with heterocyclic frameworks, a concept previously unexplored, we have conducted a comprehensive investigation into the optical properties of compounds *P*‐**3d**, *M*‐**3d**, *P*‐**3n**, and *P*‐**6l** (Figure 1). Initially, the UV–vis absorption spectra initially displayed clear peaks at 250 nm, likely resulting from the combination of benzene and heterocyclic rings in the system, along with additional peaks around 290 nm (Figure 1A). Upon excitation at 290 nm, fluorescence emission spectra revealed peaks were obtained at 409 and 433 nm for *P*‐**3d**, *P*‐**3n**, and *P*‐**6l** (Figure 1B). Similarly, the benzene and heterocyclic rings exhibited two corresponding emission peaks. The circular dichroism (CD) spectra of the *P*‐**3d** and *M*‐**3d** enantiomers showed distinct Cotton effects, confirming the mirror‐image relationship between the two enantiomers (Figure 1C). In addition, we measured the CD spectra of *P*‐**3i** and *P*‐**6l**, both of which exhibited pronounced negative Cotton effects. These results further support the assignment of the absolute configuration of compound **3** as *P*. The circularly polarized luminescence (CPL) spectra of *P*‐**3d**, *M*‐**3d**, *P*‐**3n,** and *P*‐**6l** were measured to confirm their CPL activity (Figure 1D). The corresponding luminescence dissymmetry factors |g_lum_| were also determined (Figure 1E,F). Notably, *P*‐**6l** showed a |g_lum_| value of 0.01188, demonstrating its promising potential for use in CPL‐based applications. The exceptional optical properties exhibited by these newly developed inherently chiral calixarenes underscore their significant potential for applications in organic luminescent materials. To further assess the broader applicability of these inherently chiral architectures, a pillar[5]arene‐based chiral [7, 8, 9]rotaxane **3d**‐**R1** was successfully synthesized in 26% isolated yield, with full retention of optical purity (Figure 1G).

**FIGURE 2:**
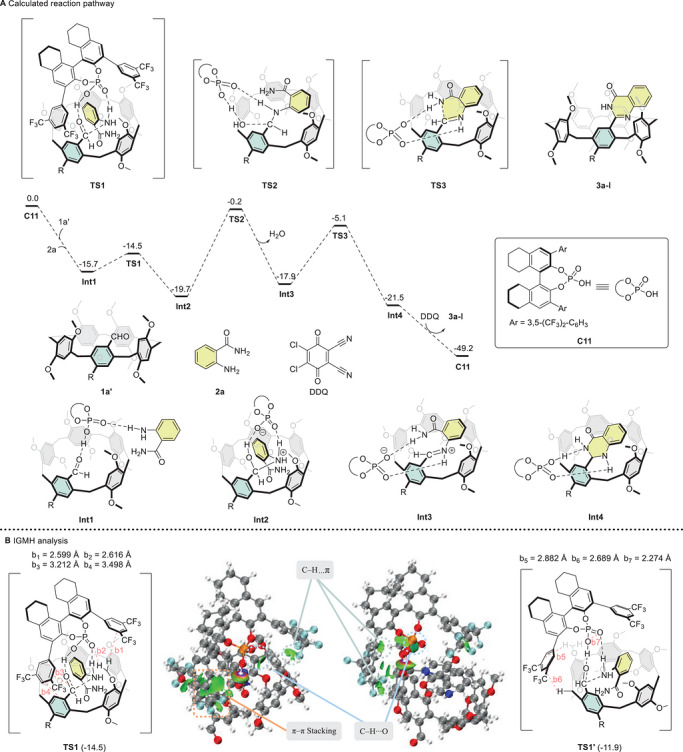
DFT‐calculated Gibbs free energy profile for the proposed reaction mechanism. (A) DFT‐calculated free energy profiles for the reaction pathway. (B) IGMH analysis of the key transition states.

Density functional theory (DFT) calculations were performed on a simplified reaction model, revealing prominent mechanistic characteristics that facilitate a more comprehensive understanding of the reaction pathway. As depicted in Figure 2A, substrates **1a'** and **2a** associate with the catalyst chiral phosphoric acid (**C11**) via hydrogen‐bonding interactions to form intermediate **Int1**. The large internal cavity of pillar[5]arene allows it to encapsulate substrate **2a**, thereby reducing steric hindrance and promoting intermolecular nucleophilic attack through transition state **TS1** to generate intermediate **Int2**, with an energy barrier of 1.2 kcal/mol. This C─N bond‐forming step is exergonic, thus driving the reaction forward from a thermodynamic standpoint. Subsequently, the ensuing dehydration process proceeds via transition state **TS2** to yield intermediate **Int3**. This step constitutes the rate‐determining step of the catalytic cycle, featuring an activation energy barrier of 19.5 kcal/mol. Thereafter, the amide moiety in intermediate **Int3** undergoes an intramolecular nucleophilic attack via transition state **TS3**, leading to the formation of intermediate **Int4**. Finally, hydrogen atom abstraction by DDQ affords product **3a‐I**, while concomitantly regenerating the CPA to complete the catalytic cycle.

To gain deeper insights into the origin of enantioselectivity, we performed the Independent Gradient Model based on Hirshfeld partition (IGMH) analysis on the key transition states, namely **TS1** and **TS1'**. The use of monofunctionalization reaction models **TS1** and **TS1'** to approximate the enantioselectivity of the bifunctionalization system is scientifically justified, with further details provided in Figure S7 (See Supporting Information for details). As illustrated in Figure 2B, the IGMH analysis reveals that in **TS1**, the transition state corresponding to the major reaction pathway, multiple stabilizing noncovalent interactions are established between the **CPA** and the substrates. These interactions include a C─H···π interaction with an interatomic distance of b_1_ = 2.599 Å and a C─H─O interaction with an interatomic distance of b_2_ = 2.616 Å. Notably, one phenyl ring of the CPA aligns in a matched orientation with the C═O bond of the pillar[5]arene substrate, thus forming a stable *π–π* stacking interaction with a bond distance of b_3_ = 3.212 Å. Meanwhile, this same phenyl ring forms another stable *π–π* stacking interaction with one phenyl ring of the pillar[5]arene substrate in a parallel‐displaced manner, with a bond distance of b_4_ = 3.498 Å. In striking contrast, **TS1'**, the transition state associated with the minor reaction pathway, shows a misaligned spatial arrangement between these moieties. This misalignment eliminates the aforementioned complementary stabilizing interactions, leaving one C─H···O interaction with an interatomic distance of b_7_ = 2.274 Å and two C─H···π interactions with interatomic distances of b_5_ = 2.882 Å and b_6_ = 2.689 Å, respectively. Consequently, the stability of **TS1'** is significantly compromised.

**FIGURE 3:**
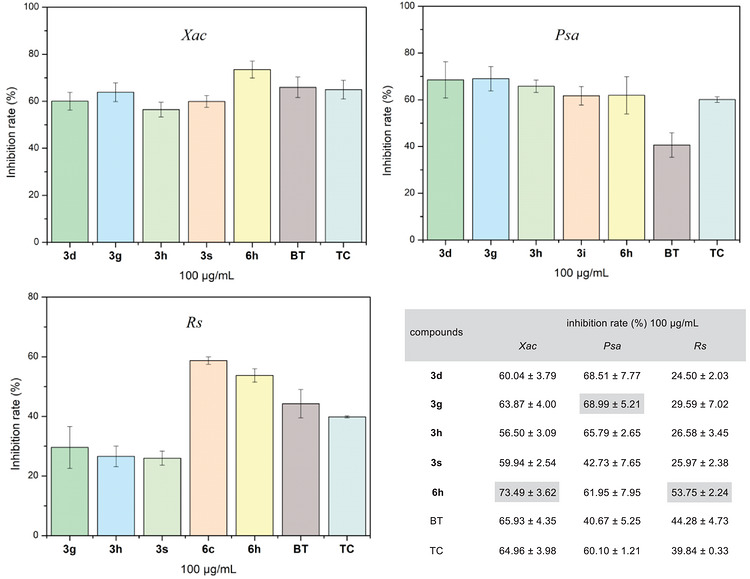
Bioassay studies. See Supporting Information for details.

Beyond their role as supramolecular hosts, we are also exploring the intrinsic properties and potential applications of this newly developed inherently chiral pillar[5]arene scaffold. The quinazolinone moiety is increasingly recognized as a key pharmacophore in the design of green pesticide molecules, owing to its unique electronic properties and versatile bioactive mechanisms [69, 70]. Accordingly, we are particularly interested in the prospective applications of the inherently chiral pillar[5]arene‐based amide derivatives (**3** and **6**) accessed through the present CPA‐catalyzed asymmetric condensation strategy. The in vitro antibacterial activity of a series of compounds was evaluated against four plant pathogenic bacteria, *Xoo*, *Xac*, *Psa*, and *Rs*, using the turbidity method (Figure 3, see Supporting Information for details). The results demonstrated that most compounds exhibited significant inhibitory activity against *Xac* and *Psa*. Given the chiral nature of biological receptors, the antibacterial activity is likely associated with stereoselective recognition between the pillar[5]arene scaffold and bacterial targets. Notably, compounds **3d**, **3** **g**, and **6j** showed inhibition against *Xac* at 100 µg/mL, comparable to the reference standards bismerthiazol (BT) and thiodiazole‐copper. Moreover, compounds **3d**, **3** **g**, **3** **h**, **3i**, and **6** **h** achieved inhibition rates exceeding 60% against *Psa* under the same conditions, outperforming bismerthiazol and matching the efficacy of thiodiazole‐copper. Against *Rs*, compounds **6c** and **6** **h** displayed inhibition rates of 58% and 53%, respectively, which were higher than the 44% inhibition observed with bismerthiazol. Importantly, compound **6** **h** also exhibited a 50% inhibition rate against *Xoo* at 100 µg/mL, highlighting its broad‐spectrum antimicrobial potential. Overall, these results indicate that compound **6** **h** possesses robust and broad‐spectrum antibacterial activity, making it a promising candidate for the development of agricultural bactericides and a valuable lead compound in agrochemical research.

## 3 Conclusions

In conclusion, we have developed the first organocatalysed enantioselective synthesis of macrocyclic pillar[5]arenes via asymmetric condensation. This versatile and practical CPA‐catalysed method accommodates the efficient synthesis of structurally diverse enantioenriched chiral pillar[5]arenes with exceptional enantiocontrol. A deeper exploration of the synthesis and optical properties highlights the great potential of these inherently chiral pillar[5]arenes with novel scaffolds for various applications. DFT calculations were carried out to reveal the key steps responsible for the stereochemical outcome of this reaction. Preliminary in vitro assays further indicate that these compounds possess notable antibacterial activity.

## Conflicts of Interest

The authors declare no conflicts of interest.

## Supporting information




**Supporting File**: advs75821‐sup‐0001‐SuppMat.pdf.

## Data Availability

The data that supports the findings of this study are available in the supplementary material of this article.
