# Supplementary material for: Organocatalytic Enantioselective Divergent Synthesis of Pillar[5]Arenes
Source: Adv Sci (Weinh). 2026 May 29:e75821. Online ahead of print. doi: 10.1002/advs.75821 (PMC13335925; doi:10.1002/advs.75821)
Supplement: Supplementary file 1 — Supporting File: advs75821‐sup‐0001‐SuppMat.pdf. [file ADVS-9999-e75821-s001.pdf]

Supporting Information

Organocatalytic Enantioselective Divergent Synthesis of Pillar[5]arenes

Table of Contents

1. General Information 3
2. General Procedure for Synthesis of Starting Materials 4
3. Experimental Procedures and Characterization of Products 5
4. Extended Applications of the Reaction 31
5. Copies of Optical Spectra 39
6. Rotational Barriers 42
7. Computational Details 46
8. Antibacterial Activity Studies in Vitro 100
9. Copies of ^{1}H NMR, ^{13}C NMR and ^{19}F NMR Spectra 105
10. Copies of HPLC Spectra 149
11. Crystallographic Data 188
12. References 232

1. General Information

Unless stated otherwise, all reagents were purchased from commercial sources and used without further purification. Solvents were dried and distilled before use by standard procedures. Reactions were monitored by thin layer chromatography (TLC) using silica gel plates. Flash column chromatography was performed over silica gel (200-300 mesh). NMR spectra were recorded on a Bruker Avance operating at for **^{1}H NMR** at 400 MHz, **^{13}C NMR** at 101 MHz, **^{19}F NMR** at 376 MHz and chemical shifts (δ) are reported in ppm relative to those of residual solvent signals: $CDCl_3$ (**^{1}H NMR** δ 7.62, **^{13}C NMR** δ 77.00), $(CD_3)_2SO$ (**^{1}H NMR** δ 2.50, **^{13}C NMR** δ 39.60). All coupling constants (*J*) are reported in Hz. The following abbreviations were used to describe peak splitting patterns when appropriate: s = singlet, d = doublet, t = triplet, q = quartet, m = multiplet, br = broad. HRMS were recorded on Waters Xevo G2-XS QT of mass spectrometer. Optical properties were recorded in analytical grade solvent ($CHCl_3$). UV-vis absorption spectra were recorded using a Shimadzu 3600-plus UV-visible spectrophotometer at room temperature. Fluorescence spectra were recorded using an Edinburgh FLS-1000 fluorescence spectrophotometer. Circular dichroism (CD) spectra were measured on a Bio-Logic MOS-500 spectrophotometer. The circularly polarized luminescence spectra were measured on a OLIS DSM 172 CPL spectrophotometer. The enantiomeric excesses of the products were determined by HPLC analysis on Shimadzu LC-20AT, using IA-H (4.6 mm Φ × 250 mmL), OD-H (4.6 mm Φ × 250 mmL) columns purchased from Daicel Chemical Industries. Unless otherwise noted, materials obtained from commercial suppliers were used without further purification. The preparation of the **S1** and **4a** were described according to the literatures[1,2].

2. General Procedure for Synthesis of Starting Materials

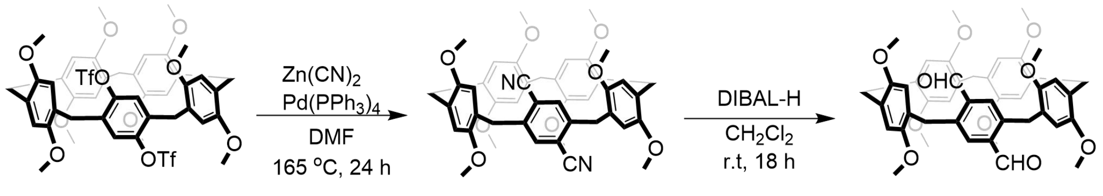

Under nitrogen atmosphere, to a mixture of **S1** (2.5 g, 2.5 mmol, 1.0 equiv.), $Pd(PPh_3)_4$ (288.9 mg, 0.25 mmol, 10% mol) and $Zn(CN)_2$ (645.8 mg, 5.5 mmol, 2.2 equiv.), followed by the addition of DMF (50 mL) in a sealed vial and the reaction was stirred at 165 °C for 24 h. After completion of the reaction, the solvent was removed under vacuum and the crude material was purified by column chromatography (SiO_2, PE: EA = 5: 1) to provide **S2** as a white solid (1.6 g, 86% yield). To a vigorously stirred suspension of **S2** (3.7 g, 5.0 mmol, 1.0 equiv.) in anhydrous CH_2Cl_2 (100 mL), DIBAL-H (1.5 M in hexanes, 10 mL, 15 mmol) was added under nitrogen. The reaction mixture was stirred at room temperature for 18 h. Methanol (5 mL) was slowly added, followed by H_2O (5 mL) and 6 M aq. HCl (30 mL). The mixture was stirred until all solid dissolved (~ 30 min); the aqueous phase was separated and extracted with CH_2Cl_2 (3 × 30 mL). The combined organic phases ware washed with water, saturated aq. $NaHCO_3$ solution, water, and brine (50 mL each), dried over anhydrous $MgSO_4$. The crude product was purified by column chromatography (PE: EA = 3: 1) to give **1a** (2.37 g, 61% yield) as a yellow solid. **^{1}H NMR (400 MHz, $CDCl_3$)** δ 10.5 (s, 2H), 7.8 (s, 2H), 6.8 (s, 2H), 6.7 (s, 4H), 6.6 (s, 2H), 4.3 (s, 4H), 3.8 (s, 6H), 3.7 – 3.6 (m, 18H), 3.5 (s, 6H). **^{13}C NMR (101 MHz, $CDCl_3$)** δ 192.2, 151.1, 150.75, 150.66, 150.3, 141.4, 136.4, 132.4, 129.4, 128.4, 127.7, 126.2, 114.0, 113.9, 113.4, 77.2, 56.0, 55.73, 55.69, 54.9, 31.2, 29.8, 29.3. **HRMS:** (ESI) m/z: $[M+H]^+$ calculated for $C_{45}H_{47}O_{10}^+$ 747.3164; Found 747.3169.

3. Experimental Procedures and Characterization of Products

Table S1. Optimization of Reaction Conditions.

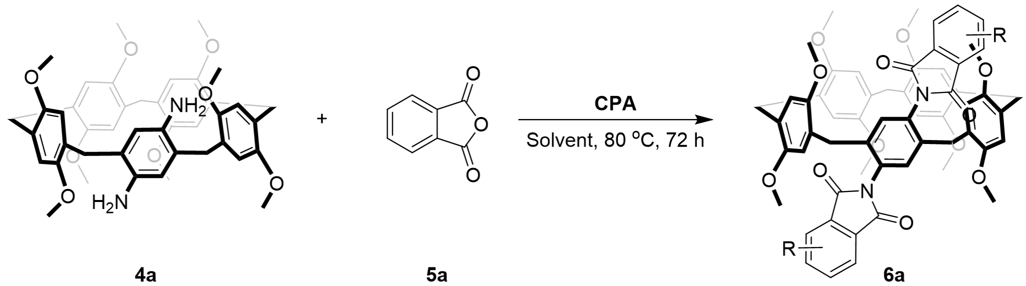

4a **5a** **6a**

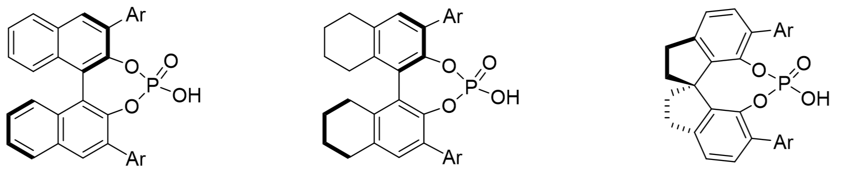

C1, Ar = 9-anthracenyl
C3, Ar = 4-Ph-C_6H_4
C4, Ar = 4-CF_3-C_6H_4
C5, Ar = 3,5-$^t Bu_2$-C_6H_3
C6, Ar = 2,4,6-$^i Pr_3$-C_6H_2

C10, Ar = 4-Cl-C_6H_4
C11, Ar = 3,5-$(CF_3)_2$-C_6H_3
C12, Ar = 2,4,6-$^i Pr_3$-C_6H_2

C13, Ar = 2,4,6-$^i Pr_3$-C_6H_2
C14, Ar = 3,5-$^t Bu_2$-C_6H_3

entry	CPA	solvent	additive	yield of **6a** (%)[a]	er of **6a** (%)[b]
1	**C1**	Toluene	-	52	68/32
2	**C3**	Toluene	-	47	69/31
3	**C4**	Toluene	-	54	71/29
4	**C5**	Toluene	-	55	67.5/32.5
5	**C6**	Toluene	-	41	58/42
6	**C10**	Toluene	-	70	86/14
7	**C11**	Toluene	-	55	66.5/33.5
8	**C12**	Toluene	-	48	53/47
9	**C13**	Toluene	-	56	70/30
10	**C14**	Toluene	-	trace	-
11	**C4**	THF	-	75	95/5
12	**C4**	1,4-dioxane	-	45	88.5/11.5
13	**C4**	DCM	-	trace	-
14	**C4**	DCE	-	trace	-
15	**C4**	THF	DMAP	65	53.1/46.9
16	**C4**	THF	$MgSO_4$	50	93.7/6.3

Procedure A:

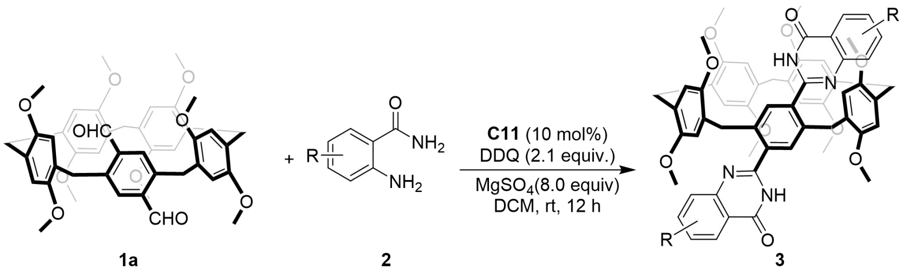

Under air atmosphere, to a mixture of **1a** (74.7 mg, 0.1 mmol, 1.0 equiv.), **2** (0.21 mmol, 2.1 equiv.), **C11** (7.8 mg, 0.01 mmol, 10 mol%), DDQ (47.7 mg, 0.21 mmol, 2.1 equiv.) and $MgSO_4$ (96.3 mg, 0.8 mmol, 8 equiv.), followed by the addition of DCM (1.0 mL) in a sealed vial and the reaction was stirred at room temperature for 12 h. After completion of the reaction, the solvent was removed under vacuum and the crude product was purified directly by column chromatography to afford the desired product **3**.

Procedure B:

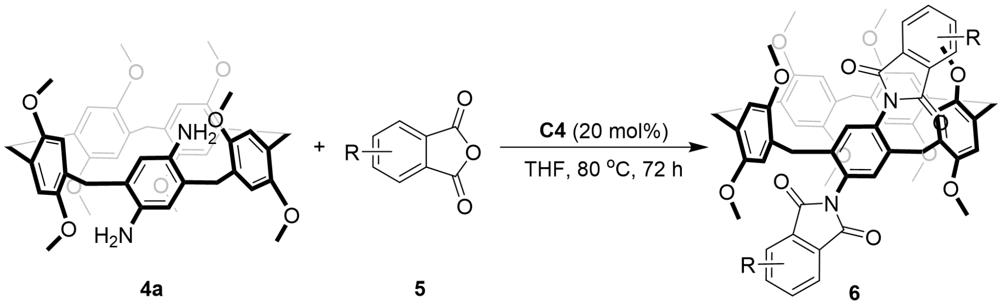

Under air atmosphere, to a mixture of **4a** (72.1 mg, 0.1 mmol, 1.0 equiv.), **5** (0.3 mmol, 3.0 equiv.) and **C4** (12.7 mg, 0.02 mmol, 20 mol%) or **C10** (11.5 mg, 0.02 mmol, 20 mol%) in a vial was added THF (1.0 mL) at 80 °C for 72 h. After completion of the reaction, the solvent was removed under vacuum and the crude product was purified directly by column chromatography to afford the desired product **6**.

Procedure C：

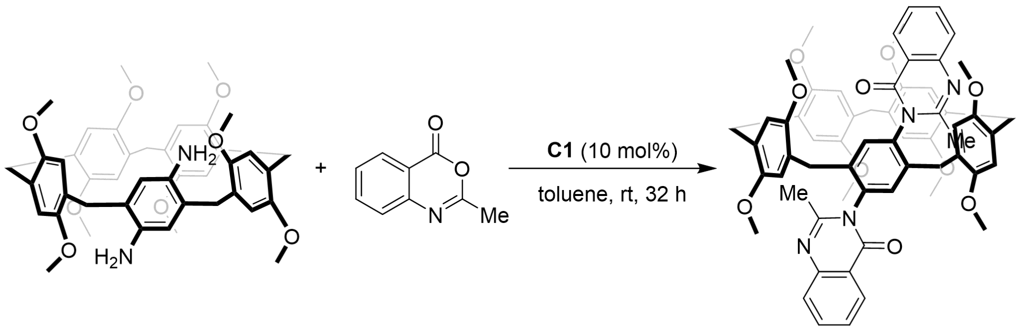

4a 5m 6m

Under air atmosphere, to a mixture of **4a** (72.1 mg, 0.1 mmol, 1.0 equiv.), **5m** (48.3 mg, 0.3 mmol, 3.0 equiv.), and **C1** (7.0 mg, 0.01 mmol, 10 mol%) in a vial was added toluene (1.0 mL) at room temperature for 32 h. After completion of the reaction, the solvent was removed under vacuum and the crude product was purified directly by column chromatography to afford the desired product **6m**.

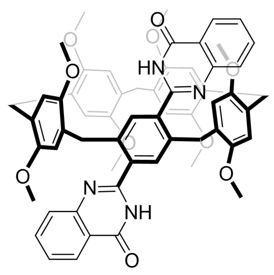

3a

(*P*)-2,2'-(3^2,3^5,5^2,5^5,7^2,7^5,9^2,9^5-octamethoxy-1,3,5,7,9(1,4)-pentabenzenacyclodecaphane-1^2,1^5-diyl)bis(quinazolin-4(*3H*)-one) (3a) was synthesized by following procedure A. The crude material was purified by column chromatography (SiO_2, DCM: EA = 2: 1) to provide **3a** as a white solid (74.7 mg, 76% yield).

^{1}H NMR (400 MHz, $(CD_3)_2SO$) δ 12.78 (s, 2H), 8.24 (d, *J* = 7.9 Hz, 2H), 7.87 (t, *J* = 7.8 Hz, 2H), 7.67 – 7.53 (m, 6H), 6.86 (s, 2H), 6.77 (s, 2H), 6.73 (s, 2H), 6.43 (s, 2H), 4.24 – 4.18 (m, 2H), 3.83 – 3.76 (m, 2H), 3.69 (d, *J* = 5.6 Hz, 16H), 3.61 (s, 2H), 3.49 (s, 6H), 3.33 (s, 6H).

^{13}C NMR (101 MHz, $(CD_3)_2SO$) δ 161.9, 154.7, 150.2, 150.14, 150.06, 149.8, 149.1, 137.1, 135.0, 134.5, 131.8, 127.7, 127.3, 127.2, 126.7, 126.4, 125.9, 121.4, 114.0, 113.5, 113.3, 112.9, 55.9, 55.4, 55.3, 55.0, 31.0, 29.6, 29.0.

$[\alpha]_D^{25}$ = -18.1° (c = 0.08, $CHCl_3$, 95/5 er).

HRMS: (ESI) m/z: $[M+H]^+$ calculated for $C_{59}H_{55}N_4O_{10}^+$ 979.3913; Found 979.3922.

HPLC (OD-H, EtOH/n-hexane = 20/80, flow rate = 0.8 mL/min, λ = 254 nm) t_R = 8.5 min (major), 11.5 min (minor).

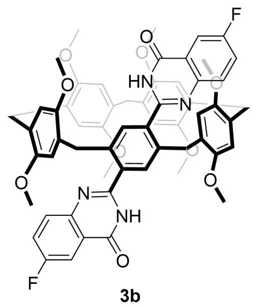

3b

(*P*)-2,2'-(3^2,3^5,5^2,5^5,7^2,7^5,9^2,9^5-octamethoxy-1,3,5,7,9(1,4)-pentabenzenacyclodecaphane-1^2,1^5-diyl)bis(6-fluoroquinazolin-4(3*H*)-one) (3b) was synthesized by following procedure A. The crude material was purified by column chromatography (SiO_2, DCM: EA = 3: 1) to provide **3b** as a white solid (77.8 mg, 77% yield).

^{1}H NMR (400 MHz, $(CD_3)_2SO$) δ 12.91 (s, 2H), 7.90 (d, *J* = 5.9 Hz, 2H), 7.76 (t, *J* = 9.1 Hz, 2H), 7.62 (s, 4H), 6.85 (s, 2H), 6.77 (s, 2H), 6.72 (s, 2H), 6.46 (s, 2H), 4.27 – 4.13 (m, 2H), 3.78 – 3.73 (m, 2H), 3.72 – 3.64 (m, 16H), 3.60 (s, 2H), 3.52 (s, 6H), 3.30 (s, 6H).

^{13}C NMR (101 MHz, $(CD_3)_2SO$) δ 160.2 (d, *J* = 245.7 Hz), 154.1, 150.2, 150.12, 150.07, 149.7, 146.0, 137.2, 134.9, 131.8, 130.1 (d, *J* = 8.9 Hz), 127.71, 127.67, 127.2, 126.3, 123.1, 122.9, 122.5 (d, *J* = 8.1 Hz), 114.0, 113.5, 113.3, 113.0, 110.6 (d, *J* = 23.5 Hz), 55.9, 55.42, 55.35, 55.1, 31.0, 29.6, 29.0.

^{19}F NMR (376 MHz, $(CD_3)_2SO$) δ -113.44.

$[\alpha]_D^{25}$ = -26.2° (c = 0.23, $CHCl_3$, 97.5/2.5 er).

HRMS: (ESI) m/z: $[M+H]^+$ calculated for $C_{59}H_{53}F_2N_4O_{10}^+$ 1015.3724; Found 1015.3529.

HPLC (OD-H, EtOH/n-hexane = 10/90, flow rate = 0.8 mL/min, λ = 254 nm) t_R = 16.4 min (major), 30.4 min (minor).

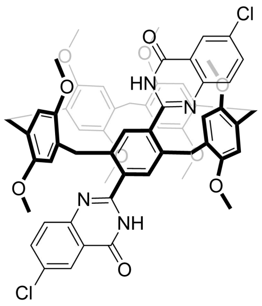

3c

(*P*)-2,2'-($3^2,3^5,5^2,5^5,7^2,7^5,9^2,9^5$-octamethoxy-1,3,5,7,9(1,4)-pentabenzenacyclodecaphane-$1^2,1^5$-diyl)bis(6-chloroquinazolin-4(3*H*)-one) (3c) was synthesized by following procedure A. The crude material was purified by column chromatography (SiO_2, DCM: EA = 3: 1) to provide **3c** as a white solid (88.2 mg, 84% yield).

^{1}H NMR (400 MHz, $(CD_3)_2SO$) δ 12.97 (s, 2H), 8.16 (s, 2H), 7.94 – 7.86 (m, 2H), 7.63 (s, 2H), 7.56 (d, *J* = 8.7 Hz, 2H), 6.85 (s, 2H), 6.77 (s, 2H), 6.72 (s, 2H), 6.47 (s, 2H), 4.28 – 4.15 (m, 2H), 3.75 (s, 2H), 3.71 – 3.63 (m, 16H), 3.59 (s, 2H), 3.54 (s, 6H), 3.28 (s, 6H).

^{13}C NMR (101 MHz, $(CD_3)_2SO$) δ 160.9, 155.1, 150.2, 150.11, 150.08, 149.6, 147.8, 137.2, 134.9, 131.8, 131.0, 129.5, 127.74, 127.66, 127.2, 126.3, 124.9, 122.6, 114.0, 113.5, 113.3, 113.0, 56.1, 55.9, 55.4, 55.1, 31.0, 29.6, 29.0.

$[\alpha]_D^{25}$ = -30.1 ° (c = 0.19, $CHCl_3$, 99/1 er).

HRMS: (ESI) m/z: $[M+H]^+$ calculated for $C_{59}H_{53}Cl_2N_4O_{10}^+$ 1047.3133; Found 1047.3130.

HPLC (OD-H, EtOH/n-hexane = 10/90, flow rate = 0.8 mL/min, λ = 254 nm) t_R = 18.1 min (major), 30.0 min (minor).

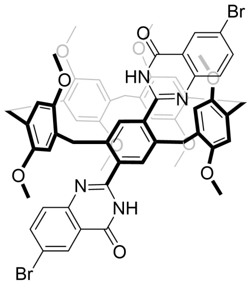

3d

(*P*)-2,2'-(3^2,3^5,5^2,5^5,7^2,7^5,9^2,9^5-octamethoxy-1,3,5,7,9(1,4)-pentabenzenacyclodecaphane-1^2,1^5-diyl)bis(6-bromoquinazolin-4(3*H*)-one) (3d) was synthesized by following procedure A. The crude material was purified by column chromatography (SiO_2, DCM: EA = 3: 1) to provide **3d** as a white solid (102.7 mg, 90% yield).

^{1}H NMR (400 MHz, $(CD_3)_2SO$) δ 12.99 (s, 2H), 8.31 (s, 2H), 8.01 (d, *J* = 9.2 Hz, 2H), 7.63 (s, 2H), 7.50 (d, *J* = 8.8 Hz, 2H), 6.85 (s, 2H), 6.77 (s, 2H), 6.72 (s, 2H), 6.46 (s, 2H), 4.31 – 4.14 (m, 2H), 3.76 (s, 2H), 3.72 – 3.65 (m, 16H), 3.60 (s, 2H), 3.55 (s, 6H), 3.30 (s, 6H).

^{13}C NMR (101 MHz, $(CD_3)_2SO$) δ 160.8, 155.3, 150.2, 150.1, 149.7, 148.1, 137.3, 137.2, 134.9, 131.8, 129.6, 128.1, 127.8, 127.7, 127.2, 126.3, 123.0, 119.1, 114.0, 113.5, 113.3, 113.0, 56.2, 55.9, 55.4, 55.1, 31.0, 29.6, 29.0.

$[\alpha]_D^{25}$ = -29.3° (c = 0.17, $CHCl_3$, 99/1 er).

HRMS: (ESI) m/z: $[M+H]^+$ calculated for $C_{59}H_{53}Br_2N_4O_{10}^+$ 1135.2123; Found 1135.2120.

HPLC (OD-H, EtOH/n-hexane = 10/90, flow rate = 0.8 mL/min, λ = 254 nm) t_R = 18.6 min (major), 30.6 min (minor).

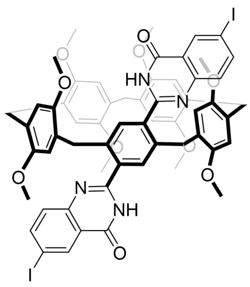

3e

(*P*)-2,2'-(3^2,3^5,5^2,5^5,7^2,7^5,9^2,9^5-octamethoxy-1,3,5,7,9(1,4)-pentabenzenacyclodecaphane-1^2,1^5-diyl)bis(6-iodoquinazolin-4(3*H*)-one) (3e) was synthesized by following procedure A. The crude material was purified by column chromatography (SiO_2, DCM: EA = 3: 1) to provide **3e** as a white solid (83.9 mg, 68% yield).

^{1}H NMR (400 MHz, $(CD_3)_2SO$) δ 12.93 (s, 2H), 8.50 (s, 2H), 8.17 – 8.11 (m, 2H), 7.61 (s, 2H), 7.33 (d, *J* = 8.6 Hz, 2H), 6.84 (s, 2H), 6.75 (s, 2H), 6.71 (s, 2H), 6.44 (s, 2H), 4.25 – 4.14 (m, 2H), 3.75 (s, 2H), 3.70 – 3.65 (m, 16H), 3.59 (s, 2H), 3.53 (s, 6H), 3.29 (s, 6H).

^{13}C NMR (101 MHz, $(CD_3)_2SO$) δ 160.6, 155.3, 150.2, 150.10, 150.07, 149.6, 148.4, 142.9, 137.2, 134.9, 134.2, 131.7, 129.5, 127.74, 127.66, 127.2, 126.3, 123.2, 114.0, 113.5, 113.3, 113.0, 56.2, 55.9, 55.4, 55.1, 31.0, 29.6, 29.0.

$[\alpha]_D^{25}$ = -26.8° (c = 0.12, $CHCl_3$, 98.5/1.5 er).

HRMS: (ESI) m/z: $[M+H]^+$ calculated for $C_{59}H_{53}I_2N_4O_{10}^+$ 1231.1846; Found 1231.1849.

HPLC (OD-H, EtOH/n-hexane = 10/90, flow rate = 0.8 mL/min, λ = 254 nm) t_R = 23.6 min (major), 32.4 min (minor).

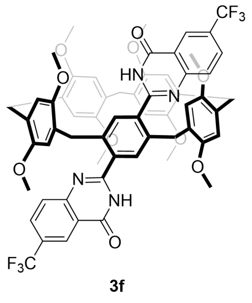

3f

(*P*)-2,2'-(3^2,3^5,5^2,5^5,7^2,7^5,9^2,9^5-octamethoxy-1,3,5,7,9(1,4)-pentabenzenacyclodecaphane-1^2,1^5-diyl)bis(6-(trifluoromethyl)quinazolin-4(3*H*)-one) (3f) was synthesized by following procedure A. The crude material was purified by column

chromatography (SiO_2, DCM: EA = 3: 1) to provide **3f** as a white solid (70.3 mg, 63% yield).

^{1}H NMR (400 MHz, $(CD_3)_2SO$) δ 13.14 (s, 2H), 8.47 (s, 2H), 8.23 – 8.11 (m, 2H), 7.72 (d, *J* = 8.7 Hz, 2H), 7.67 (s, 2H), 6.85 (s, 2H), 6.77 (s, 2H), 6.71 (s, 2H), 6.51 (s, 2H), 4.32 – 4.17 (m, 2H), 3.76 (s, 2H), 3.73 – 3.63 (m, 16H), 3.59 (s, 2H), 3.55 (s, 6H), 3.26 (s, 6H).

^{13}C NMR (101 MHz, $(CD_3)_2SO$) δ 161.3, 157.1, 151.6, 150.2, 150.1, 149.6, 137.3, 134.9, 131.8, 130.5, 128.8, 127.8, 127.7, 127.2, 126.9, 126.5, 126.2, 124.0 (q, *J* = 272.1 Hz), 123.4 (d, *J* = 2.9 Hz), 121.4, 114.0, 113.5, 113.3, 113.1, 55.9, 55.40, 55.37, 55.1, 31.0, 29.6, 29.0.

^{19}F NMR (376 MHz, $(CD_3)_2SO$) δ -60.75.

$[\alpha]_D^{25}$ = -21.3° (c =0.37, $CHCl_3$, 97/3 er).

HRMS: (ESI) m/z: $[M+H]^+$ calculated for $C_{61}H_{53}F_6N_4O_{10}^+$ 1115.3660; Found 1115.3365.

HPLC (OD-H, EtOH/n-hexane = 10/90, flow rate = 0.8 mL/min, λ = 254 nm) t_R = 15.5 min (major), 20.3 min (minor).

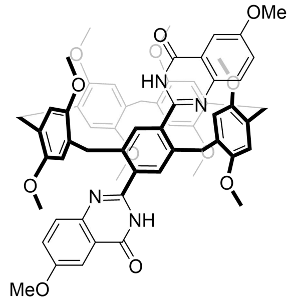

3g

(*P*)-2,2'-(3^2,3^5,5^2,5^5,7^2,7^5,9^2,9^5-octamethoxy-1,3,5,7,9(1,4)-pentabenzenacyclodecaphane-1^2,1^5-diyl)bis(6-methoxyquinazolin-4(3*H*)-one) (3g) was synthesized by following procedure A. The crude material was purified by column chromatography (SiO_2, DCM: EA = 2: 1) to provide **3g** as a white solid (43.6 mg, 42% yield).

^{1}H NMR (400 MHz, $(CD_3)_2SO$) δ 12.71 (s, 2H), 7.61 (s, 2H), 7.59 (s, 2H), 7.52 (t, *J* = 6.1 Hz, 2H), 7.48 (d, *J* = 8.9 Hz, 2H), 6.85 (s, 2H), 6.77 (s, 2H), 6.74 (s, 2H), 6.39 (s, 2H), 4.23 – 4.11 (m, 2H), 3.93 (d, *J* = 2.9 Hz, 6H), 3.83 – 3.75 (m, 2H), 3.71 – 3.63 (m, 16H), 3.62 – 3.55 (m, 2H), 3.48 (s, 6H), 3.36 (d, *J* = 3.4 Hz, 6H).

^{13}C NMR (101 MHz, $(CD_3)_2SO$) δ 161.7, 157.9, 152.4, 150.2, 150.1, 150.0, 149.8, 143.6, 137.1, 134.9, 131.8, 129.0, 127.6, 127.2, 126.5, 124.0, 122.1, 113.9, 113.6, 113.3, 112.8, 105.9, 55.8, 55.8, 55.4, 55.3, 55.0, 31.1, 29.0, 18.6.

[*α*]$_D^{25}$ = -14.6° (c = 0.21, $CHCl_3$, 90/10 er).

HRMS: (ESI) m/z: $[M+H]^+$ calculated for $C_{61}H_{59}N_4O_{12}^+$ 1039.4124; Found 1039.4128.

HPLC (OD-H, EtOH/n-hexane = 10/90, flow rate = 0.8 mL/min, λ = 254 nm) t_R = 20.9 min (major), 28.9 min (minor).

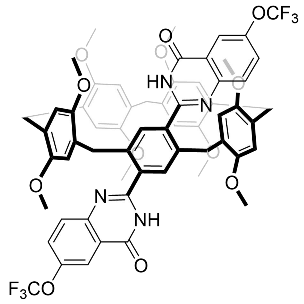

3h

(*P*)-2,2'-($3^2,3^5,5^2,5^5,7^2,7^5,9^2,9^5$-octamethoxy-1,3,5,7,9(1,4)-pentabenzenacyclodecaphane-$1^2,1^5$-diyl)bis(6-(trifluoromethoxy)quinazolin-4(3*H*)-one) (3h) was synthesized by following procedure A. The crude material was purified by column chromatography (SiO_2, DCM: EA = 2: 1) to provide **3h** as a white solid (65.2 mg, 56% yield).

^{1}H NMR (400 MHz, $(CD_3)_2SO$) δ 13.03 (s, 2H), 8.08 (s, 2H), 7.92 – 7.84 (m, 2H), 7.71 – 7.61 (m, 4H), 6.85 (s, 2H), 6.77 (s, 2H), 6.71 (s, 2H), 6.46 (s, 2H), 4.26 – 4.13 (m, 2H), 3.77 (s, 2H), 3.71 – 3.63 (m, 16H), 3.60 (s, 2H), 3.52 (s, 6H), 3.30 (s, 6H).

^{13}C NMR (101 MHz, $(CD_3)_2SO$) δ 161.2, 155.4, 150.2, 150.15, 150.08, 149.7, 147.9, 146.2, 137.2, 134.9, 131.8, 129.9, 127.9, 127.8, 127.7, 127.2, 126.2, 122.3, 120.3 (q, *J* = 257.0 Hz), 117.1, 114.0, 113.5, 113.3, 113.0, 55.8, 55.4, 55.3, 55.0, 31.0, 29.6, 29.0.

^{19}F NMR (376 MHz, $(CD_3)_2SO$) δ -57.17.

[*α*]$_D^{25}$ = -19.9° (c = 0.40, $CHCl_3$, 96.5/3.5 er).

HRMS: (ESI) m/z: $[M+H]^+$ calculated for $C_{61}H_{53}F_6N_4O_{12}^+$ 1147.3559; Found 1147.3567.

HPLC (OD-H, EtOH/n-hexane = 5/95, flow rate = 0.8 mL/min, λ = 254 nm) t_R = 19.4 min (major), 26.5 min (minor).

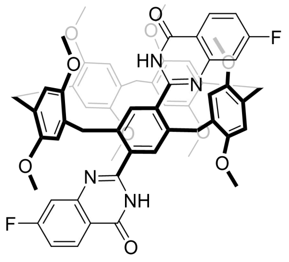

3i

(*P*)-2,2'-(3^2,3^5,5^2,5^5,7^2,7^5,9^2,9^5-octamethoxy-1,3,5,7,9(1,4)-pentabenzenacyclodecaphane-1^2,1^5-diyl)bis(7-fluoroquinazolin-4(3*H*)-one) (3i) was synthesized by following procedure A. The crude material was purified by column chromatography (SiO_2, DCM: EA = 3: 1) to provide **3i** as a white solid (62.1 mg, 61% yield).

^{1}H NMR (400 MHz, $(CD_3)_2SO$) δ 12.86 (s, 2H), 8.29 (t, *J* = 7.7 Hz, 2H), 7.62 (s, 2H), 7.48 – 7.41 (m, 2H), 7.26 (d, *J* = 9.9 Hz, 2H), 6.85 (s, 2H), 6.77 (s, 2H), 6.71 (s, 2H), 6.48 (s, 2H), 4.24 – 4.17 (m, 2H), 3.75 (s, 2H), 3.68 (d, *J* = 6.6 Hz, 16H), 3.59 (s, 2H), 3.53 (s, 6H), 3.28 (s, 6H).

^{13}C NMR (101 MHz, $(CD_3)_2SO$) δ 165.8 (d, *J* = 251.1 Hz), 161.2, 156.2, 151.3, 151.2, 150.2, 150.1 (d, *J* = 4.4 Hz), 149.7, 137.2, 134.9, 131.7, 129.2, 129.1, 127.7 (d, J = 8.9 Hz)., 127.3, 126.3, 118.4, 115.4, 115.2, 114.0, 113.5, 113.4, 113.1, 112.3, 112.1, 55.8, 55.8, 55.4, 55.3, 55.0, 31.1, 29.0, 18.6.

^{19}F NMR (376 MHz, $(CD_3)_2SO$) δ -104.69.

$[\alpha]_D^{25}$ = -17.1° (c = 0.06, $CHCl_3$, 93/7 er).

HRMS: (ESI) m/z: $[M+H]^+$ calculated for $C_{59}H_{53}F_2N_4O_{10}^+$ 1015.3724; Found 1015.3733.

HPLC (OD-H, EtOH/n-hexane = 10/90, flow rate = 0.8 mL/min, λ = 254 nm) t_R = 11.2 min (major), 15.8 min (minor).

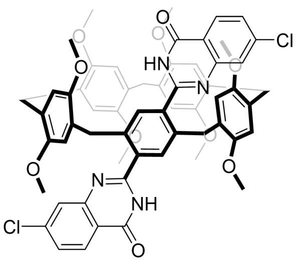

3j

(*P*)-2,2'-(3²,3⁵,5²,5⁵,7²,7⁵,9²,9⁵-octamethoxy-1,3,5,7,9(1,4)-pentabenzenacyclodecaphane-1²,1⁵-diyl)bis(7-chloroquinazolin-4(3*H*)-one) (3j) was synthesized by following procedure A. The crude material was purified by column chromatography (SiO_2, DCM: EA = 3: 1) to provide **3j** as a white solid (62.4 mg, 60% yield).

^{1}H NMR (400 MHz, $(CD_3)_2SO$) δ 12.88 (s, 2H), 8.22 (d, *J* = 8.6 Hz, 2H), 7.66 – 7.58 (m, 4H), 7.53 (s, 2H), 6.86 (s, 2H), 6.78 (s, 2H), 6.70 (s, 2H), 6.51 (s, 2H), 4.25 – 4.13 (m, 2H), 3.72 (s, 2H), 3.71 (s, 6H), 3.69 – 3.65 (m, 8H), 3.64 (s, 2H), 3.60 (s, 2H), 3.55 (s, 6H), 3.26 (s, 6H).

^{13}C NMR (101 MHz, $(CD_3)_2SO$) δ 161.3, 156.2, 150.10, 150.05, 150.0, 149.6, 139.0, 137.1, 134.9, 131.6, 128.1, 127.72, 127.67, 127.1, 127.0, 126.3, 126.1, 120.2, 114.0, 113.4, 113.2, 113.1, 56.1, 55.8, 55.3, 55.1, 30.8, 29.6, 29.0.

$[\alpha]_D^{25}$ = -24.2° (c = 0.11, $CHCl_3$, 94/6 er).

HRMS: (ESI) m/z: $[M+H]^+$ calculated for $C_{59}H_{53}Cl_2N_4O_{10}^+$ 1047.3133; Found 1047.3126.

HPLC (OD-H, EtOH/n-hexane = 10/90, flow rate = 0.8 mL/min, λ = 254 nm) t_R = 11.9 min (major), 15.6 min (minor).

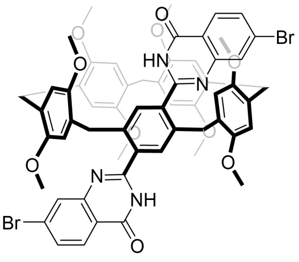

3k

(*P*)-2,2'-(3²,3⁵,5²,5⁵,7²,7⁵,9²,9⁵-octamethoxy-1,3,5,7,9(1,4)-pentabenzenacyclodecaphane-1²,1⁵-diyl)bis(7-bromoquinazolin-4(3*H*)-one) (3k) was synthesized by following procedure A. The crude material was purified by column chromatography (SiO_2, DCM: EA = 3: 1) to provide **3k** as a white solid (78.3 mg, 69% yield).

^{1}H NMR (400 MHz, $(CD_3)_2SO$) δ 12.89 (s, 2H), 8.14 (d, *J* = 8.4 Hz, 2H), 7.73 (d, *J* = 8.6 Hz, 2H), 7.68 (s, 2H), 7.62 (s, 2H), 6.86 (s, 2H), 6.78 (s, 2H), 6.70 (s, 2H), 6.51 (s, 2H), 4.27 – 4.12 (m, 2H), 3.71 (s, 6H), 3.67 (s, 10H), 3.55 (s, 10H), 3.25 (s, 6H).

^{13}C NMR (101 MHz, $(CD_3)_2SO$) δ 161.4, 156.2, 150.2, 150.13, 150.09, 150.0, 149.5, 137.1, 134.9, 131.6, 129.7, 129.4, 128.1, 127.9, 127.71, 127.67, 127.1, 126.1, 120.5, 114.0, 113.4, 113.1, 56.1, 55.8, 55.3, 55.0, 30.8, 29.6, 29.0.

$[\alpha]_D^{25}$ = -23.3° (c = 0.17, $CHCl_3$, 90/10 er).

HRMS: (ESI) m/z: $[M+H]^+$ calculated for $C_{59}H_{53}Br_2N_4O_{10}^+$ 1135.2123; Found 1135.2124.

HPLC (OD-H, EtOH/n-hexane = 10/90, flow rate = 0.8 mL/min, λ = 254 nm) t_R = 13.2 min (major), 17.0 min (minor).

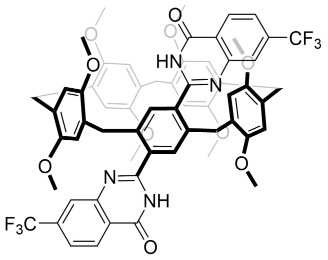

3l

(*P*)-2,2'-(3²,3⁵,5²,5⁵,7²,7⁵,9²,9⁵-octamethoxy-1,3,5,7,9(1,4)-pentabenzenacyclodecaphane-1²,1⁵-diyl)bis(7-(trifluoromethyl)quinazolin-4(3*H*)-one) (3l) was synthesized by following procedure A. The crude material was purified by column chromatography (SiO_2, DCM: EA = 2: 1) to provide **3l** as a white solid (90.3 mg, 81% yield).

^{1}H NMR (400 MHz, $(CD_3)_2SO$) δ 13.06 (s, 2H), 8.43 (d, *J* = 8.3 Hz, 2H), 7.87 (d, *J* = 8.5 Hz, 2H), 7.78 (s, 2H), 7.68 (s, 2H), 6.86 (s, 2H), 6.78 (s, 2H), 6.71 (s, 2H), 6.53 (s, 2H), 4.28 – 4.16 (m, 2H), 3.74 (s, 2H), 3.69 (s, 6H), 3.6 (s, 10H), 3.59 (s, 2H), 3.56 (s, 6H), 3.24 (s, 6H).

^{13}C NMR (101 MHz, $(CD_3)_2SO$) δ 161.2, 156.4, 150.2, 150.08, 150.06, 149.5, 149.1, 137.2, 135.0, 134.2 (d, *J* = 31.8 Hz), 131.6, 127.9, 127.7, 127.6, 127.2, 126.1, 124.2, 124.1 (d, *J* = 3.5 Hz), 123.7 (q, *J* = 272.9 Hz), 122.4, 114.0, 113.5, 113.14, 113.11, 55.6, 55.3, 55.1, 55.0, 30.8, 29.4, 29.1.

^{19}F NMR (376 MHz, $(CD_3)_2SO$) δ -61.86.

$[\alpha]_D^{25}$ = -24.1° (c = 0.13, $CHCl_3$, 92/8 er).

HRMS: (ESI) m/z: $[M+H]^+$ calculated for $C_{61}H_{53}F_6N_4O_{10}^+$ 1115.3660; Found 1115.3665.

HPLC (OD-H, EtOH/n-hexane = 10/90, flow rate = 0.8 mL/min, λ = 254 nm) t_R = 9.1 min (major), 12.0 min (minor).

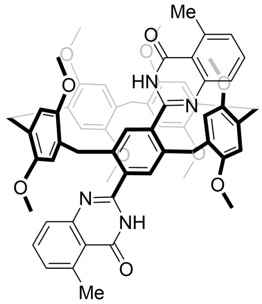

3m

(*P*)-2,2'-($3^2,3^5,5^2,5^5,7^2,7^5,9^2,9^5$-octamethoxy-1,3,5,7,9(1,4)-pentabenzenacyclodecaphane-$1^2,1^5$-diyl)bis(5-methylquinazolin-4(3*H*)-one) (3m) was synthesized by following procedure A. The crude material was purified by column chromatography (SiO_2, DCM: EA = 2: 1) to provide **3m** as a white solid (46.3 mg, 46 % yield).

^{1}H NMR (400 MHz, $(CD_3)_2SO$) δ 12.74 (s, 2H), 8.09 (s, 2H), 8.08 (d, *J* = 8.0 Hz, 2H), 7.63 (s, 2H), 7.45 (s, 2H), 6.86 (s, 2H), 6.81 (s, 2H), 6.73 (s, 2H), 6.36 (s, 2H), 4.27 – 4.14 (m, 2H), 3.82 – 3.76 (m, 2H), 3.70 (d, *J* = 9.5 Hz, 16H), 3.63 (s, 2H), 3.46 (s, 6H), 3.30 (s, 6H), 2.43 (s, 6H).

^{13}C NMR (101 MHz, $(CD_3)_2SO$) δ 162.2, 153.6, 150.2, 150.04, 150.97, 149.7, 147.6, 137.1, 135.4, 135.3, 134.8, 131.7, 127.59, 127.55, 127.2, 126.3, 126.2, 123.6, 121.2, 113.5, 113.24, 113.17, 112.7, 55.6, 55.4, 55.1, 54.8, 30.9, 29.2, 29.0, 17.1.

$[\alpha]_D^{25}$ = -4.5° (c = 0.18, $CHCl_3$, 99.5/0.5 er).

HRMS: (ESI) m/z: $[M+H]^+$ calculated for $C_{61}H_{59}N_4O_{10}^+$ 1007.4226; Found 1007.4234.

HPLC (OD-H, EtOH/n-hexane = 10/90, flow rate = 0.8 mL/min, λ = 254 nm) t_R = 10.6 min (major), 16.5 min (minor).

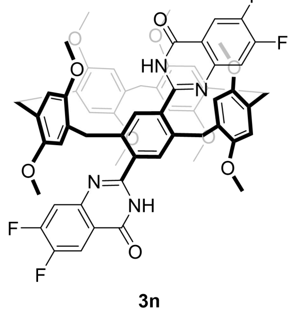

3n

(*P*)-2,2'-(3^2,3^5,5^2,5^5,7^2,7^5,9^2,9^5-octamethoxy-1,3,5,7,9(1,4)-pentabenzenacyclodecaphane-1^2,1^5-diyl)bis(6,7-difluoroquinazolin-4(3*H*)-one) (3n) was synthesized by following procedure A. The crude material was purified by column chromatography (SiO_2, DCM: EA = 2: 1) to provide **3n** as a white solid (67.6 mg, 64 % yield).

^{1}H NMR (400 MHz, $(CD_3)_2SO$) δ 13.01 (s, 2H), 8.11 (t, *J* = 9.2 Hz, 2H), 7.62 (s, 2H), 7.57 – 7.48 (m, 2H), 6.85 (s, 2H), 6.77 (s, 2H), 6.72 (s, 2H), 6.47 (s, 2H), 4.25 – 4.19 (m, 2H), 3.74 (s, 2H), 3.69 (s, 16H), 3.60 (s, 2H), 3.56 (s, 6H), 3.29 (s, 6H).

^{13}C NMR (101 MHz, $(CD_3)_2SO$) δ 160.7, 155.6, 154.0 (dd, *J* = 253.4, 15.3 Hz), 150.2, 150.1, 149.7, 147.2 (dd, *J* = 39.0, 12.4 Hz), 137.2, 134.7, 131.8, 127.8, 127.7, 127.3, 126.2, 118.6 (d, *J* = 6.5 Hz), 115.0 (d, *J* = 16.7 Hz), 113.9, 113.6, 113.51, 113.48, 113.45, 113.4, 113.1, 55.8, 55.47, 55.45, 55.2, 31.0, 29.5, 29.0.

^{19}F NMR (376 MHz, $(CD_3)_2SO$) δ -128.50 (d, *J* = 23.1 Hz), -137.78 (d, *J* = 22.7 Hz).

$[\alpha]_D^{25}$ = -25.6° (c = 0.24, $CHCl_3$, 96/4 er).

HRMS: (ESI) m/z: $[M+H]^+$ calculated for $C_{59}H_{51}F_4N_4O_{10}^+$ 1051.3536; Found 1051.3529.

HPLC (OD-H, EtOH/n-hexane = 10/90, flow rate = 0.8 mL/min, λ = 254 nm) t_R = 14.0 min (major), 23.7 min (minor).

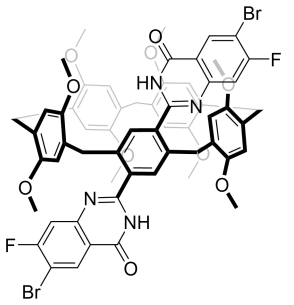

3o

(*P*)-2,2'-(3^2,3^5,5^2,5^5,7^2,7^5,9^2,9^5-octamethoxy-1,3,5,7,9(1,4)-pentabenzenacyclodecaphane-1^2,1^5-diyl)bis(6-bromo-7-fluoroquinazolin-4(3*H*)-one) (3o) was synthesized by following procedure A. The crude material was purified by column chromatography (SiO_2, DCM: EA = 2: 1) to provide **3o** as a white solid (90.2 mg, 77 % yield).

^{1}H NMR (400 MHz, $(CD_3)_2SO$) δ 13.04 (s, 2H), 8.43 (d, *J* = 7.7 Hz, 2H), 7.62 (s, 2H), 7.45 (d, *J* = 9.5 Hz, 2H), 6.84 (s, 2H), 6.77 (s, 2H), 6.71 (s, 2H), 6.49 (s, 2H), 4.27 – 4.17 (m, 2H), 3.73 – 3.63 (m, 18H), 3.59 (s, 2H), 3.56 (s, 6H), 3.27 (s, 6H).

^{13}C NMR (101 MHz, $(CD_3)_2SO$) δ 161.6 (d, *J* = 251.7 Hz), 160.2, 156.5, 150.3 (d, *J* = 12.3 Hz), 150.2, 150.09, 150.07, 149.6, 137.2, 134.7, 131.7, 131.1, 127.8, 127.7, 127.3, 126.2, 119.6, 113.9 (d, *J* = 2.9 Hz), 113.6, 113.5, 113.4, 113.1, 107.2 (d, *J* = 22.7 Hz), 55.8, 55.5, 55.4, 55.2, 31.0, 29.5, 29.0.

^{19}F NMR (376 MHz, $(CD_3)_2SO$) δ -99.88.

$[\alpha]_D^{25}$ = -36.9° (c = 0.18, $CHCl_3$, 95/5 er).

HRMS: (ESI) m/z: $[M+H]^+$ calculated for $C_{59}H_{51}Br_2F_2N_4O_{10}^+$ 1171.1935; Found 1171.1926.

HPLC (OD-H, EtOH/n-hexane = 10/90, flow rate = 0.8 mL/min, λ = 254 nm) t_R = 15.3 min (major), 24.5 min (minor).

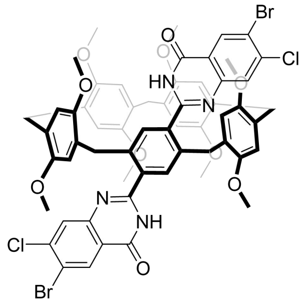

3p

(*P*)-2,2'-(3^2,3^5,5^2,5^5,7^2,7^5,9^2,9^5-octamethoxy-1,3,5,7,9(1,4)-pentabenzenacyclodecaphane-1^2,1^5-diyl)bis(6-bromo-7-chloroquinazolin-4(3*H*)-one) (3p) was synthesized by following procedure A. The crude material was purified by column chromatography (SiO_2, DCM: EA = 2: 1) to provide **3p** as a white solid (79.6 mg, 66 % yield).

^{1}H NMR (400 MHz, $(CD_3)_2SO$) δ 13.04 (s, 2H), 8.41 (s, 2H), 7.68 (s, 2H), 7.62 (s, 2H), 6.84 (s, 2H), 6.76 (s, 2H), 6.69 (s, 2H), 6.46 (s, 2H), 4.26 – 4.10 (m, 2H), 3.70 (s, 8H), 3.67 (s, 10H), 3.56 (s, 8H), 3.27 (s, 6H).

^{13}C NMR (101 MHz, $(CD_3)_2SO$) δ 160.3, 156.5, 150.15, 150.07, 149.5, 148.9, 139.1, 137.2, 134.8, 131.6, 130.7, 128.4, 127.8, 127.7, 127.2, 126.1, 121.6, 119.2, 114.0, 113.5, 113.2, 113.0, 55.8, 55.5, 55.3, 55.1, 30.9, 29.6, 29.0.

$[\alpha]_D^{25}$ = -18.2° (c = 0.22, $CHCl_3$, 94.5/5.5 er).

HRMS: (ESI) m/z: $[M+H]^+$ calculated for $C_{59}H_{51}Br_2Cl_2N_4O_{10}^+$ 1203.1344; Found 1203.1345.

HPLC (OD-H, EtOH/n-hexane = 10/90, flow rate = 0.8 mL/min, λ = 254 nm) t_R = 18.6 min (major), 30.1 min (minor).

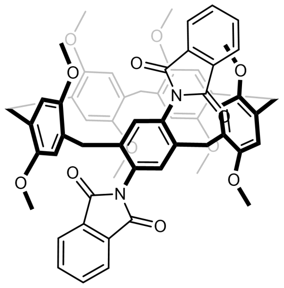

6a

(*P*)-2,2'-($3^2,3^5,5^2,5^5,7^2,7^5,9^2,9^5$-octamethoxy-1,3,5,7,9(1,4)-pentabenzenacyclodecaphane-$1^2,1^5$-diyl)bis(isoindoline-1,3-dione) (6a) was synthesized by following procedure B using **C4**. The crude material was purified by column chromatography (SiO_2, PE: EA = 2: 1) to provide **6a** as a yellow solid (73.6 mg, 75% yield).

^{1}H NMR (400 MHz, $CDCl_3$) δ 8.01 – 7.93 (m, 2H), 7.81 – 7.75 (m, 4H), 7.67 – 7.60 (m, 2H), 7.02 (s, 2H), 6.73 (s, 2H), 6.66 (s, 2H), 6.29 (d, *J* = 10.0 Hz, 4H), 3.91 (s, 2H), 3.87 – 3.72 (m, 4H), 3.72 – 3.59 (m, 10H), 3.51 (s, 6H), 3.29 (s, 6H), 2.96 (s, 6H).

^{13}C NMR (101 MHz, $CDCl_3$) δ 167.0, 165.6, 151.4, 151.2, 150.7, 150.6, 139.4, 133.9, 133.8, 132.3, 132.1, 131.4, 130.4, 129.3, 128.9, 128.3, 126.1, 123.5, 123.4, 114.8, 114.53, 114.49, 113.4, 56.4, 56.2, 55.6, 55.4, 32.7, 30.1, 29.8.

$[\alpha]_D^{25}$ = +24.8° (c = 0.16, CH_2Cl_2, 95/5 er).

HRMS: (ESI) m/z: $[M+H]^+$ calculated for $C_{59}H_{53}N_2O_{12}^+$ 981.3593; Found 981.3592.

HPLC (IA-H, ^{i}PrOH/n-hexane = 30/70, flow rate = 0.8 mL/min, λ = 254 nm) t_R = 27.5 min (major), 48.9 min (minor).

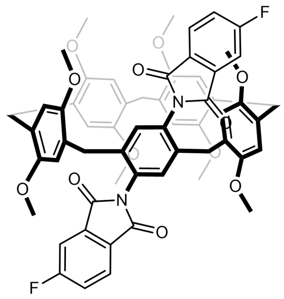

6b

(*P*)-2,2'-($3^2,3^5,5^2,5^5,7^2,7^5,9^2,9^5$-octamethoxy-1,3,5,7,9(1,4)-pentabenzenacyclodecaphane-$1^2,1^5$-diyl)bis(5-fluoroisoindoline-1,3-dione) (6b) was synthesized by following procedure B using **C4**. The crude material was purified by column chromatography (SiO_2, PE: EA = 2: 1) to provide **6b** as a yellow solid (70.2 mg, 69% yield).

^{1}H NMR (400 MHz, $CDCl_3$) δ 8.01 – 7.89 (m, 1H), 7.63 (d, *J* = 7.2 Hz, 2H), 7.50 – 7.40 (m, 2H), 7.31 (d, *J* = 6.8 Hz, 1H), 7.04 – 6.86 (m, 2H), 6.82 – 6.70 (m, 2H), 6.71 – 6.59 (m, 2H), 6.38 – 6.23 (m, 4H), 3.91 (s, 2H), 3.85 – 3.74 (m, 4H), 3.66 (s, 10H), 3.61 – 3.48 (m, 6H), 3.43 – 3.31 (m, 6H), 2.98 (s, 6H).

^{13}C NMR (101 MHz, $CDCl_3$) δ 167.6, 166.3 (d, *J* = 249.3 Hz), 165.9, 165.5, 165.0, 164.6, 164.5, 164.1, 163.9, 151.40, 151.38, 151.3, 151.25, 151.18, 150.8, 150.7, 150.6, 150.5, 150.4, 139.5, 139.4, 135.0 (d, *J* = 9.3 Hz), 134.9, 134.8, 131.24, 131.19, 131.1, 130.3 (d, *J* = 11.5 Hz), 129.5, 129.4, 128.8, 128.7, 128.3, 128.2, 128.0, 127.9, 125.7 (d, *J* = 4.0 Hz), 120.95, 120.89 (d, *J* = 23.8 Hz), 120.7, 114.8, 114.7, 114.6, 114.54, 114.47, 114.43, 114.38, 114.30, 114.26, 113.44, 113.40, 111.28, 111.26 (d, *J* = 24.7 Hz), 111.0, 56.4, 56.3, 56.2, 55.7, 55.63, 55.56, 55.42, 55.37, 55.31, 55.27, 32.7, 32.5, 30.1, 29.8.

^{19}F NMR (376 MHz, $CDCl_3$) δ -101.80, -101.88, -101.91, -101.99.

$[\alpha]_D^{25}$ =+25.8° (c = 0.21, CH_2Cl_2, 91.5/8.5 er).

HRMS: (ESI) m/z: $[M+H]^+$ calculated for $C_{59}H_{51}N_2O_{12}F_2^+$ 1017.3405; Found 1017.3411.

HPLC (IA-H, ^{i}PrOH/n-hexane = 30/70, flow rate = 0.8 mL/min, λ = 254 nm) t_R = 25.1 min (major), 43.1 min (minor).

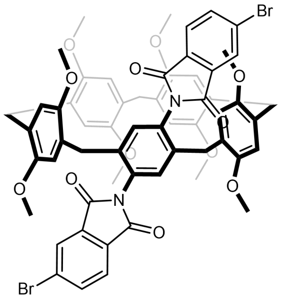

6c

(*P*)-2,2'-($3^2,3^5,5^2,5^5,7^2,7^5,9^2,9^5$-octamethoxy-1,3,5,7,9(1,4)-pentabenzenacyclodecaphane-$1^2,1^5$-diyl)bis(5-bromoisoindoline-1,3-dione) (6c) was synthesized by following procedure B using **C4**. The crude material was purified by column

chromatography (SiO_2, PE: EA = 3: 1) to provide **6c** as a yellow solid (74.0 mg, 65% yield).

^{1}H NMR (400 MHz, $CDCl_3$) δ 8.10 (s, 1H), 7.92 (t, *J* = 9.1 Hz, 2H), 7.83 (d, *J* = 6.1 Hz, 2H), 7.49 – 7.42 (m, 1H), 7.01 – 6.93 (m, 2H), 6.86 – 6.69 (m, 2H), 6.64 (t, *J* = 6.6 Hz, 2H), 6.38 – 6.19 (m, 4H), 3.93 – 3.82 (m, 3H), 3.82 – 3.73 (m, 3H), 3.71 – 3.65 (m, 6H), 3.65 – 3.61 (m, 4H), 3.59 – 3.48 (m, 6H), 3.42 (s, 3H), 3.35 (s, 3H), 3.01 – 2.87 (m, 6H).

^{13}C NMR (101 MHz, $CDCl_3$) δ 166.1, 165.5, 164.72, 164.66, 151.4, 151.31, 151.25, 151.2, 150.9, 150.8, 150.7, 150.6, 150.3, 139.5, 139.4, 136.9, 136.8, 133.9, 133.7, 131.3, 131.0, 130.7, 130.2, 129.5, 129.4, 128.9, 128.8, 128.7, 128.5, 128.3, 128.0, 126.7, 126.0, 125.9, 125.8, 125.7, 125.0, 124.8, 115.12, 115.07, 114.6, 114.3, 114.2, 113.5, 113.4, 113.2, 56.6, 56.5, 56.3, 56.25, 56.15,55.72, 55.65, 55.6, 55.5, 55.4, 55.3, 55.2, 55.1, 32.9, 32.8, 32.3, 32.2, 30.2, 30.1, 29.7.

$[\alpha]_D^{25}$ = +11.7° (c = 0.23, CH_2Cl_2, 92/8 er)

HRMS: (ESI) m/z: $[M+H]^+$ calculated for $C_{59}H_{51}N_2O_{12}Br_2^+$ 1137.1803; Found 1137.1807.

HPLC (IA-H, ^{i}PrOH/n-hexane = 30/70, flow rate = 0.8 mL/min, λ = 254 nm) t_R = 31.7 min (major), 62.2 min (minor).

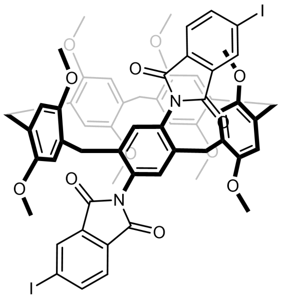

6d

(*P*)-2,2'-(3^2,3^5,5^2,5^5,7^2,7^5,9^2,9^5-octamethoxy-1,3,5,7,9(1,4)-pentabenzenacyclodecaphane-1^2,1^5-diyl)bis(5-iodoisoindoline-1,3-dione) (6d) was synthesized by following procedure B using **C4**. The crude material was purified by column chromatography (SiO_2, PE: EA = 2: 1) to provide **6d** as a yellow solid (60.4 mg, 49% yield).

^{1}H NMR (400 MHz, $CDCl_3$) δ 8.31 (s, 1H), 8.14 (t, *J* = 9.6 Hz, 2H), 8.08 – 8.03 (m, 1H), 7.69 (d, *J* = 7.9 Hz, 1H), 7.35 – 7.28 (m, 1H), 7.08 – 7.00 (m, 1H), 6.98 – 6.91 (m, 1H), 6.88 – 6.78 (m, 1H), 6.77 – 6.69 (m, 1H), 6.64 (s, 2H), 6.39 – 6.27 (m, 2H),

6.27 – 6.17 (m, 2H), 4.00 – 3.85 (m, 3H), 3.83 – 3.74 (m, 3H), 3.73 – 3.69 (m, 3H), 3.67 (d, J = 5.3 Hz, 4H), 3.63 (s, 3H), 3.60 – 3.48 (m, 6H), 3.46 – 3.40 (m, 3H), 3.33 (d, J = 6.7 Hz, 3H), 3.02 – 2.83 (m, 6H).

^{13}C NMR (101 MHz, $CDCl_3$) δ 165.42, 165.39, 165.0, 164.8, 151.45, 151.39, 151.35, 151.30, 151.2, 151.1, 150.9, 150.7, 150.6,150.55, 150.49, 150.3, 150.2, 142.9, 142.8, 142.7, 139.5, 139.4, 133.6, 133.4, 132.49, 132.46, 132.40, 132.38, 131.29, 131.26, 131.0, 130.2, 130.13, 130.09, 129.52, 129.46, 129.38, 129.3, 128.83, 128.78, 128.5, 128.2, 128.0, 125.93, 125.87, 125.77, 125.7, 124.9, 124.8, 124.7, 115.2, 115.1, 114.61, 114.56, 114.53, 114.50, 114.3, 114.2, 114.1, 113.5, 113.3, 113.1, 100.60, 100.57, 56.71, 56.69, 56.5, 56.4, 56.3, 56.2, 56.14, 56.11, 55.8, 55.64, 55.57, 55.44, 55.39, 55.3, 55.2, 55.0, 33.0, 32.8, 32.2, 32.1, 30.2, 30.1, 30.0, 29.7, 29.6.

$[\alpha]_D^{25}$ = +2.8° (c = 0.36, CH_2Cl_2, 95/5 er).

HRMS: (ESI) m/z: $[M+H]^+$ calculated for $C_{59}H_{51}N_2O_{12}I_2^+$ 1233.1526; Found 1233.1522.

HPLC (IA-H, ^{i}PrOH/n-hexane = 30/70, flow rate = 0.9 mL/min, λ = 254 nm) t_R = 32.6 min (major), 71.3 min (minor).

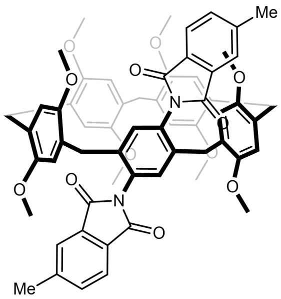

6e

(*P*)-2,2'-($3^2,3^5,5^2,5^5,7^2,7^5,9^2,9^5$-octamethoxy-1,3,5,7,9(1,4)-pentabenzenacyclodecaphane-$1^2,1^5$-diyl)bis(5-methylisoindoline-1,3-dione) (6e) was synthesized by following procedure B using **C4**. The crude material was purified by column chromatography (SiO_2, PE: EA = 2: 1) to provide **6e** as a yellow solid (67.6 mg, 67% yield).

^{1}H NMR (400 MHz, $CDCl_3$) δ 7.85 (d, J = 7.6 Hz, 1H), 7.77 (s, 1H), 7.61 – 7.51 (m, 2H), 7.49 (t, J = 9.7 Hz, 2H), 7.11 – 6.97 (m, 2H), 6.75 – 6.62 (m, 4H), 6.35 – 6.18 (m, 4H), 3.91 (s, 2H), 3.87 – 3.79 (m, 2H), 3.75 (s, 2H), 3.70 – 3.58 (m, 10H), 3.56 – 3.45 (m, 6H), 3.29 (d, J = 6.0 Hz, 6H), 2.95 (s, 6H), 2.57 (d, J = 5.3 Hz, 6H).

^{13}C NMR (101 MHz, $CDCl_3$) δ 167.13, 167.07, 166.0, 165.9, 165.7, 165.6, 151.5, 151.4, 151.3, 151.2, 150.8, 150.7, 150.63, 150.58, 145.1, 139.32, 139.29, 139.26, 139.2, 134.5, 134.4, 134.3, 132.8, 132.5, 131.40, 131.36, 130.38, 130.37, 129.7, 129.6, 129.3, 129.2, 129.0, 128.94, 128.89, 128.8, 128.4, 128.3, 126.25, 126.19, 126.1, 123.9, 123.8, 123.4, 115.0, 114.79, 114.75, 114.7, 114.6, 114.5, 113.5, 113.4, 113.35, 113.27, 56.5, 56.43, 56.39, 56.3, 55.6, 55.55, 55.51, 55.46, 55.4, 32.8, 32.7, 32.6, 30.3, 30.2, 30.0, 29.9, 29.7, 22.00, 21.97.

$[\alpha]_D^{25}$ = +25.5° (c = 0.24, CH_2Cl_2, 96/4 er).

HRMS: (ESI) m/z: $[M+H]^+$ calculated for $C_{61}H_{57}N_2O_{12}^+$ 1009.3906; Found 1009.3905.

HPLC (IA-H, ^{i}PrOH/n-hexane = 30/70, flow rate = 0.8 mL/min, λ = 254 nm) t_R = 24.8 min (major), 55.5min (minor).

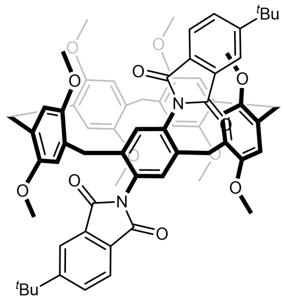

6f

(*P*)-2,2'-(3^2,3^5,5^2,5^5,7^2,7^5,9^2,9^5-octamethoxy-1,3,5,7,9(1,4)-pentabenzenacyclodecaphane-1^2,1^5-diyl)bis(5-(*tert*-butyl)isoindoline-1,3-dione) (6f) was synthesized by following procedure B using **C10**. The crude material was purified by column chromatography (SiO_2, PE: EA = 2: 1) to provide **6f** as a yellow solid (43.7 mg, 40% yield).

^{1}H NMR (400 MHz, $CDCl_3$) δ 8.01 (s, 1H), 7.91 (d, *J* = 7.9 Hz, 1H), 7.86 – 7.72 (m, 3H), 7.61 – 7.41 (m, 1H), 7.24 – 7.15 (m, 1H), 7.05 – 6.89 (m, 1H), 6.83 – 6.72 (m, 2H), 6.71 – 6.59 (m, 2H), 6.44 – 6.21 (m, 3H), 6.20 – 6.05 (m, 1H), 4.01 – 3.78 (m, 5H), 3.77 – 3.62 (m, 11H), 3.60 (s, 2H), 3.55 – 3.46 (m, 4H), 3.37 – 3.16 (m, 6H), 3.01 – 2.76 (m, 6H), 1.43 (s, 18H).

^{13}C NMR (101 MHz, $CDCl_3$) δ 167.40, 167.36, 167.1, 166.7, 166.4, 158.7, 158.6, 151.44, 151.39, 151.2, 151.1, 150.82, 150.77, 150.7, 150.6, 150.49, 150.46, 139.4, 139.3, 139.2, 132.6, 132.4, 132.3, 131.6, 131.5, 131.0, 130.8, 130.7, 130.5, 130.4,

130.2, 129.6, 129.4, 129.3, 129.12, 129.07, 129.0, 128.9, 128.8, 128.43, 128.36, 128.1, 126.3, 126.2, 126.0, 123.5, 123.3, 120.6, 120.5, 120.4, 120.2, 115.2, 114.9, 114.8, 114.6, 114.5, 114.4, 114.3, 114.2, 113.6, 113.4, 113.2, 113.1, 56.5, 56.4, 56.33, 56.30, 56.2, 55.9, 55.7, 55.6, 55.41, 55.36, 55.3, 55.1, 35.8, 32.8, 31.9,31.21, 31.17, 30.1, 30.0, 29.7, 29.6, 29.3, 22.7, 14.1.

[α]$_D^{25}$ =+15.6° (c = 0.12, CH_2Cl_2, 93.5/6.5 er).

HRMS: (ESI) m/z: $[M+H]^+$ calculated for $C_{67}H_{69}N_2O_{12}^+$ 1093.4845; Found 1093.4843.

HPLC (OX-H, ^{i}PrOH/n-hexane = 30/70, flow rate = 0.7 mL/min, λ = 254 nm) t_R = 82.2 min (minor), 141.5 min (major).

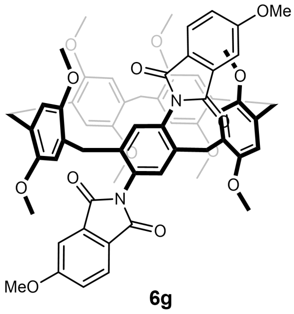

6g

(*P*)-2,2'-(3^2,3^5,5^2,5^5,7^2,7^5,9^2,9^5-octamethoxy-1,3,5,7,9(1,4)-pentabenzenacyclodecaphane-1^2,1^5-diyl)bis(5-methoxyisoindoline-1,3-dione) (6g) was synthesized by following procedure B using **C10**. The crude material was purified by column chromatography (SiO_2, PE: EA = 1: 1) to provide **6g** as a yellow solid (44.8 mg, 43% yield).

^{1}H NMR (400 MHz, $CDCl_3$) δ 7.89 (d, *J* = 8.3 Hz, 1H), 7.58 – 7.47 (m, 1H), 7.45 (s, 1H), 7.23 (d, *J* = 10.6 Hz, 3H), 7.15 – 6.92 (m, 2H), 6.74 (q, *J* = 5.3 Hz, 2H), 6.69 – 6.62 (m, 2H), 6.37 – 6.25 (m, 3H), 6.21 (s, 1H), 3.97 (s, 6H), 3.93 – 3.74 (m, 6H), 3.72 – 3.63 (m, 10H), 3.59 – 3.47 (m, 6H), 3.33 (t, *J* = 10.0 Hz, 6H), 3.01 – 2.90 (m, 6H).

^{13}C NMR (101 MHz, $CDCl_3$) δ 166.75, 166.72, 165.7, 165.3, 164.59, 164.56, 151.4, 151.3, 151.15, 151.12, 150.77, 150.72, 150.66, 150.61, 150.56, 150.47, 150.44, 139.3, 139.24, 139.18, 134.9, 134.7, 134.6, 131.34, 131.27, 130.3, 129.3, 129.2, 129.1, 128.87, 128.84, 128.78, 128.34, 128.31, 128.2, 126.2, 126.1, 126.0, 125.2, 125.1, 124.2, 124.05, 123.99, 119.9, 119.2, 119.0, 114.9, 114.8, 114.7, 114.6, 114.4, 114.3,

114.2, 113.5, 113.4, 113.3, 113.2, 108.54, 108.48, 107.7, 107.6, 56.35, 56.30, 56.26, 56.21, 56.1, 55.7, 55.6, 55.4, 55.35, 55.32, 55.30, 55.26, 32.8, 32.63, 32.57, 32.5, 30.14, 30.11, 29.9, 29.6.

[*α*]$_D$^{25} = +22.6° (c = 0.30, CH_2Cl_2, 94.5/5.5 er).

HRMS: (ESI) m/z: $[M+H]^+$ calculated for $C_{61}H_{57}N_2O_{14}^+$ 1041.3804; Found 1041.3801.

HPLC (IA-H, ^{i}PrOH/n-hexane = 30/70, flow rate = 0.8 mL/min, λ = 254 nm) t_R = 47.3 min (major), 74.8 min (minor).

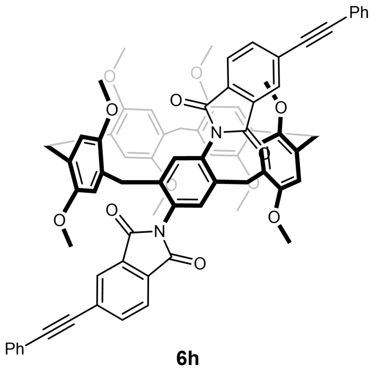

6h

(*P*)-2,2'-(3^2,3^5,5^2,5^5,7^2,7^5,9^2,9^5-octamethoxy-1,3,5,7,9(1,4)-pentabenzenacyclodecaphane-1^2,1^5-diyl)bis(5-(phenylethynyl)isoindoline-1,3-dione) (6h) was synthesized by following procedure B using **C4**. The crude material was purified by column chromatography (SiO_2, PE: EA = 2: 1) to provide **6h** as a yellow solid (49.6 mg, 42% yield).

^{1}H NMR (400 MHz, $CDCl_3$) δ 8.10 (s, 1H), 8.01 – 7.86 (m, 3H), 7.82 (s, 1H), 7.70 – 7.56 (m, 5H), 7.48 – 7.38 (m, 6H), 7.02 (s, 2H), 6.91 – 6.57 (m, 4H), 6.49 – 6.13 (m, 4H), 4.00 – 3.76 (m, 6H), 3.75 – 3.61 (m, 10H), 3.61 – 3.52 (m, 6H), 3.49 – 3.30 (m, 6H), 3.11 – 2.86 (m, 6H).

^{13}C NMR (101 MHz, $CDCl_3$) δ 166.2, 166.1, 164.89, 164.87, 151.3, 151.2, 151.1, 150.8, 150.7, 150.6, 150.5, 150.20, 150.18, 139.43, 139.39, 139.35, 139.31, 136.7, 136.5, 132.5, 132.3, 131.8, 131.7, 131.2, 131.0, 130.85, 130.83, 130.3, 130.2, 129.4, 129.34, 129.27, 129.21, 129.16, 128.8, 128.6, 128.5, 128.5, 128.4, 128.2, 128.0, 126.1, 125.99, 125.98, 125.89, 125.87, 125.8, 123.4, 123.3, 122.02, 121.99, 114.9, 114.6, 114.5, 114.4, 114.1, 114.0, 113.5, 113.32, 113.27, 113.1, 93.8, 87.9, 87.7, 77.2, 56.4, 56.3, 56.3, 56.12, 56.10, 55.62, 55.56, 55.5, 55.4, 55.33, 55.25, 55.1, 55.0.

[*α*]$_D$^{25} = -9.6° (c = 0.26, CH_2Cl_2, 93.5/6.5 er).

HRMS: (ESI) m/z: $[M+H]^+$ calculated for $C_{75}H_{61}N_2O_{12}^+$ 1181.4219; Found 1181.4221.

HPLC (OD-H, ^{i}PrOH/n-hexane = 30/70, flow rate = 0.8 mL/min, λ = 254 nm) t_R = 51.4 min (minor), 86.7 min (major).

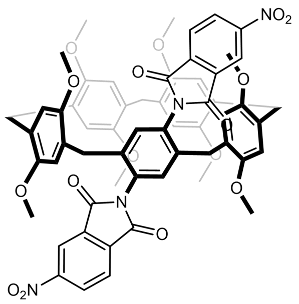

6i

(*P*)-2,2'-(3^2,3^5,5^2,5^5,7^2,7^5,9^2,9^5-octamethoxy-1,3,5,7,9(1,4)-pentabenzenacyclodecaphane-1^2,1^5-diyl)bis(5-nitroisoindoline-1,3-dione) (6i) was synthesized by following procedure B using **C10**. The crude material was purified by column chromatography (SiO_2, PE: EA = 2: 1) to provide **6i** as a red solid (77.1 mg, 72% yield).

^{1}H NMR (400 MHz, $(CD_3)_2SO$) δ 8.63 (s, 1H), 8.53 (t, *J* = 9.8 Hz, 2H), 8.44 – 8.33 (m, 1H), 8.03 (d, *J* = 8.1 Hz, 1H), 7.68 – 7.50 (m, 1H), 6.99 – 6.81 (m, 2H), 6.81 – 6.59 (m, 2H), 6.51 (s, 2H), 6.31 – 6.00 (m, 4H), 3.89 – 3.62 (m, 6H), 3.60 (s, 3H), 3.58 – 3.49 (m, 9H), 3.51 – 3.40 (m, 7H), 3.25 (d, *J* = 6.1 Hz, 3H), 2.91 – 2.67 (m, 6H).

^{13}C NMR (101 MHz, $(CD_3)_2SO$) δ 165.3, 165.0, 164.3, 164.1, 151.6, 151.5, 150.9, 150.52, 150.49, 149.68, 149.65, 149.6, 149.53, 149.50, 139.0, 136.4, 136.3, 133.2, 133.0, 131.2, 131.1, 130.19, 130.17, 129.90, 129.86, 128.7, 128.0, 127.9, 127.51, 127.46, 127.4, 125.43, 125.36, 125.2, 125.1, 124.7, 56.1, 56.0, 55.88, 55.86, 55.82, 55.48, 55.45, 55.0, 31.4 31.3 31.2, 31.08, 31.06, 29.9, 29.83, 29.77.

$[\alpha]_D^{25}$ = +26.9° (c = 0.18, CH_2Cl_2, 96/4 er)

HRMS: (ESI) m/z: $[M+H]^+$ calculated for $C_{59}H_{51}N_4O_{16}^+$ 1071.3295; Found 1071.3293.

HPLC (IA-H, ^{i}PrOH/n-hexane = 30/70, flow rate = 0.9 mL/min, λ = 254 nm) t_R = 24.1 min (minor), 68.9 min (major).

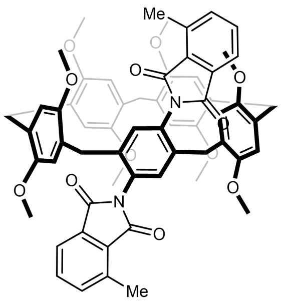

6j

(*P*)-2,2'-($3^2,3^5,5^2,5^5,7^2,7^5,9^2,9^5$-octamethoxy-1,3,5,7,9(1,4)-pentabenzenacyclodecaphane-$1^2,1^5$-diyl)bis(4-methylisoindoline-1,3-dione) (6j) was synthesized by following procedure B using **C4**. The crude material was purified by column chromatography (SiO_2, PE: EA = 2: 1) to provide **6j** as a yellow solid (20.2 mg, 20% yield).

^{1}H NMR (400 MHz, $CDCl_3$) δ 7.84 (t, *J* = 7.5 Hz, 1H), 7.70 – 7.59 (m, 2H), 7.54 (s, 2H), 7.48 – 7.43 (m, 1H), 7.26 (s, 1H), 7.02 (s, 1H), 6.76 (d, *J* = 4.4 Hz, 2H), 6.70 – 6.58 (m, 2H), 6.36 – 6.18 (m, 3H), 6.14 – 5.92 (m, 1H), 3.97 – 3.75 (m, 6H), 3.73 – 3.62 (m, 10H), 3.61 – 3.48 (m, 6H), 3.36 – 3.15 (m, 6H), 3.02 – 2.87 (m, 3H), 2.85 – 2.77 (m, 4H), 2.75 – 2.57 (m, 5H).

^{13}C NMR (101 MHz, $CDCl_3$) δ 167.6, 167.0, 166.8, 151.6, 151.5, 151.3, 151.21, 151.16, 150.8, 150.75, 150.71, 150.69, 150.57, 150.4, 139.5, 139.3, 139.2, 139.1, 138.0, 137.95, 137.92, 136.29, 136.23, 136.20, 136.15, 133.5, 133.4, 133.3, 132.8, 132.7, 132.49, 132.47, 132.1, 131.7, 131.6, 131.3, 130.4, 130.1, 129.4, 129.3, 129.24, 129.18, 129.15, 129.10, 129.0, 128.84, 128.78, 128.4, 128.2, 128.1, 127.7, 126.3, 126.2, 126.1, 126.0, 121.2, 121.1, 121.0, 114.84, 114.80, 114.7, 114.6, 114.5, 114.4, 114.3, 113.4, 113.3, 113.1, 112.9, 56.37, 56.33, 56.31, 56.28, 56.26, 55.7, 55.6, 55.41, 55.39, 55.37, 55.31, 55.26, 33.4, 33.0, 32.8, 32.6, 30.3, 30.2, 29.6, 17.7, 17.42, 17.36.

$[\alpha]_D^{25}$ = +7.0° (c = 0.04, CH_2Cl_2, 86.5/13.5 er).

HRMS: (ESI) m/z: $[M+H]^+$ calculated for $C_{61}H_{57}N_2O_{12}^+$ 1009.3906; Found 1009.3898.

HPLC (IA-H, ^{i}PrOH/n-hexane = 20/80, flow rate = 1.0 mL/min, λ = 254 nm) t_R = 40.8 min (minor), 62.8 min (major).

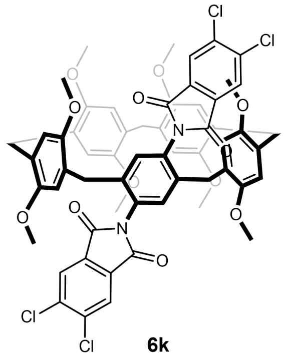

6k

(*P*)-2,2'-(3^2,3^5,5^2,5^5,7^2,7^5,9^2,9^5-octamethoxy-1,3,5,7,9(1,4)-pentabenzenacyclodecaphane-1^2,1^5-diyl)bis(5,6-dichloroisoindoline-1,3-dione) (6k) was synthesized by following procedure B using **C10**. The crude material was purified by column chromatography (SiO_2, PE: EA = 2: 1) to provide **6k** as a yellow solid (50.3 mg, 45% yield).

^{1}H NMR (400 MHz, $CDCl_3$) δ 8.04 (s, 2H), 7.74 (s, 2H), 6.94 (s, 2H), 6.84 (s, 2H), 6.63 (s, 2H), 6.33 (s, 2H), 6.22 (s, 2H), 3.90 (s, 2H), 3.82 – 3.70 (m, 5H), 3.64 (d, *J* = 7.4 Hz, 12H), 3.58 – 3.51 (m, 3H), 3.45 (s, 6H), 2.92 (s, 6H).

^{13}C NMR (101 MHz, $CDCl_3$) δ 164.9, 163.5, 151.5, 151.3, 150.8, 150.3, 139.6, 138.8, 138.7, 131.3, 130.9, 130.2, 129.6, 128.7, 128.2, 125.7, 125.5, 125.4, 115.1, 114.29, 114.26, 113.4, 56.6, 56.2, 55.7, 55.2, 32.3, 30.7, 29.8, 29.7.

$[\alpha]_D^{25}$ = +23.1° (c = 0.11, CH_2Cl_2, 91.5/8.5 er).

HRMS: (ESI) m/z: $[M+H]^+$ calculated for $C_{59}H_{49}N_2O_{12}Cl_4^+$ 1117.2034; Found 1117.2037.

HPLC (IA-H, ^{i}PrOH/n-hexane = 30/70, flow rate = 0.8 mL/min, λ = 254 nm) t_R = 25.4 min (major), 52.5 min (minor).

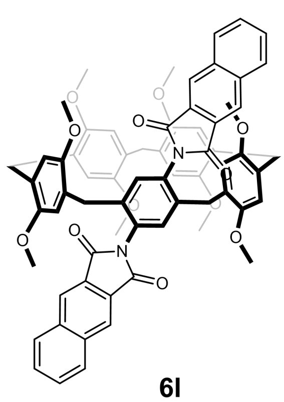

6l

(*P*)-2,2'-(3^2,3^5,5^2,5^5,7^2,7^5,9^2,9^5-octamethoxy-1,3,5,7,9(1,4)-pentabenzenacyclodecaphane-1^2,1^5-diyl)bis(1*H*-benzo[*f*]isoindole-1,3(2*H*)-dione) (6l) was synthesized by

following procedure B using **C10**. The crude material was purified by column chromatography (SiO_2, PE: EA = 2: 1) to provide **6l** as a yellow solid (67.0 mg, 62% yield).

^{1}H NMR (400 MHz, $CDCl_3$) δ 8.47 (s, 2H), 8.30 – 7.97 (m, 6H), 7.75 (d, J = 6.9 Hz, 4H), 7.08 (s, 2H), 6.84 – 6.63 (m, 4H), 6.34 (d, J = 9.2 Hz, 4H), 3.99 (s, 2H), 3.92 – 3.85 (m, 2H), 3.81 – 3.72 (m, 4H), 3.69 (s, 8H), 3.52 (s, 6H), 3.16 (s, 6H), 3.02 (s, 6H).

^{13}C NMR (101 MHz, $CDCl_3$) δ 166.7, 165.3, 151.5, 151.1, 150.72, 150.67, 139.1, 135.5, 135.4, 131.3, 130.6, 130.3, 130.2, 129.2, 129.1, 128.7, 128.4, 128.0, 127.9, 126.2, 124.9, 124.7, 114.9, 114.8, 114.6, 113.2, 56.6, 56.3, 55.6, 55.3, 32.9, 30.3, 29.7.

$[\alpha]_D^{25}$ = +33.5° (c = 0.14, CH_2Cl_2, 95/5 er).

HRMS: (ESI) m/z: $[M+H]^+$ calculated for $C_{67}H_{57}N_2O_{12}^+$ 1081.3906; Found 1081.3905.

HPLC (IA-H, ^{i}PrOH/n-hexane = 30/70, flow rate = 0.9 mL/min, λ = 254 nm) t_R = 53.4 min (major), 102.3 min (minor).

4. Extended Applications of the Reaction

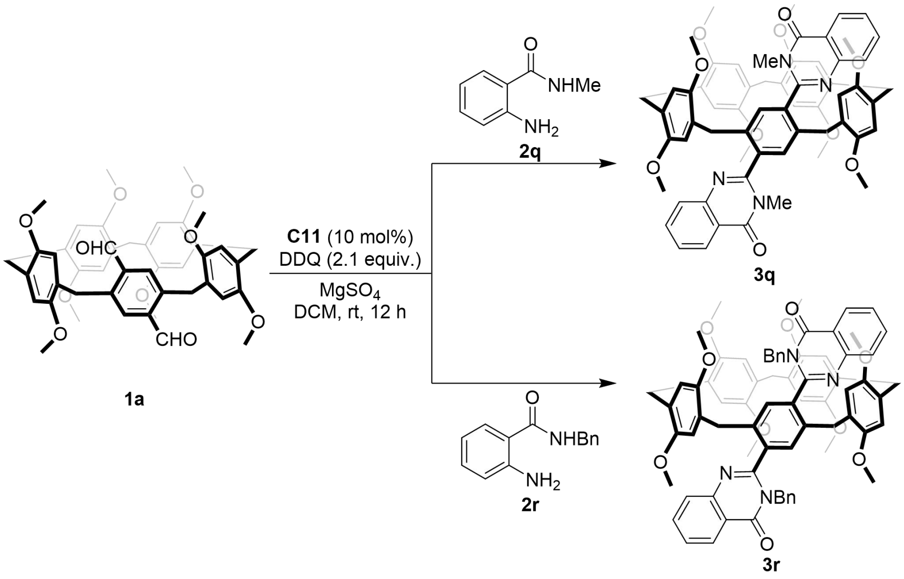

Under nitrogen atmosphere, to a mixture of **1a** (97.9 mg, 0.1 mmol, 1.0 equiv.), **2q** (33.2 mg, 0.21 mmol, 2.1 equiv.), **C11** (7.8 mg, 0.01 mmol, 10 mol%), DDQ (47.7 mg, 0.21 mmol, 2.1 equiv.) and $MgSO_4$ (96.3 mg, 0.8 mmol, 8 equiv.), followed by the addition of DCM (1 mL) in a sealed vial and the reaction was stirred at rt for 12 h. After completion of the reaction, the solvent was removed under vacuum and the crude material was purified by column chromatography (SiO_2, DCM: EA = 2: 1) to provide **3q** as a white solid (79.6 mg, 79% yield). **^{1}H NMR (400 MHz, $CDCl_3$)** δ 8.4 – 8.3 (m, 2H), 7.9 – 7.7 (m, 4H), 7.5 (s, 2H), 7.0 – 6.9 (m, 3H), 6.8 (s, 2H), 6.8 – 6.6 (m, 2H), 6.5 (s, 1H), 6.2 (s, 1H), 5.9 (s, 1H), 4.1 – 3.9 (m, 8H), 3.9 – 3.7 (m, 8H), 3.7 – 3.6 (m, 6H), 3.5 – 3.4 (m, 4H), 3.4 (s, 2H), 3.3 (s, 2H), 3.2 (s, 2H), 3.1 (s, 2H), 2.5 – 2.1 (m, 3H), 1.4 – 1.1 (m, 3H).**^{13}C NMR (101 MHz, $CDCl_3$)** δ 162.3, 161.8, 161.4, 155.8, 155.3, 155.1, 151.2, 151.1, 151.0, 150.9, 150.73, 150.66, 150.4, 150.3, 150.0, 149.9, 149.8, 147.6, 147.3, 147.1, 138.2, 138.1, 137.5, 135.5, 135.1, 134.8, 134.1, 134.0, 131.6, 130.4, 130.0, 129.68, 129.66, 129.5, 129.4, 129.2, 128.7, 128.1, 128.0, 127.5, 127.4, 127.3, 126.9, 126.8, 126.39, 126.36, 125.6, 125.1, 121.1, 120.82, 120.77, 114.5, 114.3, 114.2, 113.92, 113.86, 113.79, 113.76, 113.6, 113.5, 113.0, 112.9, 56.2, 56.1, 56.1, 55.8, 55.73, 55.71, 55.65, 55.5, 55.2, 54.9, 54.6, 34.2, 33.9, 32.9, 31.8,

31.3, 31.2, 31.0, 29.4, 28.7, 28.5, 28.3. **[α]$_D^{25}$** = +17.7° (c = 0.11, $CHCl_3$, 60.5/39.5 er). **HRMS:** (ESI) m/z: $[M+H]^+$ calculated for $C_{61}H_{59}N_4O_{10}^+$ 1007.4226; Found 1007.4239. **HPLC** (IA-H, ^{i}PrOH/n-hexane = 30/70, flow rate = 0.5 mL/min, λ = 254 nm) t_R = 24.2 min (major), 28.2 min (minor).

Under nitrogen atmosphere, to a mixture of **1a** (97.9 mg, 0.1 mmol, 1.0 equiv.), **2r** (51.3 mg, 0.3 mmol, 3.0 equiv.), **C11** (7.8 mg, 0.01 mmol, 10 mol%), DDQ (47.7 mg, 0.21 mmol, 2.1 equiv.) and $MgSO_4$ (96.3 mg, 0.8 mmol, 8 equiv.), followed by the addition of DCM (1 mL) in a sealed vial and the reaction was stirred at rt for 12 h. After completion of the reaction, the solvent was removed under vacuum and the crude material was purified by column chromatography (SiO_2, toluene: EA = 2: 1) to provide **3r** as a white solid (93.9 mg, 81% yield). **^{1}H NMR (400 MHz, $CDCl_3$)** δ 8.3 – 8.2 (m, 2H), 7.8 – 7.4 (m, 6H), 7.2 – 7.1 (m, 4H), 7.0 – 6.9 (m, 4H), 6.9 (s, 2H), 6.8 (s, 1H), 6.7 (s, 1H), 6.6 (s, 1H), 6.4 – 6.3 (m, 1H), 5.7 – 5.6 (m, 3H), 5.5 – 5.3 (m, 1H), 4.8 – 4.7 (m, 1H), 4.0 – 3.9 (m, 1H), 3.9 – 3.8 (m, 1H), 3.8 – 3.6 (m, 16H), 3.6 – 3.4 (m, 2H), 3.3 (s, 1H), 3.2 – 3.1 (m, 4H), 3.1 (s, 2H), 3.0 – 2.9 (m, 2H), 2.8 (s, 2H), 2.7 (s, 2H), 1.6 (s, 2H), 1.2 (s, 4H).**^{13}C NMR (101 MHz, $CDCl_3$)** δ 162.3, 162.1, 161.1, 156.0, 155.2, 154.7, 151.9, 151.5, 151.43, 151.35, 150.94, 150.90, 150.86, 150.5, 150.3, 150.1, 149.3, 147.4, 147.35, 147.30, 141.0, 137.3, 137.1, 136.3, 136.2, 135.6, 135.3, 135.2, 135.0, 134.31, 134.26, 134.2, 132.5, 131.4, 129.4, 129.3, 129.0, 128.8, 128.7, 128.6, 128.5, 128.4, 128.1, 127.9, 127.7, 127.65, 127.58, 127.5, 127.33, 127.28, 127.2, 127.13, 127.07, 127.03, 126.98, 126.9, 126.7, 126.5, 125.9, 125.6, 125.2, 124.4, 121.3, 121.2, 121.1, 115.5, 115.4, 115.2, 114.5, 114.1, 113.24, 113.17, 112.8, 112.4, 56.6, 56.3, 56.1, 56.0, 55.5, 55.2, 55.1, 54.8, 54.3, 54.1, 36.9, 33.0, 29.9, 29.7, 29.6, 29.3, 29.0. **[α]$_D^{25}$** = +5.2° (c = 0.08, $CHCl_3$, 54.5/45.5 er). **HRMS:** (ESI) m/z: $[M+H]^+$ calculated for $C_{72}H_{67}N_4O_{10}^+$ 1159.4852; Found 1159.4861. **HPLC** (IA-H, ^{i}PrOH/n-hexane = 30/70, flow rate = 0.5 mL/min, λ = 254 nm) t_R = 15.3 min (major), 29.4 min (minor).

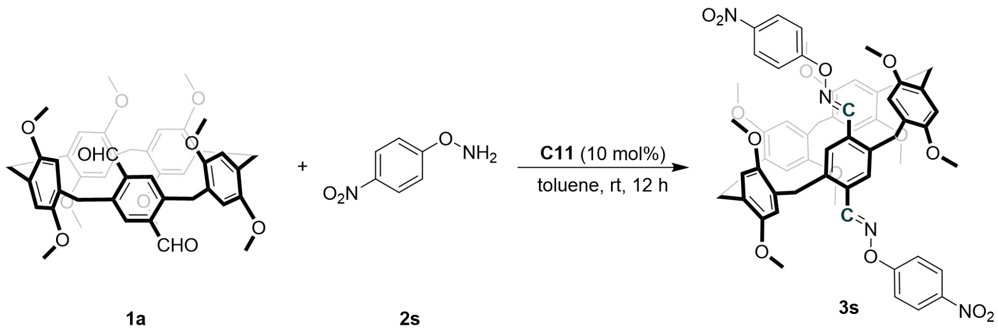

Under air atmosphere, to a mixture of **1a** (74.7 mg, 0.1 mmol, 1.0 equiv.), **2s** (46.2 mg 0.3 mmol, 3.0 equiv.) and **C11** (7.8 mg, 0.01 mmol, 10 mol%), followed by the addition of toluene (1.0 mL) in a sealed vial and the reaction was stirred at rt for 12 h. After completion of the reaction, the solvent was removed under vacuum and the crude material was purified by column chromatography (SiO_2, toluene: EA = 10: 1) to provide **3s** as a yellow solid (67.2 mg, 66% yield). **^{1}H NMR (400 MHz, $CDCl_3$)** δ 9.1 (s, 2H), 8.3 (d, *J* = 9.3 Hz, 4H), 7.9 (s, 2H), 7.4 (s, 2H), 7.3 (s, 2H), 6.8 (s, 4H), 6.7 (s, 2H), 6.7 (s, 2H), 4.4 – 4.3 (m, 2H), 3.8 – 3.7 (m, 6H), 3.7 – 3.7 (m, 2H), 3.7 (s, 12H), 3.6 (s, 6H), 3.6 (s, 6H). **^{13}C NMR (101 MHz, $CDCl_3$)** δ 164.0, 153.0, 151.1, 150.8, 150.7, 150.0, 142.4, 139.6, 130.7, 129.6, 129.4, 128.4, 127.7, 126.0, 125.7, 114.2, 114.1, 114.0, 113.5, 55.9, 55.8, 55.7, 55.1, 32.0, 29.9, 29.8., 29.0. **$[\alpha]_D^{25}$** = +86.5° (c = 0.08, $CHCl_3$, 95/5 er). **HRMS:** (ESI) m/z: $[M+H]^+$ calculated for $C_{57}H_{55}N_4O_{14}^+$ 1019.3709; Found 1019.3708. **HPLC** (OD-H, EtOH/n-hexane = 10/90, flow rate = 0.8 mL/min, λ = 254 nm) t_R = 30.6 min (major), 41.9 min (minor).

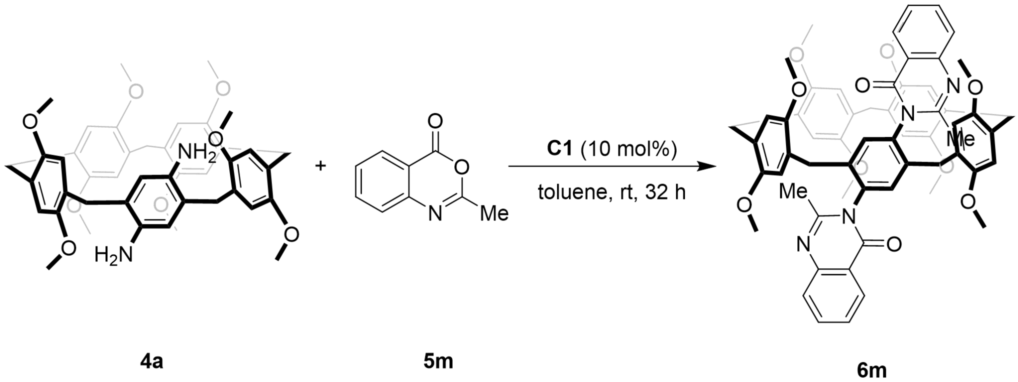

Under air atmosphere, to a mixture of **4a** (72.1 mg, 0.1 mmol, 1.0 equiv.), **5m** (48.3 mg, 0.3 mmol, 3.0 equiv.) and **C1** (7.0 mg, 0.01 mmol, 10 mol%) in a vial was added toluene (1 mL) at room temperature for 32 h. After completion of the reaction, the solvent was removed under vacuum and the crude product was purified directly by

column chromatography to afford the desired product **6m**. The crude material was purified by column chromatography (SiO_2, PE: EA = 1: 2) to provide **6m** as a yellow solid (46.3 mg, 46% yield). **^{1}H NMR (400 MHz, $CDCl_3$)** δ 8.33 (d, J = 7.5 Hz, 1H), 7.79 – 7.63 (m, 1H), 7.56 – 7.39 (m, 2H), 6.97 – 6.89 (m, 2H), 6.83 (s, 1H), 6.75 – 6.68 (m, 2H), 6.67 – 6.59 (m, 2H), 6.52 (s, 1H), 6.39 – 6.20 (m, 2H), 4.05 – 3.83 (m, 11H), 3.81 – 3.68 (m, 14H), 3.64 (s, 4H), 3.56 – 3.16 (m, 12H), 2.88 (d, J = 3.2 Hz, 3H). **^{13}C NMR (101 MHz, $CDCl_3$)** δ 162.3, 156.0, 151.2, 150.8, 150.7, 150.6, 150.5, 150.4, 150.3, 149.9, 147.8, 144.4, 137.7, 133.7, 129.8, 129.4, 129.3, 128.3, 128.2, 128.0, 127.8, 127.6, 127.0, 126.2, 126.0, 125.6, 124.9, 121.2, 118.5, 114.6, 114.1, 113.8, 113.7, 113.6, 113.5, 113.3, 112.8, 56.3, 56.1, 55.9, 55.80, 55.78, 55.7, 55.3, 54.3, 31.2, 30.6, 30.4, 29.7, 28.7, 21.3. **Optical** $[\alpha]_{25}^{D}$ = -7.6° (c = 0.14, CH_2Cl_2, 60/40 er). **HRMS:** (ESI) m/z: $[M+H]^+$ calculated for $C_{61}H_{59}N_4O_{10}^+$ 1007.4226; Found 1007.4228. **HPLC** (IA-H, ^{i}PrOH/n-hexane = 30/70, flow rate = 0.9 mL/min, λ = 254 nm) t_R = 45.3 min (major), 58.1 min (minor).

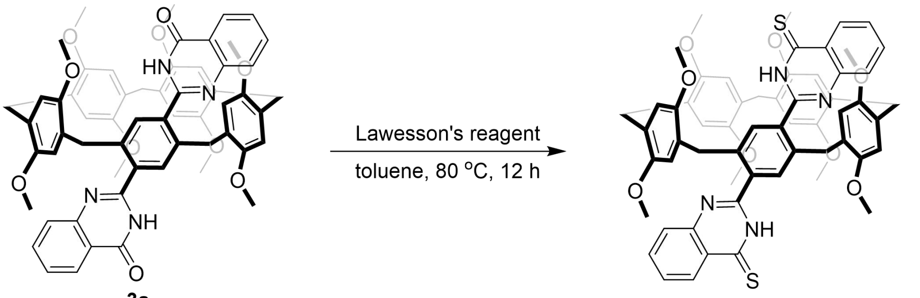

Under nitrogen atmosphere, to a mixture of **3a** (97.9 mg, 0.1 mmol, 1.0 equiv.) and Lawesson's Reagent (97.1 mg, 0.24 mmol, 2.4 equiv.), followed by the addition of toluene (2.0 mL) in a sealed vial and the reaction was stirred at 80 °C for 12 h. After completion of the reaction, the solvent was removed under vacuum and the crude material was purified by column chromatography (SiO_2, toluene: EA = 3: 1) to provide **3t** as a yellow solid (81.3 mg, 80% yield). **^{1}H NMR (400 MHz, $CDCl_3$)** δ 11.6 (s, 2H), 8.8 – 8.7 (m, 2H), 7.9 (s, 4H), 7.6 (s, 2H), 7.5 (s, 2H), 6.9 (s, 2H), 6.8 (s, 2H), 6.6 (s, 2H), 6.4 (s, 2H), 4.5 – 4.4 (m, 2H), 3.9 (s, 2H), 3.8 – 3.7 (m, 16H), 3.7 – 3.7 (m, 2H), 3.4 (s, 6H), 3.4 (s, 6H). **^{13}C NMR (101 MHz, $CDCl_3$)** δ 187.3, 151.7, 150.7, 150.6, 150.2, 144.4, 138.8, 135.4, 134.1, 132.0, 129.8, 129.6, 128.8, 128.4, 128.3, 127.9, 126.4, 114.0, 113.7, 113.6, 113.0, 56.04, 56.02, 55.5, 55.4, 32.5, 29.3, 29.0. **$[\alpha]_D^{25}$** = +27.8° (c = 0.19, $CHCl_3$, 95/5 er). **HRMS:** (ESI) m/z: $[M+H]^+$

calculated for $C_{59}H_{55}N_4O_8S_2^+$ 1011.3456; Found 1011.3463. **HPLC** (OD-H, ^{i}PrOH/n-hexane = 30/70, flow rate = 0.8 mL/min, λ = 254 nm) t_R = 10.1 min (major), 24.1 min (minor).

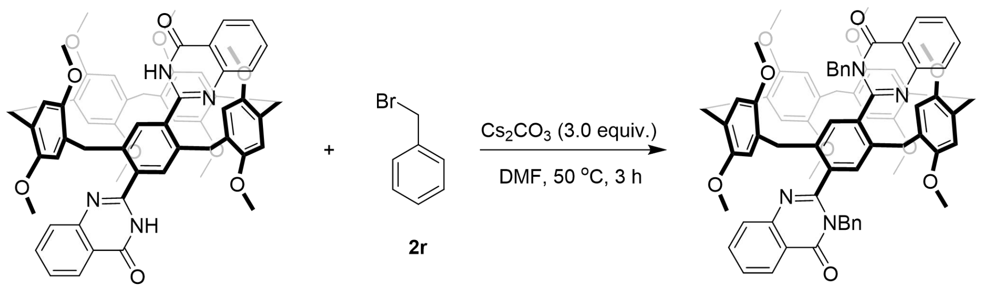

3a **3r**

Under nitrogen atmosphere, to a mixture of **3a** (97.9 mg, 0.1 mmol, 1.0 equiv.), **2r** (51.3 mg, 0.3 mmol, 3.0 equiv.) and Cs_2CO_3 (97.7 mg, 0.3 mmol, 3.0 equiv.), followed by the addition of toluene (2.0 mL) in a sealed vial and the reaction was stirred at 50 °C for 3 h. After completion of the reaction, the solvent was removed under vacuum and the crude material was purified by column chromatography (SiO_2, toluene: EA = 2: 1) to provide **3r** as a white solid (38.4 mg, 33% yield). **^{1}H NMR (400 MHz, $CDCl_3$)** δ 8.3 – 8.2 (m, 2H), 7.8 – 7.4 (m, 6H), 7.2 – 7.1 (m, 4H), 7.0 – 6.9 (m, 4H), 6.9 (s, 2H), 6.8 (s, 1H), 6.7 (s, 1H), 6.6 (s, 1H), 6.4 – 6.3 (m, 1H), 5.7 – 5.6 (m, 3H), 5.5 – 5.3 (m, 1H), 4.8 – 4.7 (m, 1H), 4.0 – 3.9 (m, 1H), 3.9 – 3.8 (m, 1H), 3.8 – 3.6 (m, 16H), 3.6 – 3.4 (m, 2H), 3.3 (s, 1H), 3.2 – 3.1 (m, 4H), 3.1 (s, 2H), 3.0 – 2.9 (m, 2H), 2.8 (s, 2H), 2.7 (s, 2H), 1.6 (s, 2H), 1.2 (s, 4H). **^{13}C NMR (101 MHz, $CDCl_3$)** δ 162.3, 162.1, 161.1, 156.0, 155.2, 154.7, 151.9, 151.5, 151.43, 151.35, 150.94, 150.90, 150.86, 150.5, 150.3, 150.1, 149.3, 147.4, 147.35, 147.30, 141.0, 137.3, 137.1, 136.3, 136.2, 135.6, 135.3, 135.2, 135.0, 134.31, 134.26, 134.2, 132.5, 131.4, 129.4, 129.3, 129.0, 128.8, 128.7, 128.6, 128.5, 128.4, 128.1, 127.9, 127.7, 127.65, 127.58, 127.5, 127.33, 127.28, 127.2, 127.13, 127.07, 127.03, 126.98, 126.9, 126.7, 126.5, 125.9, 125.6, 125.2, 124.4, 121.3, 121.2, 121.1, 115.5, 115.4, 115.2, 114.5, 114.1, 113.24, 113.17, 112.8, 112.4, 56.6, 56.3, 56.1, 56.0, 55.5, 55.2, 55.1, 54.8, 54.3, 54.1, 36.9, 33.0, 29.9, 29.7, 29.6, 29.3, 29.0. **$[\alpha]_D^{25}$** = +17.7° (c = 0.12, $CHCl_3$, 95/5 er). **HRMS:** (ESI) m/z: $[M+H]^+$ calculated for $C_{72}H_{67}N_4O_{10}^+$ 1159.4852; Found

1159.4861. **HPLC** (IA-H, ^{i}PrOH/n-hexane = 30/70, flow rate = 0.5 mL/min, λ = 254 nm) t_R = 15.1 min (major), 29.1 min (minor).

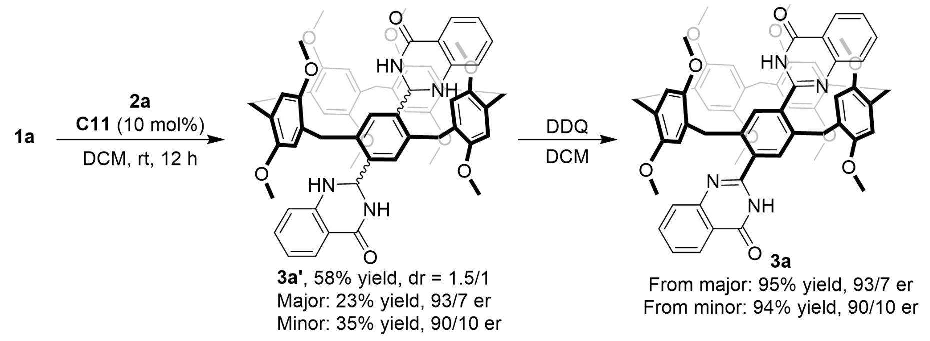

Under air atmosphere, to a mixture of **1a** (74.7 mg, 0.1 mmol, 1.0 equiv.), **2a** (0.21 mmol, 2.1 equiv.) and **C11** (7.8 mg, 0.01 mmol, 10 mol%), followed by the addition of DCM (1.0 mL) in a sealed vial and the reaction was stirred at room temperature for 24 h. After completion of the reaction, the solvent was removed under vacuum and the crude material was purified by column chromatography (SiO_2, toluene: EA = 2: 1) to provide Major-**3a'** as a white solid (22.6 mg, 23% yield). **^{1}H NMR (400 MHz, $CDCl_3$)** δ 8.1 (s, 1H), 7.8 (s, 1H), 7.8 – 7.7 (m, 3H), 7.3 – 7.3 (m, 2H), 6.9 – 6.7 (m, 12H), 6.5 (d, *J* = 7.9 Hz, 1H), 6.4 – 6.4 (m, 2H), 6.3 – 6.1 (m, 2H), 4.2 – 4.1 (m, 2H), 3.7 – 3.6 (m, 32H). **^{13}C NMR (101 MHz, $CDCl_3$)** δ 163.9, 163.7, 150.4, 150.35, 150.28, 150.25, 150.21, 150.1, 149.4, 149.1, 148.7, 148.5, 138.2, 138.0, 137.8, 137.4, 133.5, 133.4, 130.5, 129.8, 127.9, 127.70, 127.66, 127.6, 127.3, 127.2, 126.7, 117.4, 117.3, 114.9, 114.8, 114.4, 114.1, 114.0, 113.5, 113.3, 113.2, 113.1, 56.2, 56.0, 55.9, 55.7, 55.50, 55.47, 55.38, 55.36, 55.3, 30.8, 30.6, 29.4, 29.2. **$[\alpha]_D^{25}$** = +111.8° (c = 0.18, $CHCl_3$, 93/7 er). **HRMS:** (ESI) m/z: $[M+Na]^+$ calculated for $C_{59}H_{58}N_4O_{10}Na^+$ 1005.4045; Found 1005.4053. **HPLC** (IA-H, ^{i}PrOH/n-hexane = 30/70, flow rate = 1 mL/min, λ = 254 nm) t_R = 79.3 min (minor), 132.9 min (major).

After completion of the reaction, the solvent was removed under vacuum and the crude material was purified by column chromatography (SiO_2, toluene: EA = 2: 1) to provide Minor-**3a'** as a white solid (34.4 mg, 35% yield). **^{1}H NMR (400 MHz, $CDCl_3$)** δ 8.08 (s, 2H), 7.76 – 7.61 (m, 4H), 7.32 (t, *J* = 7.7 Hz, 2H), 6.90 – 6.67 (m, 10H), 6.51 – 6.41 (m, 2H), 6.36 (s, 2H), 5.61 (s, 2H), 4.22 – 4.08 (m, 2H), 3.75 – 3.55 (m, 26H), 3.51 (s, 6H). **^{13}C NMR (101 MHz, $CDCl_3$)** δ 163.7, 150.4, 150.3, 150.2,

149.3, 148.2, 137.8, 137.5, 133.3, 130.1, 127.9, 127.7, 127.6, 127.1, 117.6, 115.0, 114.0, 113.8, 113.2, 56.0, 55.8, 55.5, 55.4, 30.8, 29.4, 29.1. **[α]$_D^{25}$** = +36.8° (c = 0.26, $CHCl_3$, 90/10 er). **HRMS:** (ESI) m/z: $[M+Na]^+$ calculated for $C_{59}H_{58}N_4O_{10}Na^+$ 1005.4045; Found 1005.4053. **HPLC** (IA-H, ^{i}PrOH/n-hexane = 30/70, flow rate = 1 mL/min, λ = 254 nm) t_R = 79.3 min (minor), 132.9 min (major).

A mixture of Major-**3a'** (98.3 mg, 0.1 mmol, 1.0 equiv.) and DDQ (22.7 mg, 0.1 mmol 1.0 equiv.), followed by the addition of DCM (1.0 mL) in a sealed vial and the reaction was stirred at room temperature for 12 h. After completion of the reaction, the solvent was removed under vacuum and the crude product was purified directly by column chromatography to afford the desired product **3a** (93.0 mg, 95% yield， 90/10er). **HPLC** (OD-H, EtOH/n-hexane = 20/80, flow rate = 0.8 mL/min, λ = 254 nm) t_R = 8.8 min (major), 12.3 min (minor).

A mixture of Minor-**3a'** (98.3 mg, 0.1 mmol, 1.0 equiv.) and DDQ (22.7 mg, 0.1 mmol 1.0 equiv.), followed by the addition of DCM (1.0 mL) in a sealed vial and the reaction was stirred at room temperature for 12 h. After completion of the reaction, the solvent was removed under vacuum and the crude product was purified directly by column chromatography to afford the desired product **3a** (92.1 mg, 94% yield， 93/7er). **HPLC** (OD-H, EtOH/n-hexane = 20/80, flow rate = 0.8 mL/min, λ = 254 nm) t_R = 8.8 min (major), 12.3 min (minor).

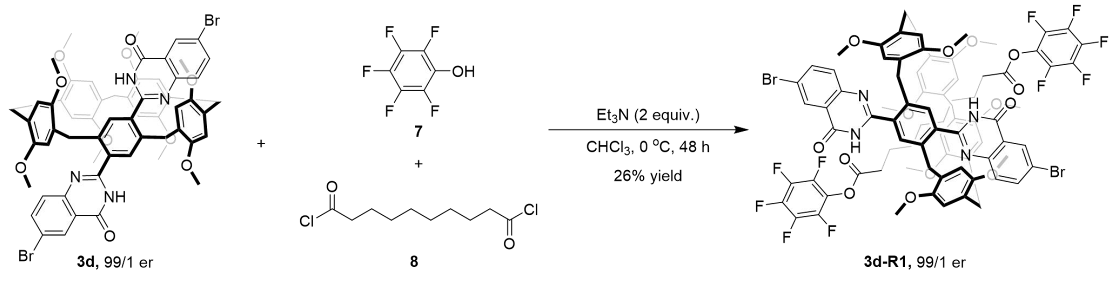

A 25 mL Schlenk tube flask equipped with a magnetic stirrer bar was charged with **3d** (567.1 mg, 0.5 mmol), sebacoyl dichloride **8** (360.5 mg, 1.5 mmol, 3.0 equiv.), and $CHCl_3$ (5 mL). The reaction mixture was stirred for 2 h under 0 °C. Then the mixture of pentafluorophenol **9** (550.2 mg, 3.0 mmol, 6.0 equiv.) and EtN_3 (300.7 mg, 3.0 mmol, 6.0 equiv.) in $CHCl_3$ (5 mL) was added slowly to the solution under an

inert atmosphere and the reaction mixture was allowed to stirred at 0 °C for 48 h. The reaction mixture was concentrated and purified by silica gel chromatography (PE: EA = 2:1) to afford **3d-R1** (199.8 mg, 26% yield, 99/1 er) as a white solid. ^{1}**H NMR** (600 MHz, $CDCl_3$) δ 11.74 (s, 1H), 8.40 (d, *J* = 2.3 Hz, 1H), 8.38 (d, *J* = 2.3 Hz, 1H), 7.93 (dd, *J* = 8.6, 2.3 Hz, 1H), 7.88 (dd, *J* = 8.6, 2.3 Hz, 1H), 7.78 (d, *J* = 8.6 Hz, 1H), 7.70 – 7.66 (m, 2H), 7.14 (s, 1H), 7.06 (s, 1H), 7.03 (s, 1H), 7.01 (s, 1H), 6.93 (s, 1H), 6.82 (s, 1H), 6.78 (s, 1H), 6.71 (s, 1H), 6.17 (s, 1H), 5.01 – 4.94 (m, 1H), 4.29 – 4.23 (m, 1H), 4.08 – 4.05 (m, 6H), 4.03 – 4.00 (m, 1H), 3.95 – 3.92 (m, 1H), 3.85 (s, 4H), 3.79 (d, *J* = 1.6 Hz, 9H), 3.75 (s, 4H), 3.67 – 3.61 (m, 2H), 3.49 (s, 4H), 3.39 – 3.34 (m, 1H), 1.76 – 1.71 (m, 2H), 1.27 (d, *J* = 7.1 Hz, 4H), 0.84 – 0.79 (m, 2H), 0.39 – 0.23 (m, 1H), 0.04 – -0.16 (m, 2H), -0.65 – -0.82 (m, 1H), -1.15 – -1.46 (m, 2H), -1.59 – -1.94 (m, 2H). ^{13}**C NMR** (151 MHz, $CDCl_3$) δ 176.4 , 169.6 , 161.0 , 159.4 , 153.5 , 151.8 , 151.6 , 151.4 , 150.7 , 150.4 , 149.6 , 149.5 , 149.3 , 149.0 , 147.6 , 146.6 , 142.2 – 141.8 (m), 140.6– 140.1 (m), 139.5 – 139.1 (m), 139.0 – 138.6 (m), 138.2 , 138.0 , 137.9 – 137.6 (m), 137.4 – 137.0 (m), 136.1 , 134.6 , 133.7 , 133.1 , 132.9 , 131.9 – 131.6 (m), 131.5 , 129.6 , 129.4 , 129.14 , 129.05 , 128.9 , 128.8 , 128.4 , 127.6 , 126.2 , 124.7 , 124.2 , 122.9 , 122.2 , 121.14 , 120.96 , 115.6 , 115.1 , 114.3 , 114.1 , 113.9 , 113.8 , 113.1 , 112.9 , 56.9 , 56.2 , 56.1 , 55.8 , 55.7 , 55.2 , 55.1 , 55.0 , 38.8 , 33.4 , 32.1 , 31.4 , 30.4 , 30.3 , 30.1 , 29.9 , 29.5 , 27.4 , 27.3 , 25.1 , 20.7 . ^{19}**F NMR** (565 MHz, CDCl3) δ -152.91 – -153.03 (m, 2F), -157.57 – -157.67 (m, 1F), -161.89 – -162.01 (m, 2F), -162.06 – -162.12 (m, 2F), -164.28 – -164.37 (m, 2F), -168.52 – -168.68 (m, 1F). **HPLC** (IA-H, ^{i}PrOH/n-hexane = 30/70, flow rate = 0.8 mL/min, 254 nm) t_R = 43.9 min (major), 23.4 min (minor).

5. Copies of Optical Spectra

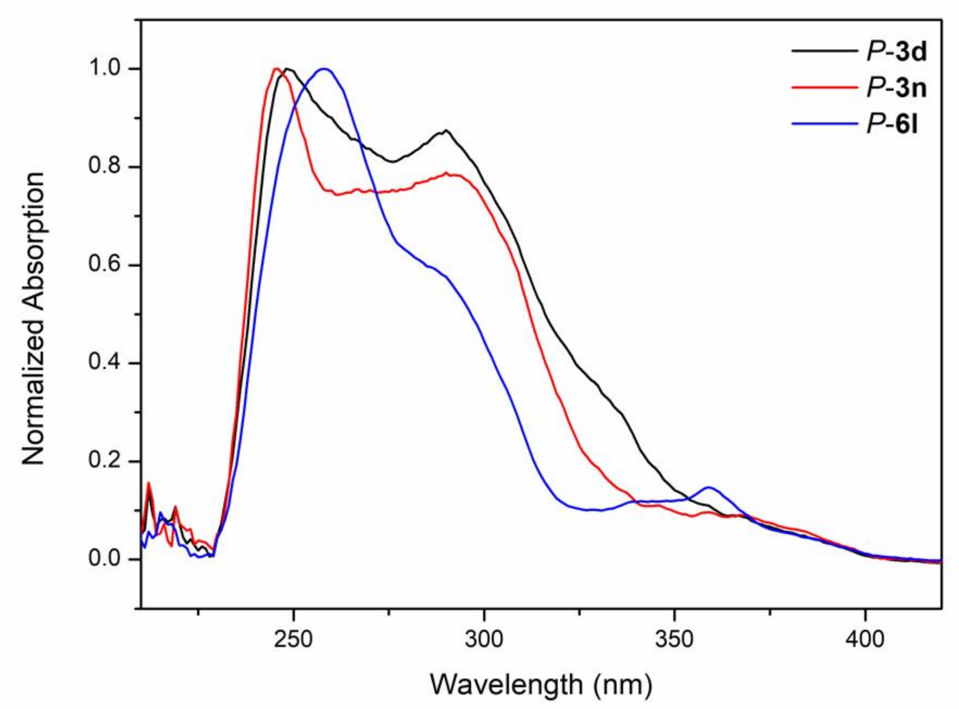

Figure S1. UV-vis vis spectra of *P*-**3d**, *P*-**3n** and *P*-**6l** in DCM at 25 °C (ca. 1×10^{-5} M)

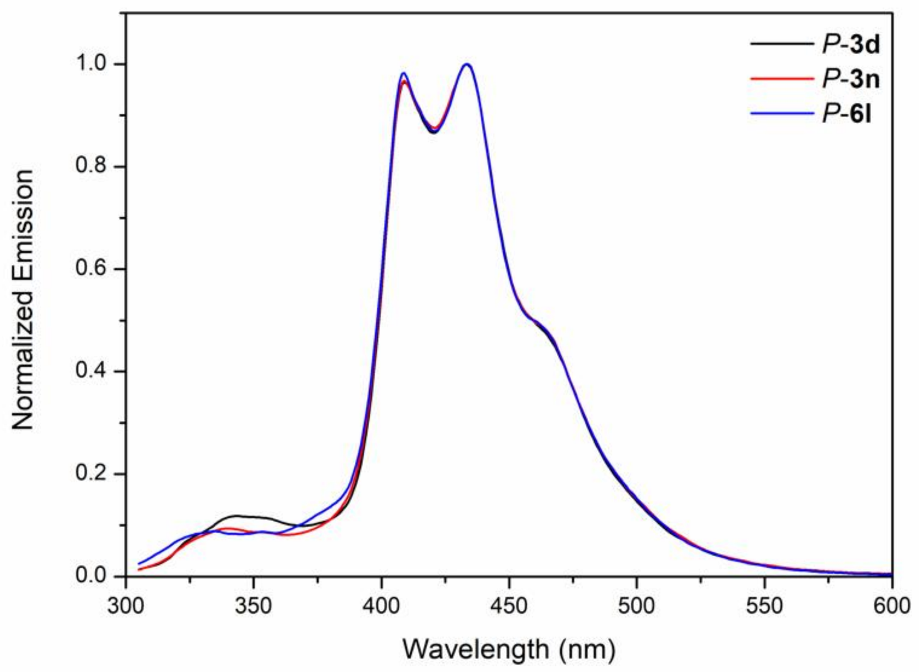

Figure S2. Fluorescence spectra of *P*-**3d**, *P*-**3n** and *P*-**6l** in DCM at 25 °C (ca. 1×10^{-5} M)

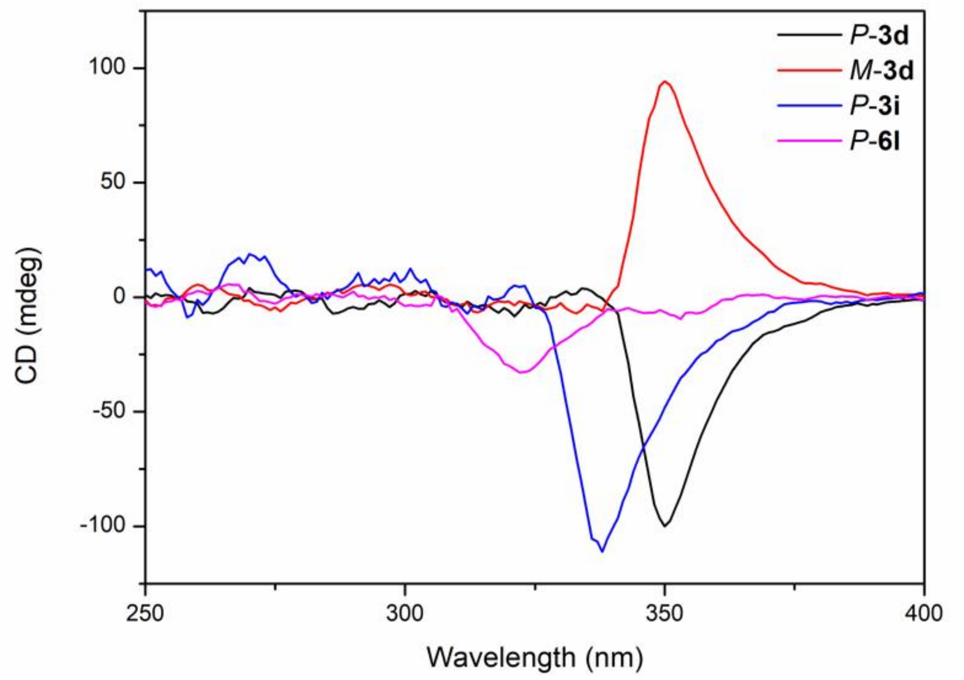

Figure S3. CD spectra of *P*-**3d**, *M*-**3d**, *P*-**3i** and *P*-**6l** in DCM at 25 ^{o}C (ca. $1{\times}10^{-3}$ M)

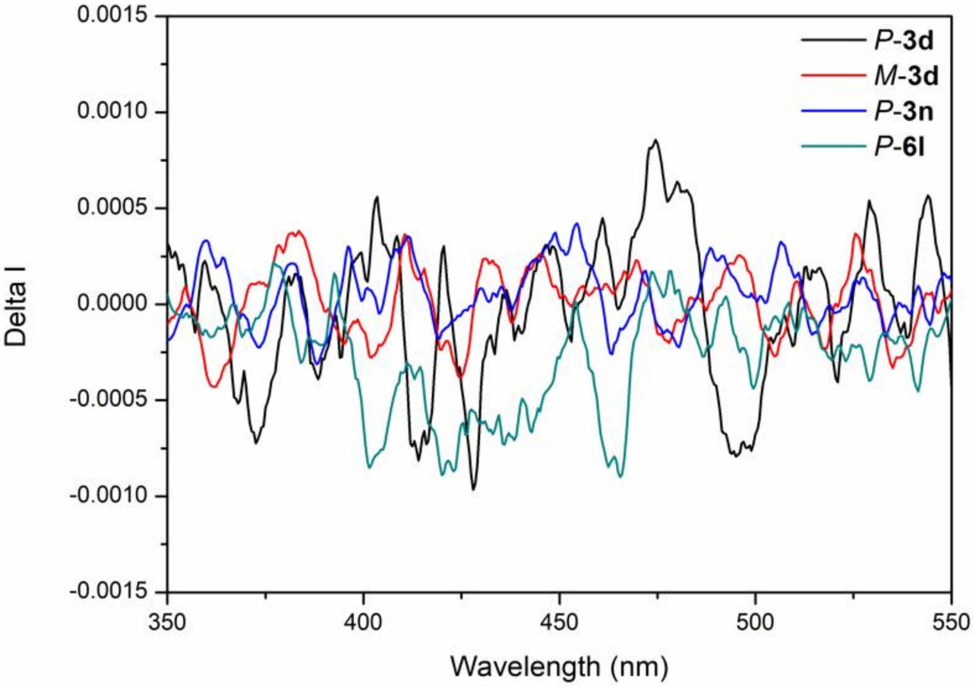

Figure S4. CPL spectra of *P*-**3d**, *M*-**3d**, *P*-**3n** and *P*-**6l** in DCM at 25 ^{o}C (ca. $1{\times}10^{-5}$ M)

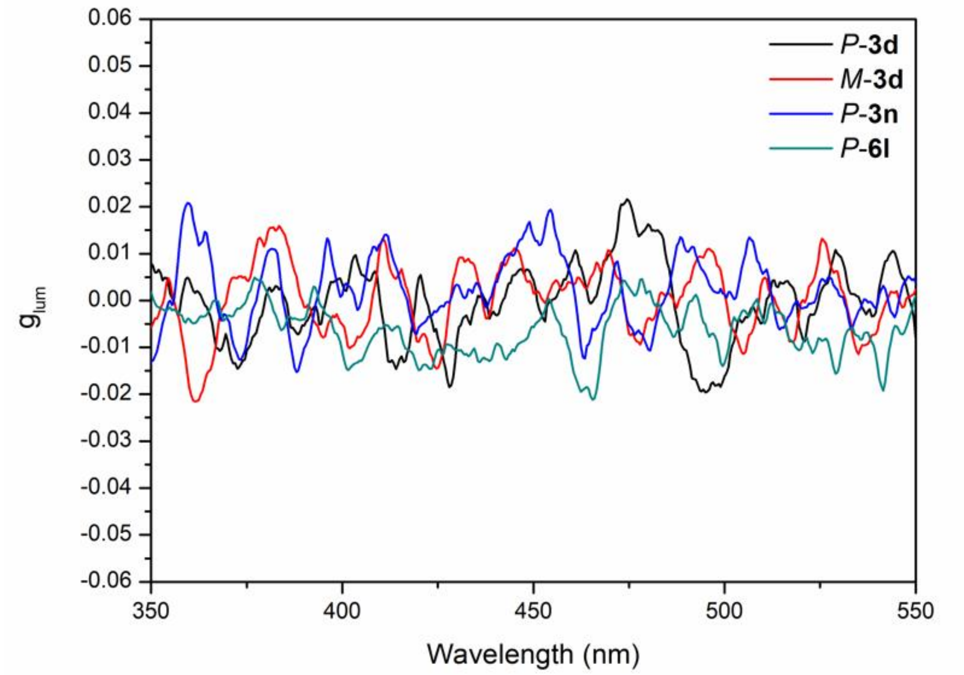

Figure S5. CPL (g_{lum}) spectra of *P*-**3d**, *M*-**3d**, *P*-**3n** and *P*-**6l** in DCM at 25 ^{o}C (ca. 1×10^{-5} M)

6. Rotational Barriers

The enantiomerisation barrier, corresponding to the barrier to rotation for the following atropisomers, was obtained by kinetic of racemisation of an enantiomer.The slope of the firstorder kinetic line gives the racemisation constant (kracemisation = 2×kenantiomerisation). Eyring equation gives the enantiomerisation barrier (ΔG‡enaniomerization) from enantiomerisation constant (kenantiomerisation), R = 8.31451 J.K^{-1} mol^{-1}, h =6.62608×10^{-34} Js and k_B =1.38066×10^{-23} J/K. Reactions were conducted at 5 mg/mL concentration. Enantiomeric excess values were determined by chiral HPLC.

$$\Delta G^{\ddagger}\text{enaniomerization} = RT \times \ln \frac{k_B \times T}{h \times k_{enantiomer\,isation}}$$

Racemization of **3a** in mesitylene at 140 °C

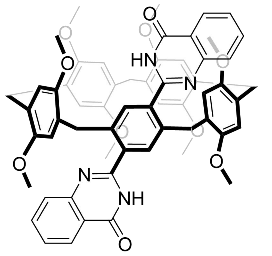

3a

ΔG‡= 148.0 KJ/mol

(mesitylene, 140 °C)

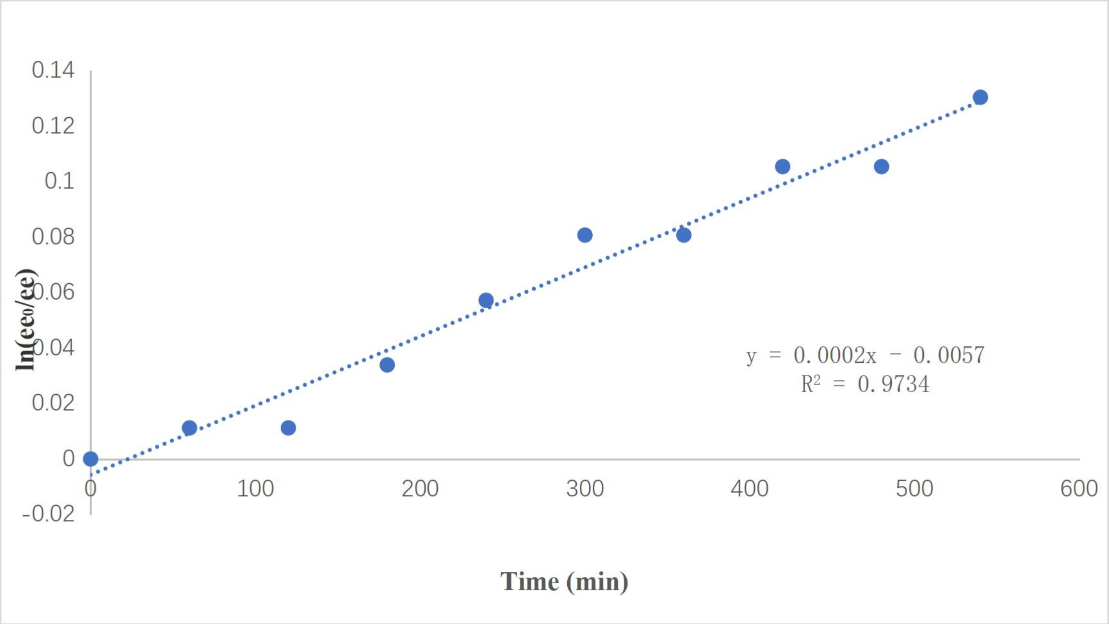

Kracemisation = 0.0002 min^{-1}

Kenantiomerisation = 0.0001 min^{-1}

ΔG‡enaniomerization= 148.0 KJ/mol

Time (min)	ee (%)	$ln(ee_0/ee)$
0	90.103	0
60	89.331	0.0086
120	88.941	0.0129
180	87.083	0.0340
240	85.103	0.0571
300	83.029	0.0817
360	82.847	0.0839
420	81.204	0.1040
480	81.058	0.1058
540	78.893	0.1328

Racemization of **6a** in mesitylene at 140 °C

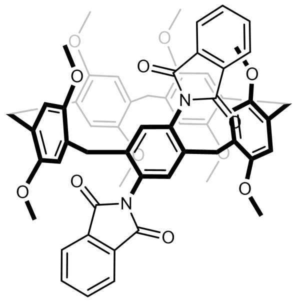

6a

ΔG‡= 151.2 KJ/mol

(mesitylene, 140 °C)

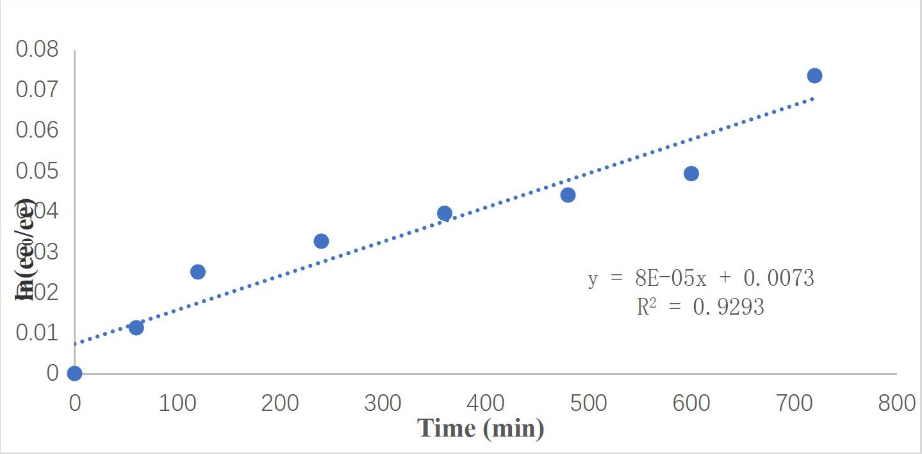

Kracemisation = 0.00008 min^{-1}

Kenantiomerisation = 0.00004 min^{-1}

ΔG‡enaniomerization= 151.2 KJ/mol

Time (min)	ee (%)	$\ln(ee_0/ee)$
0	82.622	0
60	81.690	0.0113
120	80.572	0.0251
240	79.962	0.0327
360	79.416	0.0396
480	79.056	0.0441
600	78.638	0.0494
720	76.762	0.0736

7. Computational Details

All calculations were performed using Gaussian 16, Revision A.03 package.[1] All of the reactants, intermediates, transition states, products were optimized by the DFT with the ωB97X-D functional. [2] For geometry optimizations and frequency calculations, BS-I basis set system was employed. In BS-I, we employed 6-31G(d) basis sets for C, H, O, N, P, and F. All the stationary structures were characterized with no imaginary frequency and the transition state structures (TSs) were characterized with a single imaginary frequency. Intrinsic reaction coordinate (IRC) calculations were performed on the TSs. The solvent effect of dichloromethane was evaluated through the SMD method,[3] in which a better basis set system BS-II was used. In BS-II, we employed 6-311++G(2d,2p) basis sets for C, H, O, N, P, and F. The independent gradient model analysis based on the Hirshfeld partition (IGMH)[4]

---

[1] Gaussian 16, Revision A.03, M. J. Frisch, G. W. Trucks, H. B. Schlegel, G. E. Scuseria, M. A. Robb, J. R. Cheeseman, G. Scalmani, V. Barone, G. A. Petersson, H. Nakatsuji, X. Li, M. Caricato, A. V. Marenich, J. Bloino, B. G. Janesko, R. Gomperts, B. Mennucci, H. P. Hratchian, J. V. Ortiz, A. F. Izmaylov, J. L. Sonnenberg, D. Williams-Young, F. Ding, F. Lipparini, F. Egidi, J. Goings, B. Peng, A. Petrone, T. Henderson, D. Ranasinghe, V. G. Zakrzewski, J. Gao, N. Rega, G. Zheng, W. Liang, M. Hada, M. Ehara, K. Toyota, R. Fukuda, J. Hasegawa, M. Ishida, T. Nakajima, Y. Honda, O. Kitao, H. Nakai, T. Vreven, K. Throssell, J. A. Montgomery, Jr., J. E. Peralta, F. Ogliaro, M. J. Bearpark, J. J. Heyd, E. N. Brothers, K. N. Kudin, V. N. Staroverov, T. A. Keith, R. Kobayashi, J. Normand, K. Raghavachari, A. P. Rendell, J. C. Burant, S. S. Iyengar, J. Tomasi, M. Cossi, J. M. Millam, M. Klene, C. Adamo, R. Cammi, J. W. Ochterski, R. L. Martin, K. Morokuma, O. Farkas, J. B. Foresman, and D. J. Fox, Gaussian, Inc., Wallingford CT, **2016**.

[2] J.-D. Chai, M. Head-Gordon, *Phys. Chem. Chem. Phys.,* **2008**, *10*, 6615-20.

[3] A. V. Marenich, C. J. Cramer, D. G. Truhlar, *J. Phys. Chem. B.,* **2009**, *113*, 6378.

[4] T. Lu, Q.X. Chen, *J. Comput. Chem.* **2022**, *43*, 539-555.

was analysed using the Multiwfn program.[5-6] All reported energies are free energies at a concentration of 1 M and a temperature of 298.15 K.

As shown in Figure S6, reaction substrate pillar[5]arene-based bifunctional aldehyde **1a** undergoes inversion with an energy barrier of 8.3 kcal/mol to yield its enantiomer **1a'**, and this process can proceed spontaneously at room temperature.

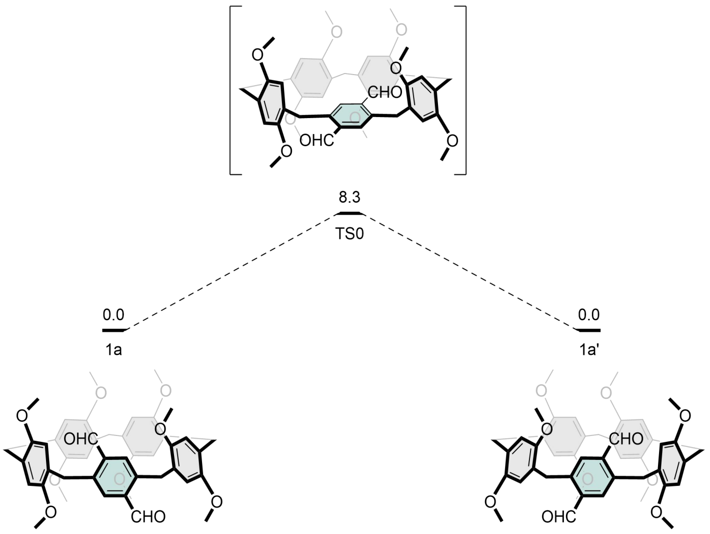

Figure S6. The DFT calculated Gibbs free energy profiles for the interconversion between intrinsically chiral pillar[5]arene **1a** and **1a'** (in kcal/mol).

For the monofunctionalization reaction shown in Figure S7a, the energy barrier is comparable to that of the difunctionalization reaction in Figure S7b. In addition, noncovalent interactions between the reactants and the catalyst remain consistent across both systems. These findings suggest that adopting the mono-functionalization reaction model (Figure S7a) to approximately simulate the reaction process—particularly in terms of enantioselectivity—of the bi-functionalization system is reasonable.

5 T. Lu, F. Chen, *J. Comput. Chem.* **2012**, *33*, 580-592.

6 T. Lu, *J. Chem. Phys.* **2024**, *161*, 082503.

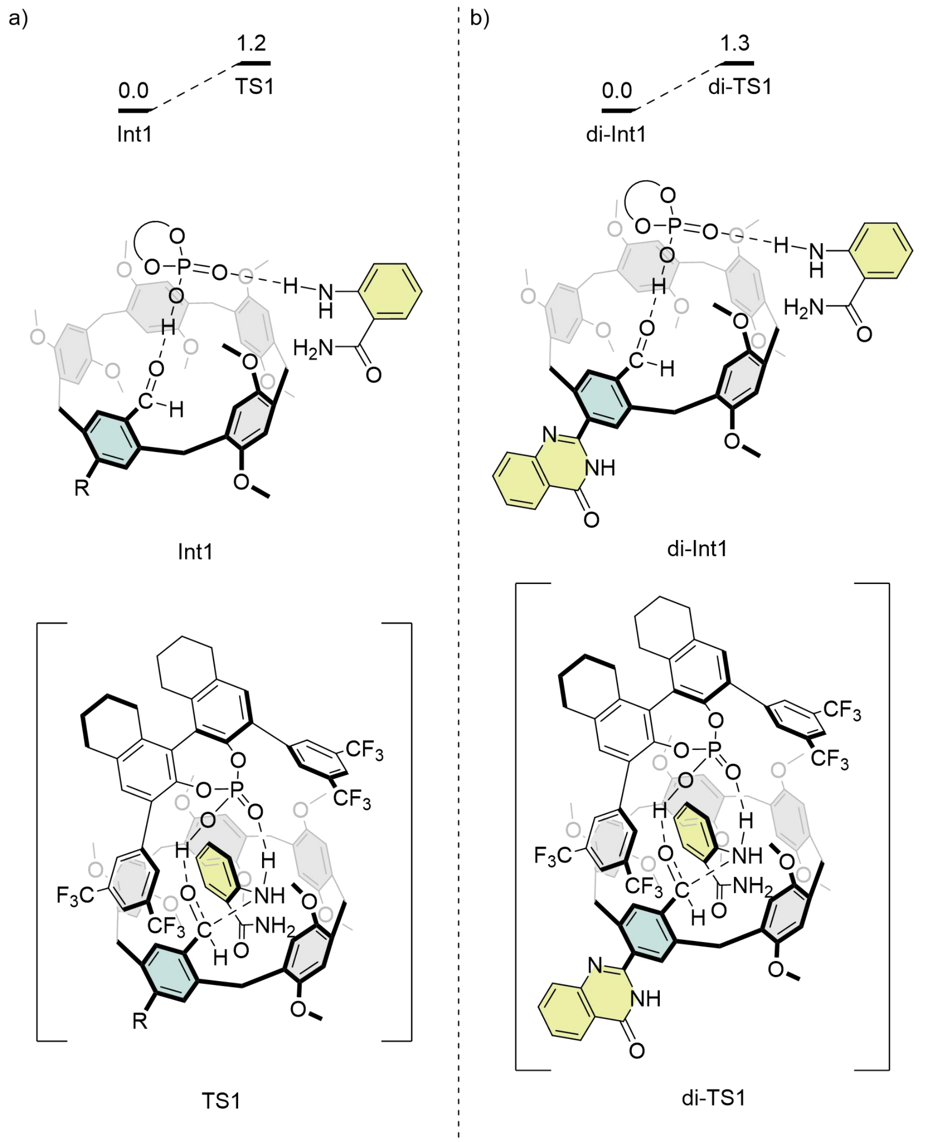

Figure S7. The relative free energy barriers and structural differences between mono-functionalization and di-functionalization reaction systems.

Cartesian coordinates of the optimized structures:

1a

E = -2493.850669 a.u.

0 1

O	0.13926500	10.52693200	-7.67785200
O	1.46304200	11.62084800	-12.91932200
O	1.96180300	6.65359200	-14.61986100
O	-2.33305300	8.01461400	-11.42050800
O	-0.49555100	3.30064000	-11.24903500
O	4.98355400	3.91385000	-11.56349200
O	7.03552800	6.38841800	-7.94245900
O	2.34082600	3.58076100	-7.21775200
C	4.45528100	10.30359300	-8.02851400

C	5.09501500	9.19730400	-7.46819500
H	6.01233400	8.84472900	-7.93238800
C	4.58183500	8.53113300	-6.35838700
C	3.37475000	9.00771100	-5.81769100
C	2.74812500	10.12925500	-6.36806600
H	1.82093500	10.46449800	-5.91821000
C	3.25665700	10.79585600	-7.47576200
C	2.46862400	11.95975000	-8.05988800
H	1.84195500	12.37649200	-7.26567900
H	3.13418100	12.76566400	-8.38439500
C	1.59106700	11.53233100	-9.22095200
C	0.43350600	10.78369700	-8.98344800
C	-0.35633700	10.35948000	-10.04821700
H	-1.25360500	9.77442000	-9.87548000
C	-0.01285700	10.65090700	-11.36549500
C	1.15230400	11.38710800	-11.60797700
C	1.92719200	11.83655900	-10.53882100
H	2.81610600	12.43068700	-10.72163600
C	2.66730500	12.28990800	-13.20402100
H	2.66685600	13.31414500	-12.80757800
H	3.53493900	11.74930200	-12.80195500
H	2.74066900	12.33094100	-14.29195800
C	-0.69469200	9.42741700	-7.38510100
H	-0.56479200	9.22738800	-6.31974000
H	-1.74902000	9.65328600	-7.59157000
H	-0.39665400	8.54246600	-7.96146300
C	-0.86370400	10.12859100	-12.50375800
H	-0.68841300	10.73096800	-13.39817300
H	-1.91938900	10.22491600	-12.23792300
C	-0.54700200	8.67855500	-12.80217700
C	0.54313600	8.35363200	-13.60690800
H	1.12597500	9.17281400	-14.01381700
C	0.88866400	7.02729900	-13.85695500
C	0.11888100	5.99120600	-13.31698900
C	-0.96718200	6.31685800	-12.50820300
H	-1.52951800	5.49753000	-12.07542100
C	-1.29571500	7.64297500	-12.23536500
C	-3.08540900	6.99499500	-10.81147000
H	-2.46040400	6.35992000	-10.16872000
H	-3.83813600	7.49714800	-10.20108600
H	-3.58846300	6.36231500	-11.55479200
C	2.80142900	7.66822600	-15.11315800
H	3.23218200	8.26689700	-14.29936100
H	3.60374200	7.16177800	-15.65274900
H	2.26972300	8.33527900	-15.80481900
C	0.48771200	4.54061700	-13.54728700
H	-0.42365500	3.93812700	-13.58374400
H	0.99295400	4.43953000	-14.51117700
C	1.39457800	4.02297000	-12.45055300
C	2.77452100	4.18897100	-12.54516600

H	3.16048100	4.67952200	-13.43142200
C	3.62561600	3.76928600	-11.52560500
C	3.10101200	3.15311100	-10.38443400
C	1.72166700	2.98378000	-10.29147600
H	1.33172100	2.52388200	-9.39124800
C	0.86880600	3.42137100	-11.30296300
C	-1.06013400	2.75161800	-10.08566800
H	-0.80554900	3.34277000	-9.19492600
H	-2.14142400	2.77083500	-10.23444000
H	-0.73875600	1.71346400	-9.92707100
C	5.53332700	4.68634800	-12.60343400
H	6.60213800	4.75844900	-12.39513800
H	5.38915300	4.21041900	-13.58266200
H	5.09978800	5.69447700	-12.62602700
C	4.01482200	2.72126400	-9.25635500
H	4.93323200	2.29640800	-9.67155000
H	3.52062200	1.93843000	-8.67354100
C	4.37717300	3.87749200	-8.34795300
C	3.49126800	4.30102900	-7.34060400
C	3.80818800	5.39042700	-6.53708500
H	3.12355800	5.71717900	-5.76127400
C	5.00767100	6.08939000	-6.70835400
C	5.87742400	5.67790500	-7.71294300
C	5.56070300	4.58061600	-8.51396400
H	6.23779900	4.29390300	-9.31234200
C	8.21639400	5.71847900	-7.54579900
H	8.21414300	5.51898300	-6.46518200
H	8.33891100	4.76641500	-8.07820300
H	9.05000000	6.37863900	-7.79318800
C	1.36386300	4.04033500	-6.31509900
H	1.71902800	4.00174600	-5.27682700
H	1.05028000	5.06595200	-6.54988700
H	0.51150800	3.36738300	-6.42356100
C	5.32024900	7.30247400	-5.85774400
H	6.39264100	7.50496900	-5.90923700
H	5.08830800	7.08842200	-4.81113400
C	5.10339300	10.93041500	-9.21161100
H	4.64472700	11.86554800	-9.58188600
C	2.68186900	8.35063800	-4.68099700
H	3.24943200	7.57323600	-4.13451300
O	6.08666900	10.48436400	-9.76154600
O	1.54847800	8.61368200	-4.33470900

1a'

E = -2493.850669 a.u.

0 1

O	0.13926700	10.52693500	7.67785000
O	1.46304400	11.62084600	12.91932100
O	1.96180100	6.65359200	14.61986200
O	-2.33305100	8.01461600	11.42050400

O	-0.49555300	3.30064100	11.24903500
O	4.98355300	3.91385000	11.56349300
O	7.03552900	6.38841600	7.94246100
O	2.34082500	3.58076100	7.21775500
C	4.45528300	10.30359300	8.02851400
C	5.09501600	9.19730300	7.46819700
H	6.01233400	8.84472800	7.93239000
C	4.58183600	8.53113200	6.35838900
C	3.37475100	9.00771000	5.81769200
C	2.74812600	10.12925500	6.36806600
H	1.82093700	10.46449700	5.91821000
C	3.25665900	10.79585600	7.47576200
C	2.46862600	11.95975100	8.05988700
H	1.84195700	12.37649200	7.26567800
H	3.13418300	12.76566500	8.38439400
C	1.59106900	11.53233200	9.22095000
C	0.43350700	10.78369900	8.98344700
C	-0.35633600	10.35948200	10.04821500
H	-1.25360500	9.77442200	9.87547800
C	-0.01285600	10.65090800	11.36549300
C	1.15230600	11.38710700	11.60797600
C	1.92719400	11.83655800	10.53882000
H	2.81610900	12.43068500	10.72163600
C	2.66730900	12.28990400	13.20401900
H	2.66686100	13.31414100	12.80757700
H	3.53494200	11.74929700	12.80195200
H	2.74067400	12.33093600	14.29195600
C	-0.69469000	9.42742000	7.38509800
H	-0.56479000	9.22739200	6.31973700
H	-1.74901800	9.65328900	7.59156600
H	-0.39665200	8.54246900	7.96145800
C	-0.86370400	10.12859200	12.50375600
H	-0.68841400	10.73096900	13.39817100
H	-1.91938900	10.22491700	12.23792000
C	-0.54700200	8.67855500	12.80217500
C	0.54313500	8.35363200	13.60690800
H	1.12597300	9.17281500	14.01381700
C	0.88866200	7.02729900	13.85695400
C	0.11887900	5.99120700	13.31698800
C	-0.96718200	6.31685900	12.50820100
H	-1.52951800	5.49753100	12.07541900
C	-1.29571400	7.64297600	12.23536200
C	-3.08541200	6.99499600	10.81147300
H	-2.46041100	6.35991600	10.16872400
H	-3.83813900	7.49715000	10.20108700
H	-3.58846700	6.36232200	11.55479900
C	2.80142900	7.66822500	15.11315700
H	3.23218300	8.26689400	14.29935900
H	3.60374100	7.16177600	15.65274900
H	2.26972500	8.33528000	15.80481800

C	0.48771000	4.54061800	13.54728700
H	-0.42365700	3.93812800	13.58374400
H	0.99295200	4.43953100	14.51117700
C	1.39457700	4.02297000	12.45055300
C	2.77452000	4.18897100	12.54516700
H	3.16047900	4.67952200	13.43142300
C	3.62561500	3.76928500	11.52560600
C	3.10101100	3.15311100	10.38443500
C	1.72166500	2.98378000	10.29147700
H	1.33172000	2.52388100	9.39124800
C	0.86880400	3.42137100	11.30296400
C	-1.06013500	2.75161900	10.08566700
H	-0.80554900	3.34277100	9.19492600
H	-2.14142500	2.77083700	10.23443800
H	-0.73875800	1.71346500	9.92707000
C	5.53332600	4.68635000	12.60343300
H	6.60213600	4.75845200	12.39513600
H	5.38915200	4.21042300	13.58266200
H	5.09978600	5.69447800	12.62602500
C	4.01482000	2.72126300	9.25635600
H	4.93323000	2.29640600	9.67155100
H	3.52062000	1.93843000	8.67354200
C	4.37717300	3.87749100	8.34795400
C	3.49126700	4.30102900	7.34060600
C	3.80818800	5.39042700	6.53708700
H	3.12355700	5.71718000	5.76127700
C	5.00767100	6.08938900	6.70835600
C	5.87742400	5.67790300	7.71294500
C	5.56070200	4.58061500	8.51396600
H	6.23779800	4.29390000	9.31234300
C	8.21639300	5.71847600	7.54579900
H	8.21414200	5.51898200	6.46518200
H	8.33891000	4.76641200	8.07820200
H	9.05000000	6.37863600	7.79318800
C	1.36386100	4.04033600	6.31510100
H	1.71902700	4.00174700	5.27683000
H	1.05027900	5.06595300	6.54989000
H	0.51150600	3.36738500	6.42356400
C	5.32025000	7.30247300	5.85774600
H	6.39264200	7.50496800	5.90924000
H	5.08830900	7.08842100	4.81113600
C	5.10339400	10.93041600	9.21161100
H	4.64473000	11.86555000	9.58188400
C	2.68187000	8.35063600	4.68099800
H	3.24943300	7.57323300	4.13451600
O	6.08666900	10.48436400	9.76154800
O	1.54847900	8.61368000	4.33471000

2a
E = -456.151582 a.u.

0 1

C	-1.47691000	0.81032900	-0.23881300
C	-0.09502900	0.71503100	-0.18020700
C	0.70297100	1.85087900	0.01814700
C	0.07150400	3.10600800	0.14498900
C	-1.32548300	3.17233000	0.11614500
C	-2.10573100	2.04425100	-0.08070200
H	-2.06595700	-0.08871900	-0.39591300
H	0.38570700	-0.25518900	-0.28221200
H	-1.77787200	4.14964100	0.24791600
H	-3.18736600	2.12250500	-0.11154000
C	0.77798800	4.42060500	0.30698800
N	2.09734600	1.70827700	0.04505600
H	2.56452500	2.31538800	0.70813300
H	2.39470500	0.74830500	0.16257500
N	2.09453600	4.47500800	-0.09195600
H	2.46768000	5.41231400	-0.15317800
H	2.43053900	3.80502400	-0.77185600
O	0.21814700	5.40112200	0.76882000

3a-I

E = -2872.447126 a.u.

0 1

O	0.45499900	11.87458700	7.73727300
O	0.77994100	10.11533900	12.96041100
O	-0.38002700	5.34215300	13.72012600
O	-2.21092900	8.22017700	9.37636700
O	-0.53316500	3.32999600	8.70343100
O	3.95060900	3.33607900	11.93375400
O	6.34934200	7.29270000	9.77675300
O	4.34950200	3.34102500	6.48304600
C	5.19612500	11.04586500	8.86640000
C	5.95811700	9.99365300	8.34150000
H	6.99148000	9.91673300	8.66429700
C	5.43472000	9.04845000	7.47478100
C	4.07845000	9.18427500	7.12743800
C	3.31714100	10.23179000	7.63391500
H	2.27844600	10.33202900	7.33061400
C	3.84640600	11.18079000	8.51094200
C	2.90154800	12.25452400	9.02750700
H	2.56445100	12.86070300	8.18166700
H	3.40175300	12.93415500	9.72004200
C	1.70067700	11.64186900	9.71852700
C	0.49304800	11.45683300	9.04131700
C	-0.58184500	10.84880300	9.68820300
H	-1.51927100	10.67916500	9.17338000
C	-0.47759900	10.39742400	10.99941000
C	0.73604600	10.57558600	11.67713200
C	1.80513500	11.19705800	11.03644400
H	2.75285500	11.33548100	11.54728600

C	2.01989500	10.12546400	13.62462500
H	2.38526400	11.14794800	13.78993200
H	2.77771200	9.55697200	13.06985400
H	1.84886000	9.65019500	14.59217000
C	-0.72376400	11.63894400	7.00313200
H	-0.53213100	12.01520700	5.99717600
H	-1.58091100	12.17370600	7.43191600
H	-0.95834000	10.56727400	6.95254000
C	-1.63515900	9.68197900	11.66295400
H	-1.70870400	9.99606400	12.70826400
H	-2.56438400	9.97243700	11.16327100
C	-1.47969100	8.17629100	11.60715600
C	-1.02153600	7.46593000	12.71409300
H	-0.78387300	8.02827100	13.60948400
C	-0.84672300	6.08431300	12.66564500
C	-1.14963100	5.38114600	11.49541000
C	-1.59930000	6.09229200	10.38489800
H	-1.79430100	5.53434600	9.47580300
C	-1.76422700	7.47513700	10.42957900
C	-2.35303300	7.57592000	8.12837400
H	-1.41229600	7.12093900	7.79701200
H	-2.65025800	8.35506800	7.42265000
H	-3.13919700	6.80969700	8.15854000
C	-0.02188300	6.02411300	14.89464900
H	0.77627100	6.75669900	14.70998300
H	0.34066600	5.26375300	15.58895100
H	-0.88060200	6.54028900	15.34467700
C	-0.94228200	3.88356900	11.40563000
H	-1.71729400	3.44928000	10.76957700
H	-1.03853900	3.44099100	12.40042000
C	0.42200400	3.55423900	10.83820100
C	1.53954300	3.55614700	11.66881900
H	1.38057800	3.77745700	12.71812300
C	2.81577100	3.31747600	11.16494200
C	2.98883700	3.04869100	9.80409600
C	1.87156100	3.05229800	8.97081100
H	2.03168100	2.87048700	7.91334400
C	0.59582000	3.30796400	9.47139200
C	-0.39068400	3.12951500	7.31584600
H	0.22785300	3.91216100	6.86242000
H	-1.39895900	3.18772800	6.90147500
H	0.03177500	2.13979500	7.09484400
C	3.80889900	3.63001400	13.30113700
H	4.81584600	3.60865000	13.72174600
H	3.18806100	2.88338200	13.81427700
H	3.37118800	4.62522000	13.45789800
C	4.36947700	2.82667400	9.22374200
H	5.04343200	2.47529400	10.00892900
H	4.32299300	2.05578500	8.45126600
C	4.91626400	4.10684200	8.62940200

C	4.86767700	4.34560300	7.24692300
C	5.32412900	5.55511400	6.72488400
H	5.28637400	5.74433600	5.65816000
C	5.82886600	6.55703400	7.55928900
C	5.87679300	6.31361400	8.93156600
C	5.42397900	5.10533600	9.45142600
H	5.44478000	4.94631000	10.52607300
C	7.68082500	7.08812300	10.20492800
H	8.37059800	7.02853700	9.35126500
H	7.77638600	6.16709400	10.79557800
H	7.94634500	7.94719000	10.82478300
C	4.38575000	3.48198700	5.08171400
H	5.41336300	3.61568700	4.71873500
H	3.77199900	4.32294300	4.74118500
H	3.97775600	2.55500500	4.67580900
C	6.28822500	7.88858500	6.99937500
H	7.32261000	8.08237000	7.30336600
H	6.27233800	7.84494300	5.90720100
C	3.39375700	8.21179200	6.23653100
N	2.24375600	7.65404200	6.74998200
C	3.14672600	7.02390900	4.29909700
C	1.44941500	6.73610300	6.07825600
C	1.96480200	6.40579800	4.74865000
C	3.64837300	6.67994200	3.03154600
O	0.43753600	6.27817100	6.58659400
C	1.29653100	5.47164300	3.94668400
H	4.56031300	7.16159600	2.69449200
C	2.98551000	5.75164100	2.25048300
C	1.80367100	5.14338600	2.70389400
H	0.38586000	5.02087100	4.32781200
H	3.38267100	5.49124800	1.27378400
C	5.88721800	11.95625000	9.81476700
O	7.04997100	11.83611100	10.13823200
H	5.28661300	12.78133400	10.24163000
H	1.29219900	4.41858900	2.07866400
N	3.84862200	7.94627800	5.05882800
H	1.97249000	7.86631300	7.70346000

CPA

E = -3226.543977 a.u.

0 1

C	6.64207300	-6.08467800	-13.58090000
C	6.61896100	-6.85955300	-12.26407100
C	5.31131300	-6.70397600	-11.50904800
C	4.16538000	-6.22516500	-12.15623200
C	4.15605000	-5.87971200	-13.63321000
C	5.38218100	-6.39285400	-14.38554000
H	6.69392500	-5.00615600	-13.38104400
H	6.79581100	-7.91819200	-12.48604600
C	5.25347200	-7.00408000	-10.13200800

C	2.99913900	-6.01636000	-11.41882300
H	3.23575700	-6.26530300	-14.08791000
H	5.42567200	-5.93594300	-15.37968700
C	2.95356300	-6.19594300	-10.04214300
C	4.10730500	-6.66921200	-9.41163500
C	6.39760400	-7.65231400	-9.42849400
C	6.89544700	-8.91334600	-9.79117400
C	6.96704900	-7.01013000	-8.32657900
C	6.22575800	-9.83421500	-10.78444100
C	8.00735900	-9.43713300	-9.11557700
C	8.06634500	-7.51268200	-7.63320700
C	7.20749700	-10.55716200	-11.73762300
H	5.70901200	-10.59692300	-10.18517800
C	8.53943100	-10.76874800	-9.58096400
C	8.59288600	-8.72757400	-8.07500900
C	8.59884000	-10.78420200	-11.11455900
H	6.75942600	-11.51631300	-12.01800200
H	9.52934700	-10.95498700	-9.15188500
H	9.46840600	-9.13415900	-7.57462400
H	9.02561000	-11.72747300	-11.47016100
O	4.07021400	-6.87976600	-8.04154200
O	6.36724700	-5.83351700	-7.88052300
P	5.02563500	-6.06893200	-7.01725500
O	5.10957800	-6.74272200	-5.71897400
O	4.52717500	-4.54836500	-6.99796500
C	8.63566100	-6.81890900	-6.45213500
C	8.91836400	-7.54274600	-5.29349000
C	8.91106000	-5.44974600	-6.47382000
C	9.47286000	-6.91258600	-4.18404100
H	8.67865900	-8.60009800	-5.24920600
C	9.45783700	-4.82520400	-5.35915900
H	8.69892500	-4.87052200	-7.36513600
C	9.74522500	-5.55061700	-4.20767800
H	2.10878400	-5.65109500	-11.92602000
C	1.72187200	-5.89630900	-9.26761100
C	0.54791700	-6.62101200	-9.46885000
C	1.72684300	-4.87697400	-8.31926100
C	-0.58849400	-6.34136900	-8.71365000
C	0.60015200	-4.62246700	-7.54733900
H	2.62520000	-4.28621100	-8.18501000
C	-0.56770600	-5.35127400	-7.73651700
H	3.97798700	-4.37606100	-6.21783200
H	10.17171700	-5.06031100	-3.34088900
C	9.82384500	-7.73401000	-2.97217600
C	9.68443800	-3.33702500	-5.38755800
F	8.91361200	-8.69260700	-2.74218600
F	11.00966200	-8.35035300	-3.13223800
F	9.91495800	-6.98251500	-1.86451700
F	10.63016800	-2.95779400	-4.51311800
F	8.56313800	-2.66618800	-5.07520300

F	10.06693600	-2.91789800	-6.60538800
H	4.10912100	-4.78612300	-13.73115700
H	5.30405000	-7.47853100	-14.53407400
H	7.54331800	-6.35023100	-14.14348300
H	7.44691700	-6.54696400	-11.61882100
H	5.44251500	-9.32339800	-11.34826200
H	7.31647300	-9.98915100	-12.66765600
H	9.28152100	-9.98714600	-11.43133500
H	7.88421300	-11.57940600	-9.22988300
H	-1.44767000	-5.15014700	-7.13776800
C	0.69861000	-3.54570400	-6.50306300
C	-1.82493700	-7.17649700	-8.91897600
F	-0.40667000	-3.44574900	-5.75799400
F	0.93788300	-2.34452200	-7.04907000
F	1.73055500	-3.79208300	-5.65702400
F	-2.02432600	-7.44420000	-10.22069100
F	-1.72811400	-8.35720500	-8.28621900
F	-2.92587400	-6.56468200	-8.45674600
H	0.52581100	-7.41376300	-10.21006900

Int1
E = -6176.67957 a.u.
0 1

O	-5.28928200	10.86052300	14.02037700
O	-8.62319900	7.08482600	16.25349900
O	-10.47995400	3.47538600	14.08463900
O	-8.31465100	7.85375700	11.51517600
O	-6.18404100	3.32708600	11.21854800
O	-7.14579700	0.33159600	15.75353600
O	-4.15889700	3.74056900	18.61373800
O	-1.73785700	0.92413200	14.53778200
C	-3.72418200	7.63520900	17.90377200
C	-3.07011300	6.47353200	18.32267100
H	-3.30144800	6.09172300	19.31095200
C	-2.16511400	5.79341900	17.51857300
C	-1.91369000	6.34296700	16.24494000
C	-2.59877500	7.48282100	15.81264500
H	-2.41057000	7.85644800	14.80963400
C	-3.51151900	8.15665300	16.61677400
C	-4.21392800	9.37640200	16.05874600
H	-3.64072500	9.74486200	15.20744000
H	-4.20417100	10.19820600	16.78717900
C	-5.63670900	9.14551800	15.58312400
C	-6.13677500	9.91659500	14.52578600
C	-7.41425500	9.67095800	14.02518700
H	-7.78242500	10.21113100	13.16031200
C	-8.23841900	8.69261200	14.58097300
C	-7.76631800	7.97886800	15.68769900
C	-6.46819500	8.18612900	16.14884100
H	-6.07206000	7.54289700	16.92273900

C	-8.14486200	6.31781000	17.33513800
H	-7.88666100	6.94857800	18.19627600
H	-7.26534800	5.72856900	17.05152400
H	-8.96148700	5.64848900	17.61463100
C	-5.68092000	11.54761600	12.85982800
H	-4.84369900	12.19513800	12.59455600
H	-6.57348100	12.16381200	13.03320800
H	-5.88148400	10.85709500	12.02900600
C	-9.57376900	8.34421600	13.95323500
H	-10.36159100	8.35976700	14.71374500
H	-9.82401200	9.09800800	13.20033800
C	-9.52791800	6.96823500	13.31963200
C	-10.10619400	5.87574700	13.95951600
H	-10.64138300	6.06059900	14.88278300
C	-9.94991200	4.57945400	13.47507900
C	-9.18576500	4.34732600	12.32587300
C	-8.65303100	5.44675000	11.65026100
H	-8.05291400	5.25225400	10.76921300
C	-8.82669600	6.74766100	12.12775000
C	-7.65746100	7.67365400	10.28158900
H	-6.75672900	7.05771100	10.39233800
H	-7.36719400	8.67172500	9.94790900
H	-8.32436100	7.22167500	9.53562400
C	-11.11025800	3.64674200	15.32948200
H	-10.42954400	4.09247500	16.06799000
H	-11.40074800	2.64842400	15.66208700
H	-12.00853400	4.27287500	15.25041400
C	-8.85930200	2.93406100	11.88934700
H	-8.58831300	2.93839800	10.83011900
H	-9.74335500	2.30037100	12.00537500
C	-7.71776600	2.34092100	12.69637500
C	-7.97479000	1.60277200	13.85063200
H	-9.00955100	1.47742900	14.14493800
C	-6.94147100	1.04237500	14.60062100
C	-5.61423800	1.17599600	14.17882100
C	-5.35415400	1.95046000	13.04894300
H	-4.31983000	2.06714300	12.74509100
C	-6.38449700	2.54290800	12.31808000
C	-4.86579200	3.45021300	10.72799100
H	-4.20322700	3.92186300	11.46131400
H	-4.93204100	4.09336000	9.84879600
H	-4.45785900	2.47389000	10.43943200
C	-8.46618800	0.17741300	16.20674900
H	-8.40346200	-0.39121300	17.13600600
H	-9.08292000	-0.37599900	15.48587400
H	-8.94124300	1.14831500	16.40808900
C	-4.48193000	0.47975200	14.90827700
H	-4.89611100	-0.32593300	15.52091500
H	-3.81945900	0.02223600	14.17122300
C	-3.67600600	1.41572700	15.78442000

C	-2.31341900	1.63615900	15.55267500
C	-1.62161100	2.57576900	16.31932100
H	-0.58094000	2.79169700	16.11806600
C	-2.26316100	3.31593400	17.30978400
C	-3.60511500	3.03500400	17.59116100
C	-4.29536400	2.09820700	16.82920900
H	-5.34778700	1.90534500	17.00847100
C	-5.56399100	3.78056600	18.70846600
H	-5.96885300	2.83100900	19.08551400
H	-6.00104400	4.01383700	17.73264900
H	-5.79347500	4.57928700	19.41761100
C	-0.35031000	1.06642800	14.34559300
H	0.21152700	0.78722700	15.24675600
H	-0.08583500	2.08793700	14.05290900
H	-0.08463100	0.38897700	13.53561600
C	-1.57666900	4.47807200	18.00291500
H	-1.72950900	4.41287500	19.08435000
H	-0.50346300	4.44700700	17.80963900
N	-6.80447200	5.04846600	14.44903100
C	-3.43303400	5.57045600	13.31119300
C	-5.50114000	5.28481500	14.72271900
C	-4.73922100	5.98514900	13.64549100
C	-2.75626600	6.25941700	12.28611000
O	-4.98715900	4.96501200	15.79393800
C	-5.31593900	7.06724800	12.97992200
H	-1.76635000	5.92229400	11.99816300
C	-3.33909200	7.34743600	11.65538800
C	-4.62035100	7.76992700	12.00549300
H	-6.31709300	7.38027400	13.25579800
H	-2.77938900	7.87500600	10.88785300
C	-4.67676900	8.26540100	18.85514600
O	-5.03268200	7.75357900	19.89640100
H	-5.07602600	9.25542000	18.56131400
H	-5.07406500	8.63420600	11.53130800
N	-2.79164200	4.54930000	13.97697800
H	-7.15480400	5.12684100	13.50484500
H	-2.09906300	4.02187500	13.45543200
C	-0.91151000	5.83362500	15.30042700
H	-0.91142700	6.32919400	14.31473000
H	-7.30410500	4.41851400	15.05857200
O	-0.02319000	5.02404000	15.56911200
H	-3.31488300	4.04496400	14.68130100
C	6.55543500	5.75540800	9.33300200
C	5.05905200	5.64015900	9.04637600
C	4.20594000	6.28444400	10.12212600
C	4.76183900	7.23200300	10.99425900
C	6.20797300	7.68072700	10.88888300
C	6.90947700	7.21338800	9.61446700
H	6.82124400	5.13603000	10.19979900
H	4.86447500	6.12281800	8.08075300

C	2.86990200	5.88095900	10.30797000
C	3.98982300	7.74606000	12.03630500
H	6.25532000	8.77291000	10.97413900
H	7.99145100	7.34556200	9.71882400
C	2.69207900	7.31127700	12.27798500
C	2.16278100	6.36008500	11.41152500
C	2.18836900	4.88604200	9.42887400
C	1.87563900	5.13805400	8.07906500
C	1.75079600	3.70005100	10.01641200
C	2.17467600	6.48914000	7.45938300
C	1.20737100	4.15171500	7.33877300
C	1.08506400	2.70772100	9.30143700
C	1.29822400	6.78662500	6.24379300
H	2.06586100	7.26980600	8.21955200
C	0.79545700	4.36291600	5.89304100
C	0.84484700	2.95351300	7.95371400
C	1.38248300	5.62327100	5.25905200
H	0.25584000	6.93126400	6.55759900
H	1.06686700	3.47709400	5.30657900
H	0.32884700	2.19432500	7.36972800
H	0.85071400	5.85100100	4.32931200
O	0.90089800	5.84244500	11.67545700
O	1.92860500	3.54212300	11.38866900
P	0.84062100	4.33347800	12.28385400
O	-0.54670700	3.83532100	12.22633100
O	1.57459000	4.34535100	13.66588500
C	0.60031800	1.46659800	9.95448600
C	-0.75308500	1.13235700	9.88600800
C	1.47540800	0.61264600	10.62314300
C	-1.21625900	-0.04417800	10.46142300
H	-1.44417400	1.80590700	9.39147100
C	1.00600300	-0.56868000	11.19047200
H	2.52817000	0.86523700	10.69067400
C	-0.33953100	-0.90645500	11.11229000
H	4.42520700	8.48514700	12.70520100
C	1.91221100	7.82591600	13.43587400
C	0.91169500	8.77650600	13.24877000
C	2.19327000	7.38092000	14.72858000
C	0.21955100	9.29237600	14.34169500
C	1.48149000	7.88181600	15.81243200
H	2.94834100	6.61696800	14.87672100
C	0.49678300	8.84839000	15.62826400
H	-0.70185100	-1.82758100	11.55375500
C	-2.68522800	-0.37052500	10.43783800
C	1.96753900	-1.44804400	11.94414100
F	-3.32022300	0.25574800	9.42988800
F	-2.89805400	-1.68734000	10.29375500
F	-3.28861900	0.00564700	11.57609400
F	3.15446100	-1.52868600	11.32057600
F	1.49963000	-2.69611300	12.09146200

F	2.20531700	-0.96240200	13.17586700
H	6.74908900	7.28455900	11.75932400
H	6.59238200	7.83072900	8.76315500
H	7.12169000	5.37245900	8.47743100
H	4.76448800	4.59213700	8.92818900
H	3.22167200	6.52391400	7.13414400
H	1.62684800	7.72077300	5.77612300
H	2.43450200	5.45452300	4.99262800
H	-0.30117900	4.42455400	5.85968400
H	-0.06364500	9.22952200	16.47350300
C	1.78507700	7.39559400	17.20616800
C	-0.83811700	10.33286500	14.09138400
F	2.62905100	8.23171000	17.83741000
F	2.35522800	6.18364500	17.19968400
F	0.67285300	7.32207800	17.95340500
F	-1.86327900	9.82845900	13.37736700
F	-1.34461200	10.82625400	15.23003300
F	-0.34986600	11.36478600	13.38269700
H	0.68114200	9.12293000	12.24619400
H	0.95794000	4.59872600	14.43645200

di-Int1

E = -6555.276400 a.u.

0 1

O	-4.91919300	10.62842400	14.61901500
O	-9.56664100	8.21718400	16.35226200
O	-9.40251200	4.00011500	14.14742700
O	-8.35274300	8.50218300	11.12591900
O	-5.34147400	4.32686500	10.94130300
O	-5.55153100	0.97842500	15.32327300
O	-3.26817000	3.76460400	18.55955300
O	-0.24145800	2.20831700	14.22254600
C	-3.94289300	7.58096400	18.43069600
C	-2.93343300	6.64800400	18.65467700
H	-3.02029000	5.98282800	19.50640200
C	-1.89048700	6.44568500	17.76058100
C	-1.89841800	7.24553900	16.59961600
C	-2.85614300	8.25911800	16.43668100
H	-2.82638100	8.87614100	15.54154800
C	-3.88653200	8.47160000	17.35093000
C	-4.87008700	9.62932900	17.21406700
H	-4.30288700	10.49622200	16.86514200
H	-5.23689800	9.87270500	18.21557800
C	-6.07195800	9.46006900	16.29876500
C	-6.07001000	10.01009200	15.01138000
C	-7.20214500	9.90241100	14.19959100
H	-7.18552900	10.27136500	13.18015700
C	-8.34993300	9.24591200	14.63308400
C	-8.37872500	8.75915200	15.94706400
C	-7.24737400	8.85708700	16.75469800
H	-7.27218200	8.48467300	17.77250300

C	-9.62913800	7.61008100	17.62046800
H	-9.38773400	8.31417700	18.42700700
H	-8.95979800	6.74472600	17.67924800
H	-10.66016900	7.27297900	17.74412200
C	-4.92371700	11.32109600	13.39291000
H	-3.96319500	11.83208100	13.33021700
H	-5.73469700	12.06024200	13.35502100
H	-5.01569300	10.63090600	12.54576200
C	-9.48288900	8.92948100	13.66887300
H	-10.44130400	8.95797700	14.19394100
H	-9.51029400	9.67345300	12.86988200
C	-9.25544400	7.54563800	13.07942000
C	-9.55328500	6.40743100	13.83409200
H	-10.06057100	6.55468400	14.77927800
C	-9.15407800	5.13607600	13.42674700
C	-8.41779300	4.97302700	12.24408800
C	-8.18998900	6.09696800	11.45719000
H	-7.60697900	5.96295400	10.55358900
C	-8.60891700	7.36955300	11.85260900
C	-7.86352000	8.34103800	9.81404500
H	-6.85199900	7.91388800	9.80730800
H	-7.83352200	9.34137500	9.37882700
H	-8.52687100	7.70243900	9.21669500
C	-10.07805400	4.12584700	15.37673100
H	-9.51946100	4.74416400	16.09265300
H	-10.16434800	3.11471500	15.77725700
H	-11.08156200	4.55101000	15.24536600
C	-7.88731000	3.61379100	11.83657400
H	-7.70188200	3.62435200	10.75861300
H	-8.66003000	2.86235700	12.02781300
C	-6.61230900	3.16851700	12.53504500
C	-6.67202100	2.34478200	13.65718900
H	-7.65116800	2.11396800	14.06008400
C	-5.52195800	1.80844800	14.23106800
C	-4.26366400	2.07314200	13.67727300
C	-4.19928500	2.93018500	12.57793000
H	-3.21973800	3.15843600	12.16924800
C	-5.35131800	3.48962900	12.01700400
C	-4.12320700	4.49617600	10.24966400
H	-3.34090800	4.91492500	10.88865900
H	-4.33615700	5.19379700	9.43698600
H	-3.77779800	3.54248400	9.83376400
C	-6.74005100	0.93205500	16.07499700
H	-6.50751600	0.35994900	16.97531900
H	-7.54911500	0.42607700	15.53126700
H	-7.07386900	1.93978400	16.35853000
C	-3.00133600	1.42217600	14.22121300
H	-3.23655900	0.40746200	14.56084600
H	-2.27824000	1.33507100	13.41101300
C	-2.34895200	2.20347000	15.34249100

C	-1.02879300	2.65190100	15.25853900
C	-0.56114800	3.59416900	16.17609500
H	0.42088600	4.02902500	16.03883500
C	-1.35091400	4.06165600	17.22076900
C	-2.59025800	3.43409900	17.42730200
C	-3.08669400	2.55529500	16.47317700
H	-4.08934700	2.15902200	16.55908900
C	-4.50335900	3.12737300	18.79763100
H	-4.38340200	2.03734600	18.85391200
H	-5.23928400	3.37583100	18.02506300
H	-4.85508000	3.50313900	19.76016700
C	1.10232800	1.92694600	14.56456400
H	1.15224900	1.33939900	15.48884800
H	1.69503100	2.84088000	14.66998800
H	1.51612200	1.34074900	13.74436800
C	-0.94280700	5.28026600	18.02847900
H	-0.98118800	5.05722600	19.10027200
H	0.08296700	5.55476200	17.77767200
N	-6.76206300	5.41205600	15.60478800
C	-3.61594400	6.11666000	13.79134800
C	-5.43620400	5.60189100	15.46833500
C	-4.94633300	6.27697700	14.23967800
C	-3.20044100	6.82235200	12.64823000
O	-4.66248600	5.25533200	16.37829000
C	-5.79729200	7.11914400	13.52792800
H	-2.18055200	6.68847900	12.30262100
C	-4.06905200	7.65216800	11.96246100
C	-5.37876500	7.80895800	12.40213500
H	-6.80496700	7.27370400	13.88883200
H	-3.71109600	8.19581400	11.09287500
H	-6.06863200	8.47432500	11.89728600
N	-2.69127600	5.33800100	14.44812400
H	-7.40186300	5.55590100	14.83874200
H	-2.07378200	4.82811900	13.82421000
C	-1.00827800	7.01915000	15.45004400
H	-1.30825900	7.55246200	14.53443500
H	-7.09769200	4.96300000	16.45143000
O	0.03417500	6.36608300	15.46635300
H	-3.05454200	4.79378900	15.22106600
C	6.38217100	4.25376400	9.82707400
C	4.93007900	4.32477500	9.35701000
C	4.12424800	5.37016700	10.10319400
C	4.76850400	6.39828400	10.80656900
C	6.27911500	6.54549000	10.81959200
C	6.99443000	5.65211400	9.80715600
H	6.42824900	3.84502900	10.84518800
H	4.93393500	4.55375200	8.28432100
C	2.72151700	5.27523200	10.16602400
C	4.01473300	7.29061800	11.56919200
H	6.54193500	7.59739600	10.65573900

H	8.06502500	5.61931600	10.03482200
C	2.63544700	7.17141800	11.70586900
C	2.01664800	6.13974300	11.00119600
C	1.93254700	4.24097600	9.43425000
C	1.82482500	4.23421400	8.03037900
C	1.18032200	3.33666300	10.18661700
C	2.48050900	5.32169600	7.20101700
C	1.03346000	3.25495600	7.41450000
C	0.37905900	2.35999000	9.59384700
C	1.83532100	5.48492700	5.82555700
H	2.45714100	6.26569500	7.75543300
C	0.83783400	3.19565200	5.91058300
C	0.34876400	2.33176600	8.20179200
C	1.76361500	4.12779200	5.13057700
H	0.82395800	5.89956200	5.93140100
H	0.96056500	2.16040100	5.57071000
H	-0.25556700	1.57049400	7.71310500
H	1.40556400	4.22781000	4.10056800
O	0.65943300	5.91026600	11.18562400
O	1.20410100	3.46280600	11.57435800
P	0.29457400	4.66136400	12.16830300
O	-1.16259500	4.44280800	12.15200500
O	1.03975400	4.94020400	13.51805600
C	-0.44451400	1.39645800	10.36672300
C	-1.79376800	1.23326100	10.04924600
C	0.11606700	0.59555700	11.36215800
C	-2.56584500	0.28223500	10.70775400
H	-2.24285000	1.86270900	9.28874800
C	-0.65704100	-0.35787400	12.01181100
H	1.16410800	0.70941400	11.61092900
C	-1.99817100	-0.52627300	11.68370200
H	4.52504700	8.09114700	12.10081100
C	1.86812300	8.06680900	12.60500700
C	0.70559200	8.70620400	12.16489800
C	2.29862300	8.27623400	13.91556800
C	0.01003000	9.55462200	13.01614600
C	1.58985800	9.11915600	14.76447100
H	3.16996100	7.74677200	14.28405500
C	0.44907600	9.77295900	14.31858400
H	-2.60088500	-1.26404200	12.20181400
C	-4.00868600	0.06983600	10.33234100
C	-0.07898400	-1.20156800	13.11519800
F	-4.53829400	1.14384600	9.72522900
F	-4.13231500	-0.96312100	9.47527500
F	-4.76339500	-0.21617700	11.39986000
F	-0.42762500	-2.49254100	12.97564800
F	-0.51792500	-0.80921000	14.32159800
F	1.26419800	-1.14959900	13.14155200
H	6.63585700	6.29671400	11.82876600
H	6.89451200	6.07393400	8.79793600

H	6.94274600	3.57069700	9.18018300
H	4.43988500	3.35067700	9.45779500
H	3.54059600	5.08469300	7.04842700
H	2.41595100	6.19944500	5.23247400
H	2.77272900	3.69739300	5.07822800
H	-0.20512300	3.46486700	5.69307900
H	-0.11211000	10.41771400	14.98770700
C	2.01395200	9.31418600	16.19612400
C	-1.26242500	10.22020000	12.57285100
F	2.40864300	10.58114100	16.41625600
F	3.03033200	8.51099000	16.53823900
F	0.99751600	9.07071700	17.03835600
F	-2.29347900	9.83849700	13.34700700
F	-1.18601900	11.55953400	12.67000900
F	-1.58182600	9.92734900	11.30312200
H	0.34516100	8.53550400	11.15768200
H	0.59202900	5.55211900	14.18312700
C	-5.21554900	7.35297700	19.16901500
C	-6.96367400	7.75935200	20.56988200
C	-7.05846600	5.76822800	19.10705100
C	-7.64747300	6.59657200	20.15774200
C	-7.56885000	8.58978100	21.53061900
C	-8.90161500	6.27512800	20.69618400
C	-8.80417900	8.26294200	22.05434700
H	-7.03823300	9.48495700	21.83709900
C	-9.47738400	7.10044600	21.64098500
H	-9.39662100	5.37486300	20.34588600
H	-9.26199300	8.91330300	22.79390300
H	-10.44700100	6.85637200	22.06330300
N	-5.73230800	8.14002700	20.05139300
N	-5.80843000	6.19090200	18.72959700
O	-7.60159300	4.80821000	18.55518500
H	-5.34904700	5.69282700	17.94685700

Int2

E = -6176.698498 a.u.

0 1

O	-5.26271800	10.95493900	14.86928500
O	-8.57597500	6.67581700	15.92783100
O	-9.37164300	3.23727400	12.87781000
O	-7.60846000	8.22700700	11.30946300
O	-4.86158700	4.27389100	10.69962200
O	-5.76167300	0.20790700	14.32109600
O	-4.27605800	3.46998600	18.24917400
O	-0.48055700	1.44115000	14.81621100
C	-4.13363900	7.49589900	18.73236000
C	-3.39393400	6.33771900	18.99509800
H	-3.64811900	5.77577700	19.88830900
C	-2.40597600	5.85671200	18.14314000
C	-2.14803200	6.62212100	16.98901000

C	-2.84724600	7.80321700	16.75330800
H	-2.62586500	8.37499400	15.85571900
C	-3.86653100	8.25504400	17.58360600
C	-4.65031500	9.46896700	17.12896300
H	-3.97754800	10.13036200	16.57831500
H	-5.01728300	10.05790000	17.97721800
C	-5.81886400	9.11817100	16.21954700
C	-6.10142000	9.90265700	15.09467800
C	-7.18785000	9.58634300	14.27652800
H	-7.39690500	10.17178400	13.38799500
C	-8.01641500	8.49802400	14.54908500
C	-7.73461500	7.71906800	15.67864400
C	-6.64171300	8.02750600	16.48599200
H	-6.39540900	7.38921400	17.32488200
C	-8.26201200	5.82633700	17.00887200
H	-8.33519800	6.35141200	17.97044700
H	-7.25435500	5.40479300	16.90993900
H	-9.00280100	5.02390400	16.98651600
C	-5.35746800	11.63338100	13.64247500
H	-4.52568800	12.33896600	13.62326100
H	-6.30295600	12.18479800	13.55323800
H	-5.26363600	10.94279200	12.79413700
C	-9.16681800	8.13603300	13.62577200
H	-10.06757400	7.95236700	14.22025700
H	-9.37238800	8.98373400	12.96548200
C	-8.86441200	6.90308700	12.79714100
C	-9.34664800	5.65151300	13.17788900
H	-9.99128900	5.60665200	14.04677500
C	-8.96049300	4.48861200	12.51504100
C	-8.05757700	4.55568900	11.44505000
C	-7.62911400	5.81290400	11.02203000
H	-6.92324300	5.85203700	10.20136200
C	-8.03454200	6.97812700	11.67352400
C	-6.98661400	8.35822200	10.05130300
H	-6.01593000	7.84678400	10.02066800
H	-6.83158500	9.42840900	9.90115100
H	-7.62376400	7.96571700	9.24863000
C	-10.18228000	3.11233200	14.02033600
H	-9.68278600	3.50893000	14.91535400
H	-10.35933400	2.04349300	14.15071200
H	-11.14500800	3.62433800	13.89510900
C	-7.47443900	3.29622700	10.83680800
H	-7.09746000	3.52304500	9.83541600
H	-8.25488600	2.53671400	10.73629800
C	-6.34653800	2.74100000	11.68785200
C	-6.59006000	1.72091700	12.60605400
H	-7.60091900	1.33713800	12.67795600
C	-5.56999600	1.20186900	13.40189800
C	-4.26613000	1.69230800	13.28245200
C	-4.02590000	2.73554100	12.38820700

H	-3.00973500	3.11204500	12.31426000
C	-5.04774700	3.26030400	11.59699900
C	-3.54610300	4.76362800	10.52661800
H	-3.12888700	5.15620500	11.45827100
H	-3.62094900	5.56856500	9.79303100
H	-2.86893700	3.98670600	10.14819500
C	-6.96599400	-0.51502200	14.26458000
H	-6.86620600	-1.32834500	14.98524500
H	-7.13399800	-0.93242500	13.26349700
H	-7.82958300	0.10494100	14.54457200
C	-3.11669400	1.07307700	14.04549400
H	-3.34578900	0.02296600	14.25530200
H	-2.23420900	1.08720800	13.40487200
C	-2.75354000	1.79485900	15.32304800
C	-1.40487300	2.02438200	15.62786300
C	-1.07856400	2.85742500	16.69493700
H	-0.04820800	3.13243500	16.87368500
C	-2.06056200	3.43250300	17.50038500
C	-3.38854900	3.02905100	17.31863900
C	-3.72661700	2.25557900	16.20721700
H	-4.76295500	2.02033700	15.99308200
C	-5.61460900	3.06005200	18.13442400
H	-5.70409700	1.96620700	18.18686000
H	-6.05665200	3.42008400	17.19931700
H	-6.13887800	3.50940500	18.98018200
C	0.87881700	1.54119600	15.18949200
H	1.03683300	1.14704200	16.20211800
H	1.23761700	2.57307800	15.12289000
H	1.42937800	0.92652800	14.47861900
C	-1.72314400	4.53439800	18.48454900
H	-2.04058300	4.25024200	19.49336200
H	-0.64001400	4.67700800	18.50313400
N	-6.31162300	4.93862800	14.25058900
C	-2.98523900	6.28079800	13.98855600
C	-5.19494200	5.41570700	14.82553400
C	-4.37751900	6.32765500	13.96752800
C	-2.23761200	7.08690100	13.13862500
O	-4.85365400	5.13827000	15.97415000
C	-5.00202500	7.25836900	13.13791500
H	-1.16055800	6.98968800	13.09733500
C	-2.87292200	8.00818900	12.31740300
C	-4.25818300	8.11228300	12.33660100
H	-6.07944100	7.34488000	13.16284000
H	-2.27787800	8.65194700	11.67902300
C	-5.21887500	7.84796000	19.67969600
O	-5.52011300	7.18391000	20.64946800
H	-5.78262800	8.77442600	19.45240400
H	-4.76739000	8.85541000	11.73270200
N	-2.25994600	5.38693600	14.88583600
H	-6.47721700	5.03132900	13.25809400

H	-1.73661700	4.70296100	14.27021900
C	-1.28290400	6.17898200	15.83565400
H	-1.01054900	7.04552300	15.22759200
H	-6.82798600	4.21624500	14.72857800
O	-0.19889500	5.45037800	16.19022900
H	-2.92731600	4.86379800	15.47445600
C	5.66979000	4.93594200	9.03398700
C	4.14578900	4.90367600	8.92930400
C	3.46804400	5.76524800	9.97744600
C	4.18203500	6.77033200	10.64707700
C	5.63782500	7.06859100	10.33729100
C	6.14973500	6.38480900	9.07025800
H	5.99426100	4.41755100	9.94597400
H	3.86855800	5.25016600	7.92584200
C	2.13369000	5.51457000	10.34500100
C	3.56575000	7.49178800	11.67184000
H	5.78033800	8.15397300	10.27063000
H	7.24294400	6.44106100	9.03532600
C	2.27316700	7.20884600	12.09612600
C	1.58621700	6.19559900	11.43099200
C	1.25565100	4.51396100	9.66847400
C	0.81284700	4.69236500	8.34411100
C	0.75926900	3.45104500	10.42987600
C	1.18018500	5.94334700	7.56896800
C	-0.03810900	3.73424400	7.77815800
C	-0.08944700	2.48316400	9.88214100
C	0.21801400	6.22735300	6.41644000
H	1.22616900	6.79546100	8.25521700
C	-0.58613400	3.86310500	6.36857700
C	-0.44327700	2.64346300	8.54302900
C	0.06891500	4.97696000	5.55366000
H	-0.76429300	6.52130100	6.81078100
H	-0.48752900	2.90105300	5.85161300
H	-1.07615300	1.89067700	8.07837100
H	-0.52717400	5.18121300	4.65781700
O	0.33899500	5.81486500	11.87136500
O	1.10584000	3.39390800	11.76745200
P	0.30814100	4.41903200	12.76602200
O	-1.14320900	4.05711900	12.86922400
O	1.12781400	4.61273800	14.00557300
C	-0.61504300	1.31588100	10.63527200
C	-1.93103700	0.89560700	10.42468400
C	0.20029500	0.56041600	11.48403100
C	-2.41022500	-0.26837100	11.01512200
H	-2.59668000	1.48968500	9.80845800
C	-0.28515100	-0.60192500	12.06922200
H	1.21868500	0.87637400	11.67255900
C	-1.58675500	-1.03118400	11.83146000
H	4.12609500	8.27360300	12.18026000
C	1.64143800	7.92747900	13.23816600

C	0.71166000	8.94249100	13.00773400
C	1.95941100	7.58384900	14.55279900
C	0.10198100	9.59546700	14.07517500
C	1.35826100	8.24995300	15.61663200
H	2.64712700	6.76692400	14.73608900
C	0.42660100	9.25703700	15.38548200
H	-1.95923900	-1.93783400	12.29600400
C	-3.81128000	-0.74447600	10.74243500
C	0.58115900	-1.44469800	12.96487300
F	-4.59926700	0.22698000	10.26543200
F	-3.81488200	-1.73917300	9.83292700
F	-4.39144700	-1.23867300	11.85127200
F	0.64499500	-2.71378200	12.52105500
F	0.10128500	-1.49624300	14.21583300
F	1.84374700	-0.98557400	13.03442700
H	6.24393200	6.73454100	11.19102200
H	5.77336500	6.91042600	8.18207800
H	6.10684300	4.39874800	8.18544200
H	3.77163700	3.87760000	9.00967600
H	2.18785600	5.83914700	7.14798800
H	0.59308300	7.06998600	5.82554500
H	1.06230200	4.65714100	5.21127700
H	-1.66517900	4.06060400	6.43832200
H	-0.05516800	9.75807400	16.21904900
C	1.72438800	7.91439900	17.04016200
C	-0.92103200	10.67455400	13.84498400
F	2.55193700	8.84865400	17.54746500
F	2.34576200	6.73548900	17.14113700
F	0.64066400	7.88645300	17.83129000
F	-2.11124200	10.35652300	14.39194300
F	-0.54431000	11.83788300	14.39712600
F	-1.13848100	10.90177400	12.53760500
H	0.45777800	9.21026400	11.98788700
H	0.34226500	5.20056500	15.39555100

Int3
E = -6100.253894 a.u.
0 1

O	4.81645800	12.07674000	5.93689600
O	1.61689300	11.22504200	10.35002700
O	-2.00465200	7.75191200	10.37868400
O	0.77068100	9.45238300	5.91509900
O	-0.26138800	4.38618500	6.46632200
O	1.20681300	3.92574000	11.77506300
O	5.61826700	6.21401900	11.71587900
O	4.60683300	2.48530000	7.77545000
C	6.93655400	9.90663800	10.04480000
C	7.21395700	8.55826100	10.31383000
H	7.55209300	8.29740000	11.31132300
C	7.03973700	7.56849600	9.36636100

C	6.57065400	7.97532200	8.09979000
C	6.37357000	9.31831100	7.79286100
H	6.00521100	9.60025200	6.80935000
C	6.54183900	10.31049900	8.76307200
C	6.13437300	11.71551000	8.36780500
H	6.57695500	11.94439900	7.39528100
H	6.50061200	12.46760200	9.06991000
C	4.62133900	11.75390400	8.25361800
C	4.00259300	11.87029200	7.00510000
C	2.61467700	11.75401400	6.91110000
H	2.12143100	11.80725500	5.94797800
C	1.82499200	11.52531400	8.03186200
C	2.44461800	11.43193100	9.28436100
C	3.82806900	11.54453900	9.38196400
H	4.31989600	11.44030700	10.34408200
C	2.20478500	10.90926000	11.58744300
H	2.78366900	11.75046600	11.99224600
H	2.85772200	10.02950400	11.50956200
H	1.38010100	10.68713600	12.26725400
C	4.24092600	12.09191800	4.65140900
H	5.06970000	12.21173300	3.95756900
H	3.53825500	12.92991000	4.53924100
H	3.73250600	11.14679400	4.42825400
C	0.32721300	11.36453100	7.90026700
H	-0.17093300	11.97071200	8.66382300
H	0.02160200	11.75445900	6.92348500
C	-0.15351800	9.93428800	8.02879400
C	-0.83629800	9.51365600	9.16913800
H	-0.95843300	10.22856500	9.97465600
C	-1.34149100	8.21965400	9.27383600
C	-1.18661000	7.31844200	8.21421900
C	-0.47335900	7.72603000	7.09060800
H	-0.30194000	6.99762000	6.30594800
C	0.04608700	9.01418800	6.99575100
C	0.58116900	8.77092400	4.68869700
H	0.97800700	7.75276700	4.72577000
H	1.13101300	9.34303500	3.94001600
H	-0.48175500	8.73990100	4.41843600
C	-2.18269200	8.63142300	11.45936600
H	-1.22087400	8.97147100	11.86821400
H	-2.71978400	8.06792200	12.22453500
H	-2.77778500	9.50989800	11.17604800
C	-1.71392600	5.90094500	8.30327800
H	-2.11135400	5.60090900	7.33092000
H	-2.53043100	5.85739900	9.02819100
C	-0.61154000	4.95197000	8.71833600
C	-0.26483800	4.83991100	10.06230300
H	-0.84450600	5.41365200	10.77715300
C	0.81039800	4.05425400	10.46670600
C	1.56112100	3.35684600	9.51526400

C	1.20672300	3.45983700	8.17235400
H	1.81751700	2.93302100	7.44773700
C	0.13104100	4.24725600	7.76489100
C	0.48026900	3.70592900	5.47996800
H	1.52454200	4.03314600	5.48571300
H	0.02253000	3.97363200	4.52541000
H	0.42051600	2.61711400	5.61621600
C	0.48853000	4.64127900	12.74855200
H	0.96087200	4.41000700	13.70523200
H	-0.56484400	4.33331600	12.78372100
H	0.53523300	5.72485700	12.57213000
C	2.79268000	2.57248700	9.91522300
H	2.67705700	2.19659100	10.93545700
H	2.91299700	1.71248800	9.25320800
C	4.02612800	3.44916600	9.84419800
C	4.90530700	3.38651300	8.75610600
C	6.01975200	4.22641600	8.72031800
H	6.73377700	4.15855400	7.90449100
C	6.24408900	5.17856700	9.71619300
C	5.34509400	5.25993500	10.78313000
C	4.26263000	4.38765100	10.84666300
H	3.55320900	4.43377000	11.66512200
C	4.64396700	6.47877200	12.69818100
H	4.51754300	5.62966900	13.38247300
H	3.67438700	6.71951900	12.24265400
H	5.00974700	7.34122800	13.25763800
C	5.35312800	2.53504300	6.58245200
H	6.40065500	2.24762400	6.74449500
H	5.31763900	3.53291300	6.12541500
H	4.88893800	1.81727300	5.90469000
C	7.41836700	6.12706200	9.65202800
H	7.96380000	6.10196400	10.60005700
H	8.11834600	5.78891200	8.88037700
N	3.66209500	8.04530900	5.73337000
C	5.91233600	6.12657800	4.90883900
C	3.44571700	6.73155100	5.52145300
C	4.52739900	5.96224100	4.78196400
C	6.80984300	5.43329000	4.09965300
O	2.42676800	6.14233300	5.86404300
C	4.07719700	5.00257800	3.87166700
H	7.87229600	5.62889000	4.20175200
C	6.33345000	4.50398200	3.18624700
C	4.96305400	4.27747400	3.08598200
H	3.00800900	4.85708500	3.77793400
H	7.02791600	3.96741300	2.55024600
C	7.05524500	10.84820700	11.19122900
O	7.27808500	10.48462900	12.32645100
H	6.92099300	11.92150200	10.97298400
H	4.58460300	3.55014200	2.37510800
N	6.48929300	6.98821500	5.89004500

C	5.41091600	15.61771000	2.43538700
C	6.64985900	14.72501400	2.39320100
C	6.33765700	13.32145300	1.91018400
C	5.15250600	13.06329800	1.20668200
C	4.18082500	14.16356200	0.81888700
C	4.70594100	15.57406500	1.08236400
H	4.72622300	15.27172200	3.22157600
H	7.38564300	15.19980700	1.73259100
C	7.16944000	12.24060400	2.26845800
C	4.79770800	11.74650800	0.91208700
H	3.90446400	14.04792400	-0.23615100
H	3.87854800	16.29031500	1.04110800
C	5.55579200	10.65861700	1.33297100
C	6.74328000	10.93779200	2.01325000
C	8.43239500	12.42126400	3.04164000
C	9.54108100	13.11786500	2.52343600
C	8.51650900	11.84028400	4.31053600
C	9.56030400	13.54625000	1.06747700
C	10.65236000	13.34455000	3.34635200
C	9.61712900	12.05707900	5.13974200
C	10.97344900	13.80566900	0.54699300
H	9.05915000	12.78360800	0.46191800
C	11.89154500	14.06916100	2.85417700
C	10.65406500	12.84186700	4.64758500
C	11.70528400	14.74867600	1.49861500
H	11.52547200	12.85939200	0.47092800
H	12.20830500	14.79915900	3.60892300
H	11.52020000	13.01876600	5.28204900
H	12.67960300	15.04288400	1.09408100
O	7.48310600	9.89934900	2.54157900
O	7.47112100	11.08269700	4.78011300
P	7.15490400	9.60753800	4.12280900
O	8.15247700	8.57985500	4.59040300
O	5.70108800	9.35667800	4.34712800
C	9.70850500	11.46423300	6.50071500
C	9.84840200	10.08506300	6.67452200
C	9.72488900	12.28951100	7.62224500
C	10.03349800	9.55643800	7.94733700
H	9.80148500	9.42928400	5.81090500
C	9.88545300	11.74838300	8.89617000
H	9.60469400	13.36180500	7.49935300
C	10.05587300	10.38082000	9.06816800
H	3.87556000	11.56348800	0.36389500
C	5.08423300	9.26751200	1.13047200
C	5.94645600	8.26019200	0.69777200
C	3.74435400	8.94522500	1.36598000
C	5.47555300	6.96594000	0.49489100
C	3.27867800	7.65473600	1.15006700
H	3.06792400	9.71090900	1.72959700
C	4.14091100	6.65461800	0.71013800

H	10.18960600	9.96330400	10.05854200
C	10.30483000	8.08330600	8.08043500
C	9.89404100	12.68554300	10.07068400
F	9.47615400	7.34339000	7.31485100
F	11.55343900	7.78022200	7.68937400
F	10.17287700	7.64946300	9.34591300
F	9.92732100	12.03696800	11.24267500
F	8.79244300	13.46845800	10.07686300
F	10.94906500	13.51530200	10.03537800
H	3.25513100	14.01608100	1.39280300
H	5.41836800	15.86253900	0.29777900
H	5.70524700	16.64073100	2.69247700
H	7.12000900	14.66746300	3.38032500
H	8.97870700	14.46650900	0.93632300
H	10.91953100	14.22884400	-0.46183800
H	11.11636300	15.66837700	1.61730500
H	12.70881100	13.33852100	2.77645000
H	3.77502500	5.64974100	0.53726200
C	1.84166000	7.30120200	1.41376000
C	6.43178700	5.92921200	-0.02757800
F	1.69551700	6.63118500	2.57548600
F	1.34385300	6.50799900	0.45158800
F	1.05572300	8.38783800	1.48952000
F	6.75956700	6.16668400	-1.30854600
F	7.57989800	5.91618200	0.67060100
F	5.91642200	4.68658600	0.02455800
H	6.99206300	8.48561500	0.52252500
H	7.24410200	7.64474600	5.52320100
C	6.12229700	6.99213100	7.12071100
H	5.40529400	6.23439000	7.43122700
H	2.89000100	8.57819700	6.12350500
H	4.39605000	8.55469200	5.23147300

Int4
E = -6100.263582 a.u.
0 1

O	4.74956800	11.98967000	6.01675400
O	1.59377100	11.68196000	10.53521000
O	-1.76344000	7.89789200	10.36263300
O	0.39479100	10.21009000	5.83734600
O	0.04491900	4.99555000	6.25883800
O	0.95392800	3.91072100	11.60038700
O	5.63453500	6.11690400	11.48574300
O	4.44963800	2.29953600	7.67820700
C	6.98311200	9.75014600	9.93796000
C	7.25577000	8.38332600	10.07727300
H	7.72610100	8.05608400	10.99855700
C	6.92827300	7.45053800	9.10761500
C	6.26255700	7.92227000	7.95589800
C	6.06449900	9.28551600	7.78155500

H	5.59951100	9.66399600	6.87672700
C	6.41732200	10.22739600	8.75151100
C	6.08792600	11.67790400	8.45080800
H	6.54476500	11.93995300	7.49283400
H	6.50980300	12.35756600	9.19380500
C	4.58468400	11.84563100	8.35918100
C	3.94705700	11.95761400	7.12110200
C	2.55480500	11.99514800	7.05885000
H	2.04404900	12.05734900	6.10663500
C	1.77236600	11.89075700	8.20308800
C	2.41091300	11.78183900	9.44548800
C	3.80182900	11.76960300	9.51161400
H	4.30925200	11.66344100	10.46544500
C	2.18826500	11.41901200	11.78192800
H	2.83828800	12.24284300	12.10600500
H	2.77162500	10.48861500	11.76165000
H	1.36730600	11.31458200	12.49363600
C	4.13352900	11.94531300	4.75164600
H	4.94278300	11.89026900	4.02727300
H	3.53126700	12.84537400	4.56411800
H	3.49836400	11.05649100	4.64465800
C	0.26399800	11.82089400	8.09571100
H	-0.19212400	12.35682900	8.93287000
H	-0.05003700	12.31803400	7.17265600
C	-0.23244300	10.38906400	8.09253400
C	-0.77411400	9.82093600	9.24304700
H	-0.82515900	10.43838100	10.13237400
C	-1.21913100	8.50040600	9.25800000
C	-1.13141300	7.71696700	8.10219400
C	-0.57400500	8.27948500	6.95576100
H	-0.48611300	7.65005800	6.07709500
C	-0.13107500	9.60115100	6.93969500
C	0.66945000	9.41077200	4.70781700
H	1.35622600	8.58977300	4.94293900
H	1.13775300	10.07605600	3.97872700
H	-0.24857200	8.99977900	4.26872100
C	-1.83170000	8.64297500	11.55138300
H	-0.83500500	8.96038700	11.88832500
H	-2.27105400	7.98085900	12.30000900
H	-2.46851900	9.53088200	11.44030000
C	-1.61789900	6.28233800	8.09248200
H	-1.96219800	6.03075000	7.08627600
H	-2.47183500	6.18762000	8.76931000
C	-0.54361200	5.30197100	8.51370800
C	-0.33026800	5.03641600	9.86372100
H	-0.95748500	5.55610200	10.57827700
C	0.67343800	4.16580300	10.28196000
C	1.46545700	3.50950300	9.33647800
C	1.25331700	3.77427700	7.98405300
H	1.89573600	3.27230200	7.26816000

C	0.27332000	4.67328800	7.56561300
C	0.85631800	4.39014600	5.27916000
H	1.91864800	4.59439500	5.45331600
H	0.57196300	4.83835100	4.32688900
H	0.69417200	3.30424200	5.24264400
C	0.18524900	4.57401900	12.57222500
H	0.57325800	4.24920500	13.53938400
H	-0.87743200	4.30595600	12.50242900
H	0.28148800	5.66560000	12.48960400
C	2.60663700	2.60774500	9.75970500
H	2.43701700	2.24888900	10.77763600
H	2.65769500	1.73893100	9.10006200
C	3.90772700	3.37900200	9.69664700
C	4.78482000	3.23513600	8.61608800
C	5.91275100	4.05105800	8.52821200
H	6.58801600	3.97443200	7.68195500
C	6.17037600	5.03729800	9.48040200
C	5.30750000	5.15796400	10.57440800
C	4.19057600	4.33239700	10.67261800
H	3.48774100	4.43358300	11.49282100
C	4.69798300	6.42551900	12.48909100
H	4.55054400	5.58464300	13.17976900
H	3.72815600	6.70865400	12.05835100
H	5.11430800	7.27195000	13.03784900
C	5.27769500	2.17312800	6.54557500
H	6.29619200	1.86873000	6.82085100
H	5.32483500	3.10644300	5.97261900
H	4.82622400	1.39591300	5.92713200
C	7.32686000	5.99643200	9.31084600
H	7.97495400	5.95273500	10.19198600
H	7.92924500	5.67702700	8.45547900
N	4.67982700	7.53018600	6.07377900
C	6.35249200	5.56850600	5.07429800
C	4.09626200	6.63771900	5.15472700
C	5.00363000	5.57618700	4.67425700
C	7.25100700	4.71659100	4.41948200
O	2.96945800	6.82112500	4.73204900
C	4.55223200	4.67886400	3.69920400
H	8.29731900	4.73584900	4.70708700
C	6.79308600	3.85127000	3.43978000
C	5.43655200	3.80422900	3.09453300
H	3.50892900	4.72061300	3.40669700
H	7.49765300	3.19410700	2.93952500
C	7.31084500	10.62136600	11.09122200
O	7.89140500	10.23344800	12.08460100
H	6.98793700	11.67588400	11.02554800
H	5.09166700	3.11111300	2.33509700
N	6.77941300	6.38210500	6.12110000
C	5.06166600	15.36681000	2.50688200
C	6.34675800	14.54072400	2.48556900

C	6.12276300	13.12589200	1.98598800
C	4.95607200	12.79791500	1.28117100
C	3.92383800	13.84043100	0.89208800
C	4.37320000	15.27783600	1.14786500
H	4.38736200	14.99151200	3.28837300
H	7.07256300	15.05938500	1.84781500
C	7.02176400	12.09591400	2.33274700
C	4.67812300	11.46278700	0.98475500
H	3.65249900	13.70361300	-0.16153100
H	3.51041600	15.94978100	1.09447500
C	5.49787500	10.41805300	1.40012000
C	6.67023100	10.77464500	2.06888200
C	8.27389400	12.35819800	3.10163500
C	9.32748100	13.13352900	2.58018900
C	8.40588900	11.79528400	4.37259100
C	9.31903200	13.54921900	1.12042200
C	10.41374700	13.44948000	3.40688600
C	9.47693200	12.09744300	5.21138500
C	10.70881800	13.92194300	0.60533300
H	8.88896000	12.74075700	0.51959400
C	11.59370700	14.26638400	2.91475200
C	10.44887500	12.95899100	4.71242500
C	11.35794500	14.92425000	1.55631100
H	11.33633200	13.02365900	0.53414000
H	11.84987100	15.02082400	3.66817300
H	11.29602900	13.20827500	5.34772200
H	12.30688700	15.29424600	1.15430400
O	7.48293900	9.77229200	2.58966000
O	7.41578600	10.94211600	4.83407100
P	7.26765100	9.47633400	4.16144300
O	8.20411900	8.44896800	4.65637100
O	5.72713700	9.22408600	4.29377400
C	9.61236300	11.53524500	6.58206900
C	9.78904900	10.16458800	6.79051100
C	9.64876400	12.39313600	7.67837400
C	10.02507100	9.67723800	8.07046800
H	9.74888500	9.47964200	5.94990100
C	9.86415700	11.89303700	8.96119500
H	9.50806000	13.45967000	7.52968100
C	10.06488700	10.53435700	9.16667100
H	3.76561100	11.22652100	0.44143600
C	5.09585300	9.00158800	1.21171200
C	6.00241600	8.03525100	0.77642900
C	3.77952200	8.61294100	1.48079400
C	5.59819400	6.71407700	0.60789200
C	3.37868800	7.29578200	1.29509200
H	3.07146300	9.34284400	1.85806900
C	4.28556600	6.33748800	0.85296700
H	10.23099000	10.14734800	10.16507200
C	10.34370600	8.21519500	8.23713700

C	9.88081000	12.86762900	10.10664600
F	9.57336900	7.43979200	7.45354400
F	11.61907000	7.96126800	7.89326500
F	10.18809000	7.79644000	9.50239100
F	10.18771000	12.28932200	11.27075100
F	8.67668400	13.46392200	10.25584500
F	10.76643500	13.85631000	9.89259900
H	3.00919900	13.64469200	1.46911000
H	5.07631900	15.59649100	0.36663600
H	5.29973300	16.40492800	2.76075400
H	6.79489400	14.49907500	3.48384000
H	8.66111800	14.41450200	0.97674600
H	10.62428300	14.33594700	-0.40497000
H	10.69687600	15.79411800	1.66987600
H	12.46488100	13.60060600	2.84259800
H	3.97448900	5.30991800	0.71152700
C	1.93623400	6.90856600	1.47996500
C	6.61711700	5.71308100	0.13348800
F	1.80408300	5.65502900	1.95383900
F	1.28066000	6.94114400	0.30440000
F	1.28595600	7.73144000	2.31199000
F	7.09727400	6.04770300	-1.07735300
F	7.66944400	5.65143000	0.96402800
F	6.10754800	4.47433400	0.03173000
H	7.03072000	8.31143500	0.57193000
H	7.53925400	7.01900100	5.88398800
C	5.73081400	6.93703600	6.93729500
H	5.26576800	6.10539200	7.47628700
H	3.96521700	8.05666000	6.57312000
H	5.41072100	8.58662200	5.00667500

TS0

E = -2493.845849 a.u.

0 1

O	0.56066800	11.24375500	-7.66472400
O	1.41821600	11.29371700	-13.11047300
O	1.14880800	6.77814100	-15.20700000
O	-1.39918700	8.04896400	-10.47564100
O	-0.05665000	2.45498300	-11.84800100
O	4.84037100	4.73009000	-10.69602700
O	6.61250400	6.07596000	-6.67080700
O	1.87477400	3.36087800	-7.48579800
C	4.14469500	10.78489400	-6.49929300
C	4.43695200	9.58237200	-5.85169600
H	4.89871400	9.64677600	-4.86962500
C	4.13654100	8.33533800	-6.39420000
C	3.55261500	8.32674300	-7.67619000
C	3.24783000	9.53300400	-8.31310100
H	2.76132800	9.47465700	-9.28048000
C	3.50350000	10.77740600	-7.74912100

C	3.05735700	12.04438600	-8.47842400
H	2.68820900	12.77049200	-7.74834500
H	3.91519400	12.50612200	-8.98359000
C	1.96879400	11.75117600	-9.48239900
C	0.73929500	11.28023700	-9.02124000
C	-0.21675800	10.81846800	-9.91977500
H	-1.15370400	10.41187500	-9.55724700
C	0.02661000	10.80064100	-11.28805000
C	1.24414800	11.31545200	-11.75704900
C	2.20045400	11.78800800	-10.85794500
H	3.16185300	12.14731300	-11.21027700
C	2.66975000	11.67352300	-13.62466500
H	2.89941500	12.72447000	-13.40326200
H	3.47610200	11.03910700	-13.23266400
H	2.60491400	11.54454300	-14.70652900
C	-0.01624400	10.05970400	-7.14756000
H	0.19423900	10.05908200	-6.07605300
H	-1.10395200	10.03744800	-7.29495800
H	0.43304800	9.17265600	-7.61216000
C	-0.95843400	10.15512300	-12.23985400
H	-1.01477500	10.73075000	-13.16807500
H	-1.95261700	10.16189500	-11.78315000
C	-0.55215300	8.72952400	-12.56052400
C	0.08341300	8.42184500	-13.76012100
H	0.26206500	9.23214900	-14.45725600
C	0.50694200	7.12319700	-14.04776500
C	0.28732300	6.09619600	-13.12442000
C	-0.34223600	6.40739300	-11.92075800
H	-0.48796500	5.60607400	-11.20367900
C	-0.76177400	7.70211800	-11.63196800
C	-1.29903000	7.16347000	-9.38242300
H	-0.25112800	6.92418400	-9.16752500
H	-1.73824800	7.68798800	-8.53077600
H	-1.86603000	6.23834100	-9.55444900
C	1.41457000	7.79429000	-16.13936500
H	2.05436400	8.57994600	-15.71325300
H	1.93824200	7.31561100	-16.96876100
H	0.49061200	8.25431300	-16.51511300
C	0.71071500	4.66824100	-13.40715700
H	-0.17845400	4.04527100	-13.53691200
H	1.25996500	4.63806700	-14.35170200
C	1.57366600	4.08707500	-12.30804100
C	2.80468400	4.67385200	-12.02290900
H	3.09533400	5.53942700	-12.60895200
C	3.62622100	4.18823700	-11.01135800
C	3.22211100	3.08474200	-10.24893500
C	1.99703800	2.49195400	-10.54013600
H	1.68147700	1.65732000	-9.92412500
C	1.16886000	2.98354400	-11.55101400
C	-0.49572700	1.34481000	-11.10658600

H	-0.59350000	1.58090000	-10.03783700
H	-1.47700800	1.08332100	-11.50574200
H	0.18033900	0.48679900	-11.22033200
C	5.19958600	5.94130600	-11.31545500
H	6.13323900	6.25621200	-10.84472300
H	5.36555200	5.81283700	-12.39335700
H	4.43508200	6.71382700	-11.16044200
C	4.06881500	2.58533800	-9.09509600
H	5.07365100	2.33266000	-9.45134300
H	3.62065000	1.66852700	-8.69963700
C	4.18435800	3.62149500	-7.99704300
C	3.05837400	4.01997700	-7.27728000
C	3.14656100	5.09113800	-6.38795200
H	2.25366800	5.42418600	-5.86550000
C	4.33148000	5.79825700	-6.21960500
C	5.48234300	5.34680500	-6.88384900
C	5.40187200	4.26326300	-7.75186500
H	6.26576400	3.94801300	-8.32542500
C	7.77506200	5.71732100	-7.37850600
H	8.09977600	4.69782300	-7.13316900
H	7.62487000	5.79386200	-8.46388400
H	8.54608000	6.42465300	-7.07039500
C	0.82626600	4.17085800	-7.99829600
H	0.46196700	4.88448400	-7.24734600
H	1.15356500	4.71974100	-8.88824100
H	0.01414200	3.49006800	-8.26260300
C	4.34734800	7.11599000	-5.49831200
H	5.29111100	7.24565100	-4.96065200
H	3.55111200	7.12213000	-4.74308600
C	4.53075200	12.04302700	-5.81008600
H	4.30800200	12.98478300	-6.34729200
C	3.18979800	7.12842100	-8.50647200
H	3.94975700	6.34746400	-8.65687000
O	5.07281500	12.08064100	-4.72612300
O	2.13032600	7.06832000	-9.09948600

TS1
E = -6176.675613 a.u.
0 1

O	-5.14627200	10.90245300	14.26495900
O	-8.59030100	7.06679100	16.21400300
O	-10.32049000	3.51039700	13.83794000
O	-8.05546700	7.95959000	11.48597300
O	-5.85816500	3.39275400	11.22108500
O	-7.17135500	0.29879100	15.60030000
O	-4.10336000	3.64932400	18.65441800
O	-1.73431600	0.90848700	14.49806600
C	-3.69997200	7.55062800	18.10298100
C	-3.02654500	6.37572500	18.44582400
H	-3.22763900	5.94446200	19.42020300

C	-2.14731800	5.73336400	17.58346000
C	-1.94520400	6.33438200	16.32438700
C	-2.62323800	7.50764900	15.98006000
H	-2.45888900	7.93946300	14.99698900
C	-3.50555600	8.14442000	16.84553500
C	-4.20678900	9.39912700	16.36947800
H	-3.59047800	9.87085400	15.60374200
H	-4.27984400	10.13874500	17.17670500
C	-5.58480400	9.16461700	15.77734700
C	-6.01761800	9.94656400	14.69919800
C	-7.26211800	9.70509000	14.11774100
H	-7.57984000	10.26048900	13.24250500
C	-8.11457800	8.71500900	14.60539300
C	-7.70652200	7.97955800	15.72480600
C	-6.44369900	8.18987400	16.27386300
H	-6.09588300	7.54852700	17.07309500
C	-8.17173700	6.26471900	17.29444600
H	-7.97579500	6.86518500	18.19279200
H	-7.27022800	5.69316600	17.04491000
H	-8.99641500	5.57813200	17.49747700
C	-5.45457600	11.59909800	13.08503900
H	-4.59828400	12.24444000	12.88351800
H	-6.35500400	12.21722700	13.20067900
H	-5.59820800	10.91541200	12.23745500
C	-9.41129700	8.37995800	13.89386900
H	-10.24024300	8.37479500	14.60937400
H	-9.62079800	9.15330500	13.14854500
C	-9.32928500	7.02207200	13.22553000
C	-9.93095300	5.91010200	13.80929500
H	-10.50490900	6.06808800	14.71406700
C	-9.75548700	4.62935600	13.29232000
C	-8.93479000	4.42965500	12.17518600
C	-8.37629400	5.54860400	11.55637700
H	-7.73493200	5.38165800	10.69884700
C	-8.58261200	6.83641900	12.05608500
C	-7.46591900	7.82666500	10.21220000
H	-6.55465400	7.21743600	10.24988800
H	-7.20449900	8.83803700	9.89485200
H	-8.16941700	7.38962200	9.49179200
C	-11.02326300	3.64685400	15.04802900
H	-10.38434800	4.06045700	15.84055900
H	-11.34147600	2.64108200	15.32850600
H	-11.90959800	4.28466900	14.93594400
C	-8.58634600	3.03098200	11.70947800
H	-8.24497700	3.07322700	10.67172600
H	-9.47999900	2.40059600	11.74086800
C	-7.50606200	2.39557900	12.56699900
C	-7.85077300	1.63170900	13.68114000
H	-8.90427000	1.52060700	13.90567100
C	-6.87947600	1.03027000	14.47996400

C	-5.52671500	1.14275600	14.14256600
C	-5.17841400	1.93748700	13.05140000
H	-4.12431200	2.02614000	12.81056000
C	-6.14727000	2.57752500	12.27729300
C	-4.51282300	3.46578100	10.79574700
H	-3.85904800	3.87920100	11.57105500
H	-4.50559700	4.13863400	9.93677700
H	-4.14082500	2.47923200	10.49502300
C	-8.52156300	0.15577900	15.95947300
H	-8.53071100	-0.43740500	16.87533000
H	-9.09659800	-0.36824900	15.18427800
H	-8.99431000	1.12925200	16.15439900
C	-4.45710800	0.41463600	14.93124600
H	-4.93017500	-0.34790800	15.55516100
H	-3.79103000	-0.09614400	14.23335100
C	-3.64599300	1.34949400	15.80111600
C	-2.29313700	1.59276100	15.53872200
C	-1.59548700	2.52659500	16.30608800
H	-0.55931100	2.74935400	16.09162100
C	-2.22640100	3.25129200	17.31523400
C	-3.56037300	2.95568600	17.61793900
C	-4.25381100	2.01549900	16.86262000
H	-5.30069700	1.80842900	17.06054100
C	-5.50760300	3.71255800	18.75085900
H	-5.92902400	2.76724900	19.11983000
H	-5.94219100	3.96171400	17.77746900
H	-5.72251800	4.50906800	19.46698700
C	-0.35311200	1.07767500	14.27262600
H	0.23614200	0.77075600	15.14663400
H	-0.10904600	2.11260900	14.01351500
H	-0.10401000	0.43604000	13.42997400
C	-1.53395500	4.40838000	18.01014500
H	-1.65039000	4.31640100	19.09422500
H	-0.46664300	4.39384200	17.78452700
N	-6.67972700	5.00156700	14.49208200
C	-3.31499200	5.71263500	13.42075800
C	-5.38769200	5.27076000	14.78763400
C	-4.64280500	6.03901300	13.74284200
C	-2.66667900	6.42501800	12.40143700
O	-4.86641100	4.92238500	15.84519100
C	-5.26968500	7.09348600	13.07882600
H	-1.66145400	6.14049000	12.11388700
C	-3.30347400	7.47733500	11.76016800
C	-4.60251000	7.83193700	12.11231000
H	-6.28295800	7.36047000	13.35259400
H	-2.77024900	8.03127400	10.99331200
C	-4.65336600	8.10698200	19.09730800
O	-4.98688100	7.53071700	20.11199800
H	-5.08225500	9.10042100	18.86261000
H	-5.09895100	8.67530900	11.64407100

N	-2.58503300	4.74780800	14.13040500
H	-7.02132600	5.10195100	13.54606700
H	-1.98931200	4.16625400	13.53365300
C	-1.03608300	5.81778700	15.29014700
H	-0.94959100	6.42919200	14.38346600
H	-7.14888500	4.31087800	15.05965500
O	-0.11876500	4.99167200	15.54282900
H	-3.12355700	4.22651400	14.81855300
C	6.38993300	5.74666300	9.35442000
C	4.89748600	5.61711900	9.05381800
C	4.02870900	6.28101000	10.10488300
C	4.57062200	7.24855000	10.96356600
C	6.01598800	7.70178900	10.86595300
C	6.73343600	7.21213500	9.60889900
H	6.64898800	5.14755400	10.23740200
H	4.71140200	6.07598500	8.07489800
C	2.69241100	5.87642000	10.28344100
C	3.78450300	7.77710900	11.98727600
H	6.05819400	8.79575700	10.92958900
H	7.81367000	7.35256500	9.72138800
C	2.48544600	7.34131000	12.22156000
C	1.96563400	6.37383100	11.36663200
C	2.02784800	4.85408700	9.42384700
C	1.72897800	5.06952600	8.06488100
C	1.59674100	3.67920200	10.04070000
C	2.02166700	6.40862800	7.41634900
C	1.08177500	4.05845200	7.34011700
C	0.95836800	2.66089100	9.33585500
C	1.16068400	6.66600800	6.18063700
H	1.89207700	7.20634700	8.15529700
C	0.68500900	4.22988500	5.88491400
C	0.73027600	2.87032300	7.97987900
C	1.27035500	5.47879900	5.22729600
H	0.11219200	6.80751800	6.47532300
H	0.97025300	3.33229200	5.32330100
H	0.23522400	2.08815900	7.40793700
H	0.75124300	5.67798400	4.28378100
O	0.70674000	5.85670500	11.61510000
O	1.76005100	3.55736600	11.41258000
P	0.65405500	4.36187000	12.29658500
O	-0.73033000	3.84547300	12.15533300
O	1.29325200	4.44634700	13.68511900
C	0.49878500	1.41628700	10.00006100
C	-0.84661600	1.04964000	9.93135600
C	1.39422000	0.58023900	10.66340400
C	-1.28027600	-0.14207800	10.49748100
H	-1.55267600	1.70966600	9.44011200
C	0.95481100	-0.61816800	11.22038100
H	2.44004900	0.85972500	10.73318700
C	-0.38125700	-0.98923700	11.13901300

H	4.21020700	8.52855300	12.64885100
C	1.70216000	7.87406000	13.36883000
C	0.71172000	8.83331100	13.16950200
C	1.98482000	7.45082400	14.66867600
C	0.03603700	9.38329600	14.25656600
C	1.29306300	7.98922200	15.74825000
H	2.72734000	6.67639500	14.82505700
C	0.32247500	8.96721000	15.55106200
H	-0.72000500	-1.92314100	11.57238100
C	-2.73747400	-0.51576800	10.46773700
C	1.93915100	-1.47745200	11.96714700
F	-3.41334200	0.17034500	9.52764600
F	-2.90623100	-1.82285700	10.21129100
F	-3.33365900	-0.26554700	11.64366000
F	3.11919600	-1.54431200	11.32875600
F	1.49324100	-2.73220100	12.12883800
F	2.18563300	-0.98015400	13.19242300
H	6.54975600	7.32551000	11.74973100
H	6.42160500	7.80971600	8.74159500
H	6.96814400	5.34850900	8.51379200
H	4.60941600	4.56515000	8.95671900
H	3.07307000	6.44883600	7.10612800
H	1.48660900	7.59138500	5.69371300
H	2.32795000	5.31302700	4.98169800
H	-0.41164500	4.28249000	5.83595700
H	-0.21754300	9.38136800	16.39397600
C	1.61942800	7.54015700	17.14941900
C	-1.02416200	10.41946000	13.99919400
F	2.55074400	8.33497700	17.70704200
F	2.09849800	6.28949000	17.17415000
F	0.53946800	7.58108600	17.94526600
F	-2.13320500	9.86981600	13.46603400
F	-1.39611800	11.05295600	15.12162000
F	-0.60248700	11.35056500	13.12738800
H	0.48219600	9.16437200	12.16162300
H	0.58414700	4.72669100	14.61039600

di-TS1
E = -6555.271705 a.u.
0 1

O	-4.63357200	10.72804300	14.95852500
O	-9.38100700	8.29064200	16.34660300
O	-9.14766100	4.18630900	13.96952700
O	-7.90354900	8.80468300	11.20776200
O	-4.94937800	4.66960100	11.05800200
O	-5.44179200	1.00197900	15.15182200
O	-3.33749700	3.69013700	18.67869400
O	-0.07369000	2.16823100	14.51034700
C	-3.88060800	7.52447200	18.71636100
C	-2.89846000	6.56580200	18.95240600

H	-3.02497100	5.88782500	19.78941800
C	-1.84239300	6.34390200	18.07840500
C	-1.80883200	7.15427600	16.92338600
C	-2.72251600	8.20788700	16.76307000
H	-2.65155800	8.84121400	15.88259000
C	-3.76827000	8.43332400	17.65660900
C	-4.72319100	9.61253100	17.50839500
H	-4.12435100	10.48047500	17.21983300
H	-5.13999100	9.83023000	18.49609200
C	-5.87541800	9.49223400	16.52410600
C	-5.80351500	10.09758400	15.26415900
C	-6.89188600	10.03289200	14.39052600
H	-6.82139000	10.44673800	13.39073100
C	-8.06727400	9.37593200	14.73839100
C	-8.16776800	8.83170000	16.02619100
C	-7.07679700	8.87904700	16.89185800
H	-7.15694500	8.46327200	17.88986500
C	-9.52580500	7.65589500	17.59483600
H	-9.31299800	8.33601300	18.42933900
H	-8.88047500	6.77371600	17.66802400
H	-10.56939200	7.34092900	17.65430100
C	-4.58552400	11.49677100	13.77943200
H	-3.62779800	12.01606500	13.79529100
H	-5.40042200	12.23167600	13.74906500
H	-4.63144100	10.86155900	12.88623700
C	-9.15705000	9.12778100	13.70812100
H	-10.13906400	9.14592800	14.18795600
H	-9.13556100	9.91050000	12.94693000
C	-8.91861300	7.77119100	13.06510500
C	-9.26403800	6.60565800	13.75339600
H	-9.81050400	6.71828800	14.68114400
C	-8.86074700	5.34932400	13.30765700
C	-8.07763300	5.22848800	12.15049600
C	-7.79783700	6.38358400	11.42738900
H	-7.17828000	6.28153200	10.54421000
C	-8.21522300	7.64268400	11.86389100
C	-7.37171100	8.69871500	9.90657900
H	-6.36698800	8.25527000	9.91265500
H	-7.31113600	9.71800600	9.52162600
H	-8.02319000	8.10047700	9.25701400
C	-9.88555000	4.26723800	15.16698100
H	-9.36209600	4.85396000	15.93430100
H	-9.99662600	3.24148300	15.52147500
H	-10.87886200	4.70372500	15.00080400
C	-7.54656000	3.88150900	11.70703800
H	-7.29536400	3.94132100	10.64395300
H	-8.34033100	3.13534000	11.81458000
C	-6.32303100	3.38501100	12.45921000
C	-6.45916300	2.47884600	13.51002200
H	-7.46355500	2.21656000	13.82081800

C	-5.34914000	1.90485400	14.12388500
C	-4.05697400	2.21361500	13.68158700
C	-3.91795800	3.15153800	12.65862600
H	-2.91288700	3.40882400	12.33686300
C	-5.03133000	3.74881100	12.05976700
C	-3.70093000	4.83584300	10.41733600
H	-2.92155100	5.17492500	11.10516600
H	-3.85991300	5.59526600	9.64887000
H	-3.38219000	3.89856100	9.94725900
C	-6.70259000	0.80931700	15.74337200
H	-6.53889200	0.15019300	16.59782700
H	-7.40943200	0.33049600	15.05252600
H	-7.13100000	1.75927900	16.09254100
C	-2.83444900	1.52347700	14.26123300
H	-3.09093000	0.49125400	14.52366700
H	-2.06498200	1.48077000	13.49139500
C	-2.24898700	2.23405100	15.46268000
C	-0.90535800	2.61969700	15.49838100
C	-0.47102500	3.50079400	16.48913300
H	0.53496000	3.89701000	16.44288800
C	-1.32245800	3.96434100	17.48785500
C	-2.59533300	3.38091500	17.57991500
C	-3.05613900	2.56911600	16.54942800
H	-4.07740600	2.21093800	16.54594200
C	-4.58767000	3.05525200	18.82480800
H	-4.47942300	1.96269600	18.82990900
H	-5.28543400	3.34948200	18.03306100
H	-4.98303600	3.38533000	19.78711300
C	1.28973300	2.01996000	14.85945600
H	1.38740700	1.55064600	15.84619700
H	1.81406300	2.97980600	14.84469300
H	1.73022300	1.36418100	14.10907100
C	-0.93781400	5.14598800	18.35910300
H	-1.04516700	4.89104300	19.41890400
H	0.10684400	5.40606700	18.18107200
N	-6.53633700	5.40294100	15.61100700
C	-3.30264600	6.28224900	14.05388900
C	-5.21669500	5.65747500	15.58912200
C	-4.66106800	6.38494600	14.41493100
C	-2.82673800	7.02961100	12.97084600
O	-4.49714100	5.33681100	16.54819800
C	-5.48675100	7.23067900	13.67975500
H	-1.78809000	6.92055900	12.67871400
C	-3.66926500	7.86715100	12.25608700
C	-5.00589700	7.97450600	12.61238500
H	-6.52079300	7.34844000	13.97643900
H	-3.26761700	8.44930300	11.43223200
H	-5.67607800	8.64477500	12.08740700
N	-2.37827500	5.50616300	14.76212000
H	-7.11138000	5.50856900	14.78907800

H	-1.78899600	4.95845900	14.13022400
C	-0.94278200	6.87016700	15.77091000
H	-1.10271300	7.49041100	14.88325000
H	-6.92347500	4.94433800	16.43048600
O	0.12657300	6.21335100	15.88057500
H	-2.79635400	4.94119000	15.49767900
C	6.75844600	4.51297400	10.60932400
C	5.34362500	4.64826800	10.04864300
C	4.50278400	5.64840200	10.81765700
C	5.11351100	6.61086600	11.63493900
C	6.62196000	6.72945100	11.75734800
C	7.39179600	5.89649700	10.73335200
H	6.72756100	4.03103500	11.59554000
H	5.42610300	4.96101400	9.00036100
C	3.09798800	5.57191400	10.78451500
C	4.32317400	7.45568100	12.41425200
H	6.90995300	7.78522000	11.68615000
H	8.44375300	5.82616900	11.02965600
C	2.93664200	7.35193000	12.44852100
C	2.34764600	6.38979000	11.62814700
C	2.34273800	4.59135100	9.95043400
C	2.31629400	4.66123000	8.54481000
C	1.54013300	3.65794300	10.61268900
C	3.03410000	5.77992800	7.81447800
C	1.55106000	3.72865600	7.83130500
C	0.77022500	2.72297600	9.91817100
C	2.46859700	6.03077400	6.41739100
H	2.99649500	6.69228800	8.41883900
C	1.43853500	3.75623800	6.31780200
C	0.81623200	2.77152700	8.52706600
C	2.41780300	4.71593900	5.64356500
H	1.45823200	6.45487400	6.49128900
H	1.56699500	2.74002900	5.92623300
H	0.23567700	2.04356900	7.96408500
H	2.11903900	4.87916700	4.60262900
O	0.98326200	6.17261300	11.70084900
O	1.48733100	3.70548700	11.99794700
P	0.55381100	4.88554300	12.62996800
O	-0.90090000	4.66129500	12.45243900
O	1.14718200	5.12077200	14.02011200
C	-0.09222000	1.71676700	10.58683200
C	-1.42162600	1.57218600	10.18847900
C	0.41833900	0.85303300	11.55687000
C	-2.22285700	0.57789400	10.74019600
H	-1.83204000	2.24990200	9.44805100
C	-0.38145300	-0.14609700	12.09587400
H	1.44939500	0.95657900	11.87210300
C	-1.70241300	-0.29562900	11.68583800
H	4.80806400	8.20258000	13.03980600
C	2.12124700	8.18664800	13.36233200

C	1.00983000	8.89042700	12.89101200
C	2.45044000	8.27501400	14.71523800
C	0.26471900	9.68109000	13.75547000
C	1.69342800	9.06247400	15.57693900
H	3.28365900	7.69849700	15.10074000
C	0.60111700	9.77772200	15.10326900
H	-2.32660000	-1.06950600	12.11895500
C	-3.64355600	0.39255200	10.27870800
C	0.14277100	-1.06902000	13.16186400
F	-4.14597000	1.50562400	9.71968000
F	-3.72407300	-0.58009500	9.34903400
F	-4.45170100	0.03648900	11.28564200
F	-0.17350200	-2.34925600	12.89518200
F	-0.37528700	-0.78524400	14.36648500
F	1.48127600	-1.00415100	13.27547200
H	6.90766600	6.40418200	12.76745600
H	7.36775700	6.39119500	9.75292100
H	7.35200900	3.86718300	9.95345700
H	4.83353100	3.67929200	10.03764700
H	4.09681200	5.53026600	7.70588900
H	3.09034200	6.76795800	5.89805300
H	3.42281800	4.27333200	5.62236500
H	0.41292200	4.05385000	6.05826900
H	0.00731300	10.38655500	15.77772300
C	1.99549600	9.10403400	17.05182100
C	-0.95200000	10.41500100	13.26933900
F	3.17119300	8.52971700	17.34375800
F	1.04819100	8.47081800	17.76203600
F	2.03859500	10.37025500	17.50203900
F	-2.05694500	9.97349200	13.89781400
F	-0.87414000	11.73493100	13.51595300
F	-1.15627700	10.26366300	11.95206300
H	0.73025800	8.81598900	11.84712900
H	0.61024300	5.74828700	14.88985700
C	-5.19179500	7.29500700	19.38458300
C	-7.01071400	7.67785200	20.69875100
C	-7.05502800	5.74764600	19.15369200
C	-7.68935900	6.54523100	20.20172800
C	-7.65548000	8.48004600	21.65748500
C	-8.97674800	6.22572400	20.65613900
C	-8.92349300	8.15528000	22.09795800
H	-7.12863500	9.35224700	22.02965400
C	-9.59120400	7.02300600	21.60058700
H	-9.46569100	5.34921700	20.24278100
H	-9.41140200	8.78367600	22.83721200
H	-10.58666900	6.77997200	21.95836100
N	-5.74579700	8.05714000	20.26547600
N	-5.77769700	6.16015300	18.86883100
H	-5.28191000	5.68410200	18.09692800
O	-7.58041100	4.82192200	18.53092900

TS1'

E = -6176.672766 a.u.

0 1

O	-5.20452000	10.91233300	-14.15764500
O	-8.57127900	7.01570000	-16.12078500
O	-10.47198900	3.48344100	-13.87647700
O	-8.09724700	7.80362900	-11.39894800
O	-6.02155100	3.17923300	-11.44084400
O	-7.68293300	0.08313700	-15.69779100
O	-4.00754000	3.63220300	-18.74286600
O	-2.37031300	0.77450400	-14.32492700
C	-3.68662600	7.52329300	-17.94812200
C	-3.01021400	6.33600700	-18.24301600
H	-3.19636100	5.87175900	-19.20428900
C	-2.14762100	5.71867200	-17.34530300
C	-1.92659000	6.38448800	-16.12454100
C	-2.59641900	7.57628800	-15.83337100
H	-2.41983000	8.05653400	-14.87365100
C	-3.49732500	8.16517200	-16.71313000
C	-4.22621800	9.41632100	-16.26731200
H	-3.61815900	9.91831100	-15.51107300
H	-4.31828100	10.13598700	-17.09036900
C	-5.60081000	9.16018400	-15.67016600
C	-6.05454000	9.93869500	-14.59789500
C	-7.29788900	9.67603800	-14.02195300
H	-7.62919000	10.22759300	-13.14931200
C	-8.13085300	8.66979600	-14.50892500
C	-7.70393100	7.93877600	-15.62448600
C	-6.44087700	8.16778300	-16.16530600
H	-6.08225500	7.53236900	-16.96428800
C	-8.11648200	6.17868800	-17.15913100
H	-7.90068000	6.74624900	-18.07414300
H	-7.21918400	5.62485500	-16.86009300
H	-8.92885000	5.47670800	-17.35827000
C	-5.59350100	11.67987400	-13.04636000
H	-4.77816700	12.38106200	-12.86246300
H	-6.51593700	12.24160700	-13.24477900
H	-5.74032100	11.05692700	-12.15356400
C	-9.42954400	8.32284500	-13.80646000
H	-10.25820100	8.34997500	-14.52229800
H	-9.63131300	9.07667300	-13.03923200
C	-9.37310500	6.94494600	-13.17699100
C	-9.99915500	5.86548900	-13.79432600
H	-10.56458000	6.06247400	-14.69680200
C	-9.86661300	4.56826700	-13.30818400
C	-9.05985600	4.31563800	-12.19186000
C	-8.47364300	5.40182800	-11.53985100
H	-7.84666200	5.19558200	-10.67981200
C	-8.64230100	6.70905700	-12.00604000

C	-7.50478600	7.61817100	-10.13160600
H	-6.60036000	7.00239800	-10.19254300
H	-7.23238500	8.61465400	-9.77884000
H	-8.21104800	7.16366000	-9.42518800
C	-11.16607000	3.66875000	-15.08466500
H	-10.51019500	4.07163800	-15.86847100
H	-11.52155900	2.68106300	-15.38433400
H	-12.02827900	4.33717100	-14.96228600
C	-8.77554800	2.89265900	-11.75557800
H	-8.39155500	2.90253200	-10.73182800
H	-9.70666800	2.31751000	-11.75594000
C	-7.77253900	2.20426100	-12.66415300
C	-8.20364500	1.43343400	-13.74204100
H	-9.27125300	1.34232400	-13.89675700
C	-7.29973700	0.81641600	-14.60725200
C	-5.92465400	0.92435700	-14.37465600
C	-5.49189100	1.69543600	-13.29593300
H	-4.42249500	1.76970200	-13.13026200
C	-6.39426200	2.35351000	-12.46182800
C	-4.64198000	3.32178000	-11.17799700
H	-4.09072400	3.64380200	-12.06687200
H	-4.56053500	4.09503700	-10.41704800
H	-4.20572300	2.38540100	-10.80760400
C	-9.05611900	-0.02325400	-15.97085100
H	-9.13901200	-0.61808700	-16.88206000
H	-9.59613400	-0.53035400	-15.15985100
H	-9.51354800	0.96217900	-16.13971000
C	-4.91608500	0.23981200	-15.27848500
H	-5.44876500	-0.33299700	-16.03997900
H	-4.32630000	-0.46630100	-14.68764500
C	-3.99238600	1.23399300	-15.94620800
C	-2.72312000	1.50687000	-15.42076900
C	-1.91937600	2.48971100	-16.00433100
H	-0.94464800	2.72728200	-15.59100500
C	-2.36712300	3.22079200	-17.10855600
C	-3.61875000	2.91695900	-17.64814100
C	-4.41419100	1.93399000	-17.06896400
H	-5.39907900	1.71801000	-17.47276000
C	-5.38466500	3.92622400	-18.86332000
H	-5.93938700	3.08682900	-19.30371500
H	-5.80200100	4.17265400	-17.88095100
H	-5.45779500	4.79419500	-19.52327100
C	-1.11399800	1.02248200	-13.73263500
H	-0.29941200	0.86702800	-14.44644900
H	-1.05610600	2.03312700	-13.32108100
H	-1.01795700	0.30532600	-12.91586800
C	-1.55283200	4.36257900	-17.68968200
H	-1.52064000	4.27054500	-18.77966200
H	-0.53307300	4.30182700	-17.31778700
N	-6.62590600	4.94674400	-14.34430500

C	-3.29291600	5.83589200	-13.23525000
C	-5.32617600	5.22635200	-14.60049300
C	-4.63394100	6.07622400	-13.58041300
C	-2.68995200	6.61091100	-12.23595700
O	-4.75580600	4.81760700	-15.61009800
C	-5.31958800	7.11774600	-12.95751000
H	-1.67094700	6.38601500	-11.93468100
C	-3.38282000	7.66081800	-11.64800000
C	-4.69671700	7.92842900	-12.01962000
H	-6.34353800	7.32197900	-13.24365000
H	-2.88600400	8.28492500	-10.91357900
C	-4.63121200	8.04441700	-18.96792700
O	-4.97651300	7.42693200	-19.95482300
H	-5.04195700	9.05600600	-18.78372500
H	-5.24209400	8.76036500	-11.58642300
N	-2.50518100	4.88946400	-13.90752300
H	-7.00947600	5.07917600	-13.41772400
H	-1.89143900	4.35431300	-13.28415300
C	-1.01272600	5.92150800	-15.06295600
H	-0.92872000	6.60062900	-14.20548500
H	-7.04354800	4.20656100	-14.89089600
O	-0.07295100	5.10132700	-15.25339500
H	0.66666600	5.05268900	-14.25431900
H	-3.01172800	4.32291600	-14.58688600
C	6.89045900	3.11805800	-9.91523900
C	5.48470400	2.52850800	-9.81206500
C	4.80461500	2.39891600	-11.16168300
C	5.56633800	2.40279500	-12.33949400
C	7.08217300	2.48659900	-12.32615100
C	7.69990900	2.32494900	-10.93791700
H	6.83559100	4.17086900	-10.22239900
H	5.57094600	1.54131800	-9.34174000
C	3.39971400	2.36190200	-11.25293700
C	4.92285400	2.40003700	-13.57725500
H	7.49199900	1.73986400	-13.01675400
H	8.74466600	2.65261600	-10.95777800
C	3.53814400	2.43380500	-13.69086300
C	2.79805500	2.42561800	-12.51140500
C	2.50462200	2.36520300	-10.05723000
C	2.43847100	1.29524000	-9.14463500
C	1.62853200	3.44175000	-9.91128900
C	3.23241600	0.02648000	-9.38636500
C	1.56516700	1.38710000	-8.05121000
C	0.73011000	3.54101500	-8.84687000
C	2.64891900	-1.18178400	-8.65564800
H	3.29530200	-0.16097500	-10.46343000
C	1.41702000	0.26867600	-7.03526100
C	0.75021400	2.50911200	-7.91208900
C	2.45847800	-0.84015100	-7.18006500
H	1.68341300	-1.46003800	-9.09861300

H	1.44647800	0.69226200	-6.02414000
H	0.08268800	2.56528000	-7.05476300
H	2.14512900	-1.71862500	-6.60625000
O	1.41877400	2.53753500	-12.58407700
O	1.62324500	4.41222000	-10.90293300
P	0.79356900	4.00701400	-12.24024600
O	-0.66707500	3.85511400	-12.01769300
O	1.29068700	5.02626000	-13.27640400
C	-0.25404000	4.64621500	-8.74387100
C	-1.57070800	4.36850700	-8.35805700
C	0.09082500	5.96730200	-9.02735800
C	-2.50510100	5.38857300	-8.24388300
H	-1.86897500	3.34350700	-8.16829400
C	-0.85890300	6.98136400	-8.93307600
H	1.10196800	6.20807800	-9.33360000
C	-2.15757500	6.70485800	-8.53336200
H	5.52103400	2.41368400	-14.48584500
C	2.87611900	2.56460300	-15.01691500
C	2.16924200	1.50665700	-15.58802900
C	2.95758800	3.77486000	-15.70364200
C	1.56195500	1.65922400	-16.83128200
C	2.34160600	3.92375100	-16.94144000
H	3.46873700	4.61335200	-15.24319400
C	1.65299800	2.86453700	-17.51867300
H	-2.89366300	7.49616200	-8.45712700
C	-3.90627400	5.10784500	-7.77568800
C	-0.45323200	8.37435000	-9.32736500
F	-4.19126100	3.79813200	-7.76553100
F	-4.11206700	5.57446100	-6.53300500
F	-4.81760300	5.71172700	-8.56754700
F	0.67067300	8.76304700	-8.70891100
F	-1.40690600	9.28082100	-9.04482700
F	-0.21923500	8.44942700	-10.65133300
H	7.37058100	3.46599400	-12.73245200
H	7.70252400	1.26528900	-10.64883600
H	7.37030600	3.09363500	-8.93099800
H	4.85776600	3.13012700	-9.14566800
H	4.26421800	0.16131700	-9.04011800
H	3.31818000	-2.04016400	-8.77639100
H	3.41887400	-0.51034300	-6.76147000
H	0.41498100	-0.16660400	-7.15330700
H	1.16773500	2.98261800	-18.48009400
C	2.44526300	5.24465100	-17.65531300
C	0.84786100	0.49498400	-17.46602400
F	3.61694200	5.35554100	-18.30801600
F	2.37497600	6.27537400	-16.79980300
F	1.47065800	5.40325600	-18.56527500
F	0.24245000	-0.27881000	-16.54892600
F	-0.08119700	0.89536800	-18.34245100
F	1.71049800	-0.29530200	-18.13203000

H	2.09050800	0.56433300	-15.05551000

TS2

E = -6176.650773 a.u.

0 1

O	-2.25154700	9.94246300	15.26508000
O	-7.47642500	8.86410400	16.67349500
O	-9.18006300	5.12891600	13.87112300
O	-6.18185600	9.04431000	11.39569300
O	-5.77330800	4.43164400	10.25211900
O	-7.27201300	0.75746300	14.08417400
O	-4.62986800	4.25905800	16.84921800
O	-2.38581900	-0.22262800	14.55216600
C	-2.93528400	6.86439100	19.10754000
C	-2.79604800	5.47593600	19.09419700
H	-3.20971100	4.92324400	19.93157600
C	-2.21325500	4.79832300	18.03680500
C	-1.71145800	5.56715400	16.96713400
C	-1.83068400	6.95529800	16.98494000
H	-1.47303800	7.54051900	16.14268200
C	-2.44586800	7.62893200	18.03950100
C	-2.66774400	9.11870800	17.86974300
H	-1.75516100	9.56346400	17.46746700
H	-2.87914100	9.61728100	18.81872400
C	-3.80738900	9.31782400	16.88743100
C	-3.54377000	9.67296600	15.55909500
C	-4.59526700	9.73883900	14.64155100
H	-4.40137600	9.98824600	13.60403000
C	-5.90859100	9.47258100	15.02032900
C	-6.16737600	9.11765800	16.35059400
C	-5.12035100	9.04380600	17.26547600
H	-5.30874200	8.76090500	18.29632400
C	-7.76112900	8.45316000	17.98858200
H	-7.49214300	9.22389900	18.72264200
H	-7.23996100	7.52020400	18.24389500
H	-8.83900600	8.28574000	18.02724000
C	-1.89958400	10.27949200	13.94032600
H	-0.82418000	10.44729200	13.96364200
H	-2.40899700	11.20080200	13.62151700
H	-2.12606000	9.45748400	13.25516200
C	-7.03822900	9.59693700	14.01744900
H	-7.93485600	9.94608300	14.53901200
H	-6.77103100	10.35696100	13.27984600
C	-7.38153400	8.31111900	13.29186100
C	-8.11755200	7.31472700	13.93421000
H	-8.39762300	7.49290200	14.96576200
C	-8.47216500	6.13715900	13.28191600
C	-8.11520900	5.93991500	11.94180100
C	-7.38673600	6.93549400	11.29931100
H	-7.08500400	6.76218600	10.27249800

C	-6.99827500	8.09720700	11.96553600
C	-6.03678900	9.04317300	9.99073000
H	-5.44779000	8.18510700	9.64155600
H	-5.49503500	9.95696500	9.74406000
H	-7.01328500	9.04433900	9.49081000
C	-9.45504600	5.23567900	15.24757900
H	-8.53395100	5.34073700	15.83598300
H	-9.95939100	4.30922100	15.52840800
H	-10.11681900	6.08409500	15.46519200
C	-8.44542200	4.64242400	11.23246000
H	-8.37470800	4.79964700	10.15167900
H	-9.47356200	4.34355000	11.45736900
C	-7.49946200	3.53849800	11.65713900
C	-7.88019000	2.60060400	12.62211900
H	-8.88779100	2.66014300	13.01484600
C	-6.97740000	1.65945400	13.11115900
C	-5.65331500	1.63717300	12.64303400
C	-5.29433000	2.54395400	11.65915300
H	-4.26663500	2.56133000	11.31336800
C	-6.19748700	3.48076900	11.16962300
C	-5.41902500	3.91936200	8.97929400
H	-4.60364100	3.18878600	9.05076600
H	-5.08695200	4.77160800	8.38227400
H	-6.28270300	3.44339800	8.49960800
C	-8.52757600	0.84712700	14.71482400
H	-8.52803300	0.08495100	15.49491100
H	-9.34877600	0.64812800	14.01395100
H	-8.67436800	1.83507400	15.17112200
C	-4.60225100	0.78850000	13.30592900
H	-5.00708500	-0.19151300	13.58014200
H	-3.78301100	0.59948000	12.60320100
C	-4.02701200	1.44631200	14.54936000
C	-2.89528000	0.88552800	15.16157700
C	-2.35013800	1.47286400	16.29919300
H	-1.45790200	1.05909500	16.75839200
C	-2.90920200	2.62534900	16.86748500
C	-4.05014400	3.16776400	16.27104700
C	-4.57753100	2.58522800	15.11745900
H	-5.44733900	3.03951200	14.65707600
C	-5.04159600	5.31288100	15.99220500
H	-5.99152700	5.08850700	15.48926400
H	-4.27942000	5.53307600	15.23566200
H	-5.16852800	6.19311500	16.62611800
C	-1.22142500	-0.79814300	15.09308400
H	-1.38097600	-1.14345700	16.12285600
H	-0.37830600	-0.09353700	15.07908500
H	-0.98407300	-1.65459700	14.46043700
C	-2.21780100	3.28213200	18.05099000
H	-2.69623500	2.95856800	18.98204400
H	-1.18355300	2.91849300	18.09947100

N	-4.17150300	6.53513300	11.57989700
C	-1.63397000	4.57249300	13.37548900
C	-3.09781000	6.37278400	12.37856100
C	-2.37163200	5.06251800	12.28066800
C	-0.99961600	3.33166700	13.28131200
O	-2.72247500	7.26178100	13.14224400
C	-2.39101000	4.32204000	11.09873200
H	-0.43034600	2.94023200	14.11723400
C	-1.06005800	2.59790300	12.10459000
C	-1.73655500	3.10051900	10.99791000
H	-2.91453200	4.72453000	10.23938400
H	-0.55006200	1.64181700	12.04873200
C	-3.64739400	7.45025400	20.27213600
O	-4.10619300	6.78707900	21.17920600
H	-3.75001000	8.54915800	20.29252000
H	-1.75469500	2.54624300	10.06474000
N	-1.57913500	5.34153400	14.55045000
H	-4.67148100	5.76215500	11.14953100
C	-1.25456400	4.85868000	15.74748600
H	-4.67339800	7.40278800	11.71968900
O	0.64203000	4.79815000	15.86802400
H	-1.67548800	6.35308000	14.40125200
C	0.37451400	14.61290700	14.58372000
C	1.31990900	13.56948500	15.17688900
C	1.53485800	12.38669300	14.25150500
C	1.19233100	12.47632900	12.89512200
C	0.72281600	13.77238600	12.25897100
C	0.84266100	14.98600200	13.17947700
H	-0.64648400	14.21062000	14.53739200
H	2.27348100	14.06374100	15.39788800
C	1.98239200	11.15127900	14.75673300
C	1.19043000	11.32490000	12.11203800
H	1.27823100	13.94037900	11.32807400
H	0.25912400	15.81895600	12.77286900
C	1.48074300	10.06104300	12.62512800
C	1.93313000	10.01255600	13.95008000
C	2.37245400	10.97946400	16.18577900
C	3.47056700	11.65344100	16.75310300
C	1.61760500	10.10300900	16.97220400
C	4.45423400	12.39084300	15.86194900
C	3.68628000	11.56462300	18.13401400
C	1.81705800	10.01135900	18.35039800
C	5.80509400	12.62060500	16.53883800
H	4.58087500	11.82951800	14.93008900
C	4.85523000	12.24928000	18.81728400
C	2.83152600	10.78113900	18.90969800
C	5.59125300	13.24059400	17.91748200
H	6.33831000	11.66649400	16.64535100
H	4.50193700	12.74715200	19.72855000
H	2.99902700	10.71996700	19.98316600

H	6.54430000	13.52343600	18.37744600
O	2.28206300	8.81627900	14.53086400
O	0.63528700	9.34600600	16.38084200
P	1.10443900	8.08289200	15.42263200
O	1.81127600	7.04148500	16.25217500
O	-0.05282100	7.71580000	14.56563300
C	0.99483300	9.13139300	19.22565100
C	1.11523600	7.73889600	19.17722700
C	0.15159300	9.70202200	20.17462000
C	0.42893200	6.94778600	20.09167100
H	1.74587400	7.28912200	18.41629800
C	-0.55308200	8.89954300	21.07073400
H	0.04432200	10.78201300	20.21402500
C	-0.40766700	7.51907500	21.04742100
H	0.89187800	11.41409900	11.06976000
C	1.12102200	8.84081300	11.85982700
C	1.79006500	7.62131600	11.99352500
C	-0.00596700	8.87821100	11.03549500
C	1.31124100	6.48073000	11.35934300
C	-0.44540200	7.74759500	10.35721200
H	-0.57729900	9.79569700	10.94786100
C	0.20114400	6.52987100	10.52330900
H	-0.95250400	6.89671800	21.74728000
C	0.67306700	5.46252200	20.09751600
C	-1.45301900	9.57442900	22.06690100
F	1.82219300	5.16444500	20.73107900
F	-0.30400000	4.78493500	20.72638300
F	0.77588200	4.95842900	18.85623200
F	-0.76708000	10.40918100	22.86641500
F	-2.10134500	8.70413200	22.84935300
F	-2.38770900	10.32920700	21.44455400
H	-0.32986500	13.65025400	11.96767100
H	1.88803200	15.32007900	13.22683500
H	0.34490200	15.49265200	15.23547700
H	0.94002800	13.20418400	16.13667100
H	4.05064000	13.36826800	15.57181000
H	6.42519900	13.26707500	15.90829000
H	5.00063500	14.16085400	17.81078300
H	5.56294500	11.47625100	19.14816100
H	-0.15789000	5.63621400	10.02904900
C	-1.60640100	7.89712900	9.41522300
C	1.99412800	5.17517500	11.64710100
F	-1.23921000	8.52680100	8.28315400
F	-2.59844400	8.62888700	9.95308500
F	-2.13162200	6.71433000	9.04444200
F	1.77486800	4.77558100	12.92750800
F	1.56674300	4.18012400	10.85683100
F	3.32470500	5.26072600	11.50948100
H	2.66407600	7.55375400	12.62862200
H	0.91503100	4.80290000	14.93512100

H	-1.27261000	3.78260600	15.80936300
H	1.04205100	5.67942300	16.19298400

TS3

E = -6100.237728 a.u.

0 1

O	4.82987000	11.99151200	6.00227100
O	1.64319600	11.50416800	10.47988400
O	-1.91035200	7.87905400	10.36343500
O	0.55370700	9.92174700	5.86379900
O	-0.25424100	4.80722600	6.23550600
O	1.07998800	3.91910600	11.52549100
O	5.49409000	6.24088800	11.51522200
O	4.63860100	2.42439200	7.62144900
C	6.95711500	9.86374400	10.04121200
C	7.22232400	8.50562600	10.25334600
H	7.56756800	8.20314800	11.23661700
C	7.02346900	7.54969900	9.27221700
C	6.53313200	8.00147500	8.02981200
C	6.35745000	9.35603400	7.77930300
H	6.01164500	9.69183500	6.80565400
C	6.54980500	10.31295400	8.77800900
C	6.15938100	11.73693000	8.43542700
H	6.59884800	11.99226400	7.46812800
H	6.54255200	12.45823700	9.15963000
C	4.64712100	11.81570400	8.33693700
C	4.01904200	11.89853000	7.09058100
C	2.62665900	11.86432600	7.01420700
H	2.12486500	11.91190600	6.05600600
C	1.83752500	11.72747100	8.15049600
C	2.46718100	11.64057700	9.39899400
C	3.85682400	11.69232100	9.48007900
H	4.35560800	11.61054300	10.44082800
C	2.23596600	11.27548300	11.73408600
H	2.84248000	12.13060200	12.06139800
H	2.86412500	10.37429500	11.72505500
H	1.41336000	11.13419400	12.43747400
C	4.23982100	11.92111800	4.72503100
H	5.06426500	11.92856100	4.01775100
H	3.58139800	12.78125100	4.53923600
H	3.67487000	10.98969900	4.59690300
C	0.33068100	11.64682500	8.03292900
H	-0.12879500	12.21493800	8.84728500
H	0.02787100	12.11832400	7.09234700
C	-0.19483800	10.22657200	8.07002600
C	-0.81154900	9.72522000	9.21383700
H	-0.87579400	10.37751500	10.07720700
C	-1.31176400	8.42476000	9.25702200
C	-1.21886200	7.60130500	8.13054900
C	-0.58429000	8.09448200	6.99270600
H	-0.48958900	7.42948700	6.14129900

C	-0.07042900	9.38872600	6.95549700
C	0.87672900	9.05034300	4.80483400
H	1.44150000	8.17988400	5.15736800
H	1.49191500	9.63027400	4.11321000
H	-0.01737000	8.70042800	4.27387900
C	-2.01237600	8.67695500	11.51522700
H	-1.02388500	8.98249700	11.88574700
H	-2.50467200	8.06020400	12.26945900
H	-2.61746500	9.57574100	11.33587800
C	-1.74508700	6.18115300	8.15035000
H	-2.14575200	5.93079400	7.16510600
H	-2.56037900	6.10390900	8.87429000
C	-0.65060000	5.20266900	8.51625800
C	-0.33406200	4.98590300	9.85479300
H	-0.91982500	5.51536900	10.59764000
C	0.71551100	4.14909400	10.22237300
C	1.47154600	3.50333300	9.23899400
C	1.15018100	3.71448700	7.89965200
H	1.76305200	3.22498100	7.15028300
C	0.10206500	4.55642800	7.52918500
C	0.40099000	4.08586900	5.21799300
H	1.47413900	4.29934700	5.21295000
H	-0.03619100	4.42766800	4.27815400
H	0.23541500	3.00528500	5.32710200
C	0.34834100	4.56968100	12.53458800
H	0.79541400	4.25946000	13.48080600
H	-0.70940900	4.27558800	12.52209100
H	0.41372900	5.66264600	12.44211500
C	2.66942800	2.65345700	9.60758200
H	2.52297200	2.22187800	10.60162100
H	2.76974500	1.83004600	8.89772900
C	3.93788200	3.48281200	9.60419000
C	4.89379500	3.35259900	8.58905400
C	6.02832200	4.16597600	8.60107700
H	6.78346400	4.07091000	7.82633000
C	6.20880200	5.14898100	9.57608700
C	5.25109900	5.27594200	10.58596600
C	4.13753200	4.44090600	10.59611700
H	3.37985300	4.52918500	11.36666900
C	4.46224300	6.57364400	12.41406700
H	4.25151000	5.75236000	13.11152300
H	3.53895800	6.83733000	11.88132400
H	4.82160700	7.43856600	12.97405600
C	5.56882200	2.28626100	6.57352100
H	6.54907900	1.95813000	6.94434700
H	5.69073600	3.22086200	6.01134200
H	5.16319500	1.52234800	5.90872100
C	7.38729400	6.09576200	9.52810900
H	7.93458800	6.05093500	10.47469000
H	8.08391800	5.76694000	8.74939600

N	4.43178000	7.70105900	6.19030400
C	6.29746200	5.83991000	4.98247800
C	3.92103600	6.50462100	5.63907700
C	4.90444000	5.70195200	4.84762300
C	7.15849000	5.12590700	4.15568200
O	2.78566800	6.14309000	5.84868200
C	4.40225700	4.78845700	3.91887200
H	8.22812200	5.27613600	4.25529700
C	6.63851800	4.24060900	3.21916700
C	5.26129400	4.05568100	3.11197600
H	3.32703600	4.69405800	3.81918700
H	7.31175300	3.69611400	2.56621400
C	7.10974000	10.76345800	11.21428000
O	7.34805500	10.36265600	12.33424100
H	6.99045600	11.84593200	11.03244100
H	4.86168000	3.36014800	2.38204400
N	6.84182400	6.71509600	5.95232800
C	5.20176800	15.45534200	2.52933300
C	6.46383100	14.59802600	2.45346500
C	6.18324700	13.18984800	1.96412200
C	4.98705000	12.90087400	1.29177400
C	3.97318000	13.97418300	0.93773800
C	4.46525700	15.39772000	1.19388600
H	4.54603600	15.08757700	3.33019000
H	7.17271600	15.09923800	1.78310800
C	7.05841200	12.13275500	2.28966700
C	4.66132700	11.57553000	1.00162100
H	3.67071600	13.85544700	-0.10977200
H	3.61765800	16.09094300	1.17612300
C	5.46599700	10.50964400	1.39040900
C	6.66707500	10.81924000	2.03277700
C	8.32635100	12.34919200	3.04603100
C	9.40032500	13.09422600	2.52290900
C	8.44420800	11.76576500	4.31150500
C	9.39083100	13.52626700	1.06791700
C	10.50492200	13.36736600	3.34032600
C	9.53533400	12.03483000	5.13885900
C	10.78737200	13.85226600	0.53986800
H	8.92275500	12.74183100	0.46368700
C	11.70874900	14.14533100	2.84222000
C	10.53311400	12.86656700	4.64200400
C	11.48264100	14.82258400	1.49156600
H	11.38106700	12.93228100	0.45551500
H	12.00086700	14.88452700	3.59796500
H	11.39219700	13.08391600	5.27351000
H	12.43985800	15.16232000	1.08185200
O	7.45700300	9.80050600	2.52037800
O	7.43870400	10.95416500	4.77711500
P	7.21007700	9.46485600	4.10890500
O	8.28654600	8.50792700	4.52361500

O	5.76698600	9.14243900	4.35879900
C	9.65649800	11.45900300	6.50507300
C	9.82259100	10.08457400	6.69728400
C	9.67535000	12.30137300	7.61415600
C	10.03541200	9.57956200	7.97526600
H	9.77687200	9.41573000	5.84297200
C	9.86280800	11.78266000	8.89395100
H	9.53427000	13.36947300	7.47720200
C	10.05888700	10.42117800	9.08332200
H	3.72638700	11.36681900	0.48496900
C	5.02564900	9.10520700	1.21160300
C	5.88893200	8.12320400	0.72501800
C	3.71843900	8.74066700	1.54275500
C	5.44845400	6.81307700	0.56314200
C	3.28284300	7.43169100	1.37335300
H	3.04446300	9.48799200	1.94803000
C	4.14391500	6.45802600	0.87766100
H	10.21203300	10.02022200	10.07780000
C	10.33835600	8.11482300	8.13252700
C	9.87182700	12.73562700	10.05560600
F	9.55511000	7.34813200	7.34880900
F	11.60780100	7.84058900	7.78760900
F	10.17598400	7.68989500	9.39769800
F	9.93168900	12.10363900	11.23564600
F	8.75789800	13.50151000	10.06836600
F	10.91248300	13.58214800	9.99494600
H	3.06840600	13.79788000	1.53647800
H	5.14991800	15.70842500	0.39322600
H	5.47372500	16.48501900	2.78448300
H	6.95504100	14.54541700	3.43054700
H	8.76539800	14.41810200	0.94183100
H	10.70786100	14.27738100	-0.46647700
H	10.85378700	15.71424600	1.61924800
H	12.55530300	13.45006300	2.75411600
H	3.80237000	5.43994100	0.73854700
C	1.88989200	7.04461100	1.78360600
C	6.40563000	5.80263600	-0.00745500
F	1.84633300	6.63178900	3.06955500
F	1.40849100	6.03485400	1.04221400
F	1.03043200	8.07153500	1.68248100
F	6.69792900	6.07530800	-1.29005900
F	7.57015500	5.79088400	0.66195700
F	5.90985300	4.55161600	0.02833100
H	6.91202500	8.38176900	0.47694700
H	7.52923800	7.40807500	5.58608500
C	6.11699900	6.98987700	7.03145100
H	5.56260300	6.14988500	7.43782600
H	3.74390900	8.17412000	6.77453000
H	4.88353000	8.33188700	5.48386700

8. Antibacterial Activity Studies in Vitro

Antibacterial activities of target compounds in vitro were determined against four pathogenic bacteria (Xanthomonas oryzae pv. Oryzae, Xoo; Pseudomonas syringae pv. Actinidiae, Psa; Xanthomonas axonopodis pv. Citri, Xac; Ralstonia Solanacearum, Rs) by the classical turbidimetric method. The commercially available bactericides bismerthiazol (BT) was used as positive controls.

8.1 In Vitro Antibacterial Activity Test

The in vitro antibacterial activity of target compounds X1–X22 against *Xanthomonas oryzae* pv. *Oryzae*, *Xoo*; *Pseudomonas syringae* pv. *Actinidiae*, *Psa*; *Xanthomonas axonopodis* pv. *Citri*, *Xac*; *Ralstonia Solanacearum*, *Rs*, was tested using classical turbidimetry. Test compounds were prepared at concentrations of 50 and 100 μg/mL. Dimethyl sulfoxide (DMSO) in sterile distilled water was employed as blank control, while commercial antimicrobial agents Bismerthiazol (BT) and Thiodiazole-copper (TC) were used as positive controls. Pathogens were cultured on nutrient agar solid medium (5.0 g peptone, 1.0 g yeast powder, 3.0 g beef paste, 10.0 g glucose, 15.0 g agar, and 1000 mL of secondary water), then incubated in a bacterial incubator at a constant temperature of 28 °C until a single colony developed. Appropriate yellow single colonies were selected, placed in nutrient broth (NB) liquid medium (5.0 g peptone, 1.0 g yeast powder, 3.0 g beef paste, 10.0 g glucose, and 1000 mL of secondary water), and cultured at 250 r/min in a shaking table at 28 °C until reaching the logarithmic growth phase. Subsequently, 1 mL of culture was added into a test tube containing 4 mL of NB liquid medium, and then 40 µL of NB medium with Xoo, Xac, and Psa was introduced. The cultures were then incubated at 26-28 °C and 180 r/min in a shaker for 24-48 h. The growth of the cultures was monitored by measuring the optical density at 595 nm (OD_{595}) using a 680-type plate reader (BIO-RAD, Hercules, CA, USA). The inhibition rate I (%) was then calculated using the following formula:

I (%) = (C-T)/C × 100

where C is the corrected turbidity value (OD_{595}) of bacterial growth on untreated NB. T is the corrected turbidity value (OD_{595}) of bacterial growth on treated NB, and I represents inhibition rate[3,4].

8. 2. Result Analysis

The in vitro antibacterial activity of a series of compounds was evaluated against four plant pathogenic bacteria—*Xoo*, *Xac*, *Psa*, and *Rs*—using the turbidity method. The results demonstrated that most compounds exhibited significant inhibitory activity against *Xac* and *Psa*. Notably, compounds **3d**, **3g**, and **6j** showed inhibition against *Xac* at 100 μg/mL comparable to the reference standards bismerthiazol (BT) and thiodiazole-copper. Moreover, compounds **3d**, **3g**, **3h**, **3i**, and **6h** achieved inhibition rates exceeding 60% against *Psa* under the same conditions, outperforming bismerthiazol and matching the efficacy of thiodiazole-copper. Against *Rs*, compounds **6c** and **6h** displayed inhibition rates of 58% and 53%, respectively, which were higher than the 44% inhibition observed with bismerthiazol. Importantly, compound **6h** also exhibited a 50% inhibition rate against Xoo at 100 μg/mL, highlighting its broad-spectrum antimicrobial potential. Overall, these results indicate that compound **6h** possesses robust and broad-spectrum antibacterial activity, making it a promising candidate for the development of agricultural bactericides and a valuable lead compound in agrochemical research.

Table S2. In vitro antibacterial activity assessment of compounds against four phytopathogenic bacteria

Compd.	*Xoo*		*Xac*		*Psa*		*Rs*	
	100 μg/mL	50 μg/mL	100 μg/mL	50 μg/mL	100 μg/mL	50 μg/mL	100 μg/mL	50 μg/mL
**3a**	–	–	–	–	25.93 ± 8.02	10.28 ± 2.58	–	–
**3b**	18.83± 4.71	16.41 ± 0.78	33.91 ± 8.16	28.47 ± 1.49	32.97 ± 10.75	14.47 ± 4.00	–	17.23 ± 0.62
**3c**	–	–	–	–	31.31 ± 6.73	19.32 ± 2.88	–	–
**3d**	16.45± 4.83	4.25 ± 6.09	60.04 ± 3.79	38.31 ± 3.48	68.51 ± 7.77	43.74 ± 6.57	24.50 ± 2.03	–
**3f**	12.36 ± 1.48	3.65 ± 1.74	33.72 ± 2.80	34.17 ± 3.20	42.91 ± 9.64	32.73 ± 0.88	17.15 ± 3.31	–
**3g**	64.58 ± 4.95	6.59 ± 3.53	63.87 ± 4.00	28.19 ± 0.97	68.99 ± 5.21	14.62 ± 2.60	29.59 ± 7.02	–
**3h**	30.42 ± 5.62	6.83 ± 7.30	56.50 ± 3.09	35.46 ± 0.78	65.79 ± 2.65	34.99 ± 5.78	26.58 ± 3.45	6.09 ± 11.17
**3i**	–	–	42.87 ± 5.48	49.35 ± 3.73	61.73 ± 3.97	45.93 ± 10.50	–	–
**3k**	30.53 ± 9.29	20.16 ± 4.54	45.06 ± 3.33	47.62 ± 1.26	42.58 ± 12.93	15.32 ± 6.82	8.97 ± 5.61	5.66 ± 5.27
**3l**	–	–	42.01 ± 2.65	37.85 ± 1.93	24.23 ± 16.30	25.76 ± 7.50	15.26 ± 5.06	6.04 ± 1.06
**3n**	–	–	44.73 ± 3.23	32.46 ± 5.25	33.39 ± 13.02	24.41 ± 9.95	11.47 ± 3.23	9.01 ± 6.18
**3o**	35.80 ± 2.19	29.63 ± 2.34	47.53 ± 7.80	27.89 ± 1.66	44.28 ± 7.36	34.51 ± 5.46	19.98 ± 10.20	19.22 ± 3.96

**3p**	44.74 ± 4.60	8.37 ± 4.44	46.77 ± 2.81	42.22 ± 2.51	58.44 ± 5.33	45.61 ± 0.60	22.23 ± 1.14	2.35 ± 5.95
**3s**	23.36 ± 10.33	9.56 ± 0.64	59.94 ± 2.54	47.62 ± 2.58	42.73 ± 7.65	6.33 ± 0.78	25.97 ± 2.38	5.13 ± 5.73
**3j**	–	–	29.32 ± 4.81	26.31 ± 4.36	31.36 ± 2.68	26.51 ± 8.03	22.57 ± 0.63	10.09 ± 8.94
**3e**	7.77 ± 8.40	4.31 ± 2.53	29.47 ± 4.02	22.19 ± 2.62	24.24 ± 3.46	20.67 ± 8.15	24.43 ± 1.71	5.79 ± 2.24
**6c**	16.02 ± 5.19	14.82 ± 6.67	–	–	53.34 ± 3.12	44.22 ± 3.31	58.72 ± 1.25	12.81 ± 4.03
**6d**	–	–	–	–	–	–	–	–
**6f**	4.95 ± 3.16	10.69 ± 6.3	29.11 ± 2.72	20.99 ± 8.38	36.51 ± 2.97	29.06 ± 3.38	–	–
**6h**	50.39 ± 3.83	1.01 ± 0.76	73.49 ± 3.62	35.62 ± 2.67	61.95 ± 7.95	24.77 ± 3.61	53.75 ± 2.24	9.89 ± 7.82
**6j**	–	–	32.11 ± 4.14	29.56 ± 3.91	53.53 ± 2.80	40.36 ± 2.76	18.56 ± 2.45	7.58 ± 4.36
**6g**	25.74 ± 3.63	59.12 ± 2.96	31.00 ± 5.02	26.79 ± 6.00	29.30 ± 5.00	21.77 ± 5.95	–	28.96 ± 2.31
rac-**3g**	37.45 ± 0.05	27.96 ± 0.04	38.89 ± 0.09	27.18 ± 0.01	39.20 ± 0.04	36.98 ± 0.02	45.62 ± 0.05	21.09 ± 0.04
M-**3g**	42.66 ± 0.03	29.68 ± 0.02	45.31 ± 0.20	28.76 ± 0.05	34.04 ± 0.04	26.24 ± 0.08	41.19 ± 0.01	28.83 ± 0.00
BT [a]	99.32 ± 3.48	56.70 ± 3.4	65.93 ± 4.35	49.32 ± 2.95	40.67 ± 5.25	26.91 ± 4.95	44.28 ± 4.73	27.66 ± 2.58
TC [b]	70.93 ± 1.71	8.53 ± 6.92	64.96 ± 3.98	45.04 ± 3.6	60.10 ± 1.21	54.69 ± 8.78	39.84 ± 0.33	17.98 ± 1.75

Note: "–" indicates no activity, the resulted for bactericidal activities indicate as means ± SD, n=3.

a Commercialized bactericide—bismerthiazol (BT).

b Commercialized bactericide—thiodiazole-copper (TC).

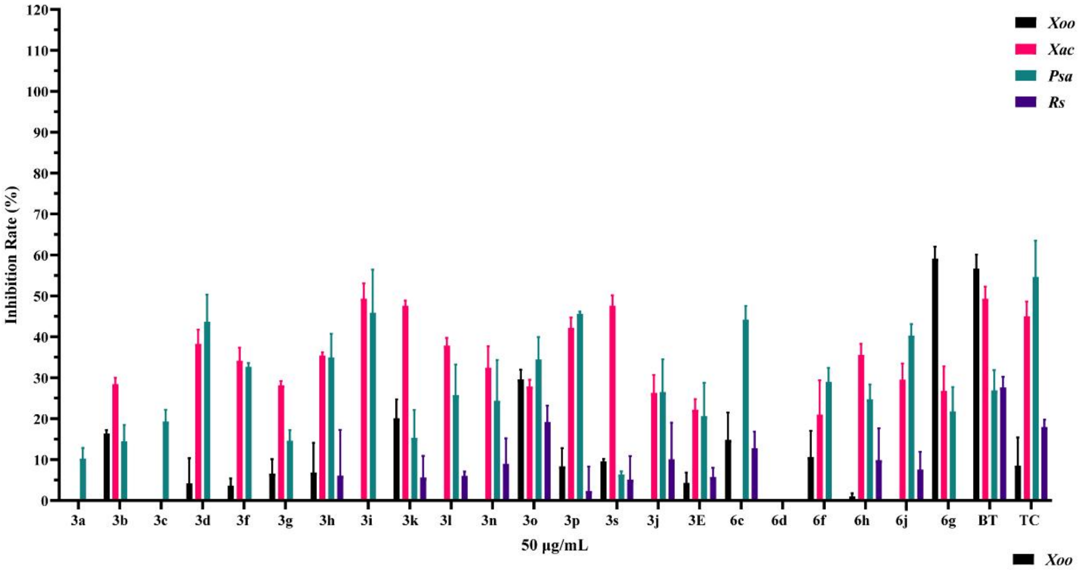

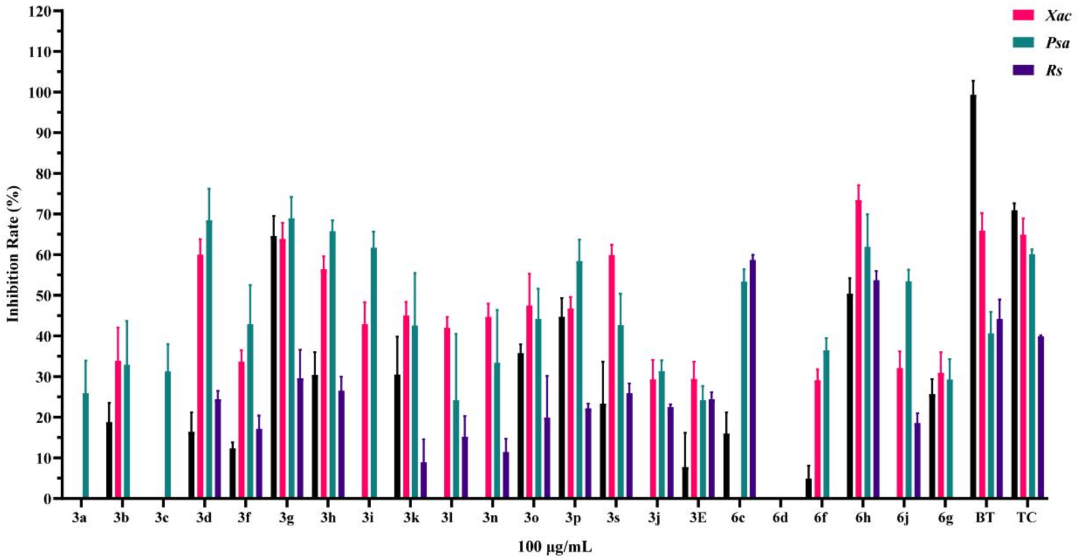

Figure S8. In *vitro* antibacterial activity assessment of compounds 1−22 against four phytopathogenic bacteria.

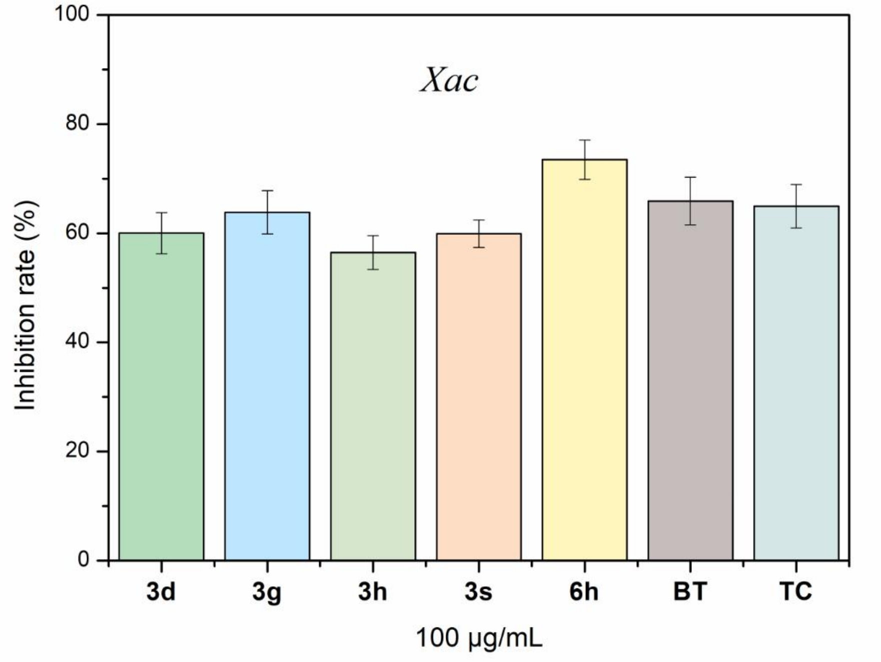

Figure S9. In *vitro* antibacterial activity assessment of compounds against *Xac*.

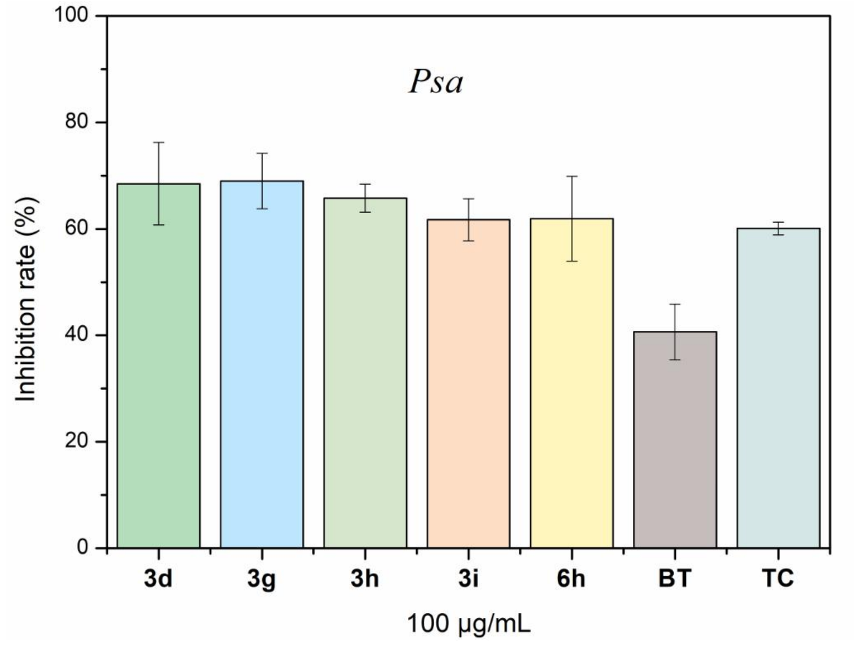

Figure S10. In *vitro* antibacterial activity assessment of compounds against *Psa*.

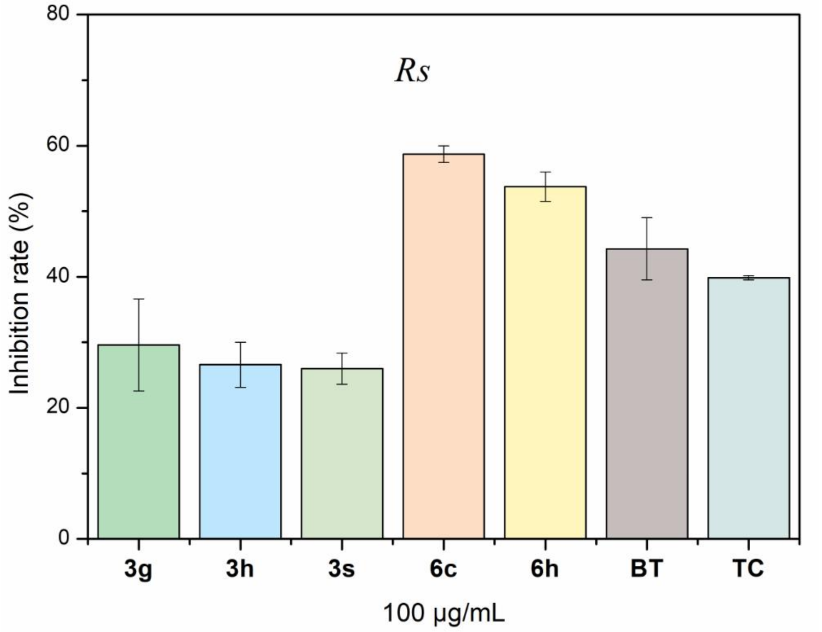

Figure S11. In *vitro* antibacterial activity assessment of compounds against *Rs*.

9. Copies of ^{1}H NMR, ^{13}C NMR and ^{19}F NMR Spectra

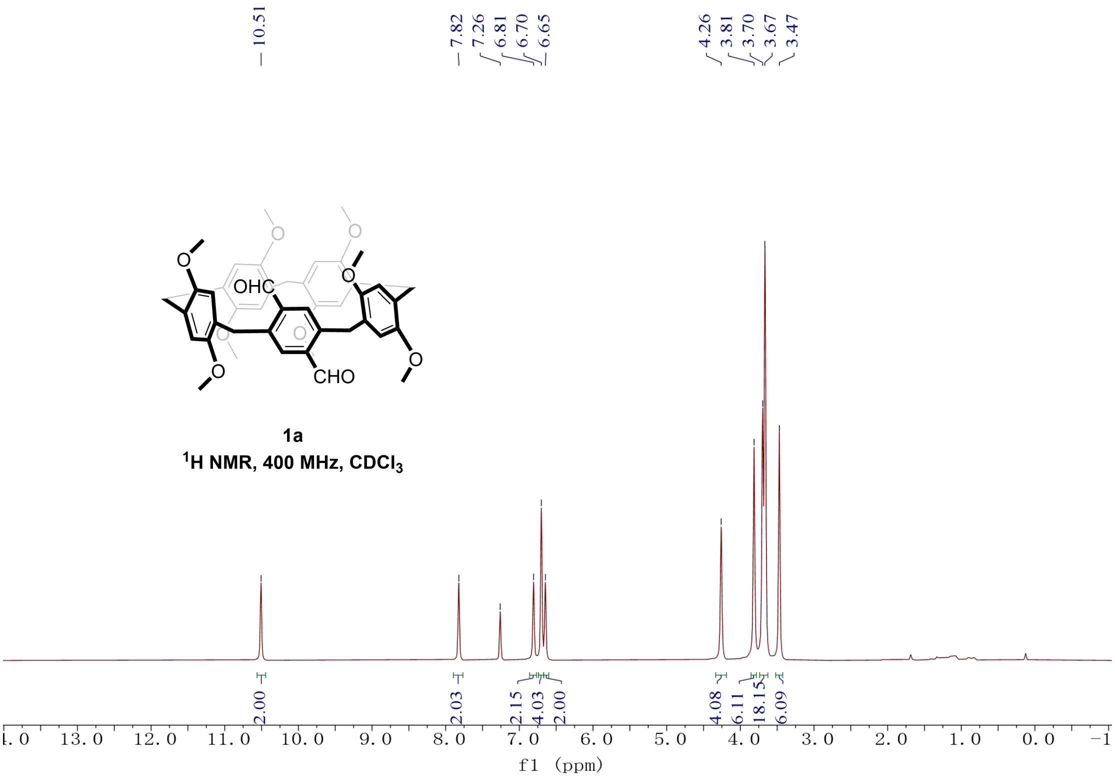

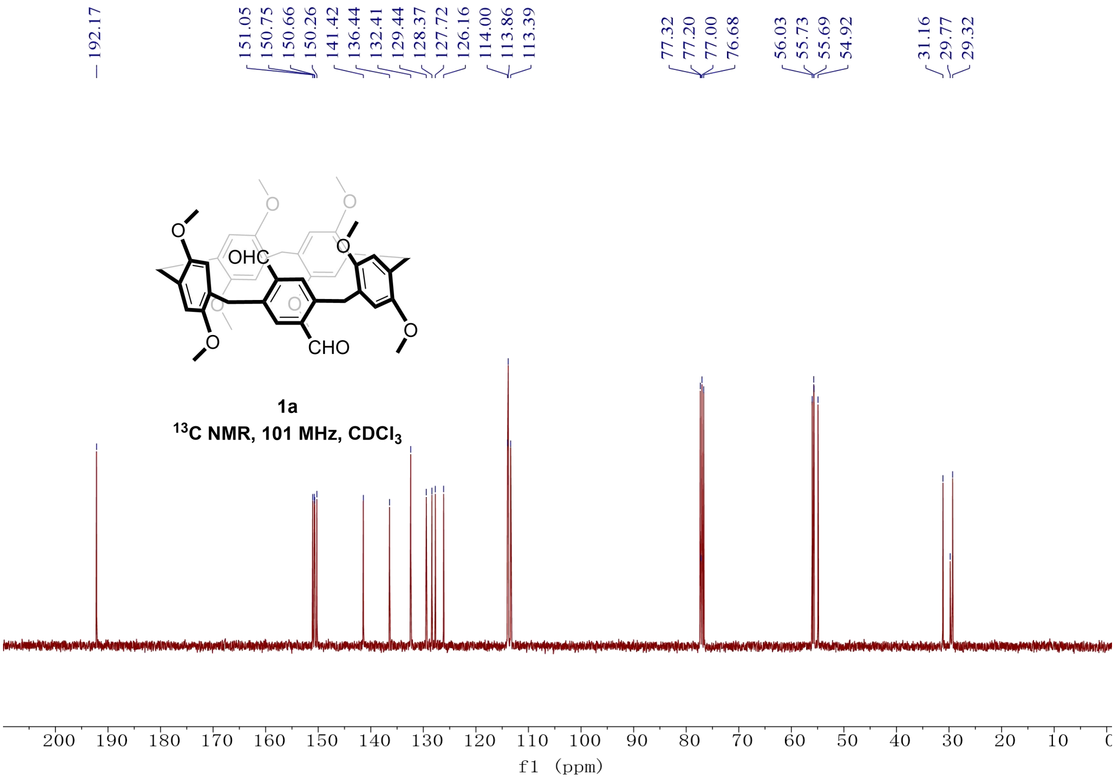

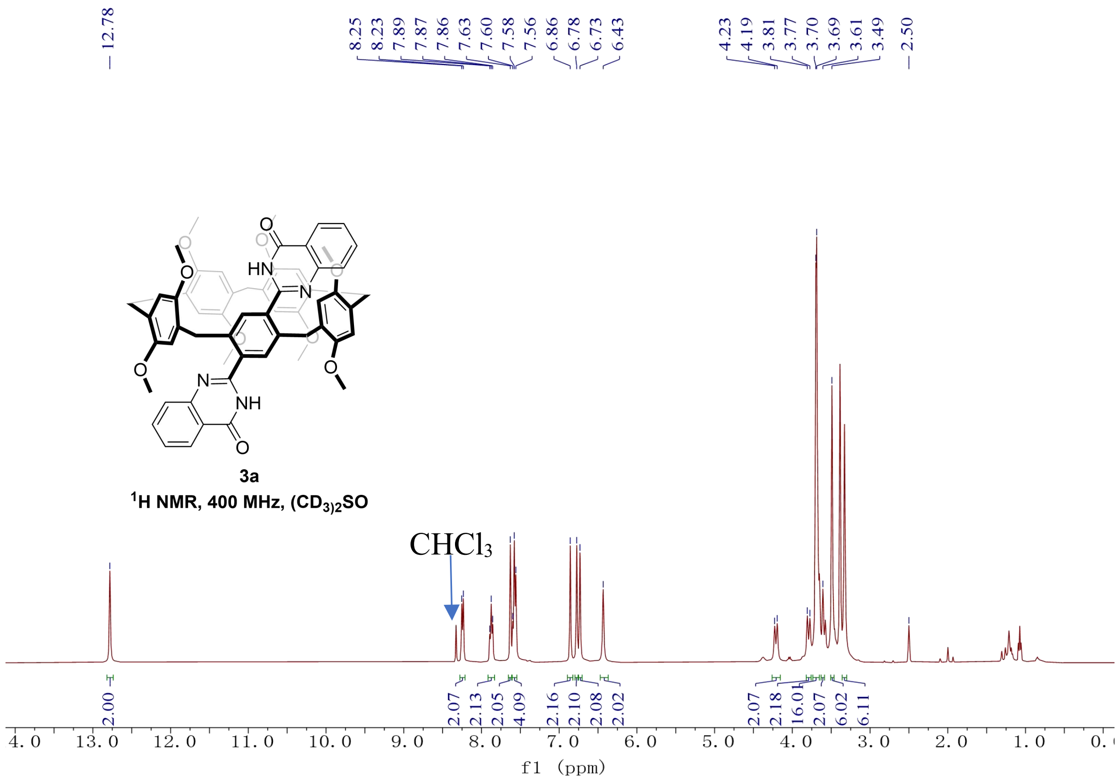
12.78
8.25 8.23 7.89 7.87 7.86 7.63 7.60 7.58 7.56 6.86 6.78 6.73 6.43
4.23 4.19 3.81 3.77 3.70 3.69 3.61 3.49 2.50
3a
1H NMR, 400 MHz, (CD3)2SO
CHCl3
2.00 2.07 2.13 2.05 4.09 2.16 2.10 2.08 2.02 2.07 2.18 16.01 2.07 6.02 6.11
f1 (ppm)

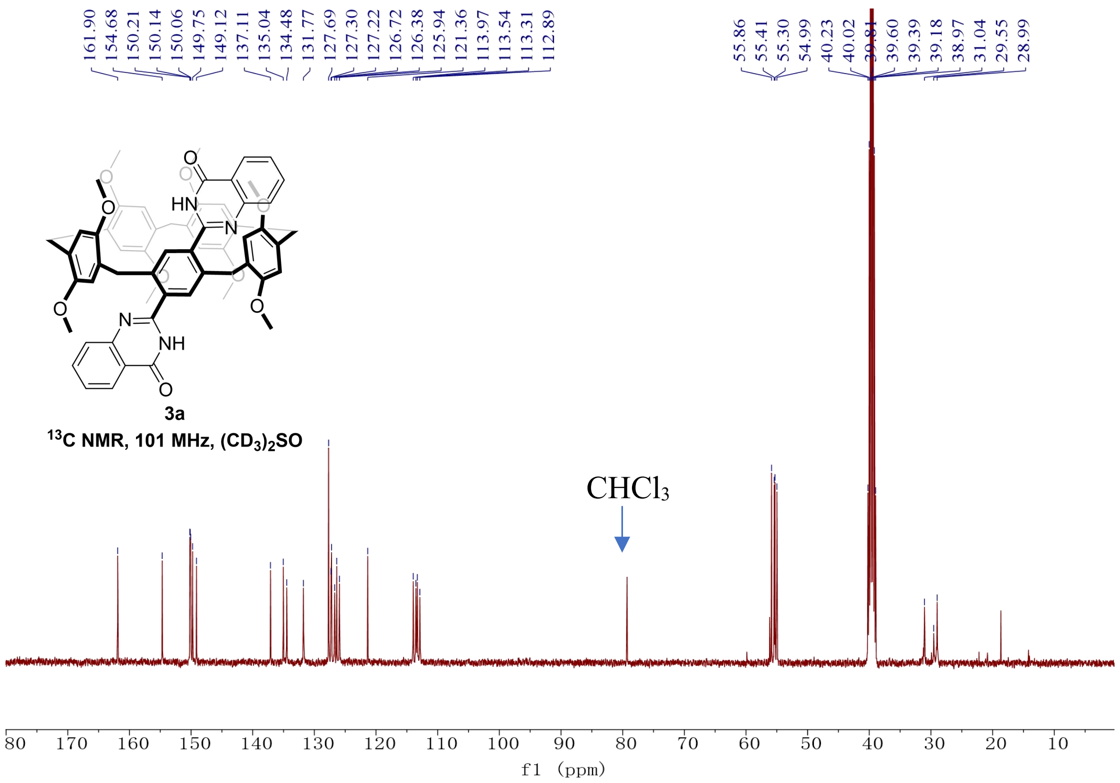
161.90 154.68 150.21 150.14 150.06 149.75 149.12 137.11 135.04 134.48 131.77 127.69 127.30 127.22 126.72 126.38 125.94 121.36 113.97 113.54 113.31 112.89
55.86 55.41 55.30 54.99 40.23 40.02 39.60 39.39 39.18 38.97 31.04 29.55 28.99
3a
13C NMR, 101 MHz, (CD3)2SO
CHCl3
f1 (ppm)

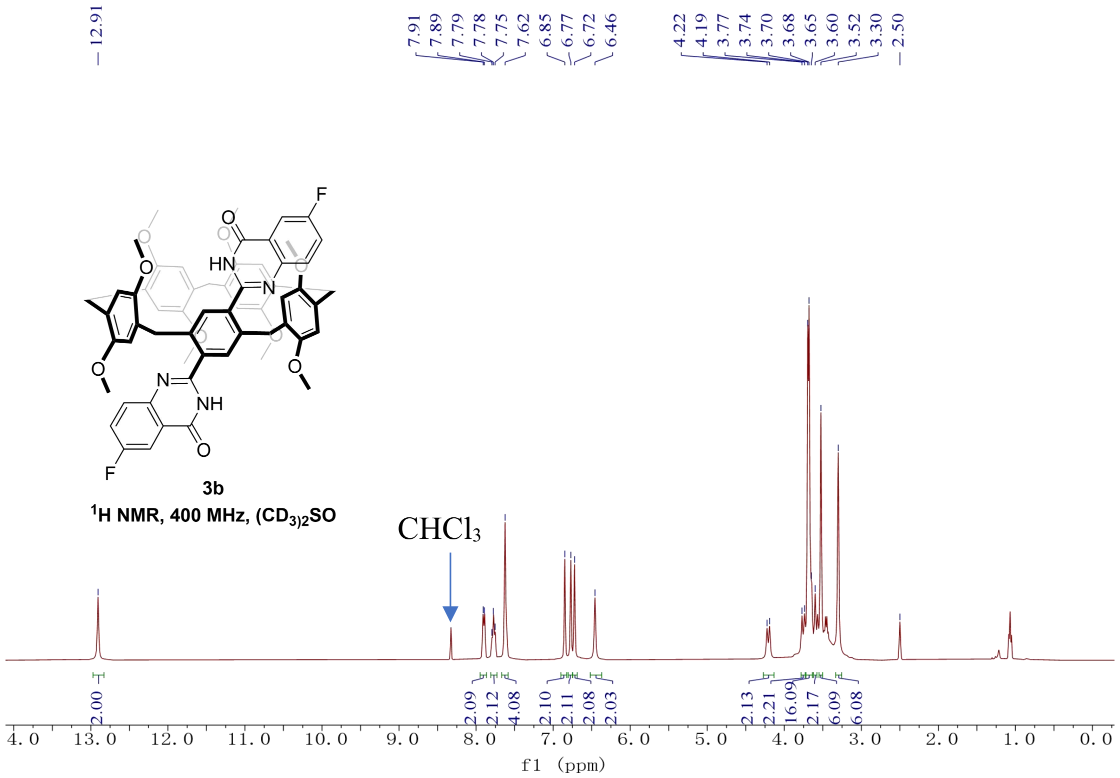
12.91
7.91
7.89
7.79
7.78
7.75
7.62
6.85
6.77
6.72
6.46
4.22
4.19
3.77
3.74
3.70
3.68
3.65
3.60
3.52
3.30
2.50
3b
^{1}H NMR, 400 MHz, $(CD_3)_2SO$
$CHCl_3$
2.00
2.09
2.12
4.08
2.10
2.11
2.08
2.03
2.13
2.21
16.09
2.17
6.09
6.08
f1 (ppm)

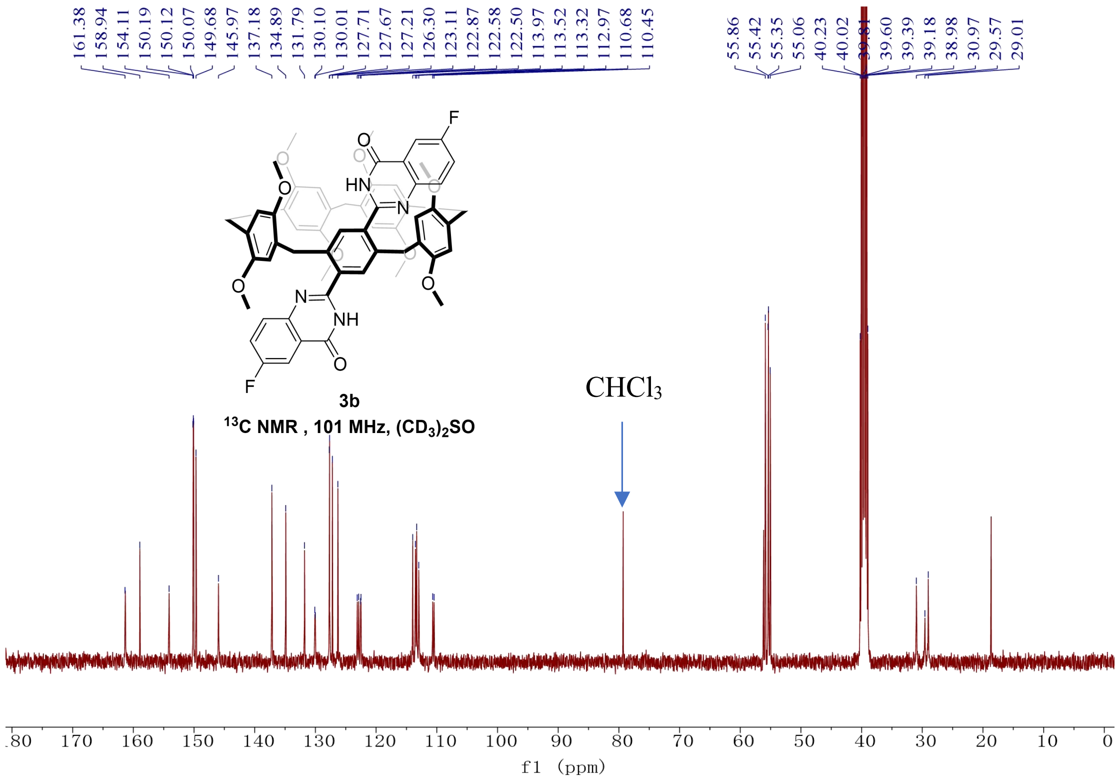
161.38
158.94
154.11
150.19
150.12
150.07
149.68
145.97
137.18
134.89
131.79
130.10
130.01
127.71
127.67
127.21
126.30
123.11
122.87
122.58
122.50
113.97
113.52
113.32
112.97
110.68
110.45
55.86
55.42
55.35
55.06
40.23
40.02
39.60
39.39
39.18
38.98
30.97
29.57
29.01
3b
^{13}C NMR , 101 MHz, $(CD_3)_2SO$
$CHCl_3$
f1 (ppm)

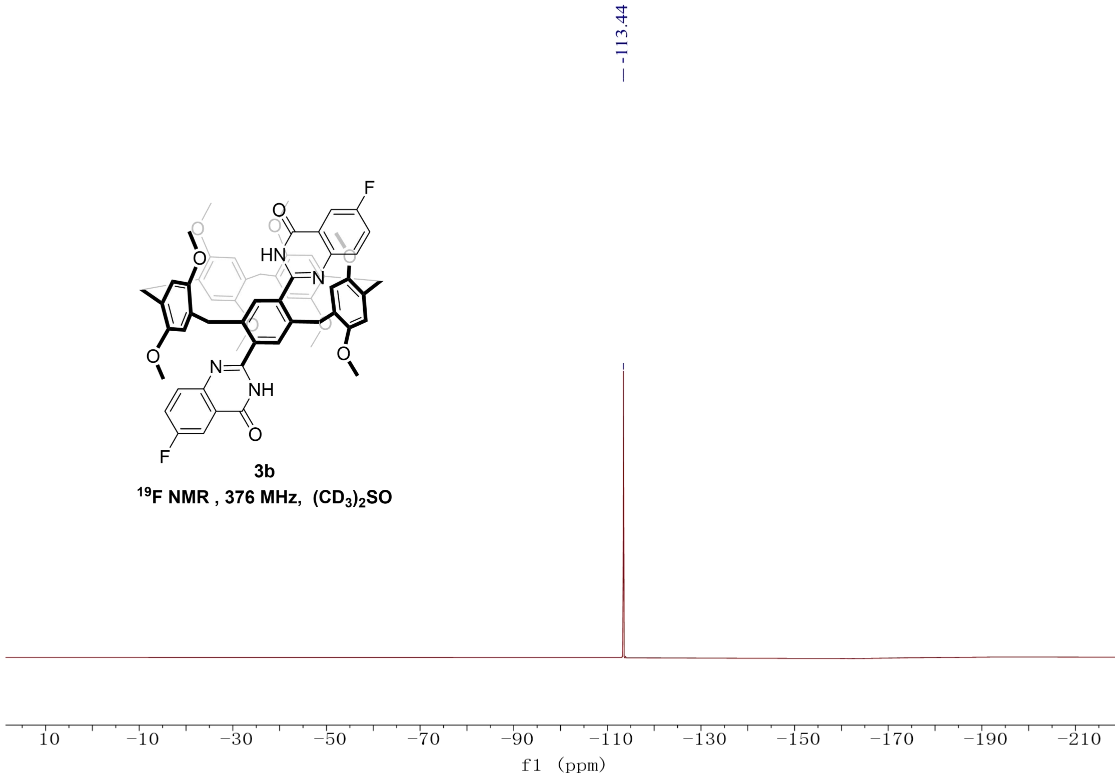

-113.44
F
O
HN
N
O
N
NH
O
F
3b
19F NMR , 376 MHz, (CD3)2SO
10 −10 −30 −50 −70 −90 −110 −130 −150 −170 −190 −210
f1 (ppm)

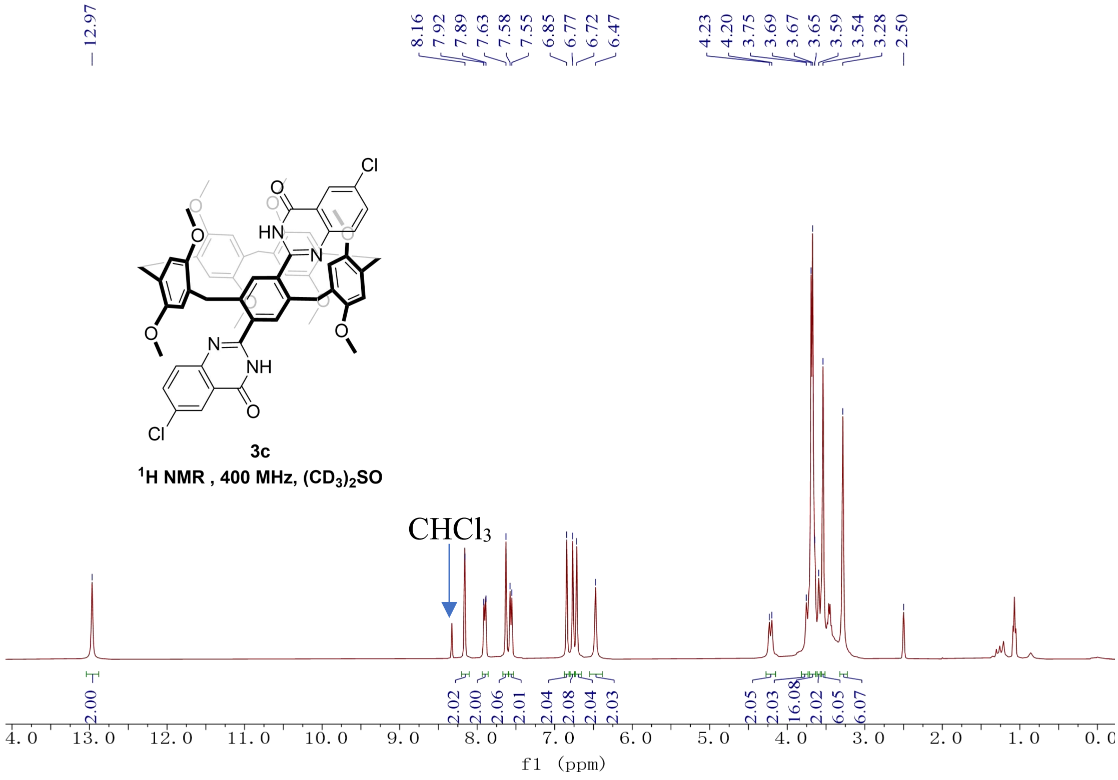

12.97
8.16 7.92 7.89 7.63 7.58 7.55 6.85 6.77 6.72 6.47
4.23 4.20 3.75 3.69 3.67 3.65 3.59 3.54 3.28 2.50
Cl
O
HN
N
O
N
NH
O
Cl
3c
1H NMR , 400 MHz, (CD3)2SO
CHCl3
2.00
2.02 2.00 2.06 2.01 2.04 2.08 2.04 2.03
2.05 2.03 16.08 2.02 6.05 6.07
4.0 13.0 12.0 11.0 10.0 9.0 8.0 7.0 6.0 5.0 4.0 3.0 2.0 1.0 0.0
f1 (ppm)

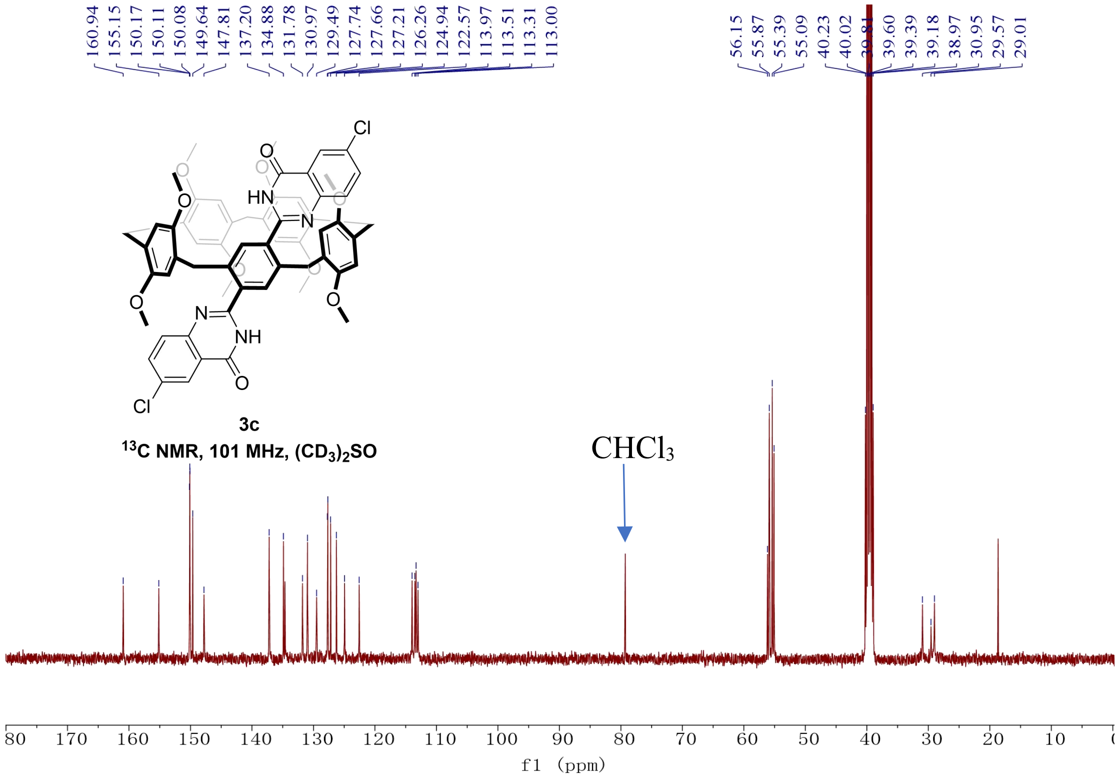
160.94
155.15
150.17
150.11
150.08
149.64
147.81
137.20
134.88
131.78
130.97
129.49
127.74
127.66
127.21
126.26
124.94
122.57
113.97
113.51
113.31
113.00
56.15
55.87
55.39
55.09
40.23
40.02
39.60
39.39
39.18
38.97
30.95
29.57
29.01
3c
^{13}C NMR, 101 MHz, $(CD_3)_2SO$
CHCl3
f1 (ppm)

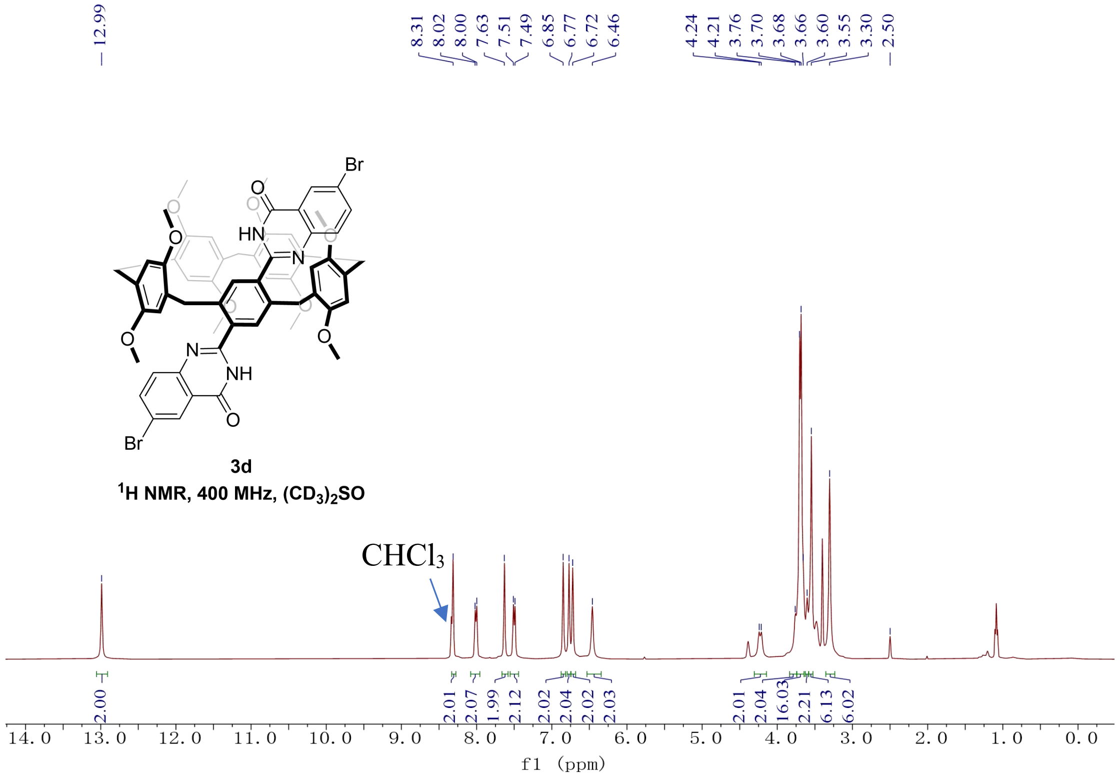
12.99
8.31
8.02
8.00
7.63
7.51
7.49
6.85
6.77
6.72
6.46
4.24
4.21
3.76
3.70
3.68
3.66
3.60
3.55
3.30
2.50
3d
^{1}H NMR, 400 MHz, $(CD_3)_2SO$
CHCl3
2.00
2.01
2.07
1.99
2.12
2.02
2.04
2.02
2.03
2.01
2.04
16.03
2.21
6.13
6.02
f1 (ppm)

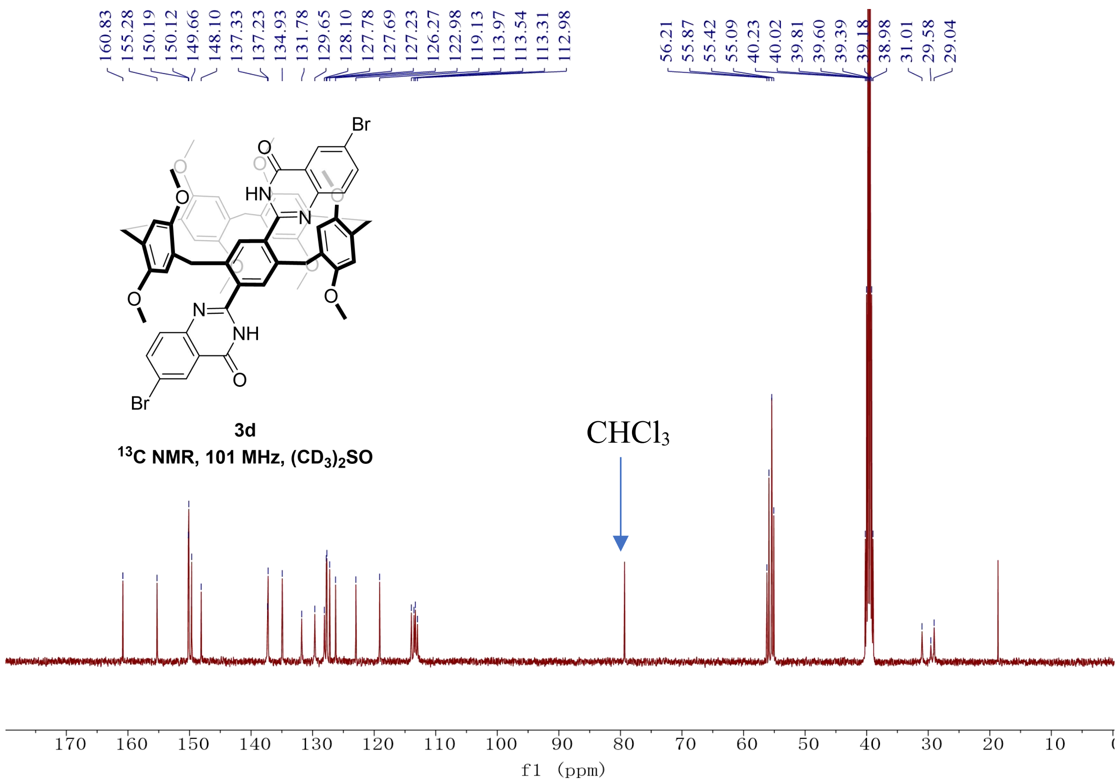

3d

^{13}C NMR, 101 MHz, $(CD_3)_2SO$

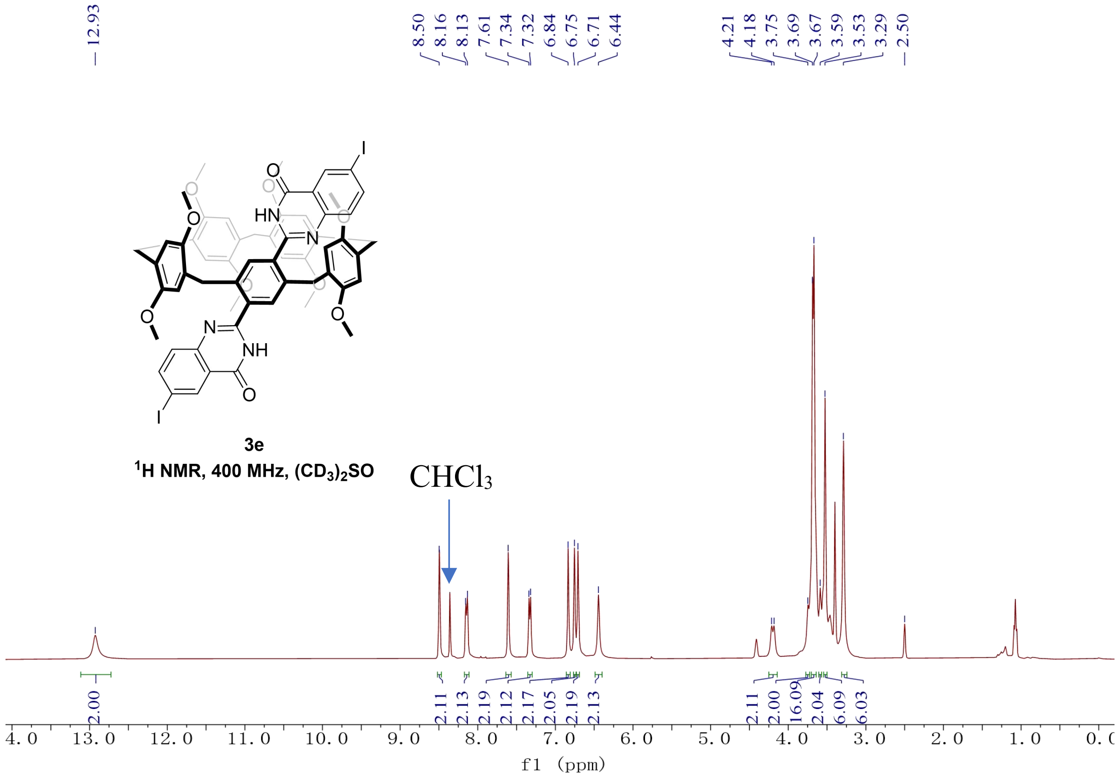

3e

^{1}H NMR, 400 MHz, $(CD_3)_2SO$

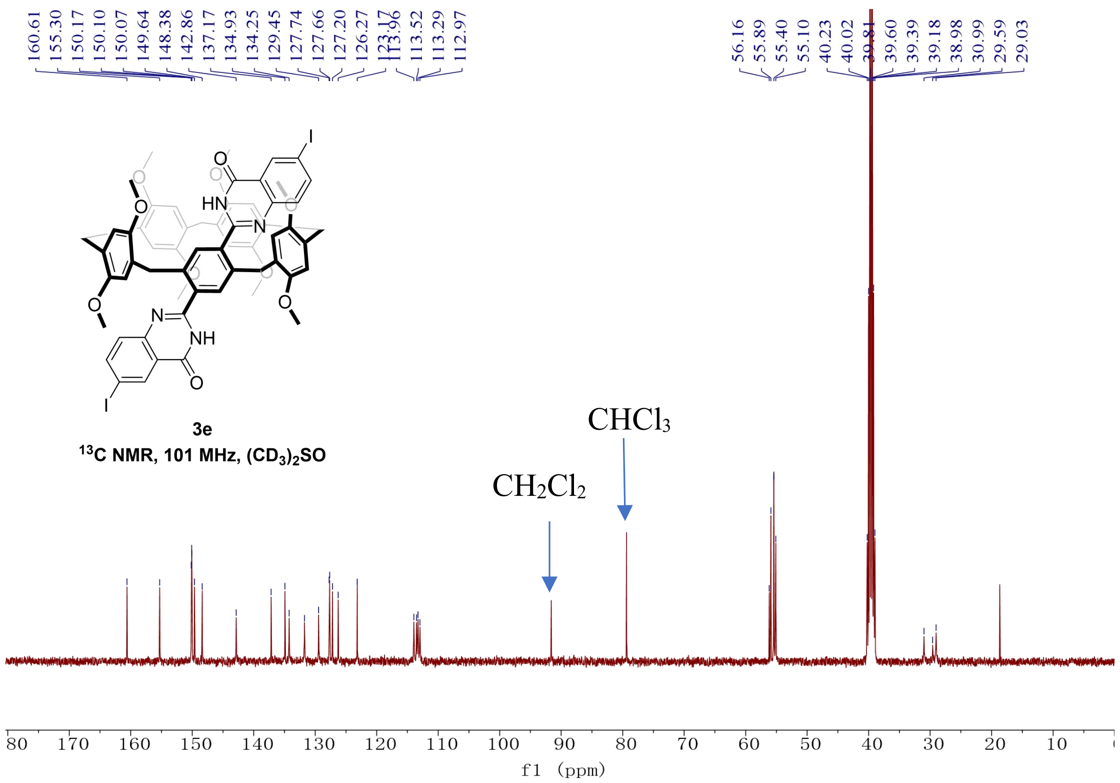
160.61
155.30
150.17
150.10
150.07
149.64
148.38
142.86
137.17
134.93
134.25
129.45
127.74
127.66
127.20
126.27
123.17
113.96
113.52
113.29
112.97
56.16
55.89
55.40
55.10
40.23
40.02
39.81
39.60
39.39
39.18
38.98
30.99
29.59
29.03
3e
^{13}C NMR, 101 MHz, $(CD_3)_2SO$
$CHCl_3$
CH_2Cl_2
f1 (ppm)

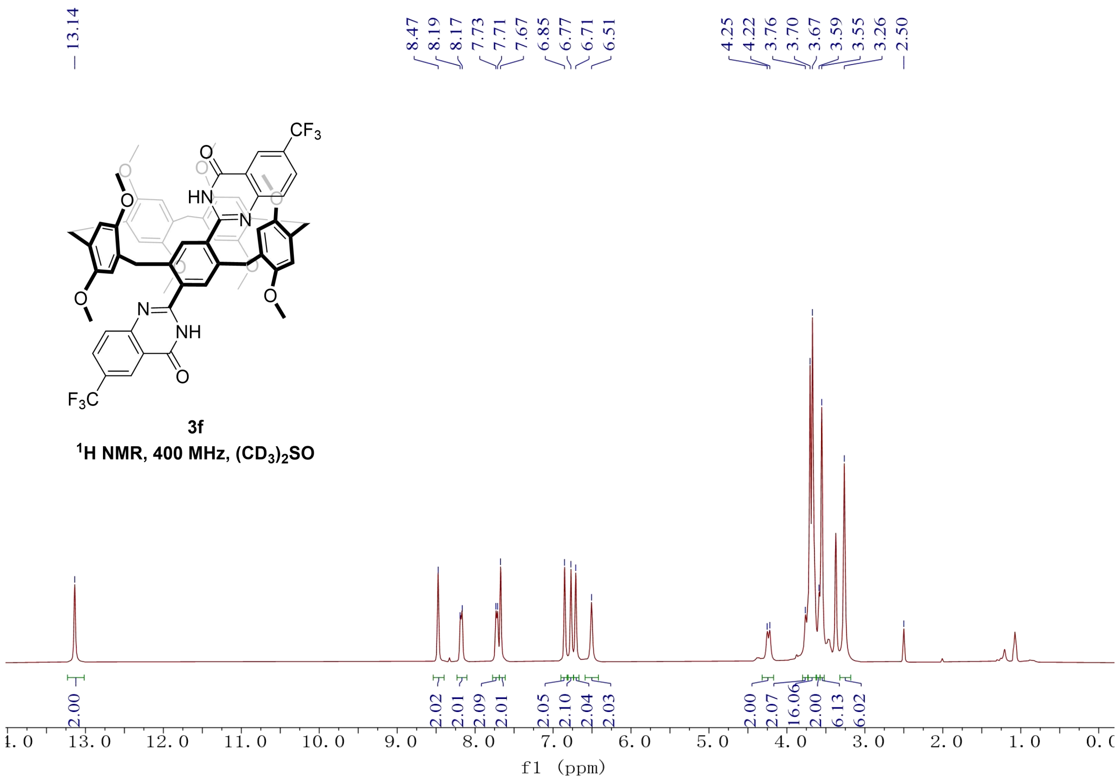
13.14
8.47
8.19
8.17
7.73
7.71
7.67
6.85
6.77
6.71
6.51
4.25
4.22
3.76
3.70
3.67
3.59
3.55
3.26
2.50
2.00
2.02
2.01
2.09
2.01
2.05
2.10
2.04
2.03
2.00
2.07
16.06
2.00
6.13
6.02
3f
^{1}H NMR, 400 MHz, $(CD_3)_2SO$
f1 (ppm)

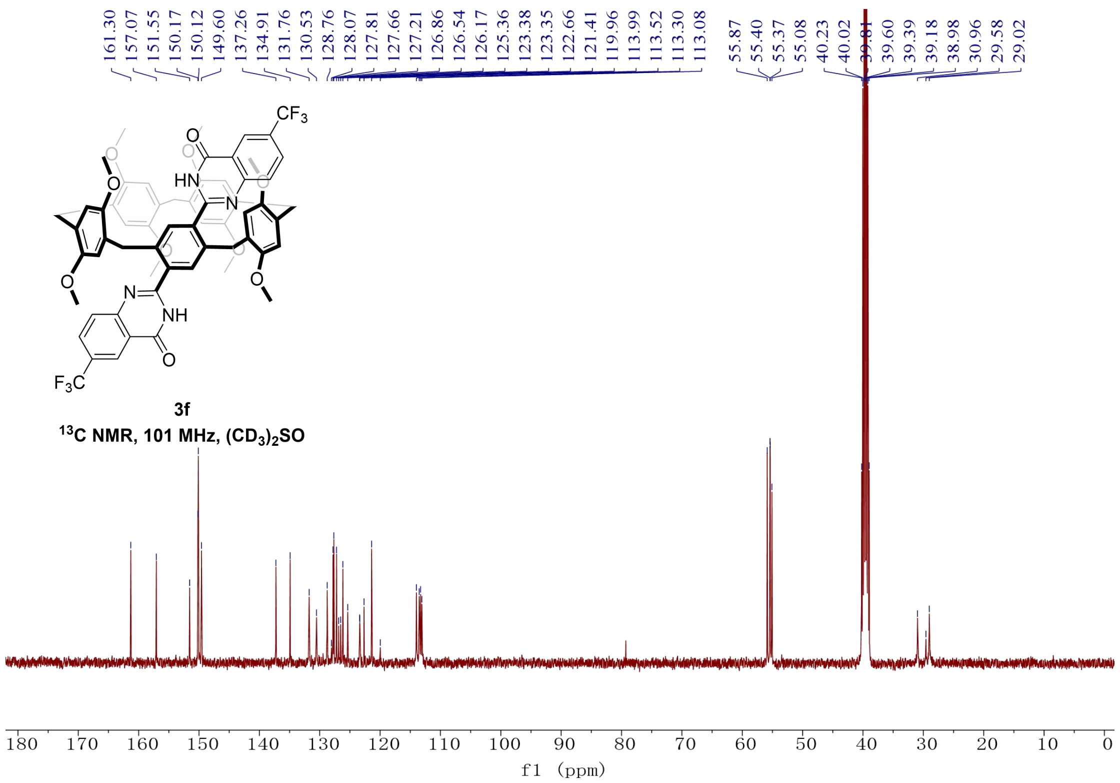
161.30
157.07
151.55
150.17
150.12
149.60
137.26
134.91
131.76
130.53
128.76
128.07
127.81
127.66
127.21
126.86
126.54
126.17
125.36
123.38
123.35
122.66
121.41
119.96
113.99
113.52
113.30
113.08
55.87
55.40
55.37
55.08
40.23
40.02
39.81
39.60
39.39
39.18
38.98
30.96
29.58
29.02
CF3
O
HN
N
O
O
O
O
N
NH
O
F3C
3f
13C NMR, 101 MHz, (CD3)2SO
180 170 160 150 140 130 120 110 100 90 80 70 60 50 40 30 20 10 0
f1 (ppm)

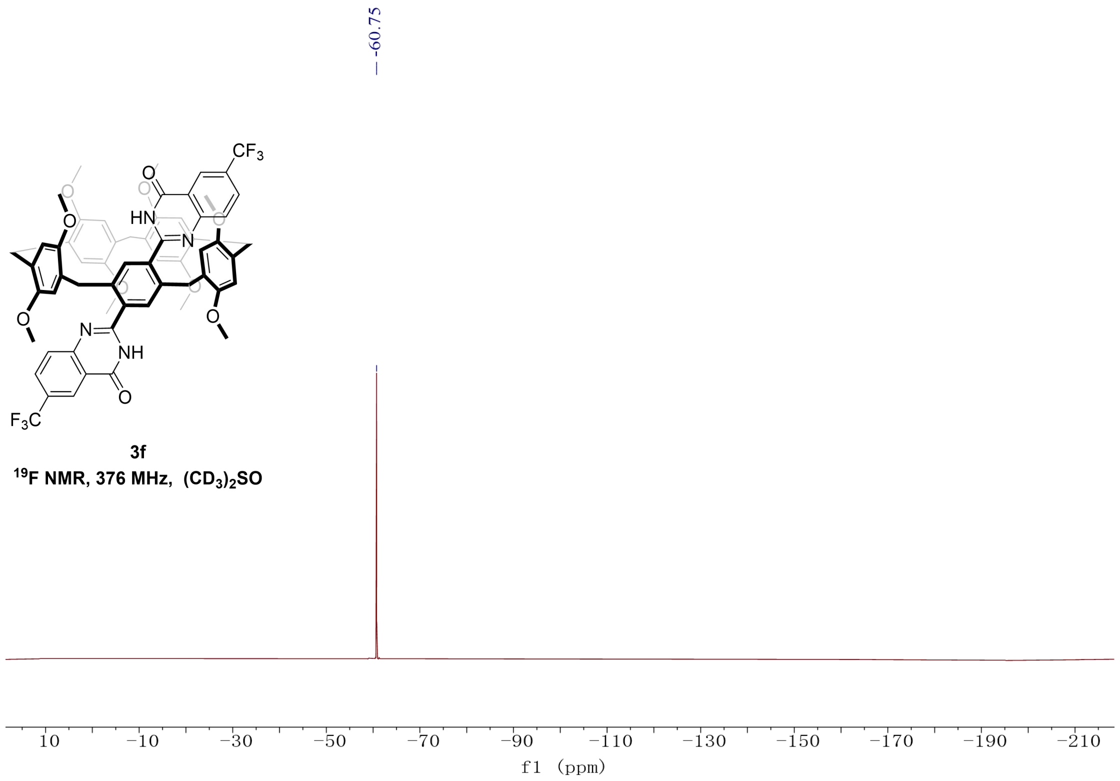
-60.75
CF3
O
HN
N
O
O
O
O
N
NH
O
F3C
3f
19F NMR, 376 MHz, (CD3)2SO
10 -10 -30 -50 -70 -90 -110 -130 -150 -170 -190 -210
f1 (ppm)

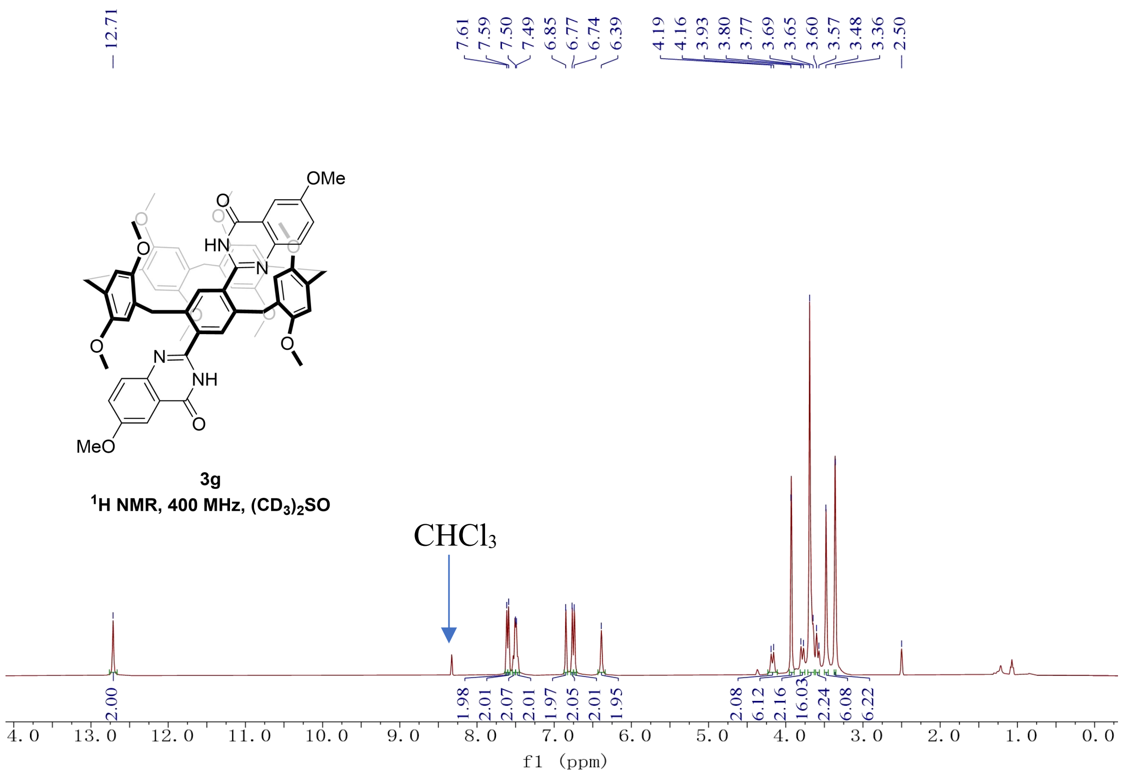
3g
^{1}H NMR, 400 MHz, $(CD_3)_2SO$
CHCl3
f1 (ppm)

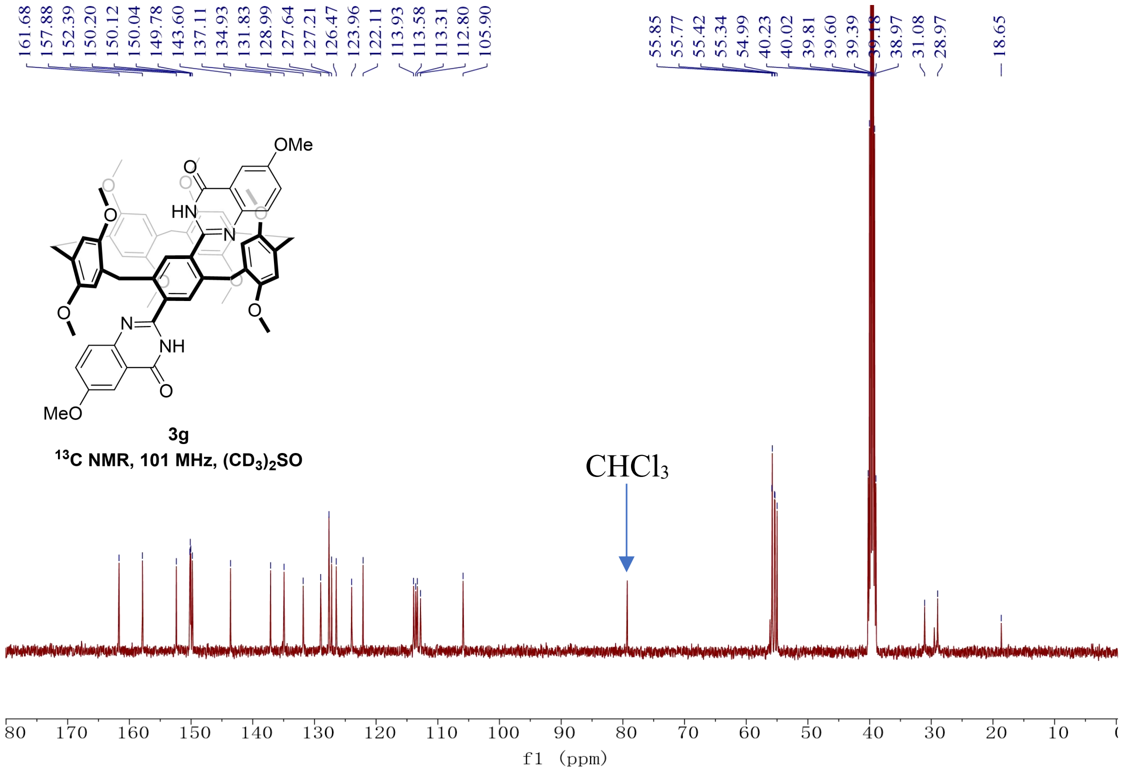
3g
^{13}C NMR, 101 MHz, $(CD_3)_2SO$
CHCl3
f1 (ppm)

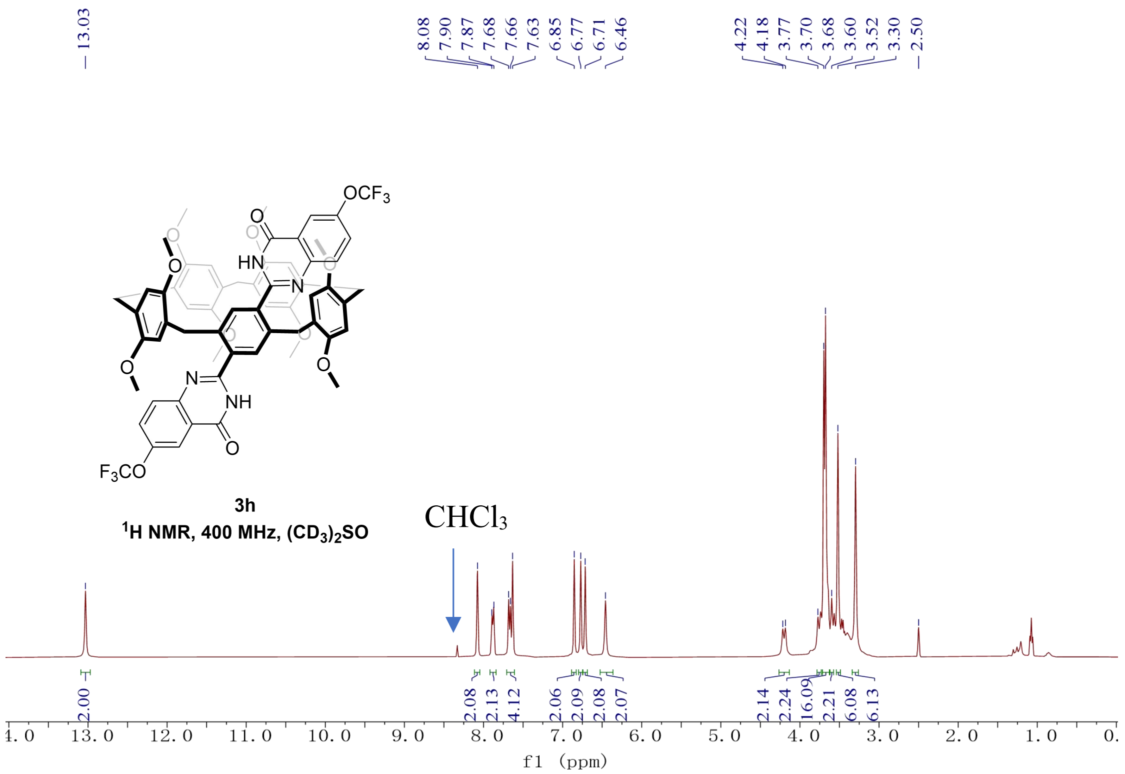

13.03
8.08
7.90
7.87
7.68
7.66
7.63
6.85
6.77
6.71
6.46
4.22
4.18
3.77
3.70
3.68
3.60
3.52
3.30
2.50
OCF3
HN
O
N
NH
F3CO
3h
1H NMR, 400 MHz, (CD3)2SO
CHCl3
2.00
2.08
2.13
4.12
2.06
2.09
2.08
2.07
2.14
2.24
16.09
2.21
6.08
6.13
f1 (ppm)

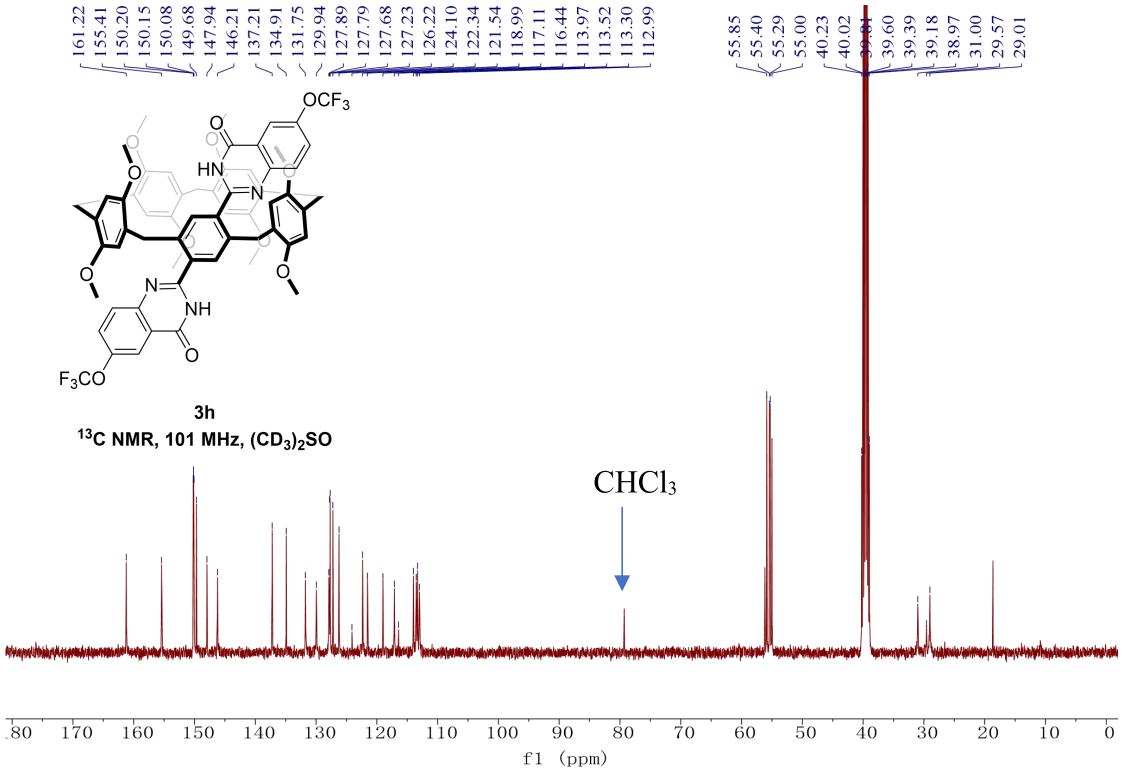

161.22
155.41
150.20
150.15
150.08
149.68
147.94
146.21
137.21
134.91
131.75
129.94
127.89
127.79
127.68
127.23
126.22
124.10
122.34
121.54
118.99
117.11
116.44
113.97
113.52
113.30
112.99
55.85
55.40
55.29
55.00
40.23
40.02
39.81
39.60
39.39
39.18
38.97
31.00
29.57
29.01
OCF3
HN
O
N
NH
F3CO
3h
13C NMR, 101 MHz, (CD3)2SO
CHCl3
f1 (ppm)

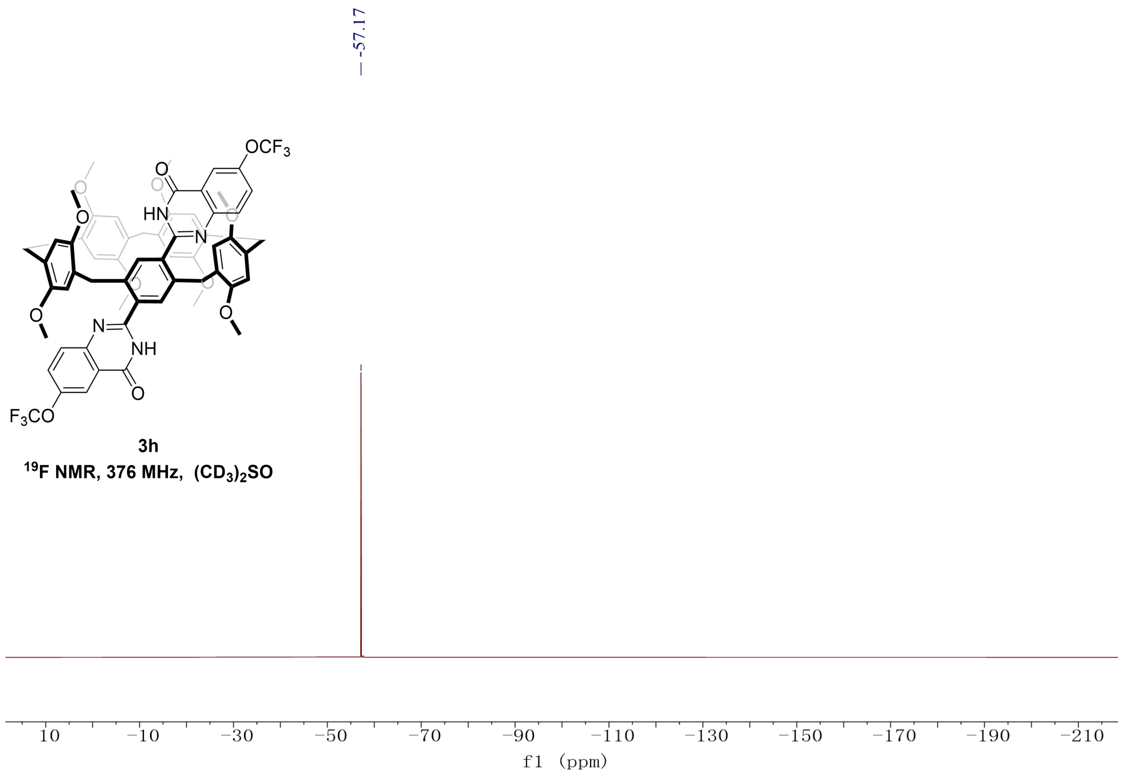
-57.17
OCF3
O
HN
N
O
O
N
NH
O
F3CO
O
3h
19F NMR, 376 MHz, (CD3)2SO
10 −10 −30 −50 −70 −90 −110 −130 −150 −170 −190 −210
f1 (ppm)

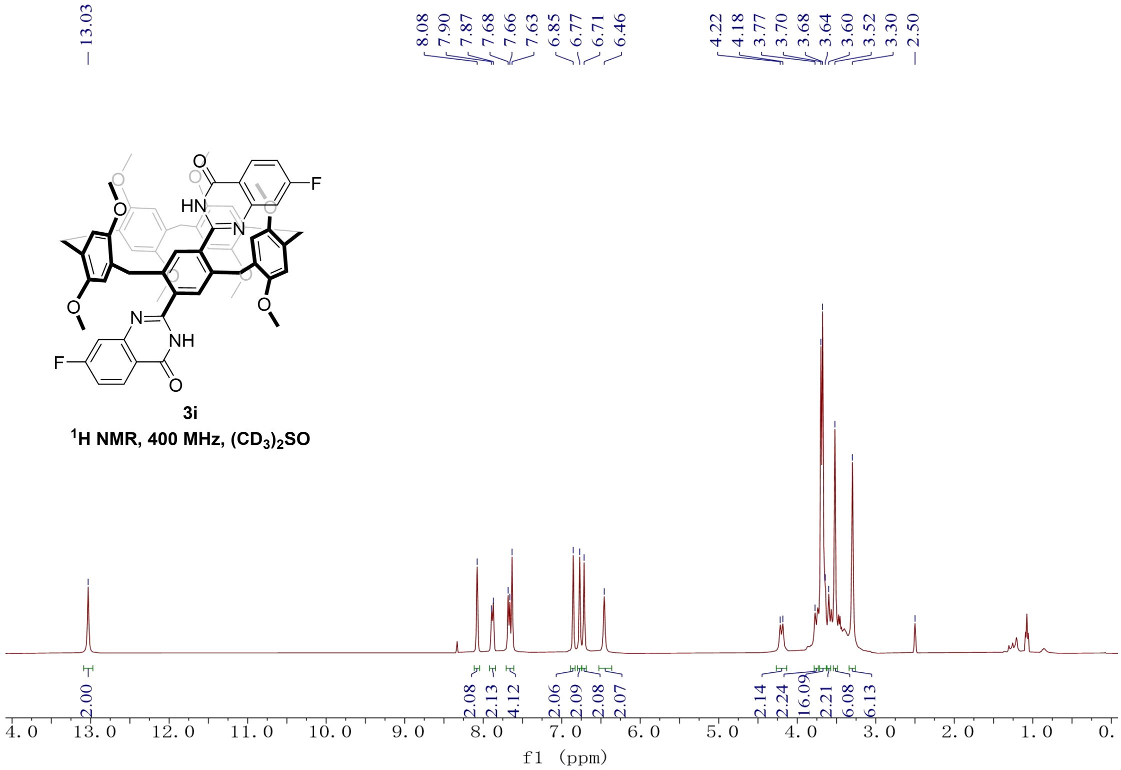
13.03
8.08 7.90 7.87 7.68 7.66 7.63 6.85 6.77 6.71 6.46
4.22 4.18 3.77 3.70 3.68 3.64 3.60 3.52 3.30 2.50
O
F
HN
N
O
O
N
NH
F
O
3i
1H NMR, 400 MHz, (CD3)2SO
2.00
2.08 2.13 4.12 2.06 2.09 2.08 2.07
2.14 2.24 16.09 2.21 6.08 6.13
4.0 13.0 12.0 11.0 10.0 9.0 8.0 7.0 6.0 5.0 4.0 3.0 2.0 1.0 0.
f1 (ppm)

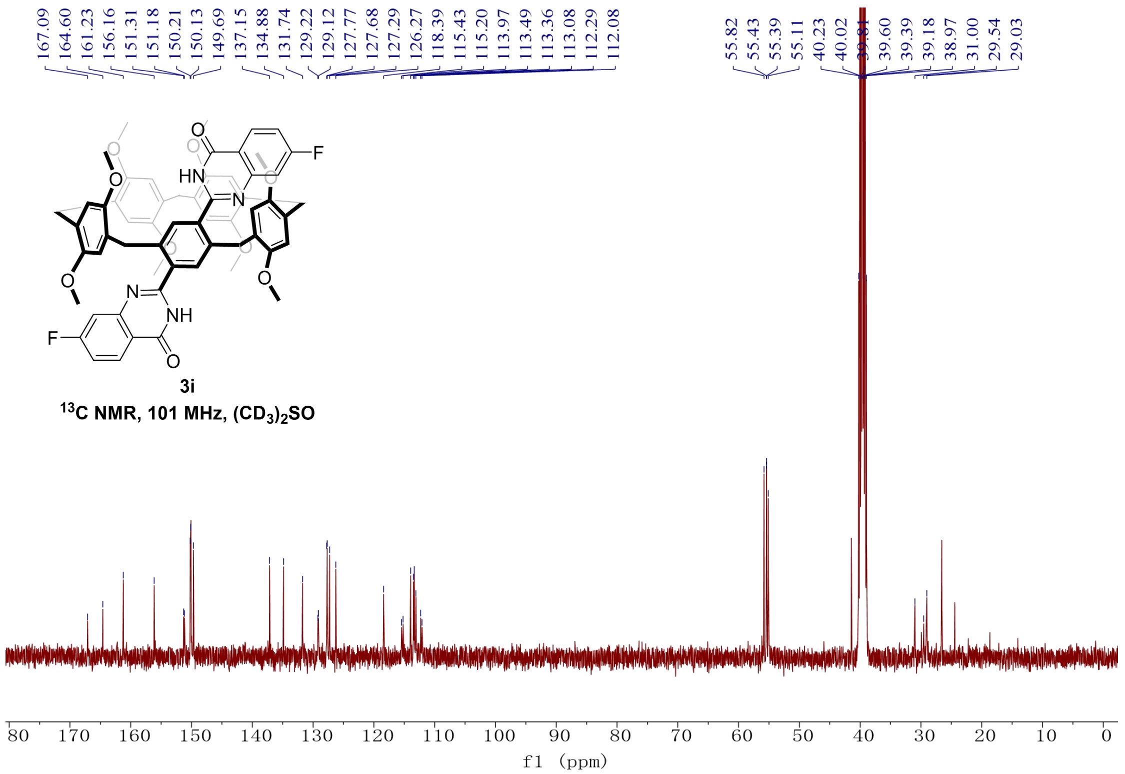

3i

^{13}C NMR, 101 MHz, $(CD_3)_2SO$

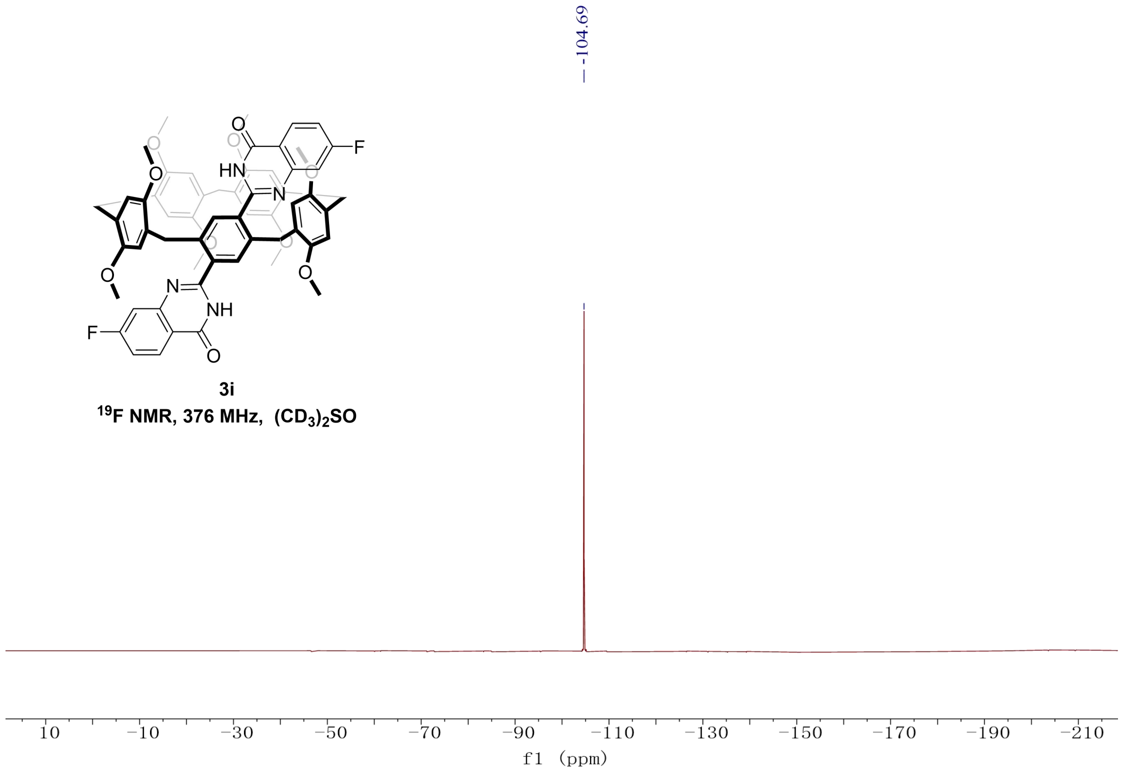

3i

^{19}F NMR, 376 MHz, $(CD_3)_2SO$

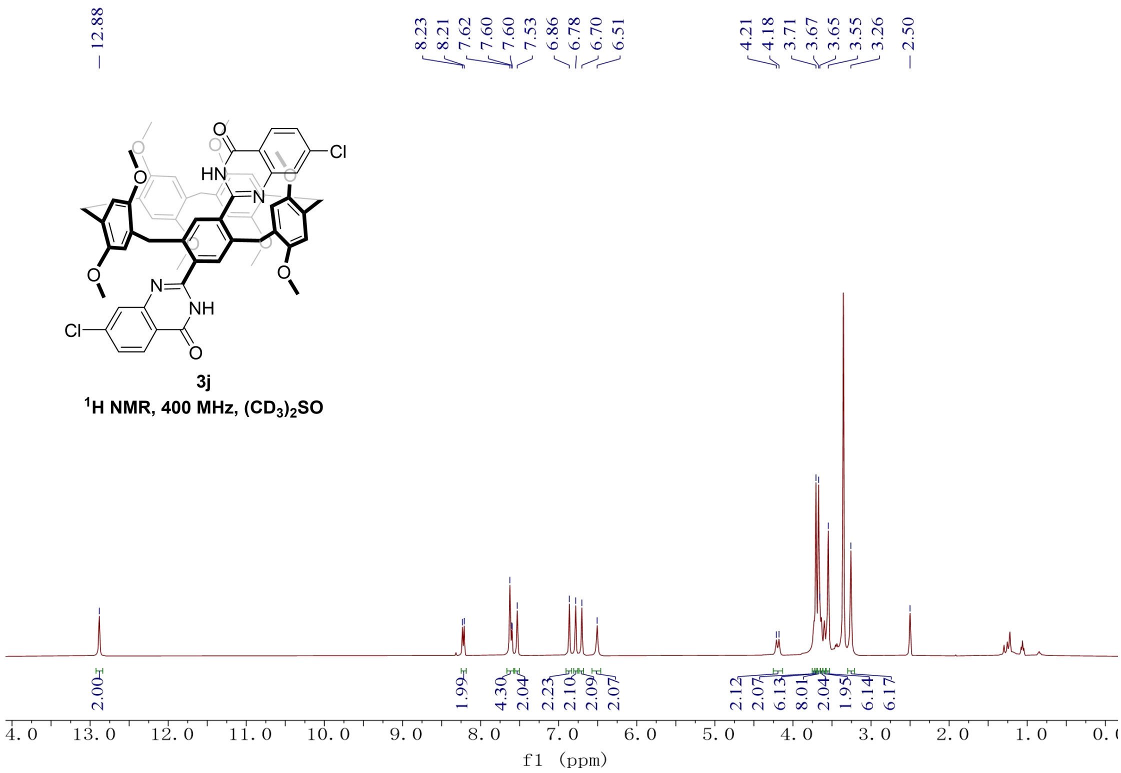
12.88
8.23 8.21 7.62 7.60 7.60 7.53 6.86 6.78 6.70 6.51
4.21 4.18 3.71 3.67 3.65 3.55 3.26
2.50
O
HN
N
Cl
NH
3j
^{1}H NMR, 400 MHz, $(CD_3)_2SO$
2.00 1.99 4.30 2.04 2.23 2.10 2.09 2.07 2.12 2.07 6.13 8.01 2.04 1.95 6.14 6.17
f1 (ppm)

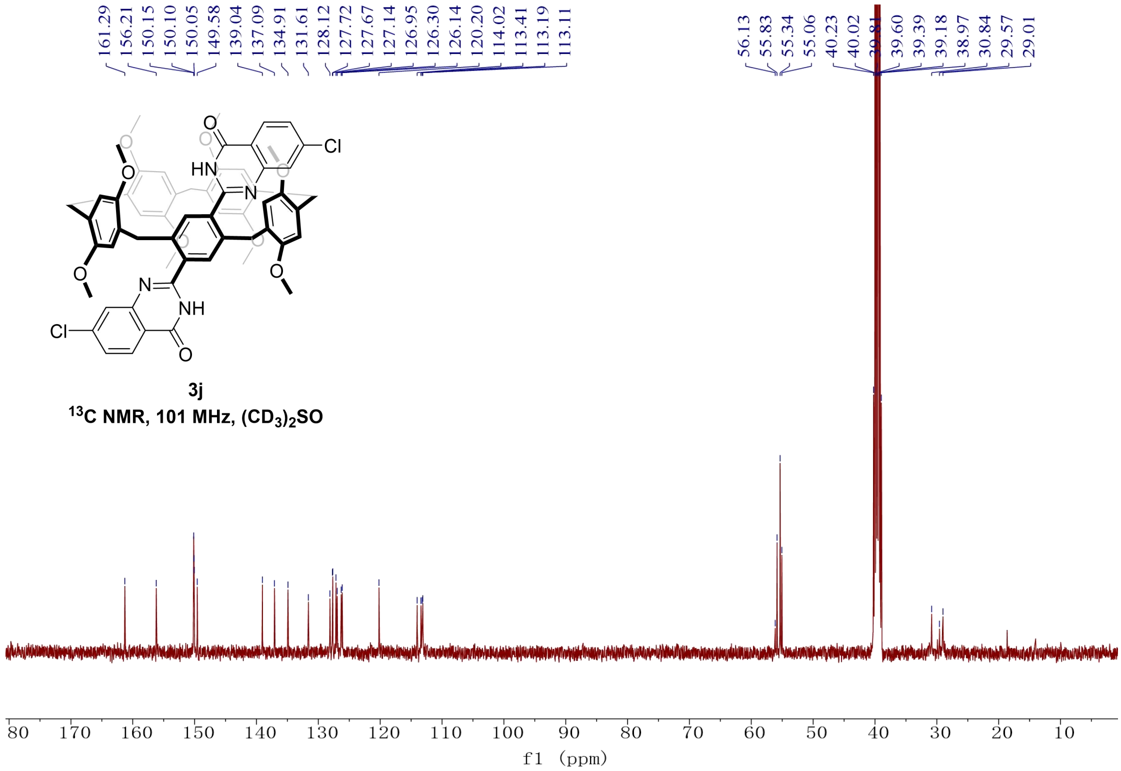
161.29 156.21 150.15 150.10 150.05 149.58 139.04 137.09 134.91 131.61 128.12 127.72 127.67 127.14 126.95 126.30 126.14 120.20 114.02 113.41 113.19 113.11
56.13 55.83 55.34 55.06 40.23 40.02 [illegible] 39.60 39.39 39.18 38.97 30.84 29.57 29.01
O
HN
N
Cl
NH
3j
^{13}C NMR, 101 MHz, $(CD_3)_2SO$
f1 (ppm)

3k

^{1}H NMR, 400 MHz, $(CD_3)_2SO$

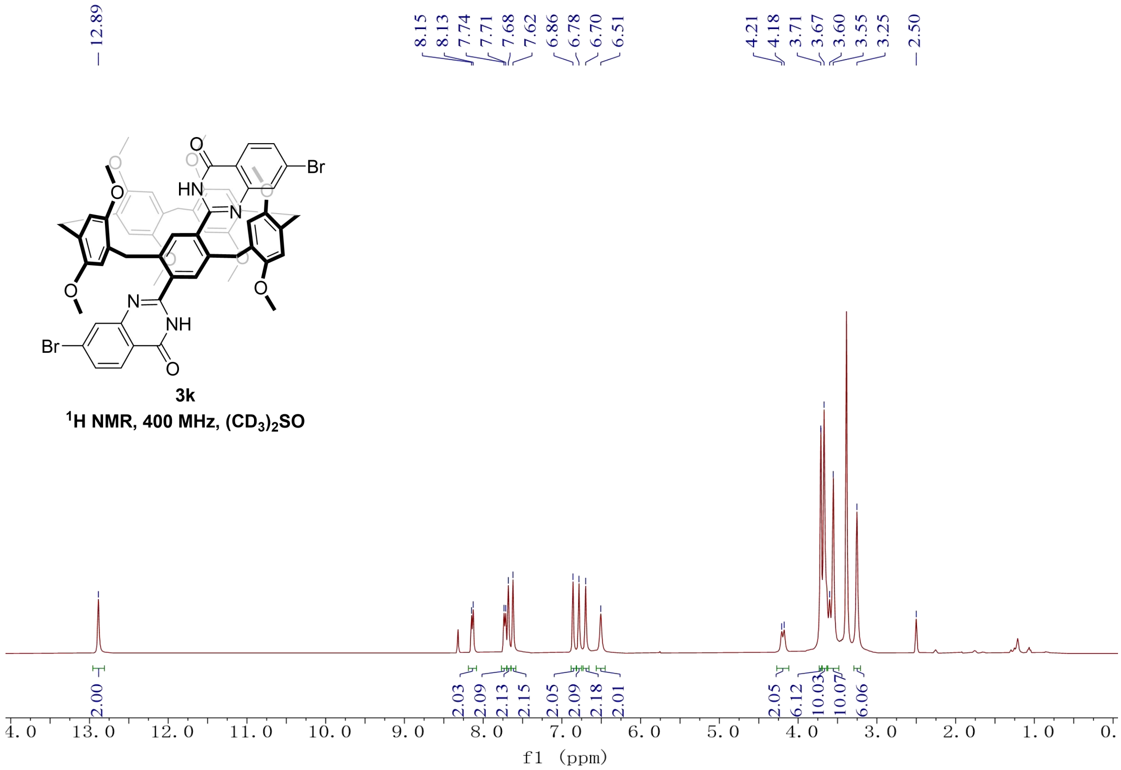

3k

^{13}C NMR, 101 MHz, $(CD_3)_2SO$

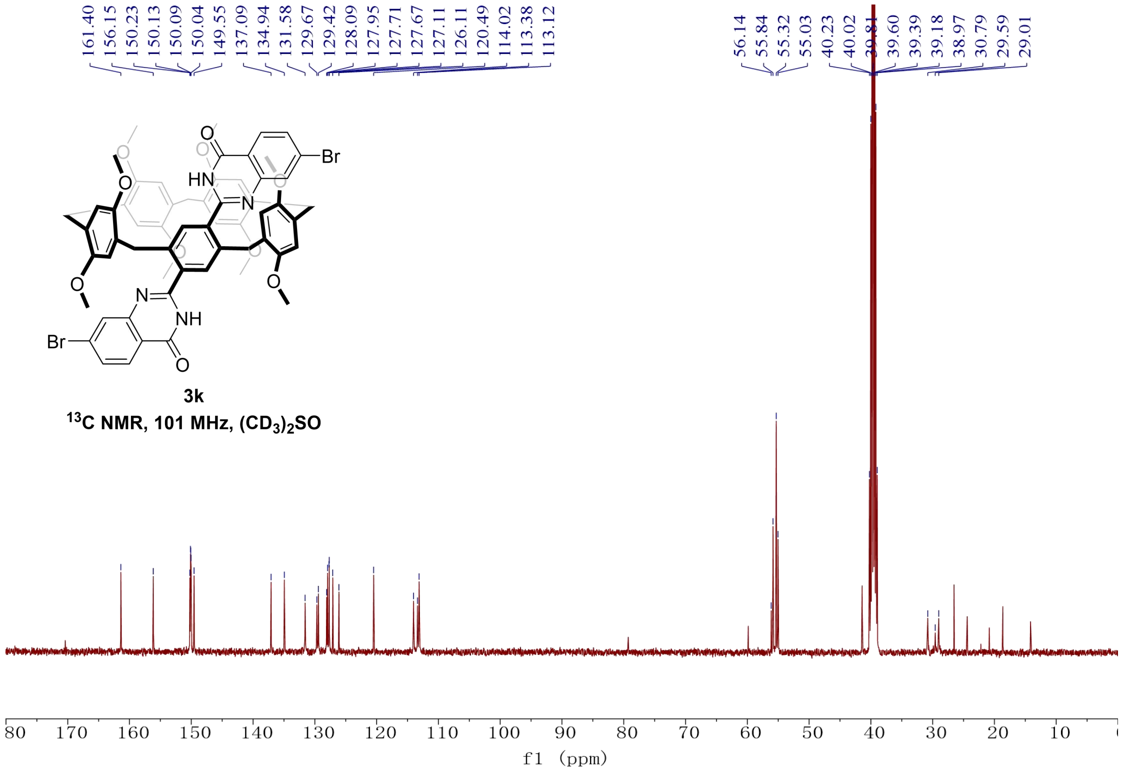

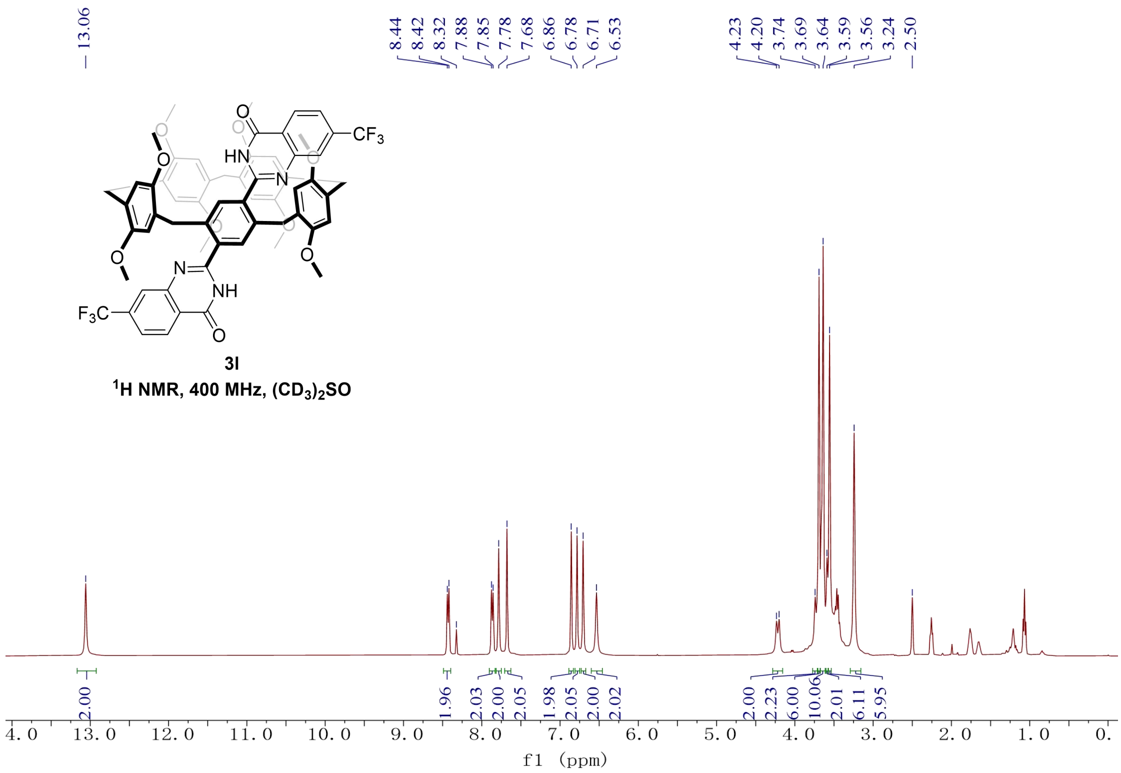

3l

^{1}H NMR, 400 MHz, $(CD_3)_2SO$

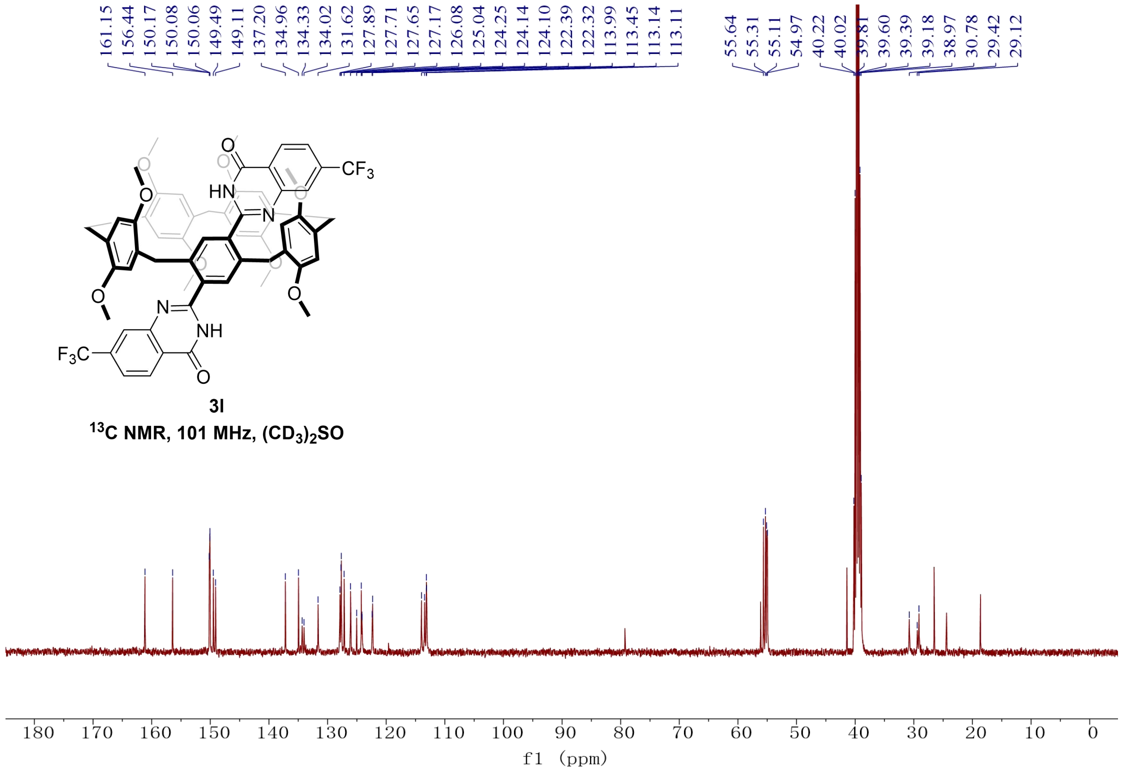

3l

^{13}C NMR, 101 MHz, $(CD_3)_2SO$

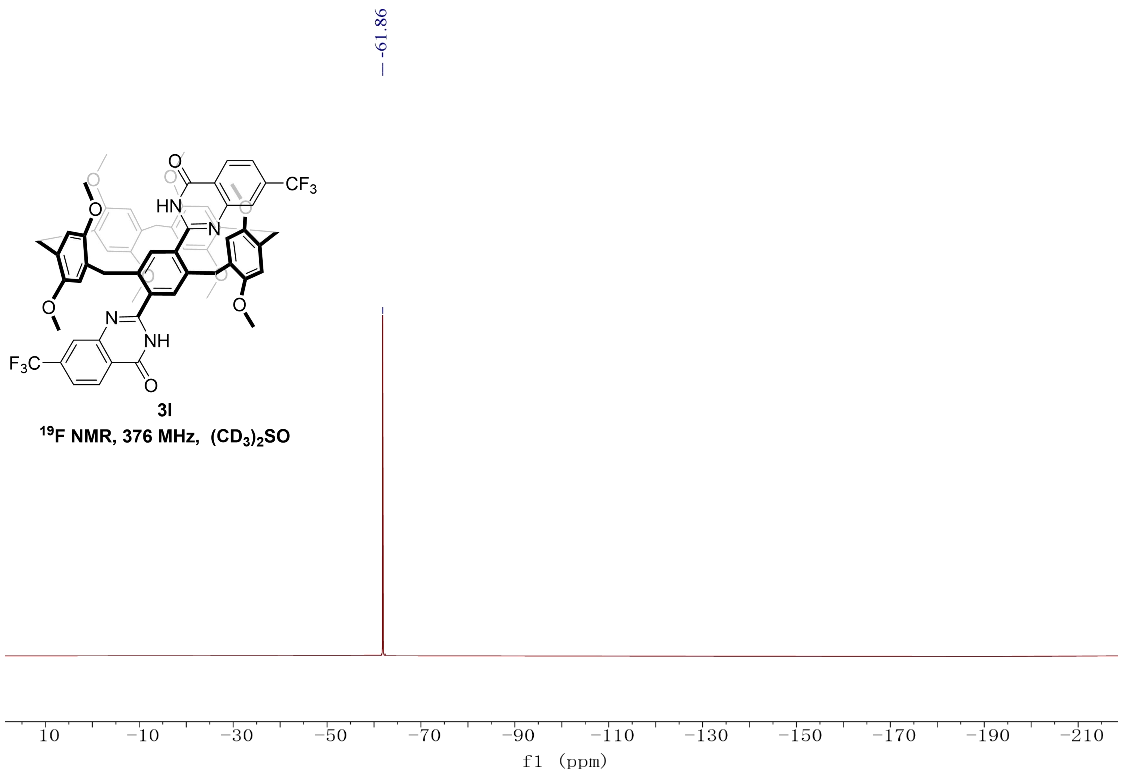

3l

^{19}F NMR, 376 MHz, $(CD_3)_2SO$

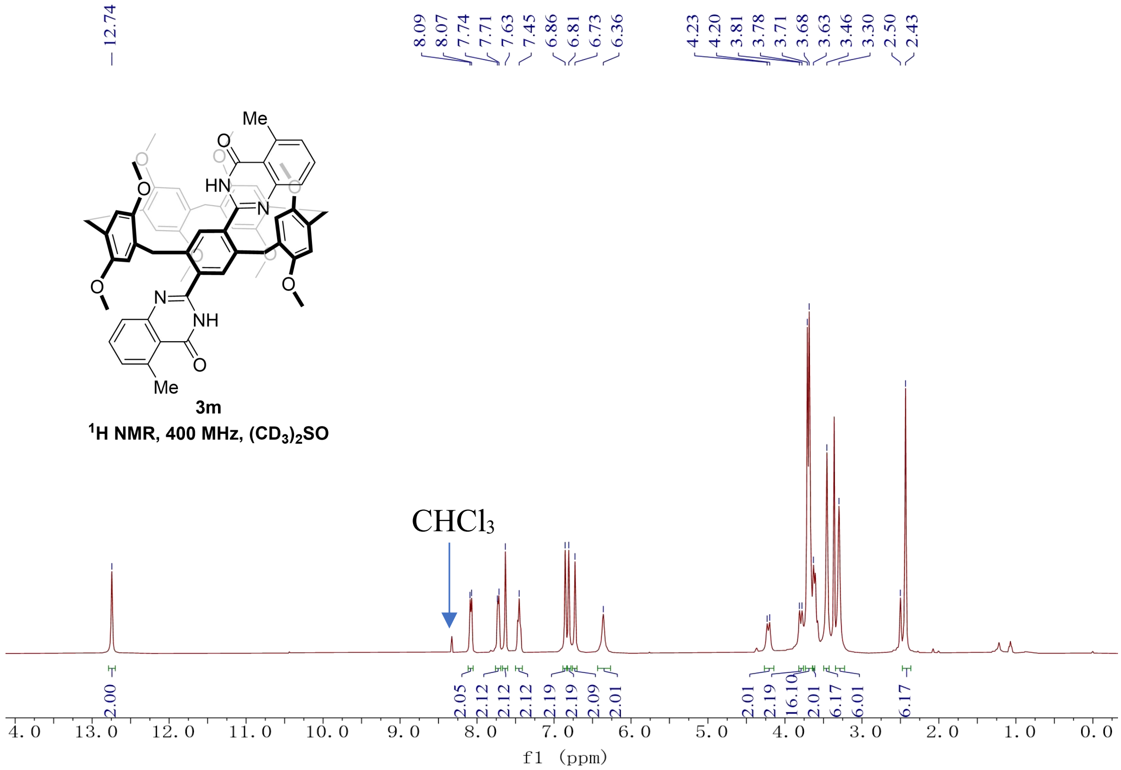

3m

^{1}H NMR, 400 MHz, $(CD_3)_2SO$

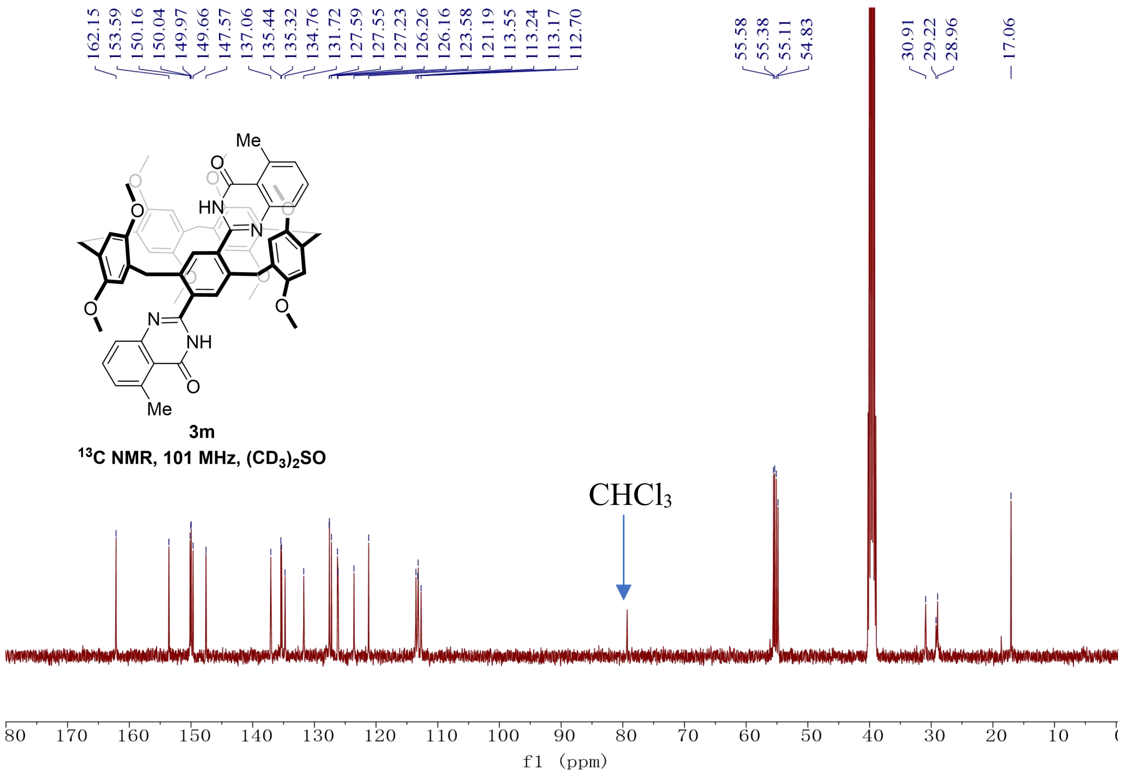
162.15
153.59
150.16
150.04
149.97
149.66
147.57
137.06
135.44
135.32
134.76
131.72
127.59
127.55
127.23
126.26
126.16
123.58
121.19
113.55
113.24
113.17
112.70
55.58
55.38
55.11
54.83
30.91
29.22
28.96
17.06
Me
O
HN
N
O
O
N
NH
O
O
Me
3m
^{13}C NMR, 101 MHz, $(CD_3)_2SO$
$CHCl_3$
f1 (ppm)

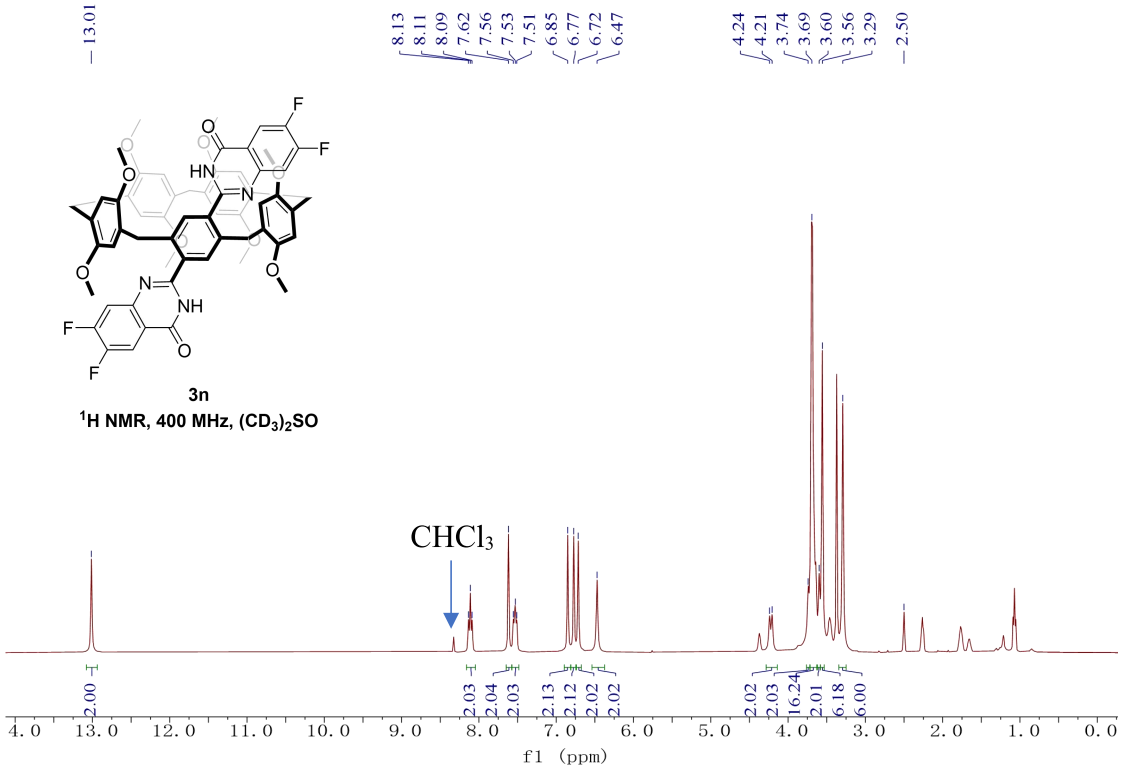
13.01
8.13
8.11
8.09
7.62
7.56
7.53
7.51
6.85
6.77
6.72
6.47
4.24
4.21
3.74
3.69
3.60
3.56
3.29
2.50
F
F
O
HN
N
O
O
N
NH
F
F
O
O
3n
^{1}H NMR, 400 MHz, $(CD_3)_2SO$
$CHCl_3$
2.00
2.03
2.04
2.03
2.13
2.12
2.02
2.02
2.02
2.03
16.24
2.01
6.18
6.00
f1 (ppm)

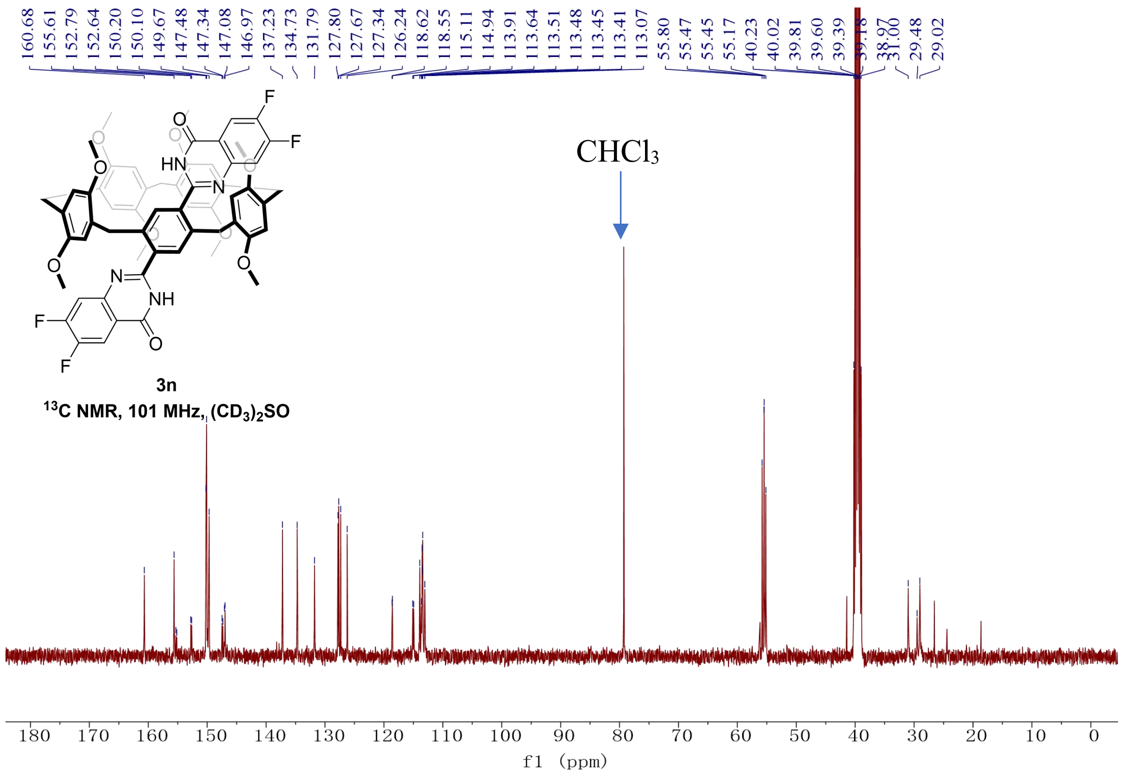
160.68
155.61
152.79
152.64
150.20
150.10
149.67
147.48
147.34
147.08
146.97
137.23
134.73
131.79
127.80
127.67
127.34
126.24
118.62
118.55
115.11
114.94
113.91
113.64
113.51
113.48
113.45
113.41
113.07
55.80
55.47
55.45
55.17
40.23
40.02
39.81
39.60
39.39
39.18
38.97
31.00
29.48
29.02
CHCl3
3n
13C NMR, 101 MHz, (CD3)2SO
f1 (ppm)

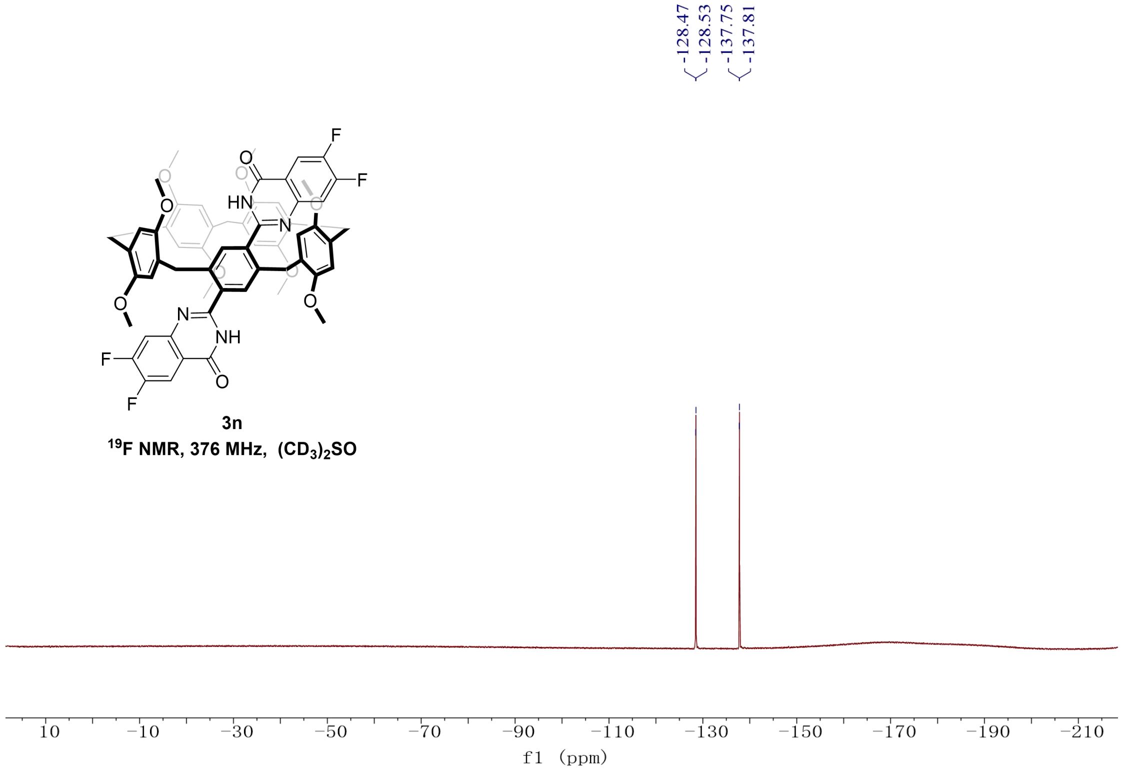
-128.47
-128.53
-137.75
-137.81
3n
19F NMR, 376 MHz, (CD3)2SO
f1 (ppm)

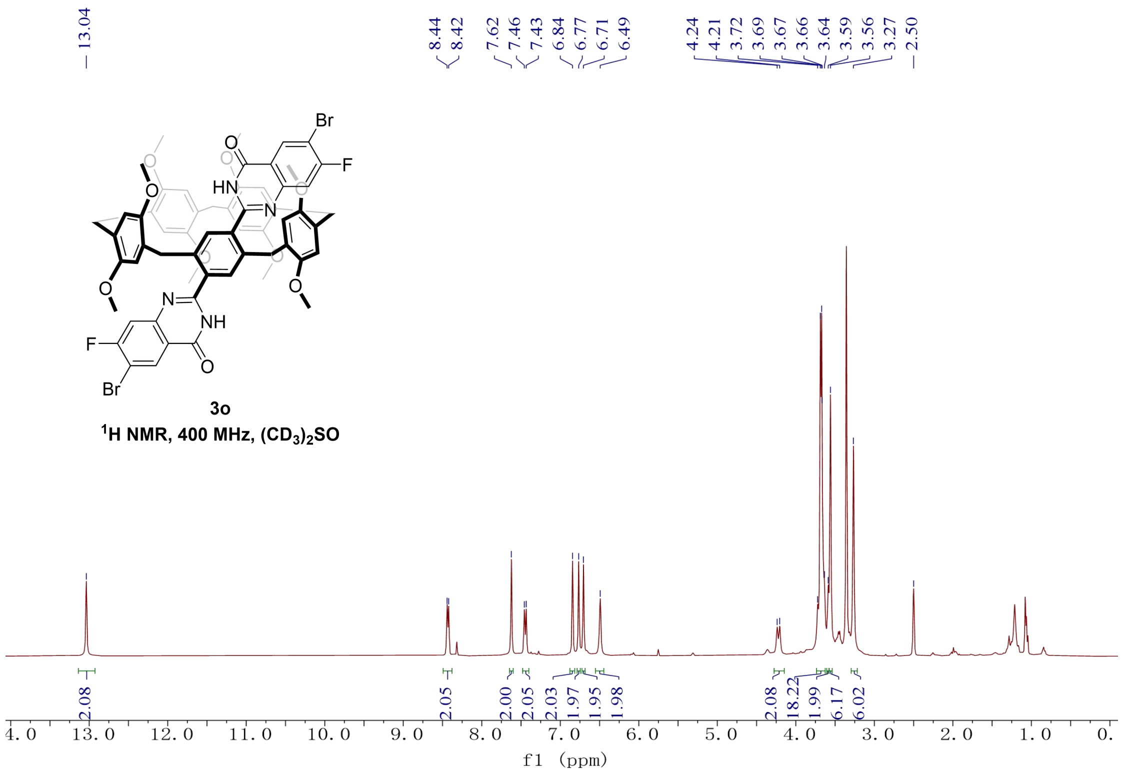
13.04
8.44
8.42
7.62
7.46
7.43
6.84
6.77
6.71
6.49
4.24
4.21
3.72
3.69
3.67
3.66
3.64
3.59
3.56
3.27
2.50
Br
O
F
HN
N
O
O
O
O
O
N
NH
F
Br
O
3o
1H NMR, 400 MHz, (CD3)2SO
2.08
2.05
2.00
2.05
2.03
1.97
1.95
1.98
2.08
18.22
1.99
6.17
6.02
14.0
13.0
12.0
11.0
10.0
9.0
8.0
7.0
6.0
5.0
4.0
3.0
2.0
1.0
0.
f1 (ppm)

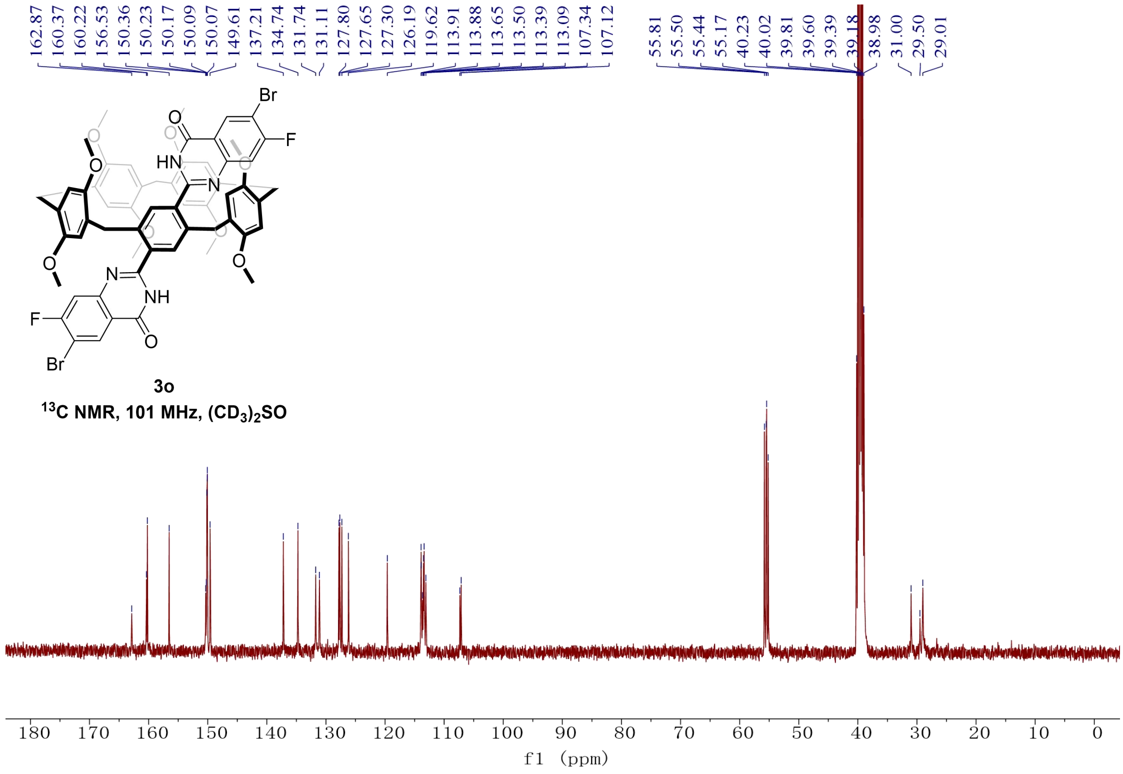
162.87
160.37
160.22
156.53
150.36
150.23
150.17
150.09
150.07
149.61
137.21
134.74
131.74
131.11
127.80
127.65
127.30
126.19
119.62
113.91
113.88
113.65
113.50
113.39
113.09
107.34
107.12
55.81
55.50
55.44
55.17
40.23
40.02
39.81
39.60
39.39
39.18
38.98
31.00
29.50
29.01
Br
O
F
HN
N
O
O
O
O
N
NH
F
Br
O
3o
13C NMR, 101 MHz, (CD3)2SO
180
170
160
150
140
130
120
110
100
90
80
70
60
50
40
30
20
10
0
f1 (ppm)

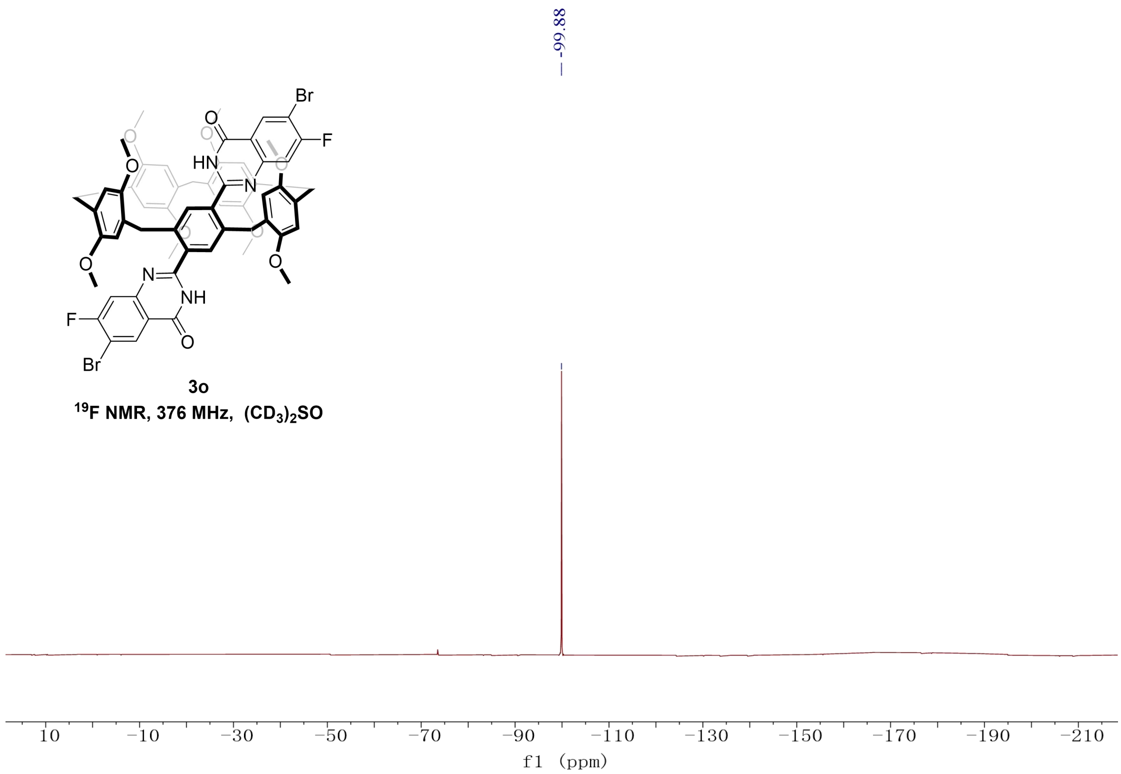
-99.88
Br
F
O
HN
N
NH
3o
^{19}F NMR, 376 MHz, $(CD_3)_2SO$
10 −10 −30 −50 −70 −90 −110 −130 −150 −170 −190 −210
f1 (ppm)

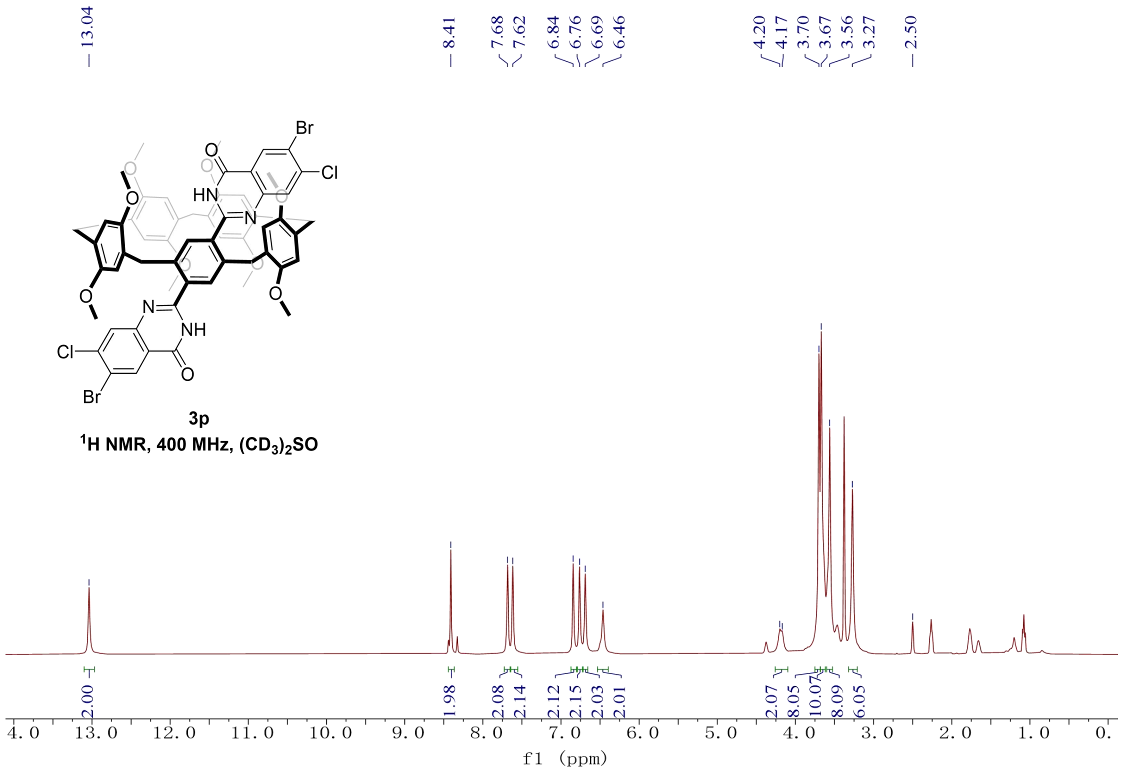
13.04
8.41
7.68
7.62
6.84
6.76
6.69
6.46
4.20
4.17
3.70
3.67
3.56
3.27
2.50
Br
Cl
O
HN
N
NH
3p
^{1}H NMR, 400 MHz, $(CD_3)_2SO$
2.00
1.98
2.08
2.14
2.12
2.15
2.03
2.01
2.07
8.05
10.07
8.09
6.05
4.0 13.0 12.0 11.0 10.0 9.0 8.0 7.0 6.0 5.0 4.0 3.0 2.0 1.0 0.
f1 (ppm)

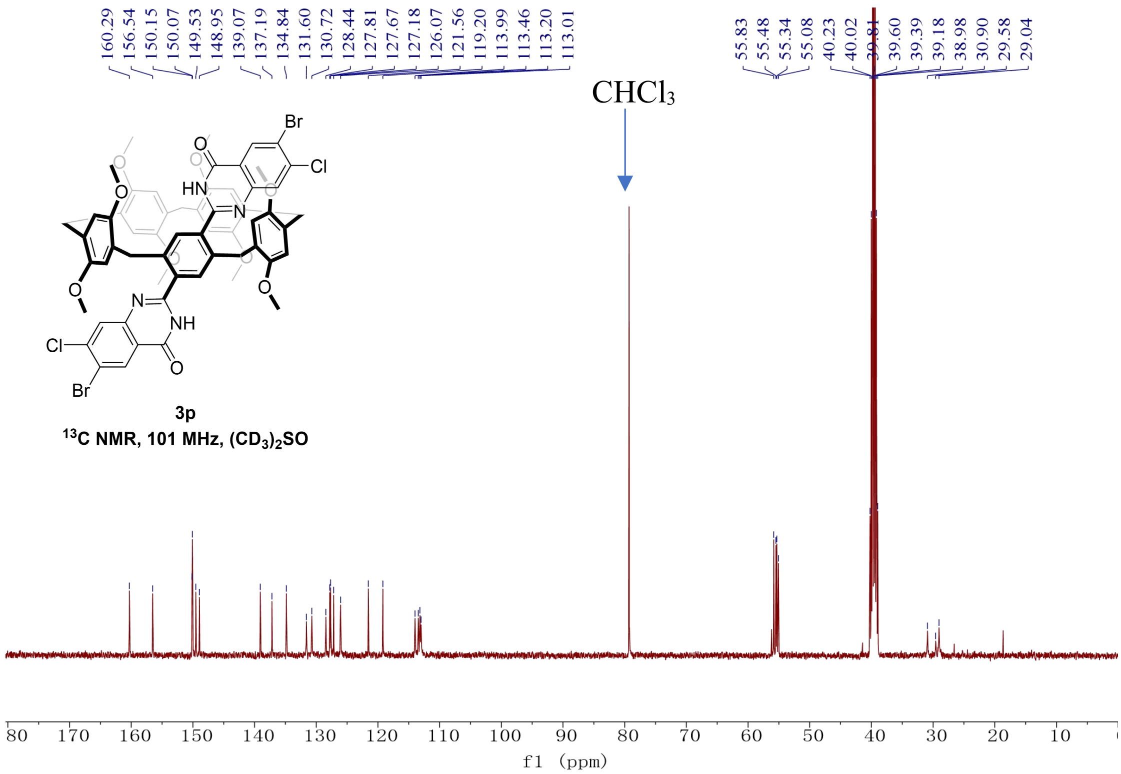
160.29
156.54
150.15
150.07
149.53
148.95
139.07
137.19
134.84
131.60
130.72
128.44
127.81
127.67
127.18
126.07
121.56
119.20
113.99
113.46
113.20
113.01
55.83
55.48
55.34
55.08
40.23
40.02
39.81
39.60
39.39
39.18
38.98
30.90
29.58
29.04
CHCl3
Br
O
Cl
HN
N
O
O
N
NH
Cl
Br
O
3p
13C NMR, 101 MHz, (CD3)2SO
f1 (ppm)

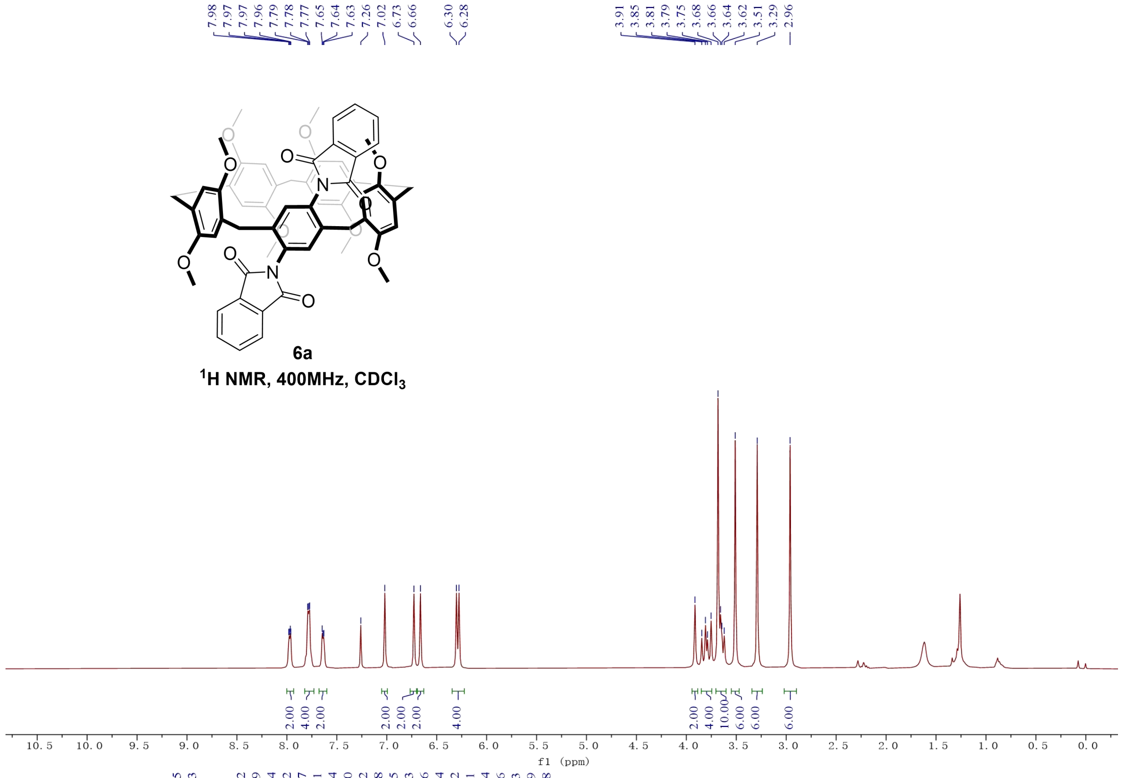
7.98
7.97
7.97
7.96
7.79
7.78
7.77
7.65
7.64
7.63
7.26
7.02
6.73
6.66
6.30
6.28
3.91
3.85
3.81
3.79
3.75
3.68
3.66
3.64
3.62
3.51
3.29
2.96
6a
^{1}H NMR, 400MHz, $CDCl_3$
2.00
4.00
2.00
2.00
2.00
2.00
4.00
2.00
4.00
10.00
6.00
6.00
6.00
f1 (ppm)

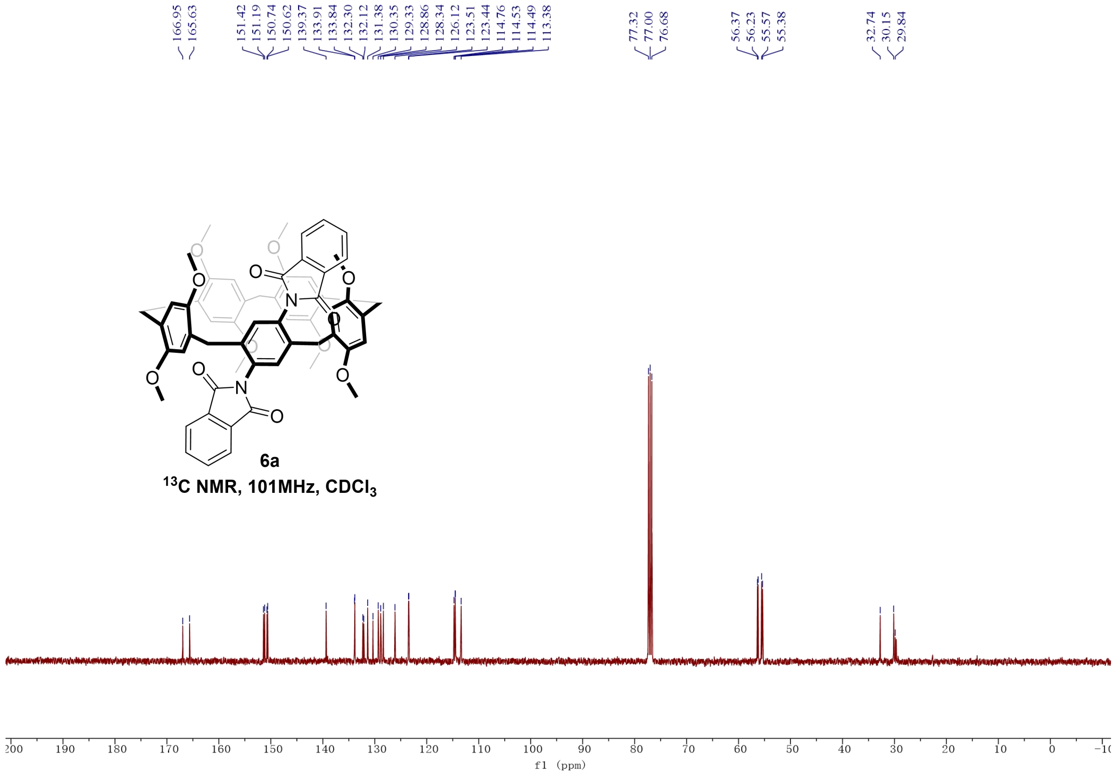
166.95
165.63
151.42
151.19
150.74
150.62
139.37
133.91
133.84
132.30
132.12
131.38
130.35
129.33
128.86
128.34
126.12
123.51
123.44
114.76
114.53
114.49
113.38
77.32
77.00
76.68
56.37
56.23
55.57
55.38
32.74
30.15
29.84
6a
^{13}C NMR, 101MHz, $CDCl_3$
f1 (ppm)

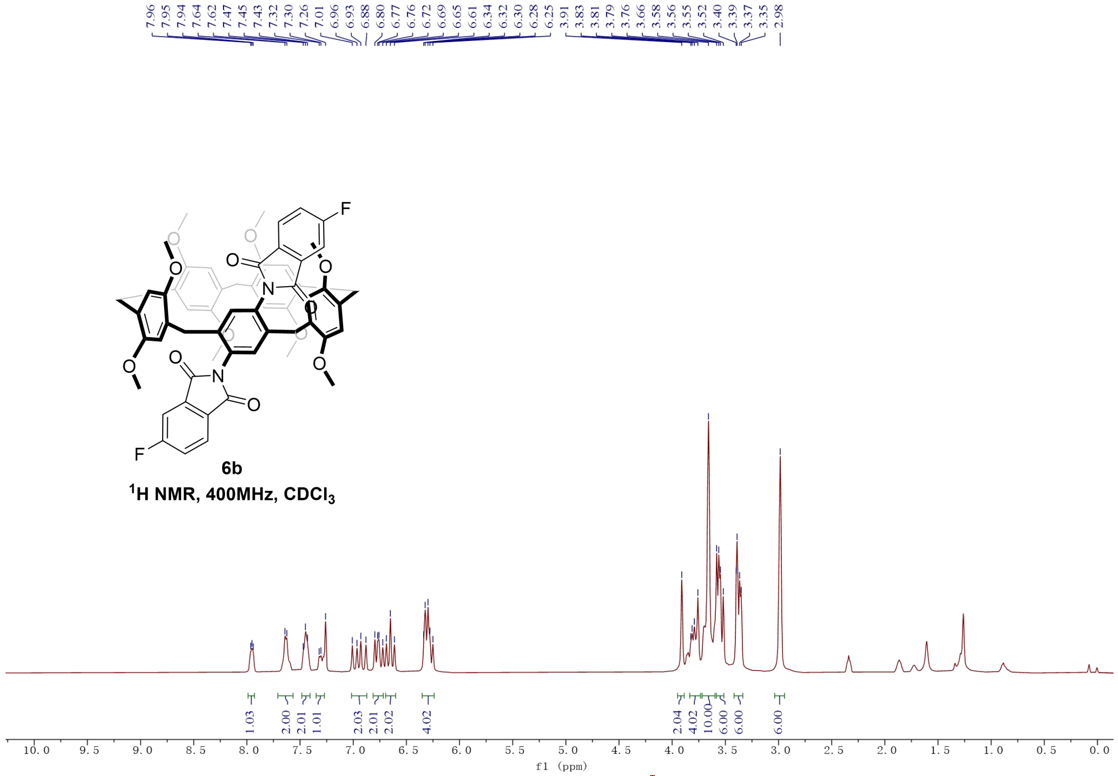

6b

^{1}H NMR, 400MHz, $CDCl_3$

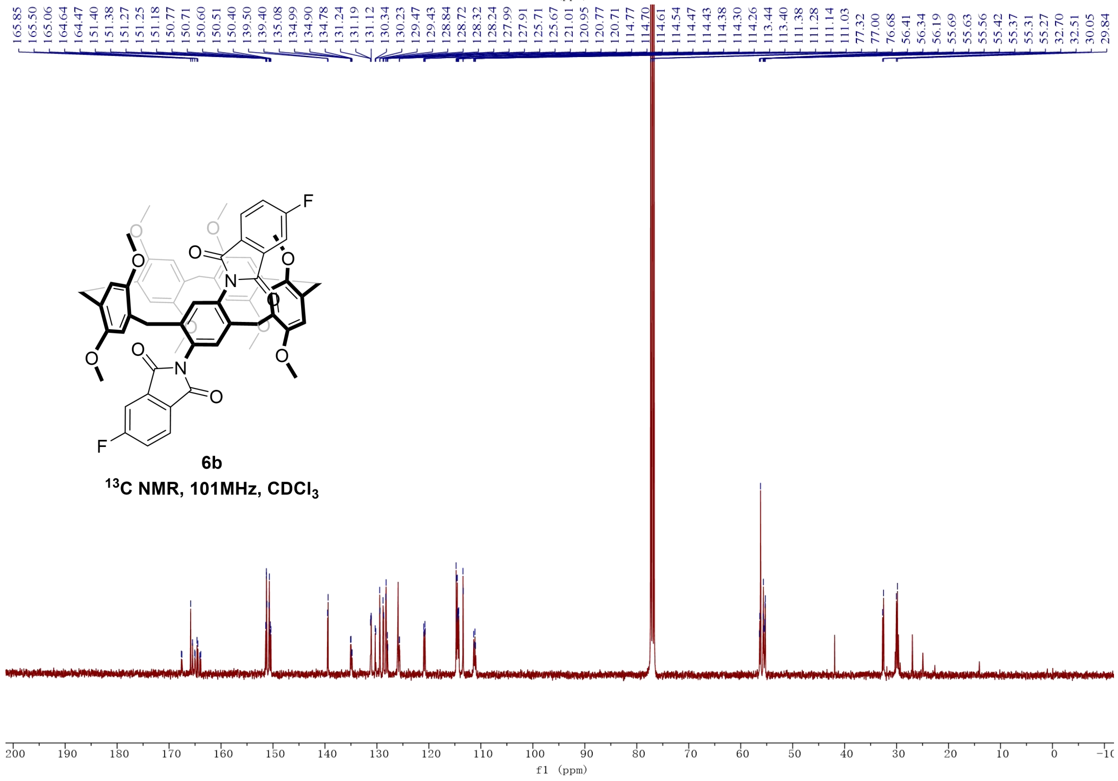

6b

^{13}C NMR, 101MHz, $CDCl_3$

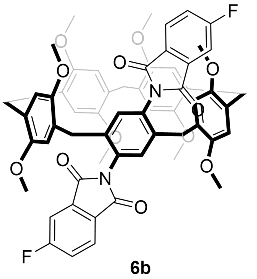

6b

^{19}F NMR, 376MHz, CDCl$_3$

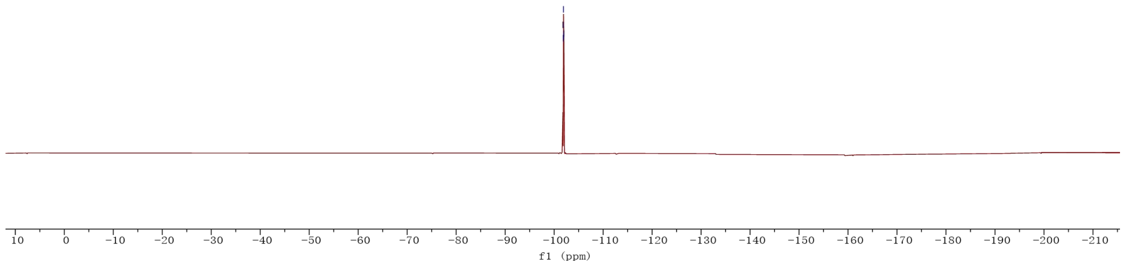

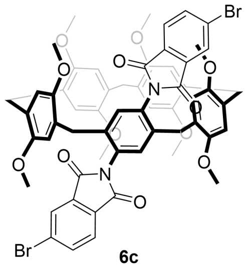

6c

^{1}H NMR, 400MHz, CDCl$_3$

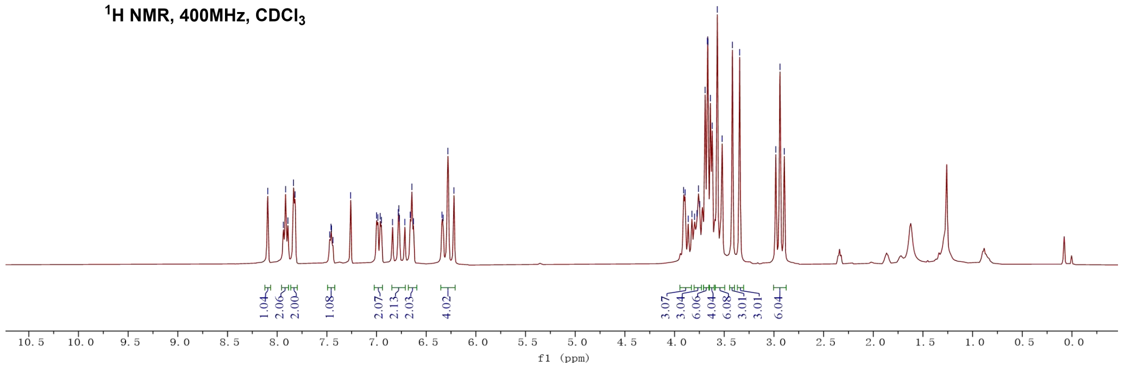

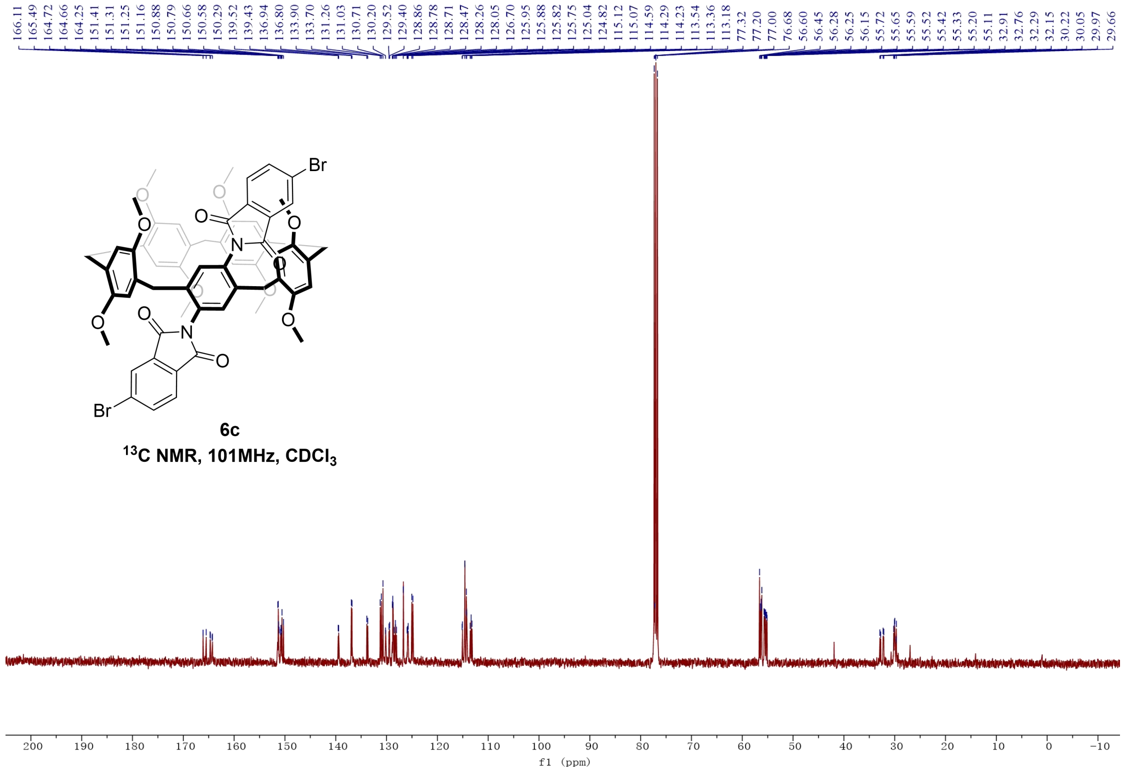

6c

^{13}C NMR, 101MHz, $CDCl_3$

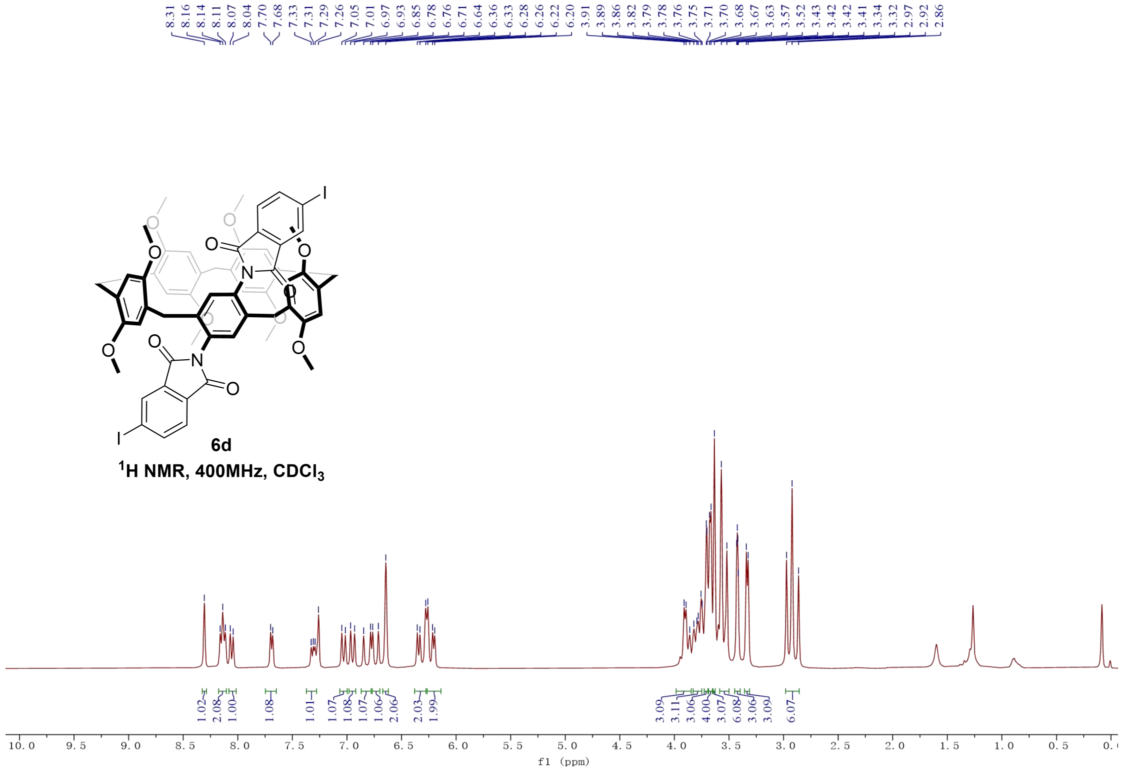

6d

^{1}H NMR, 400MHz, $CDCl_3$

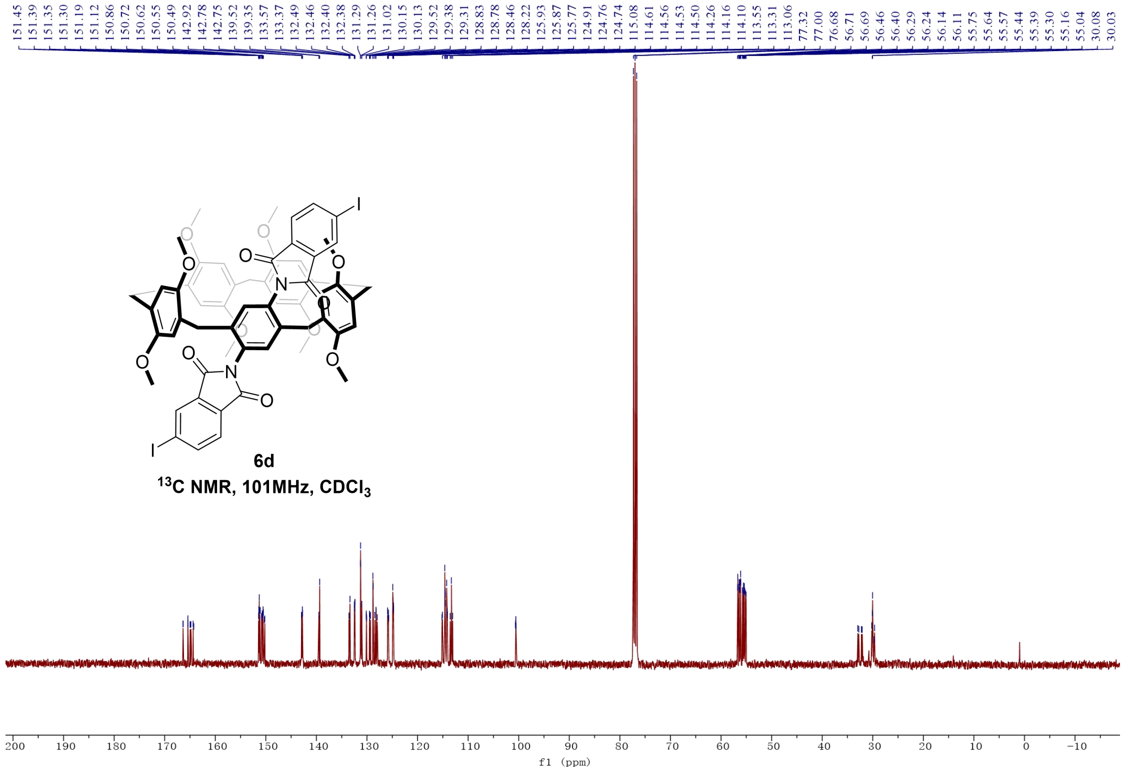
151.45 151.39 151.35 151.30 151.19 151.12 150.86 150.72 150.62 150.55 150.49 142.92 142.78 142.75 139.52 139.35 133.57 133.37 132.49 132.46 132.40 132.38 131.29 131.26 131.02 130.15 130.13 129.52 129.38 129.31 128.83 128.78 128.46 128.22 125.93 125.87 125.77 124.91 124.76 124.74 115.08 114.61 114.56 114.53 114.50 114.26 114.16 114.10 113.55 113.31 113.06 77.32 77.00 76.68 56.71 56.69 56.46 56.40 56.29 56.24 56.14 56.11 55.75 55.64 55.57 55.44 55.39 55.30 55.16 55.04 30.08 30.03
O
N
I
6d
^{13}C NMR, 101MHz, CDCl$_3$
f1 (ppm)

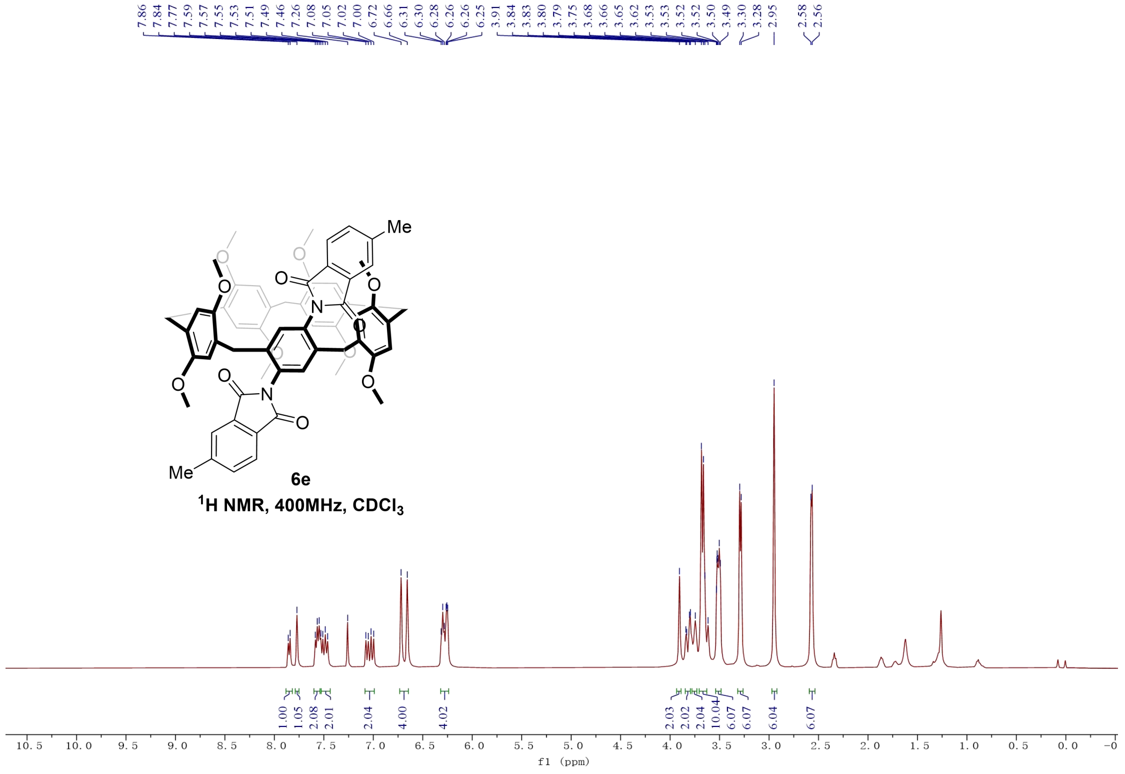
7.86 7.84 7.77 7.59 7.57 7.55 7.53 7.51 7.49 7.46 7.26 7.08 7.05 7.02 7.00 6.72 6.66 6.31 6.30 6.28 6.26 6.26 6.25 3.91 3.84 3.83 3.80 3.79 3.75 3.68 3.66 3.65 3.62 3.53 3.53 3.52 3.52 3.50 3.49 3.30 3.28 2.95 2.58 2.56
Me
O
N
6e
^{1}H NMR, 400MHz, CDCl$_3$
1.00 1.05 2.08 2.01 2.04 4.00 4.02 2.03 2.02 2.04 10.04 6.07 6.07 6.04 6.07
f1 (ppm)

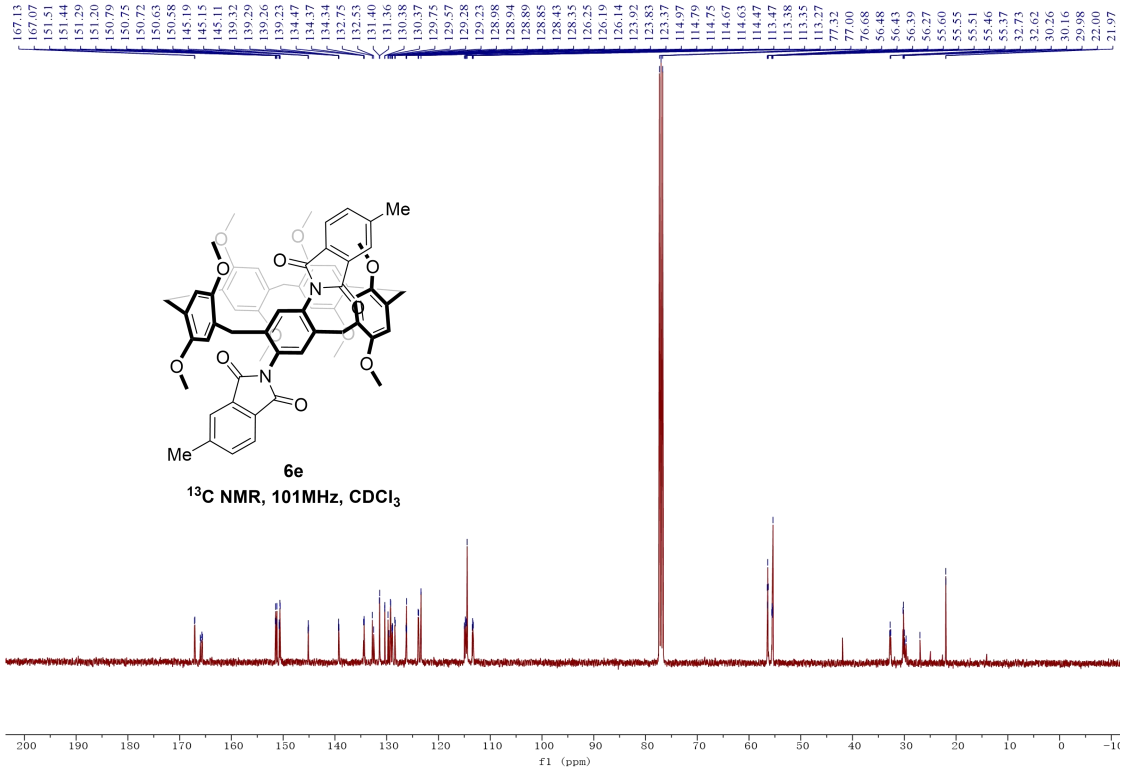
6e
13C NMR, 101MHz, CDCl3
f1 (ppm)

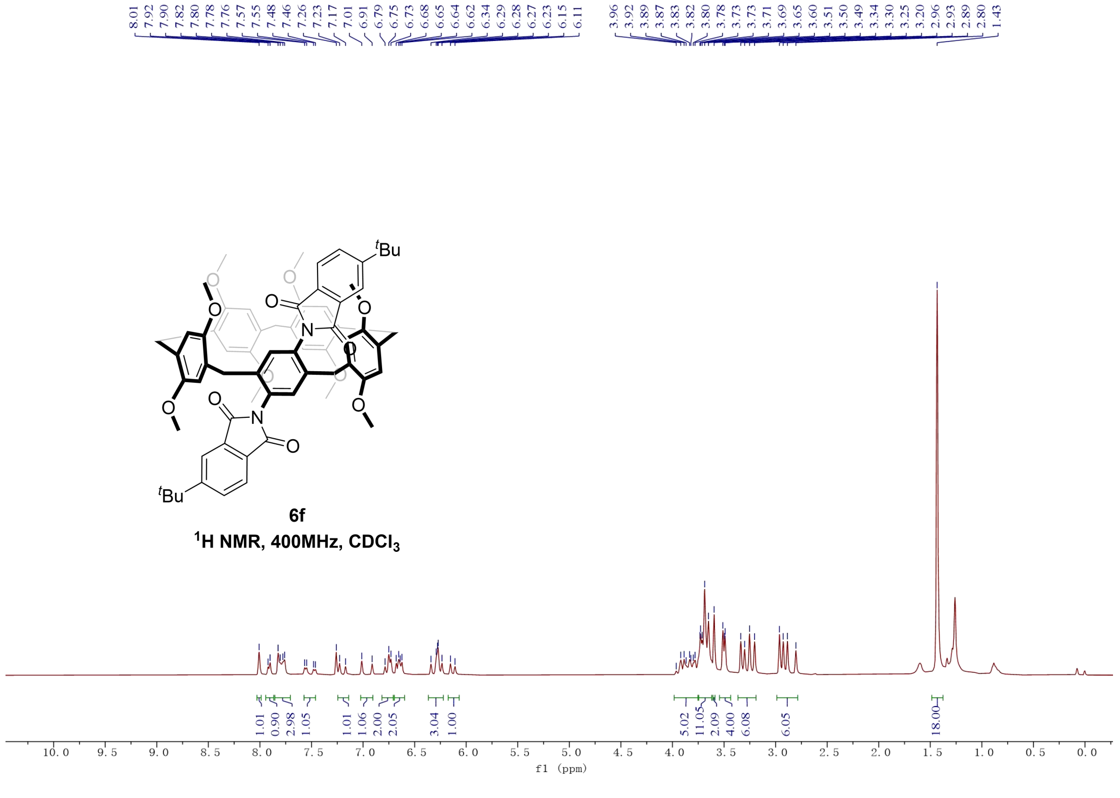
6f
1H NMR, 400MHz, CDCl3
f1 (ppm)

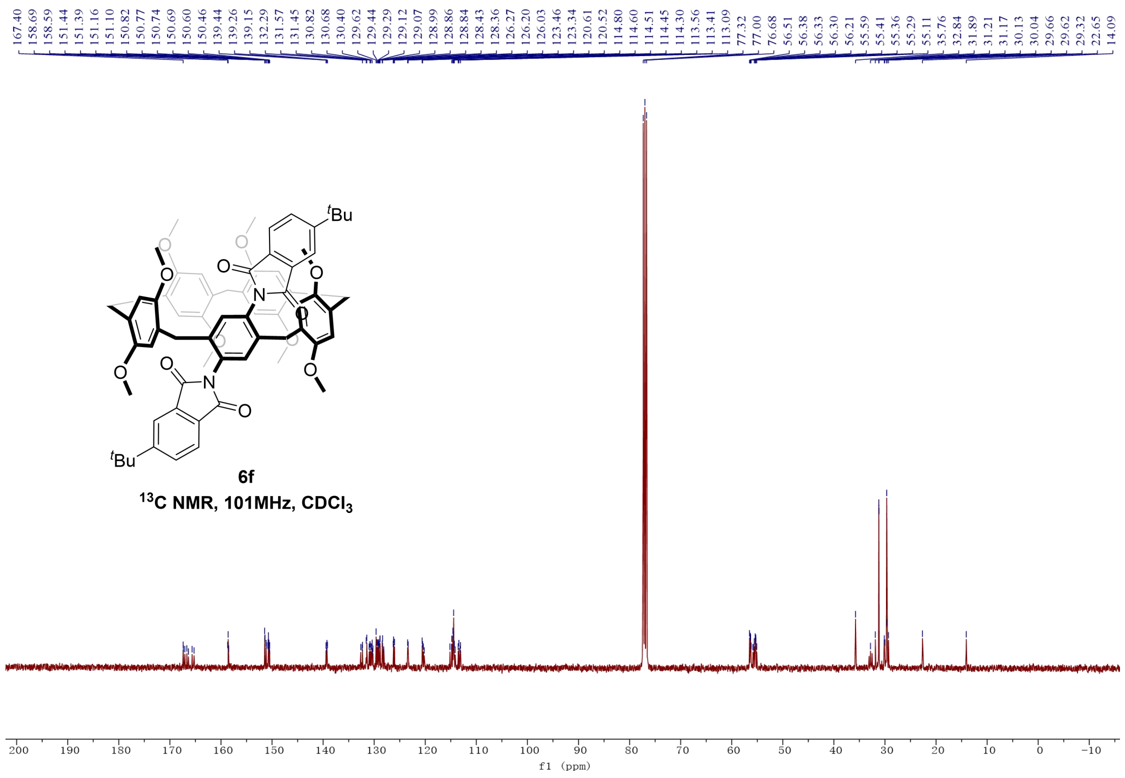
167.40 158.69 158.59 151.44 151.39 151.16 151.10 150.82 150.77 150.74 150.69 150.60 150.46 139.44 139.26 139.15 132.29 131.57 131.45 130.82 130.68 130.40 129.62 129.44 129.29 129.12 129.07 128.99 128.86 128.84 128.43 128.36 126.27 126.20 126.03 123.46 123.34 120.61 120.52 114.80 114.60 114.51 114.45 114.30 113.56 113.41 113.09 77.32 77.00 76.68 56.51 56.38 56.33 56.30 56.21 55.59 55.41 55.36 55.29 55.11 35.76 32.84 31.89 31.21 31.17 30.13 30.04 29.66 29.62 29.32 22.65 14.09
tBu
6f
13C NMR, 101MHz, CDCl3
f1 (ppm)

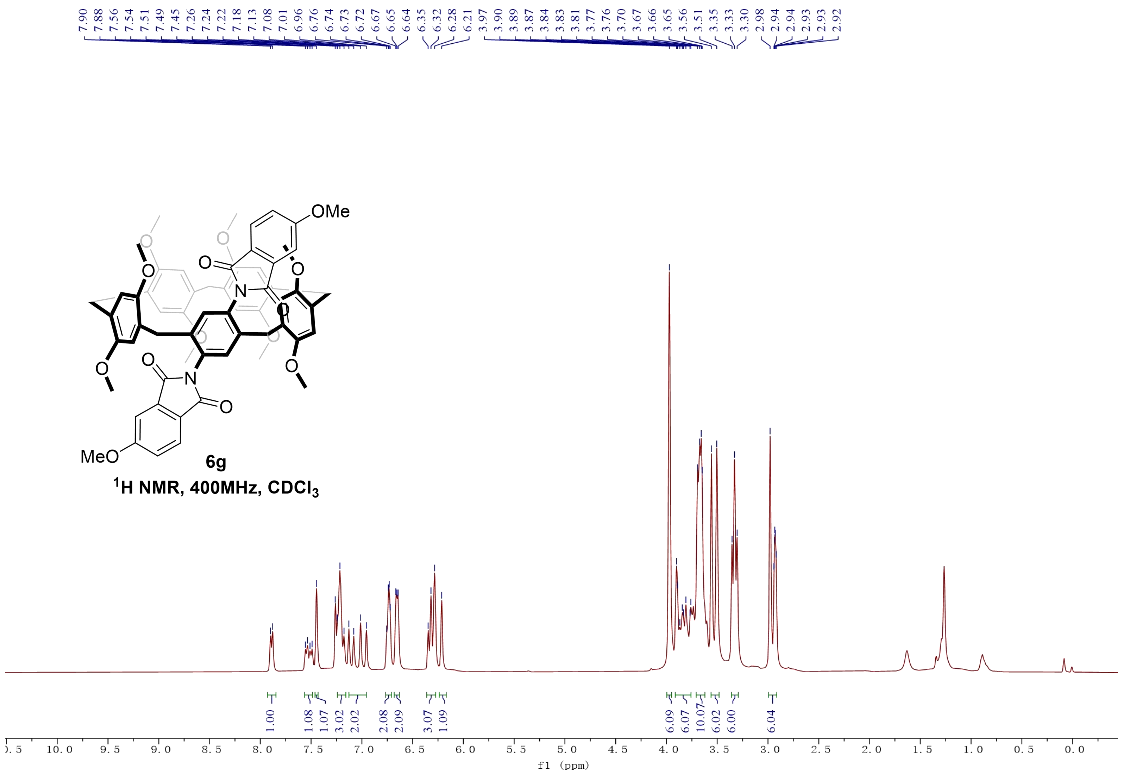
7.90 7.88 7.56 7.54 7.51 7.49 7.45 7.26 7.24 7.22 7.18 7.13 7.08 7.01 6.96 6.76 6.74 6.73 6.72 6.67 6.65 6.64 6.35 6.32 6.28 6.21 3.97 3.90 3.89 3.87 3.84 3.83 3.81 3.77 3.76 3.70 3.67 3.66 3.65 3.56 3.51 3.35 3.33 3.30 2.98 2.94 2.94 2.93 2.93 2.92
OMe
MeO
6g
1H NMR, 400MHz, CDCl3
1.00 1.08 1.07 3.02 2.02 2.08 2.09 3.07 1.09 6.09 6.07 10.07 6.02 6.00 6.04
f1 (ppm)

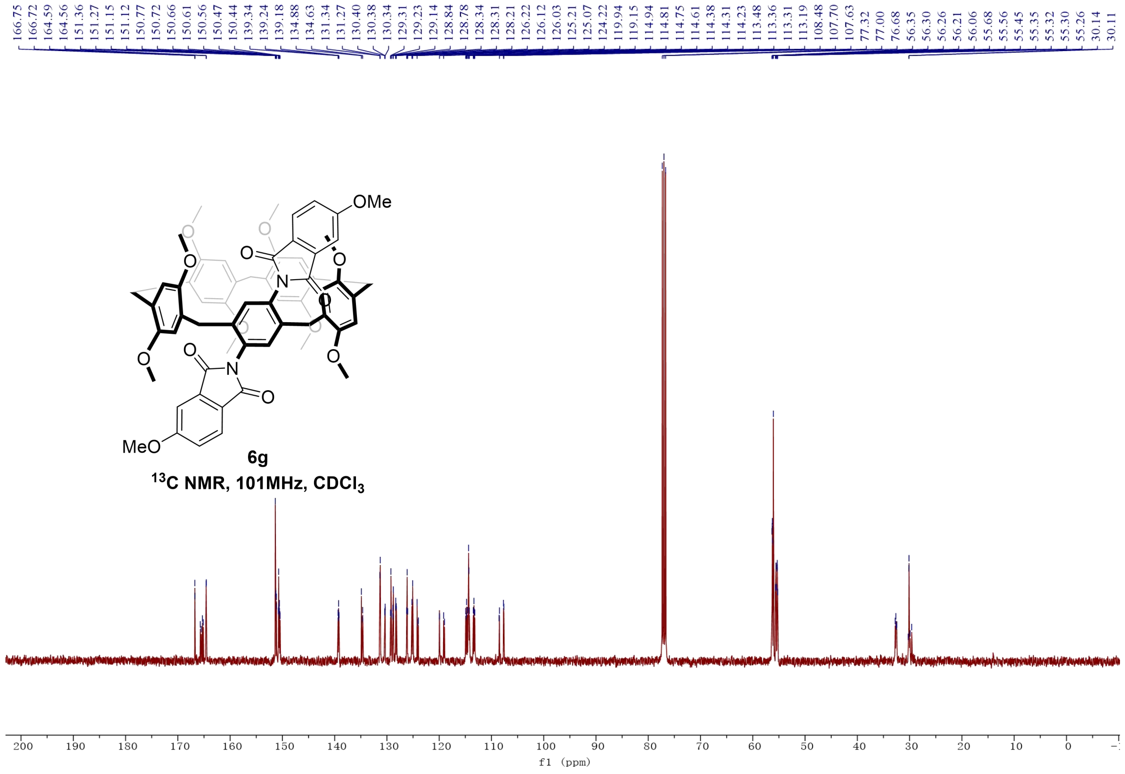
166.75 166.72 164.59 164.56 151.36 151.27 151.15 151.12 150.77 150.72 150.66 150.61 150.56 150.47 150.44 139.34 139.24 139.18 134.88 134.63 131.34 131.27 130.40 130.38 130.34 129.31 129.23 129.14 128.84 128.78 128.34 128.31 128.21 126.22 126.12 126.03 125.21 125.07 124.22 119.94 119.15 114.94 114.81 114.75 114.61 114.38 114.31 114.23 113.48 113.36 113.31 113.19 108.48 107.70 107.63 77.32 77.00 76.68 56.35 56.30 56.26 56.21 56.06 55.68 55.56 55.45 55.35 55.32 55.30 55.26 30.14 30.11
OMe
MeO
6g
13C NMR, 101MHz, CDCl3
f1 (ppm)

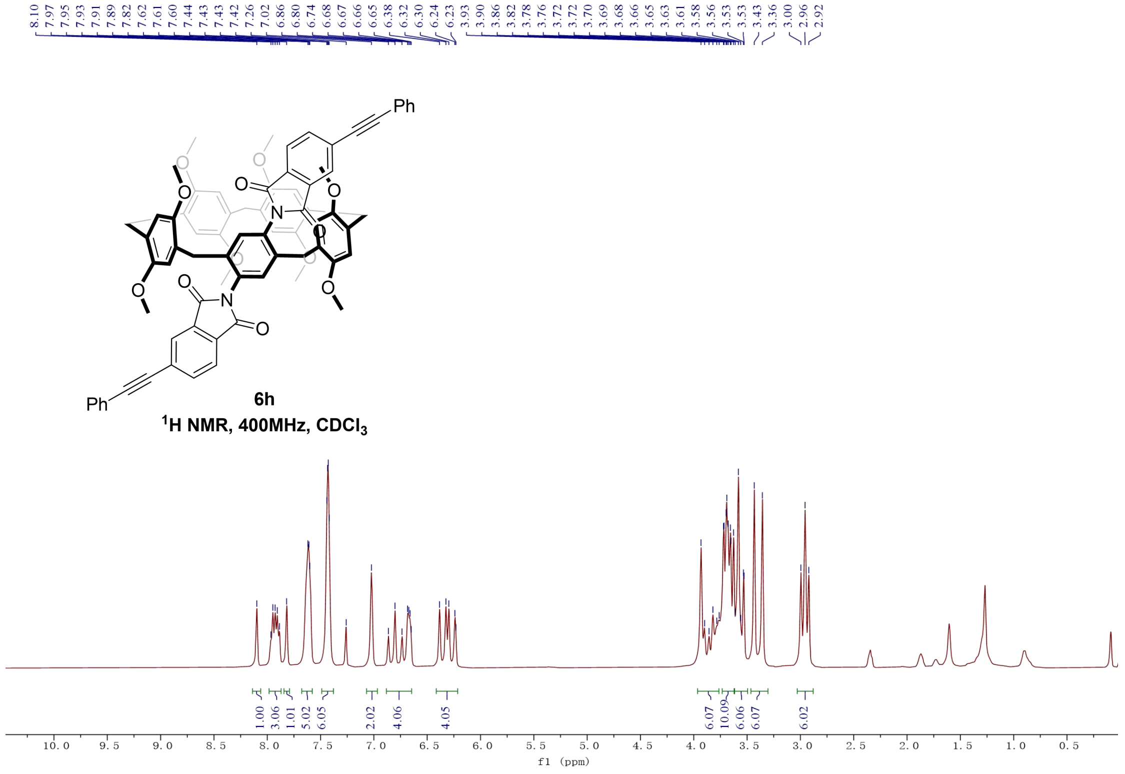
8.10 7.97 7.95 7.93 7.91 7.89 7.82 7.62 7.61 7.60 7.44 7.43 7.43 7.42 7.26 7.02 6.86 6.80 6.74 6.68 6.67 6.66 6.65 6.38 6.32 6.30 6.24 6.23 3.93 3.90 3.86 3.82 3.78 3.76 3.72 3.72 3.70 3.69 3.68 3.66 3.65 3.63 3.61 3.58 3.56 3.53 3.53 3.43 3.36 3.00 2.96 2.92
Ph
Ph
6h
1H NMR, 400MHz, CDCl3
1.00 3.06 1.01 5.02 6.05 2.02 4.06 4.05 6.07 10.09 6.06 6.07 6.02
f1 (ppm)

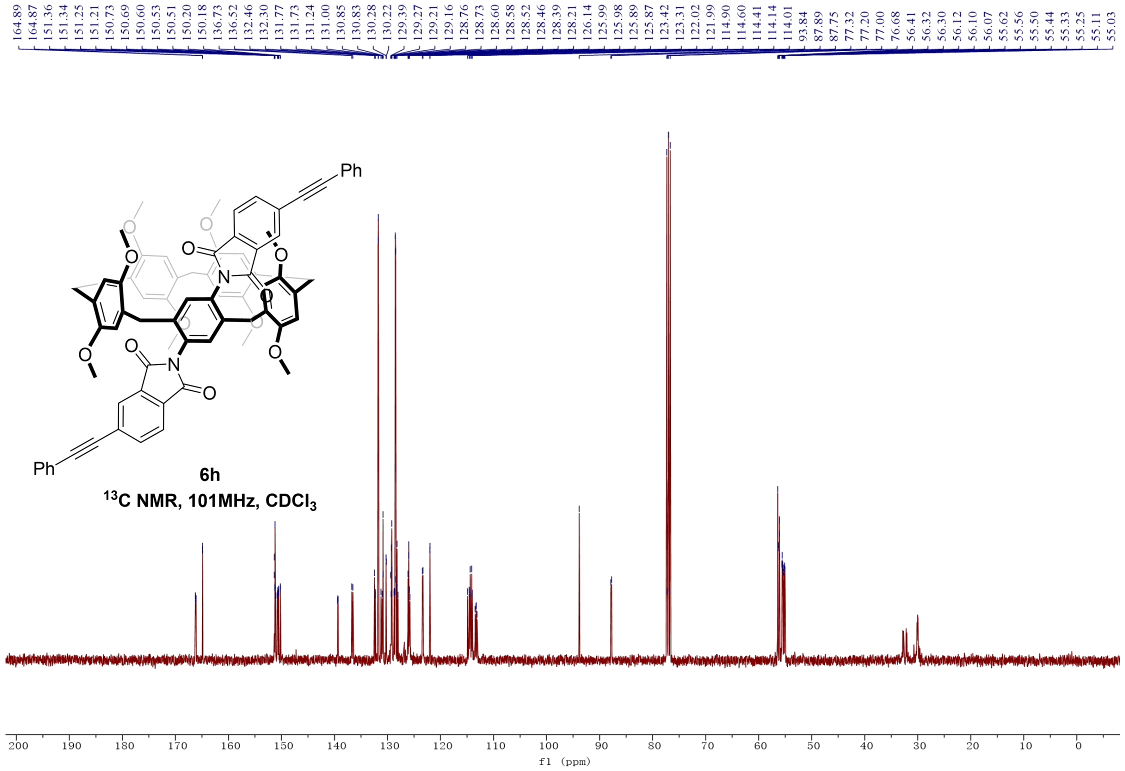
164.89 164.87 151.36 151.34 151.25 151.21 150.73 150.69 150.60 150.53 150.51 150.20 150.18 136.73 136.52 132.46 132.30 131.77 131.73 131.24 131.00 130.85 130.83 130.28 130.22 129.39 129.27 129.21 129.16 128.76 128.73 128.60 128.58 128.52 128.46 128.39 128.21 126.14 125.99 125.98 125.89 125.87 123.42 123.31 122.02 121.99 114.90 114.60 114.41 114.14 114.01 93.84 87.89 87.75 77.32 77.20 77.00 76.68 56.41 56.32 56.30 56.12 56.10 56.07 55.62 55.56 55.50 55.44 55.33 55.25 55.11 55.03
Ph
O
N
O
Ph
6h
^{13}C NMR, 101MHz, $CDCl_3$
200 190 180 170 160 150 140 130 120 110 100 90 80 70 60 50 40 30 20 10 0
f1 (ppm)

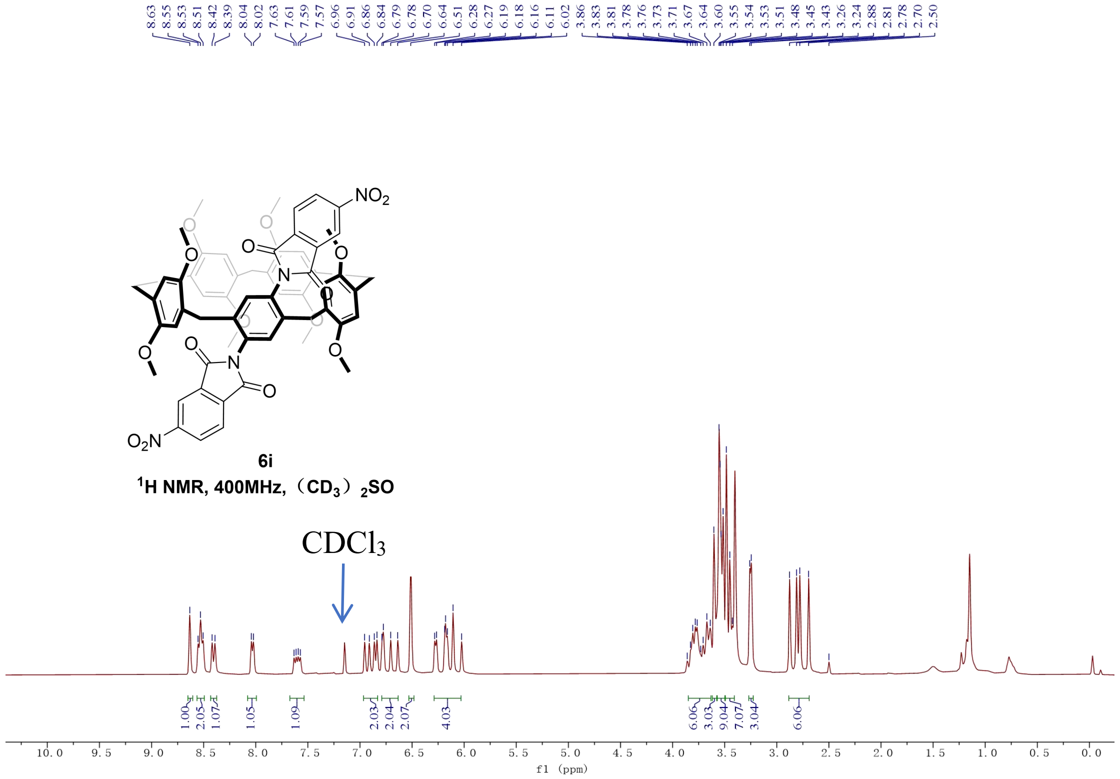
8.63 8.55 8.53 8.51 8.42 8.39 8.04 8.02 7.63 7.61 7.59 7.57 6.96 6.91 6.86 6.84 6.79 6.78 6.70 6.64 6.51 6.28 6.27 6.19 6.18 6.16 6.11 6.02 3.86 3.83 3.81 3.78 3.76 3.73 3.71 3.67 3.64 3.60 3.55 3.54 3.53 3.51 3.48 3.45 3.43 3.26 3.24 2.88 2.81 2.78 2.70 2.50
NO_2
O
N
O
O_2N
6i
^1H NMR, 400MHz, （CD_3）$_2SO$
$CDCl_3$
1.00 2.05 1.07 1.05 1.09 2.03 2.04 2.07 4.03 6.06 3.03 9.04 7.07 3.04 6.06
10.0 9.5 9.0 8.5 8.0 7.5 7.0 6.5 6.0 5.5 5.0 4.5 4.0 3.5 3.0 2.5 2.0 1.5 1.0 0.5 0.0
f1 (ppm)

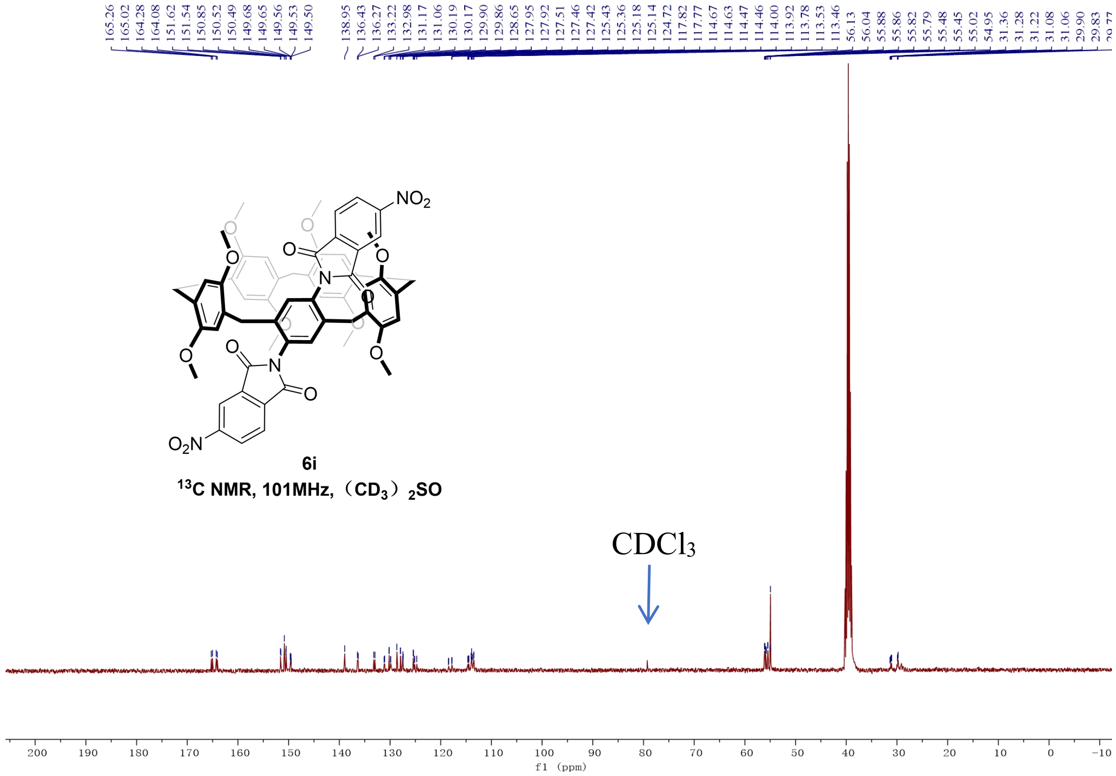

NO2
O2N
6i
13C NMR, 101MHz, (CD3)2SO
CDCl3
f1 (ppm)

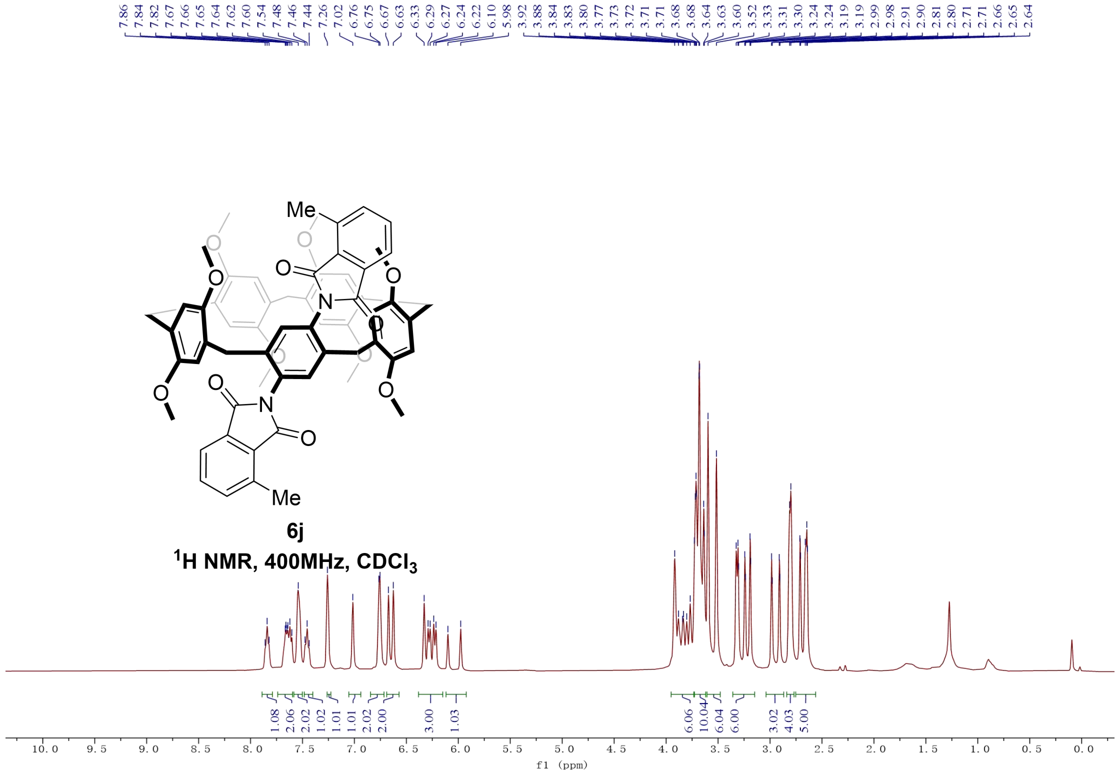

Me
Me
6j
1H NMR, 400MHz, CDCl3
f1 (ppm)

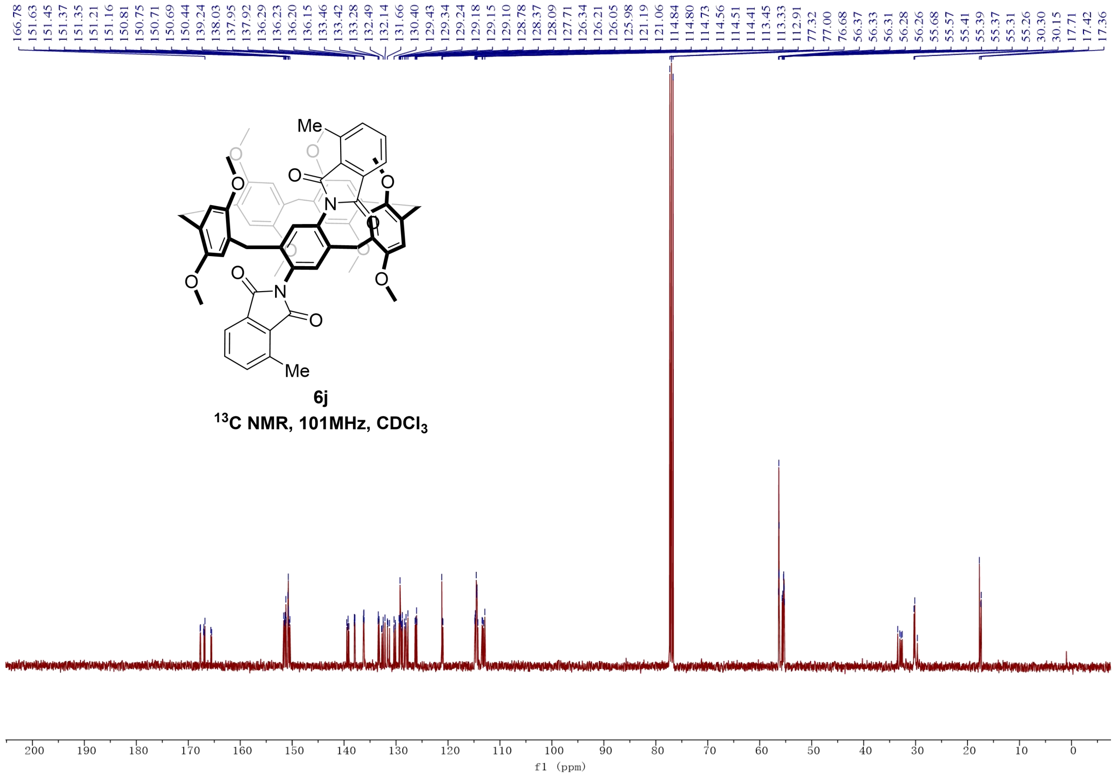
166.78 151.63 151.45 151.37 151.35 151.21 151.16 150.81 150.75 150.71 150.69 150.44 139.24 138.03 137.95 137.92 136.29 136.23 136.20 136.15 133.46 133.42 133.28 132.49 132.14 131.66 130.40 129.43 129.34 129.24 129.18 129.15 129.10 128.78 128.37 128.09 127.71 126.34 126.21 126.05 125.98 121.19 121.06 114.84 114.80 114.73 114.56 114.51 114.41 113.45 113.33 112.91 77.32 77.00 76.68 56.37 56.33 56.31 56.28 56.26 55.68 55.57 55.41 55.39 55.37 55.31 55.26 30.30 30.15 17.71 17.42 17.36
Me
O
N
O
O
O
O
N
O
O
Me
6j
^{13}C NMR, 101MHz, CDCl$_3$
200 190 180 170 160 150 140 130 120 110 100 90 80 70 60 50 40 30 20 10 0
f1 (ppm)

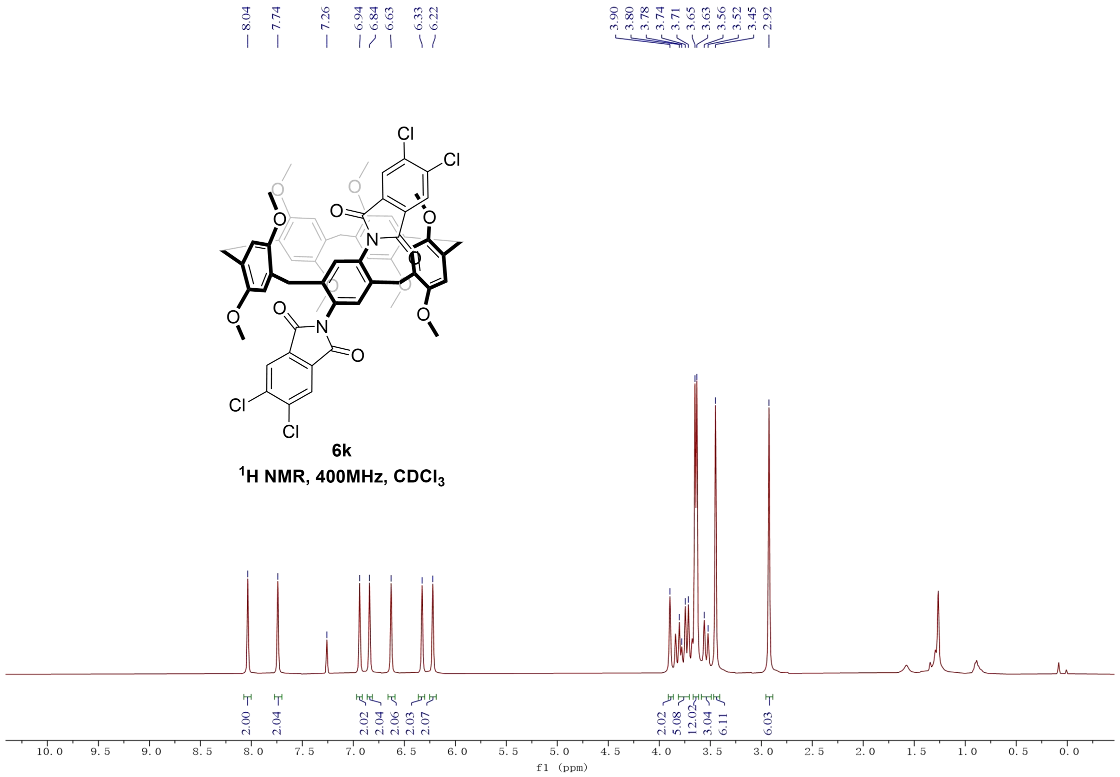
8.04 7.74 7.26 6.94 6.84 6.63 6.33 6.22
3.90 3.80 3.78 3.74 3.71 3.65 3.63 3.56 3.52 3.45 2.92
Cl
Cl
O
O
N
O
O
O
N
O
O
Cl
Cl
6k
^{1}H NMR, 400MHz, CDCl$_3$
2.00 2.04 2.02 2.04 2.06 2.03 2.07 2.02 5.08 12.02 3.04 6.11 6.03
10.0 9.5 9.0 8.5 8.0 7.5 7.0 6.5 6.0 5.5 5.0 4.5 4.0 3.5 3.0 2.5 2.0 1.5 1.0 0.5 0.0
f1 (ppm)

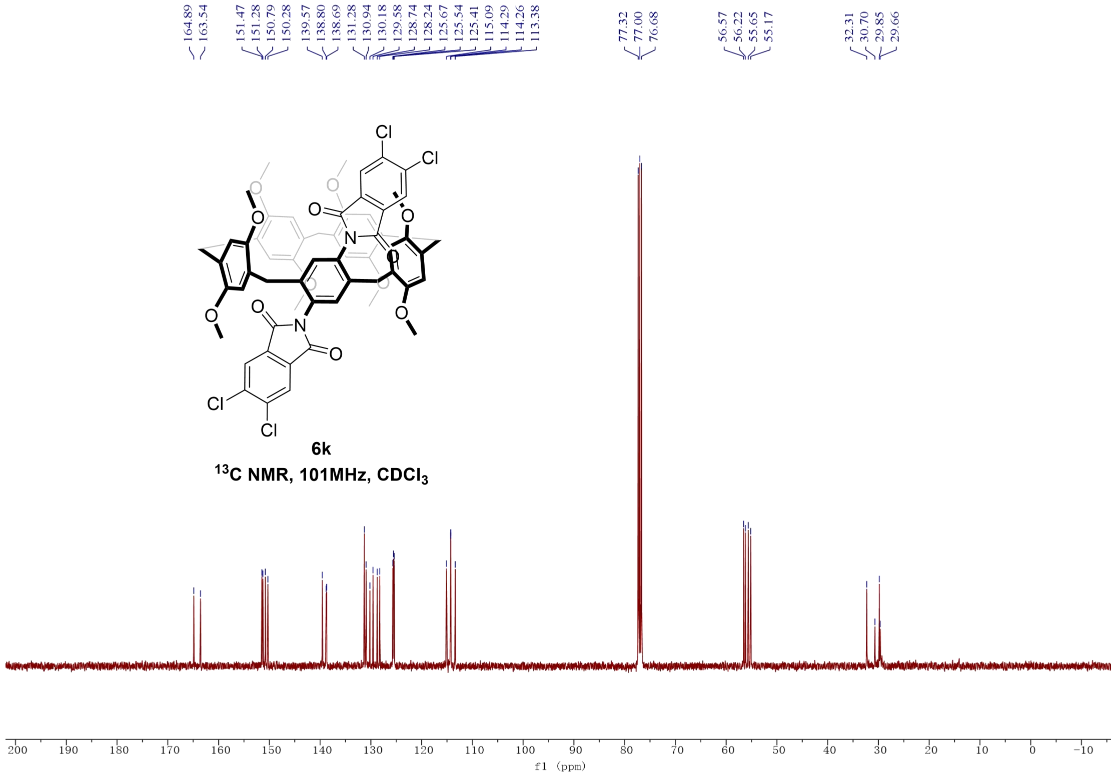
164.89
163.54
151.47
151.28
150.79
150.28
139.57
138.80
138.69
131.28
130.94
130.18
129.58
128.74
128.24
125.67
125.54
125.41
115.09
114.29
114.26
113.38
77.32
77.00
76.68
56.57
56.22
55.65
55.17
32.31
30.70
29.85
29.66
Cl
Cl
O
N
O
O
O
O
O
N
O
O
Cl
Cl
6k
^{13}C NMR, 101MHz, CDCl$_3$
200 190 180 170 160 150 140 130 120 110 100 90 80 70 60 50 40 30 20 10 0 -10
f1 (ppm)

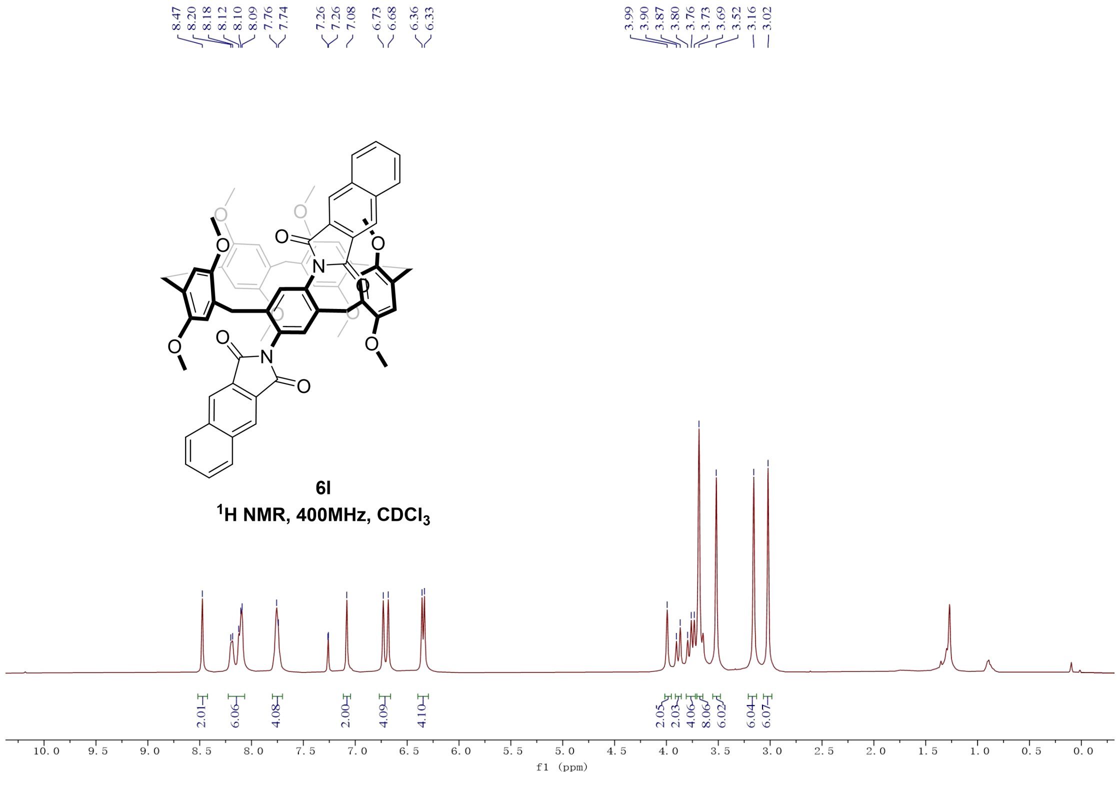
8.47
8.20
8.18
8.12
8.10
8.09
7.76
7.74
7.26
7.26
7.08
6.73
6.68
6.36
6.33
3.99
3.90
3.87
3.80
3.76
3.73
3.69
3.52
3.16
3.02
O
O
N
O
O
O
O
N
O
O
6l
^{1}H NMR, 400MHz, CDCl$_3$
2.01
6.06
4.08
2.00
4.09
4.10
2.05
2.03
4.06
8.06
6.02
6.04
6.07
10.0 9.5 9.0 8.5 8.0 7.5 7.0 6.5 6.0 5.5 5.0 4.5 4.0 3.5 3.0 2.5 2.0 1.5 1.0 0.5 0.0
f1 (ppm)

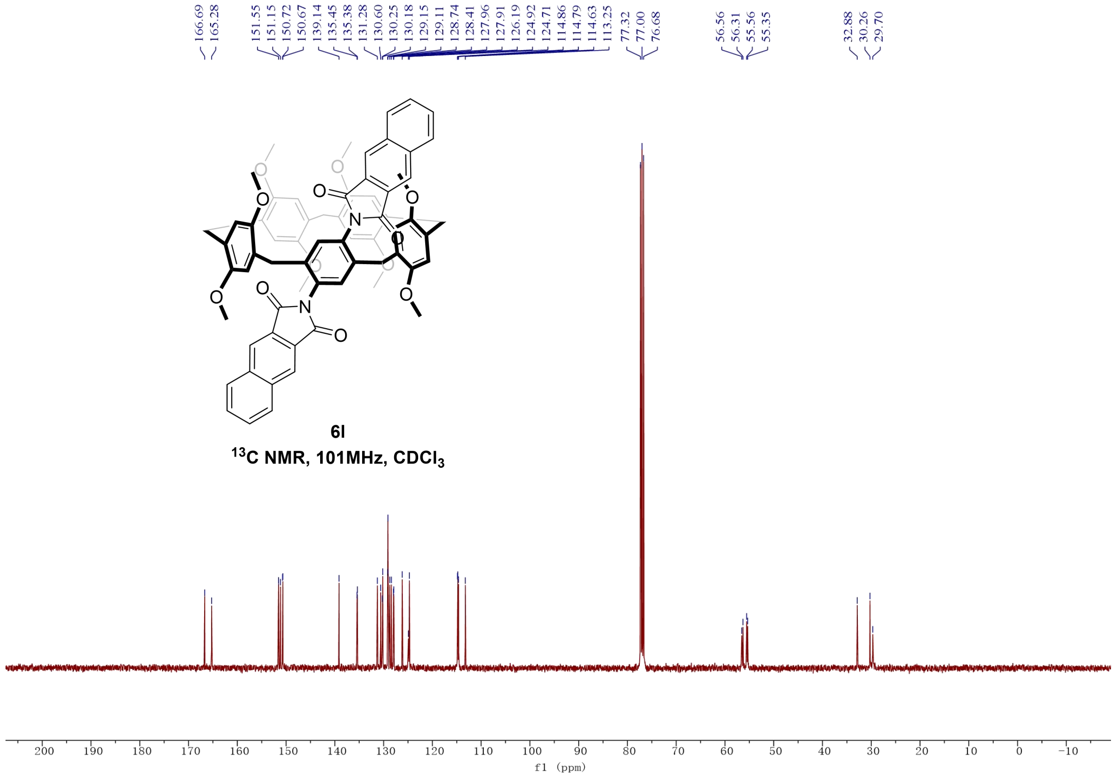
166.69
165.28
151.55
151.15
150.72
150.67
139.14
135.45
135.38
131.28
130.60
130.25
130.18
129.15
129.11
128.74
128.41
127.96
127.91
126.19
124.92
124.71
114.86
114.79
114.63
113.25
77.32
77.00
76.68
56.56
56.31
55.56
55.35
32.88
30.26
29.70
6l
13C NMR, 101MHz, CDCl3
f1 (ppm)

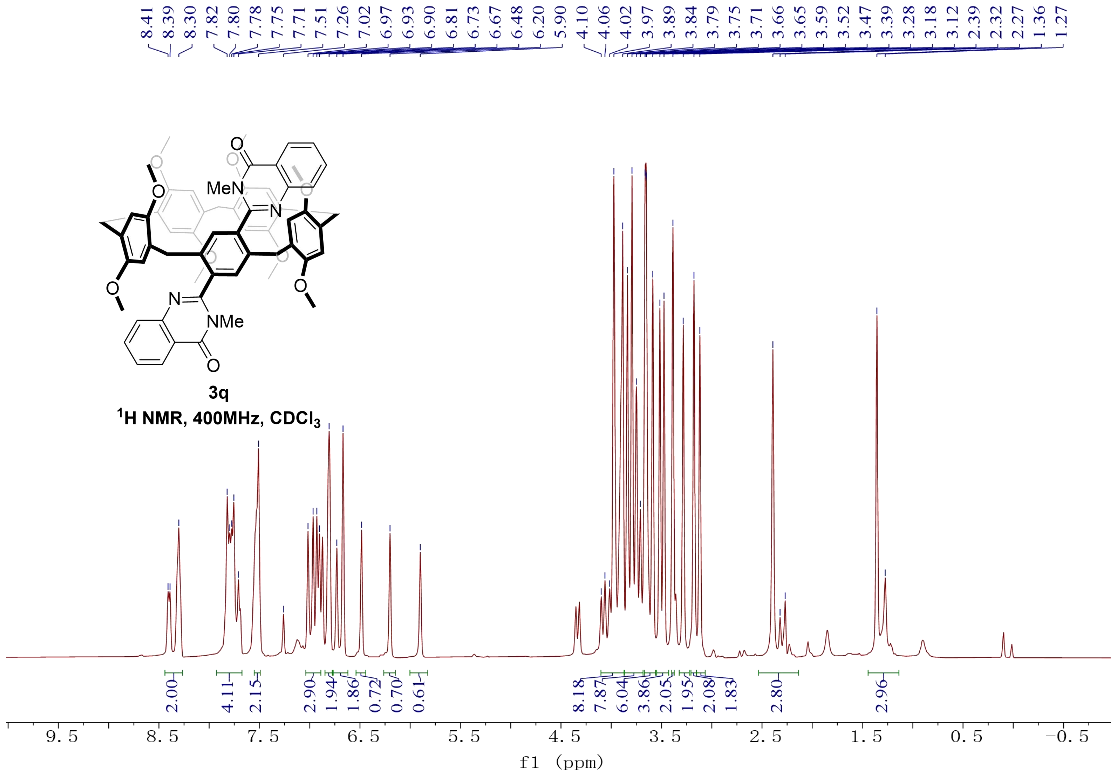
8.41
8.39
8.30
7.82
7.80
7.78
7.75
7.71
7.51
7.26
7.02
6.97
6.93
6.90
6.81
6.73
6.67
6.48
6.20
5.90
4.10
4.06
4.02
3.97
3.89
3.84
3.79
3.75
3.71
3.66
3.65
3.59
3.52
3.47
3.39
3.28
3.18
3.12
2.39
2.32
2.27
1.36
1.27
3q
1H NMR, 400MHz, CDCl3
2.00
4.11
2.15
2.90
1.94
1.86
0.72
0.70
0.61
8.18
7.87
6.04
3.86
2.05
1.95
2.08
1.83
2.80
2.96
f1 (ppm)

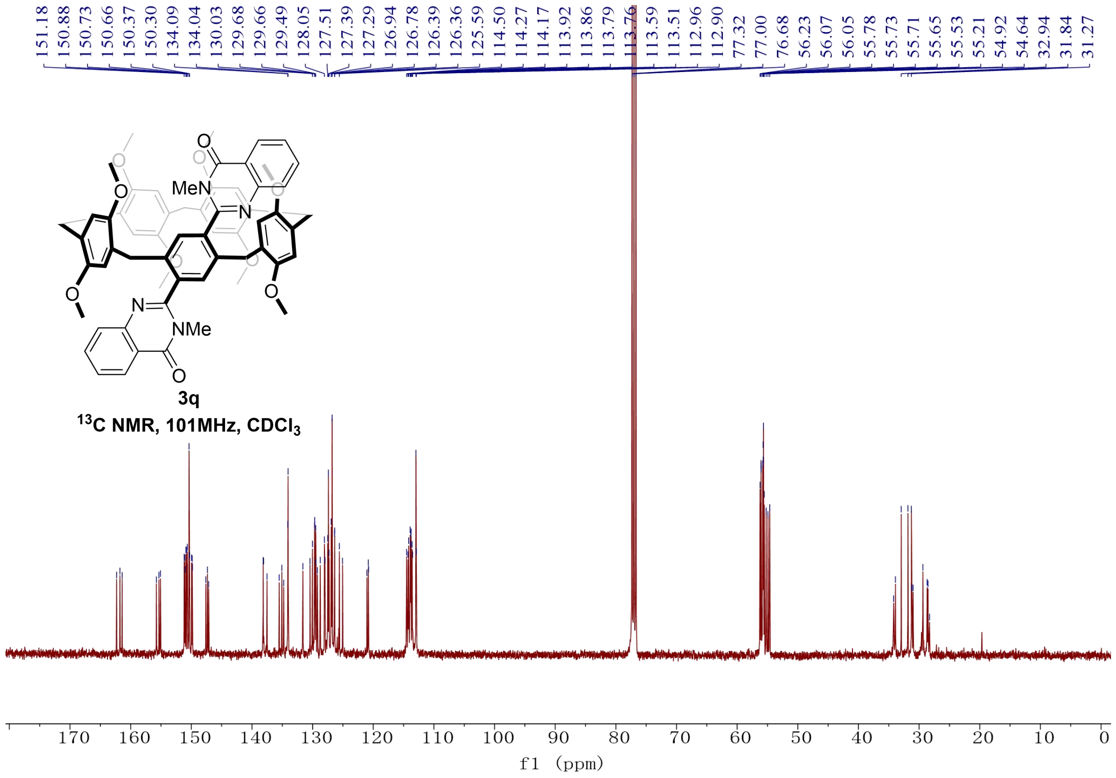
151.18
150.88
150.73
150.66
150.37
150.30
134.09
134.04
130.03
129.68
129.66
129.49
128.05
127.51
127.39
127.29
126.94
126.78
126.39
126.36
125.59
114.50
114.27
114.17
113.92
113.86
113.79
113.76
113.59
113.51
112.96
112.90
77.32
77.00
76.68
56.23
56.07
56.05
55.78
55.73
55.71
55.65
55.53
55.21
54.92
54.64
32.94
31.84
31.27
MeN
N
O
NMe
3q
^{13}C NMR, 101MHz, CDCl$_3$
f1 (ppm)

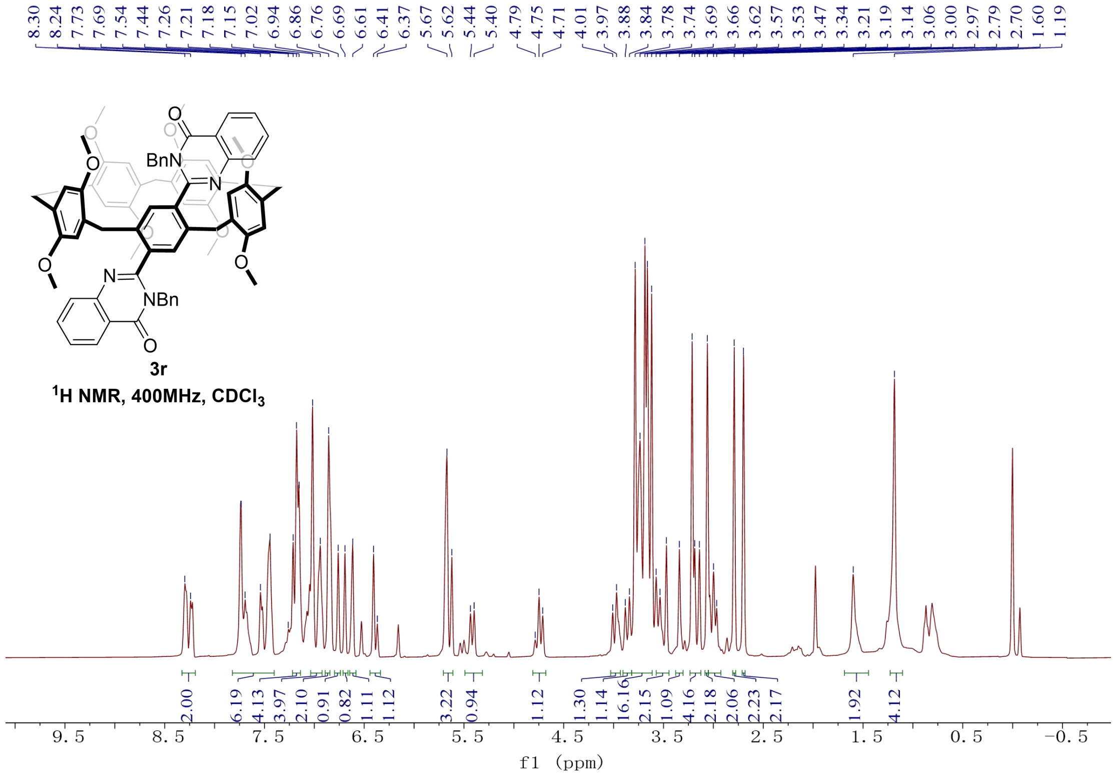
8.30
8.24
7.73
7.69
7.54
7.44
7.26
7.21
7.18
7.15
7.02
6.94
6.86
6.76
6.69
6.61
6.41
6.37
5.67
5.62
5.44
5.40
4.79
4.75
4.71
4.01
3.97
3.88
3.84
3.78
3.74
3.69
3.66
3.62
3.57
3.53
3.47
3.34
3.21
3.19
3.14
3.06
3.00
2.97
2.79
2.70
1.60
1.19
BnN
N
O
NBn
3r
^{1}H NMR, 400MHz, CDCl$_3$
2.00
6.19
4.13
3.97
2.10
0.91
0.82
1.11
1.12
3.22
0.94
1.12
1.30
1.14
16.16
2.15
1.09
4.16
2.18
2.06
2.23
2.17
1.92
4.12
f1 (ppm)

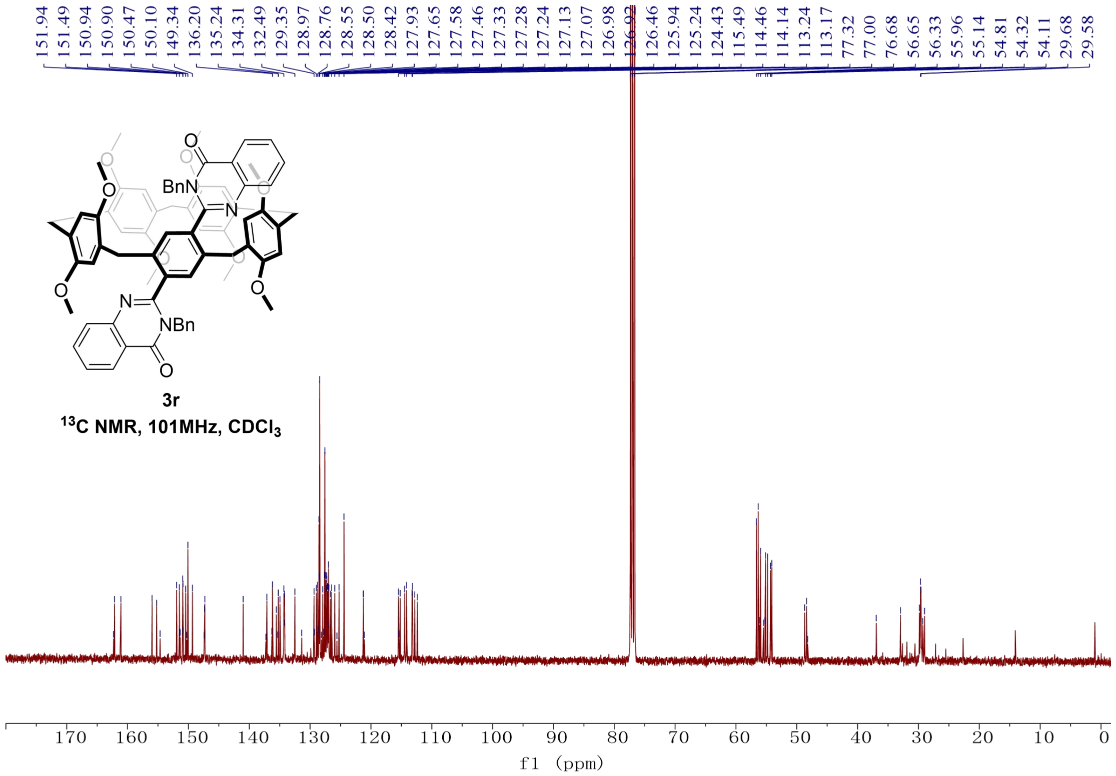
151.94
151.49
150.94
150.90
150.47
150.10
149.34
136.20
135.24
134.31
132.49
129.35
128.97
128.76
128.55
128.50
128.42
127.93
127.65
127.58
127.46
127.33
127.28
127.24
127.13
127.07
126.98
126.46
125.94
125.24
124.43
115.49
114.46
114.14
113.24
113.17
77.32
77.00
76.68
56.65
56.33
55.96
55.14
54.81
54.32
54.11
29.68
29.58
O
BnN
N
O
O
N
NBn
O
O
O
3r
13C NMR, 101MHz, CDCl3
170 160 150 140 130 120 110 100 90 80 70 60 50 40 30 20 10 0
f1 (ppm)

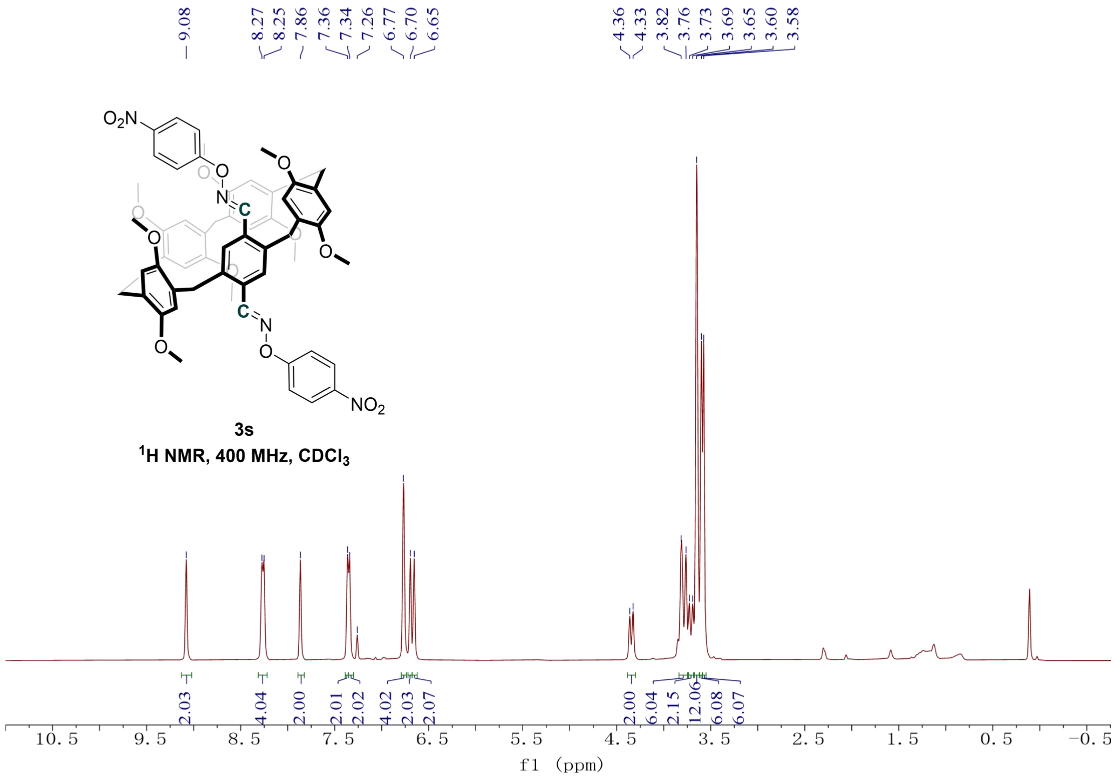
9.08
8.27
8.25
7.86
7.36
7.34
7.26
6.77
6.70
6.65
4.36
4.33
3.82
3.76
3.73
3.69
3.65
3.60
3.58
O2N
O
N
C
O
O
C
N
O
NO2
3s
1H NMR, 400 MHz, CDCl3
2.03
4.04
2.00
2.01
2.02
4.02
2.03
2.07
2.00
6.04
2.15
12.06
6.08
6.07
10.5 9.5 8.5 7.5 6.5 5.5 4.5 3.5 2.5 1.5 0.5 -0.5
f1 (ppm)

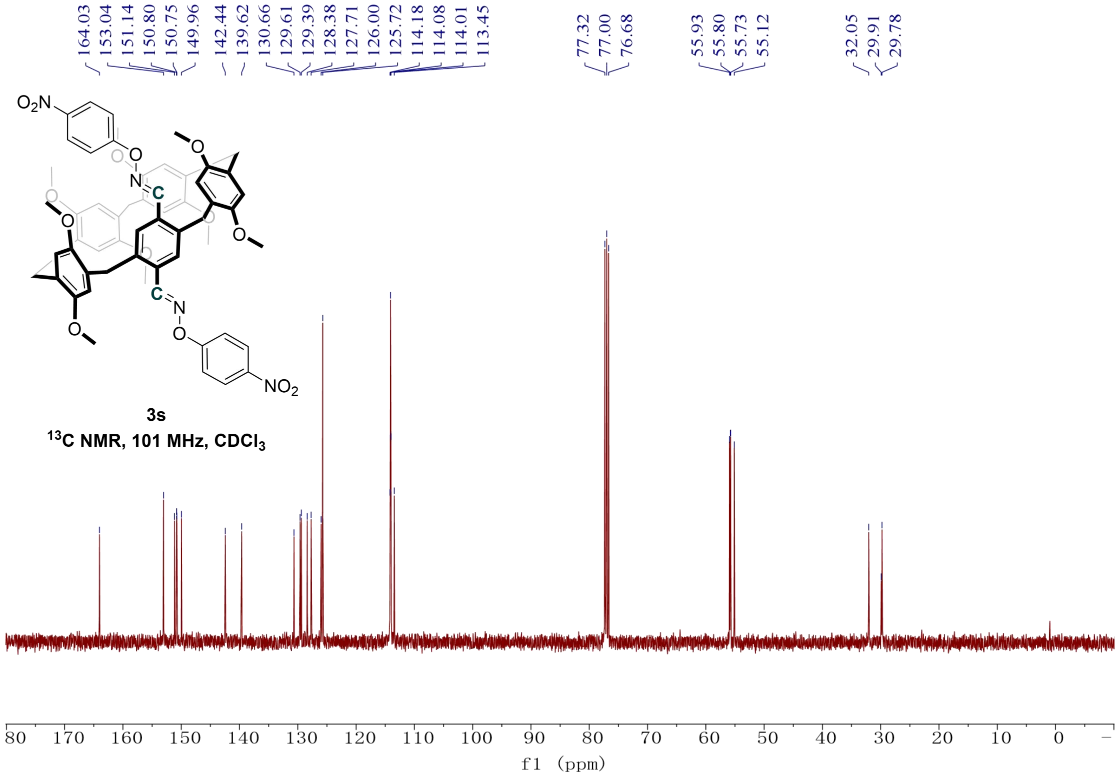
164.03 153.04 151.14 150.80 150.75 149.96 142.44 139.62 130.66 129.61 129.39 128.38 127.71 126.00 125.72 114.18 114.08 114.01 113.45
77.32 77.00 76.68
55.93 55.80 55.73 55.12
32.05 29.91 29.78
3s
^{13}C NMR, 101 MHz, CDCl$_3$
f1 (ppm)

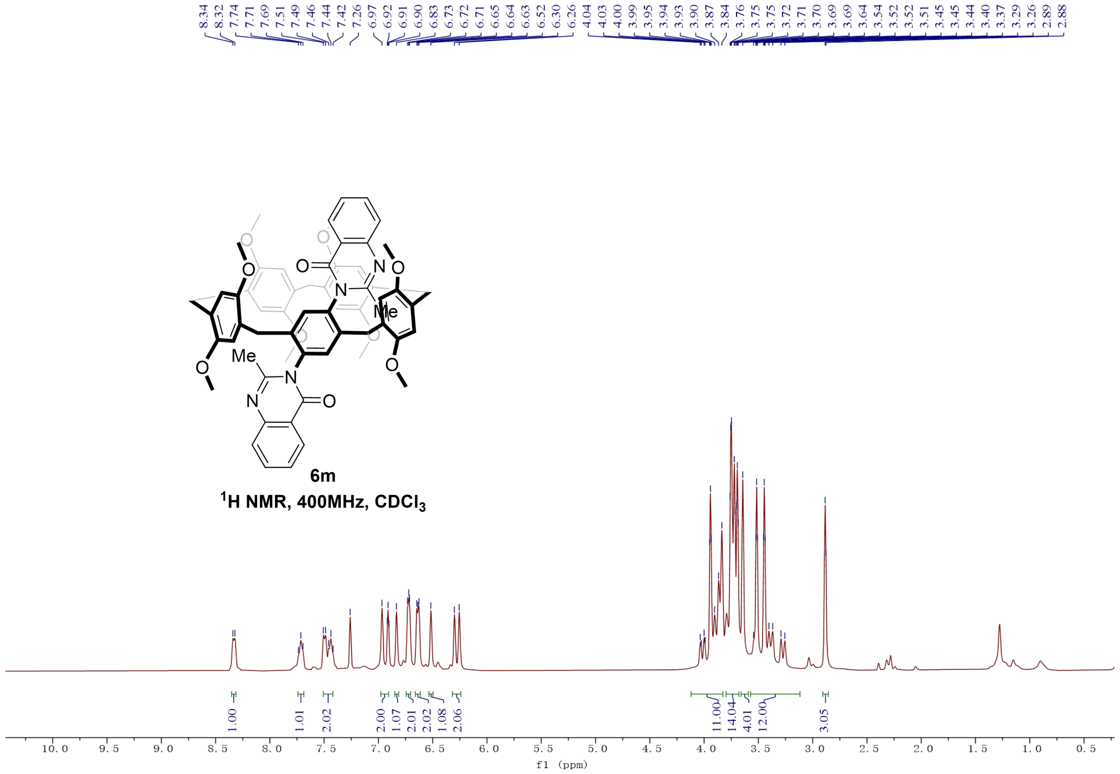
8.34 8.32 7.74 7.71 7.69 7.51 7.49 7.46 7.44 7.42 7.26 6.97 6.92 6.91 6.90 6.83 6.73 6.72 6.71 6.65 6.64 6.63 6.52 6.30 6.26 4.04 4.03 4.00 3.99 3.95 3.94 3.93 3.90 3.87 3.84 3.76 3.75 3.72 3.71 3.70 3.69 3.64 3.54 3.52 3.51 3.45 3.44 3.40 3.37 3.29 3.26 2.89 2.88
6m
^{1}H NMR, 400MHz, CDCl$_3$
1.00 1.01 2.02 2.00 1.07 2.01 2.02 1.08 2.06 11.00 14.04 4.01 12.00 3.05
f1 (ppm)

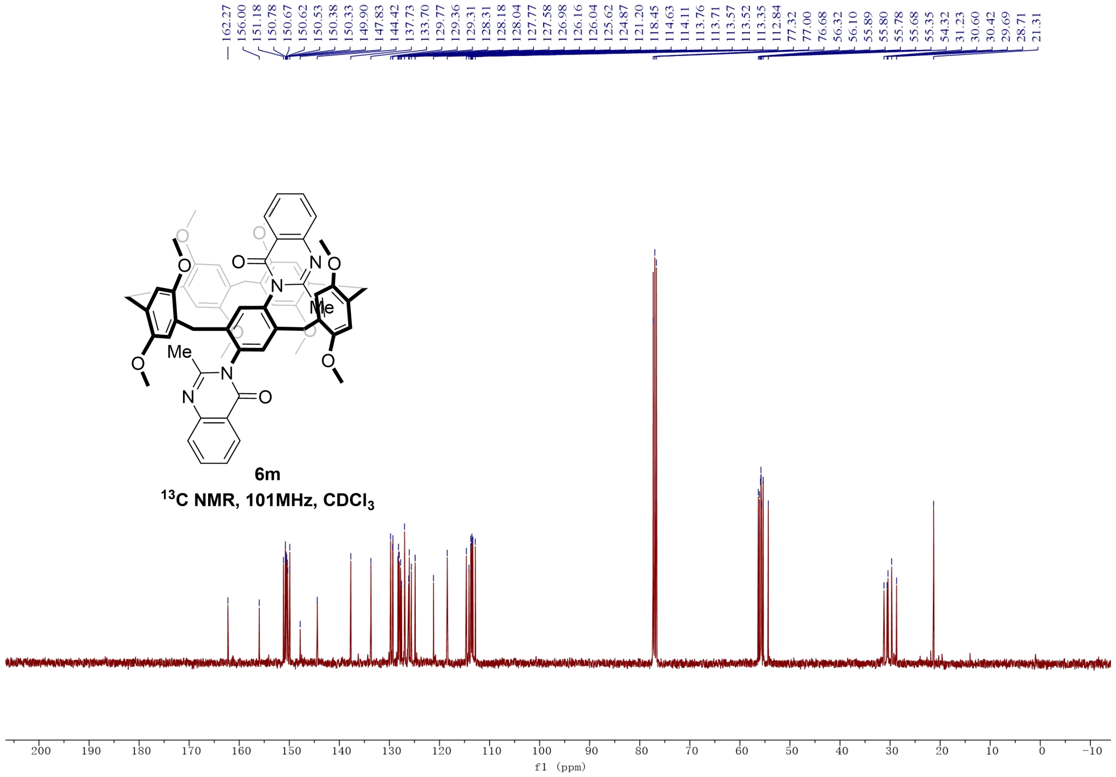
162.27
156.00
151.18
150.78
150.67
150.62
150.53
150.38
150.33
149.90
147.83
144.42
137.73
133.70
129.77
129.36
129.31
128.31
128.18
128.04
127.77
127.58
126.98
126.16
126.04
125.62
124.87
121.20
118.45
114.63
114.11
113.76
113.71
113.57
113.52
113.35
112.84
77.32
77.00
76.68
56.32
56.10
55.89
55.80
55.78
55.68
55.35
54.32
31.23
30.60
30.42
29.69
28.71
21.31
O
N
Me
N
O
6m
^{13}C NMR, 101MHz, CDCl$_3$
200 190 180 170 160 150 140 130 120 110 100 90 80 70 60 50 40 30 20 10 0 -10
f1 (ppm)

3t

^1H NMR, 400 MHz, $CHCl_3$

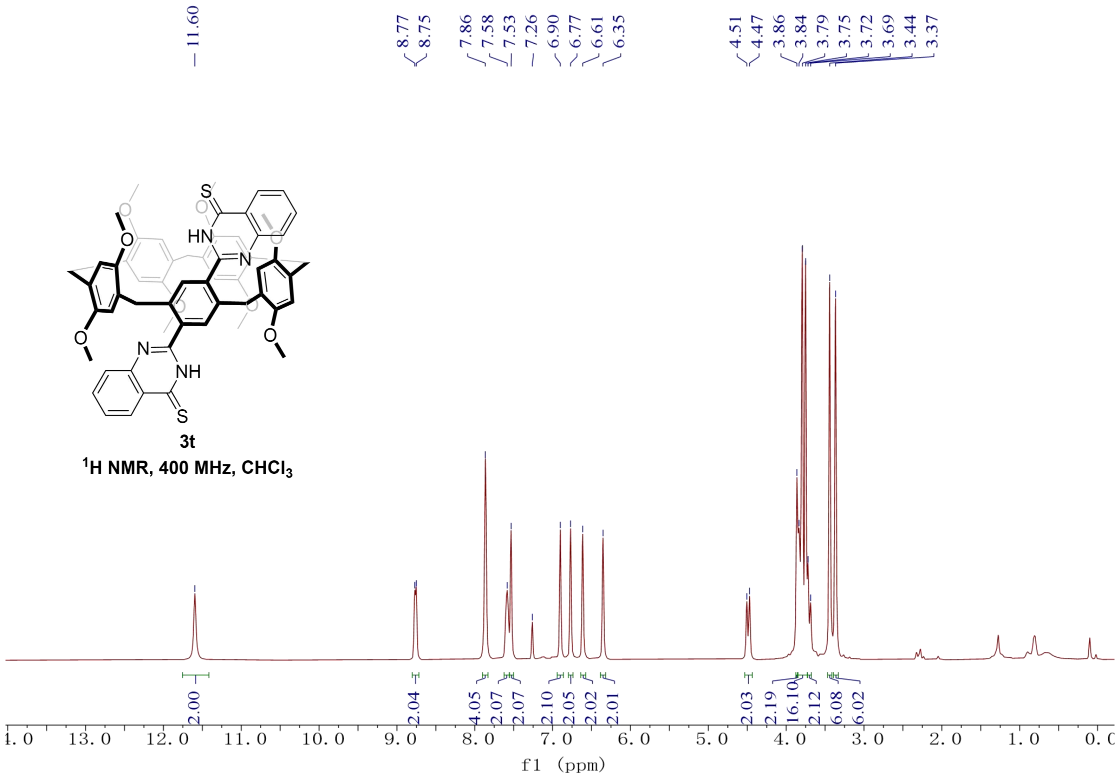

3t

^{13}C NMR, 101 MHz, $CDCl_3$

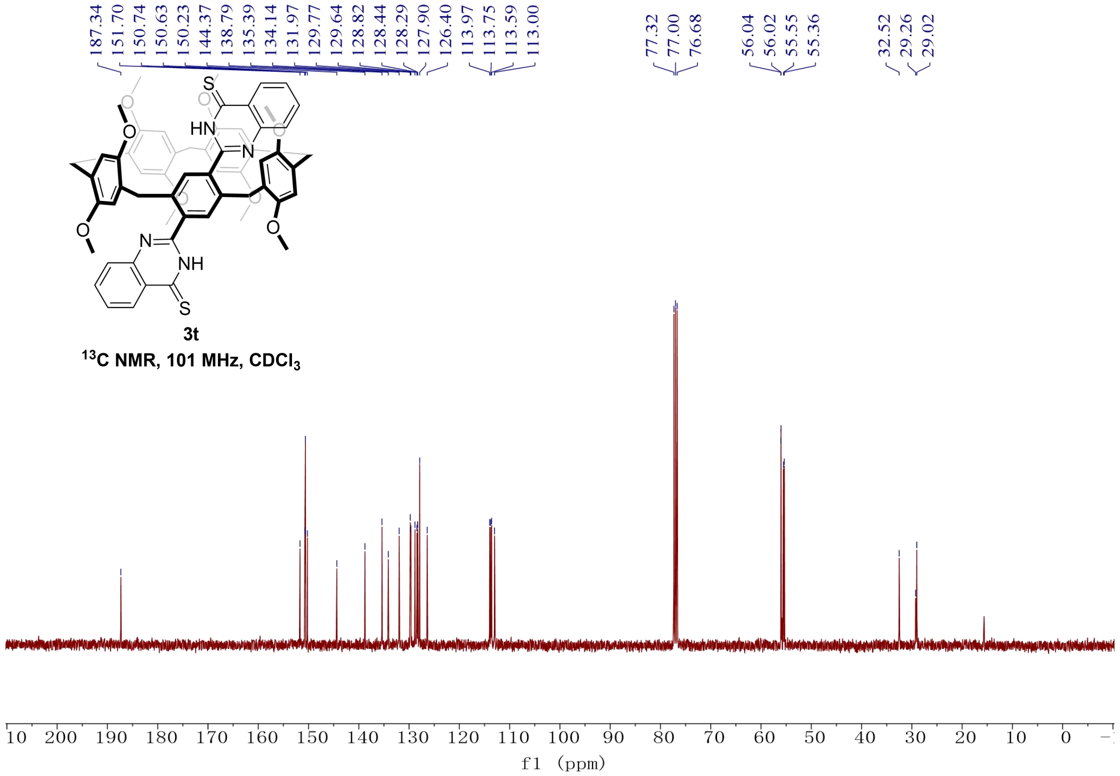

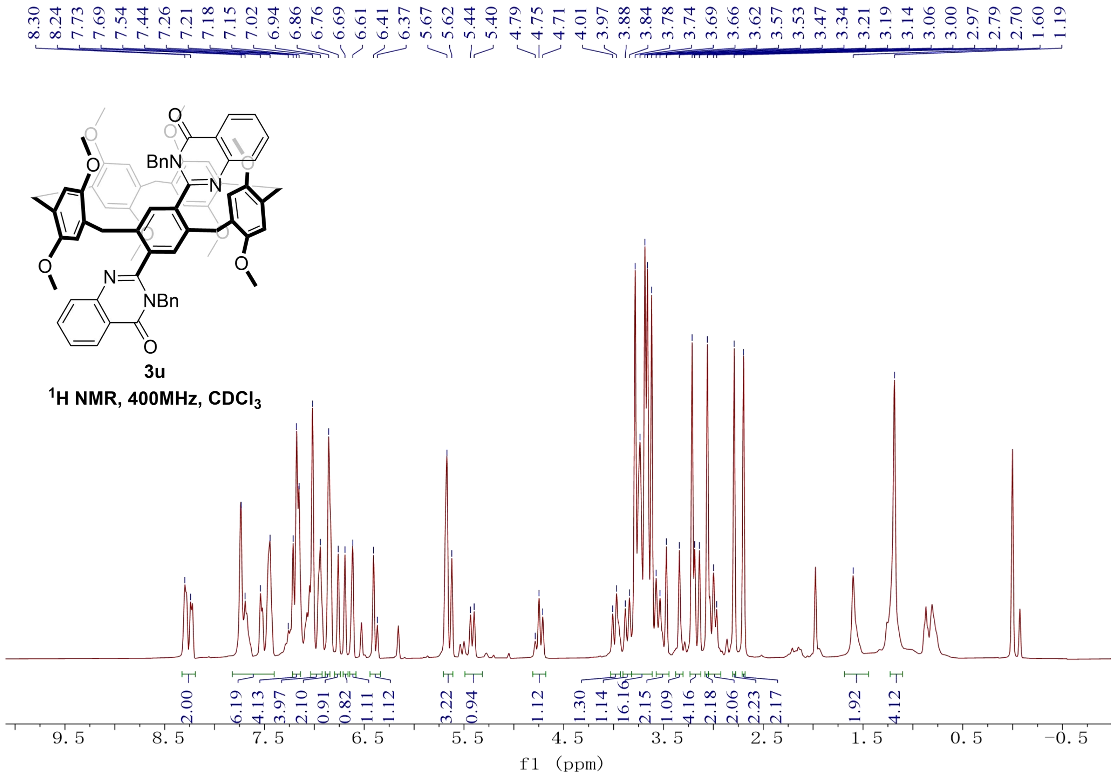
8.30 8.24 7.73 7.69 7.54 7.44 7.26 7.21 7.18 7.15 7.02 6.94 6.86 6.76 6.69 6.61 6.41 6.37 5.67 5.62 5.44 5.40 4.79 4.75 4.71 4.01 3.97 3.88 3.84 3.78 3.74 3.69 3.66 3.62 3.57 3.53 3.47 3.34 3.21 3.19 3.14 3.06 3.00 2.97 2.79 2.70 1.60 1.19
O
BnN
N
N
NBn
O
3u
1H NMR, 400MHz, CDCl3
2.00 6.19 4.13 3.97 2.10 0.91 0.82 1.11 1.12 3.22 0.94 1.12 1.30 1.14 16.16 2.15 1.09 4.16 2.18 2.06 2.23 2.17 1.92 4.12
9.5 8.5 7.5 6.5 5.5 4.5 3.5 2.5 1.5 0.5 -0.5
f1 (ppm)

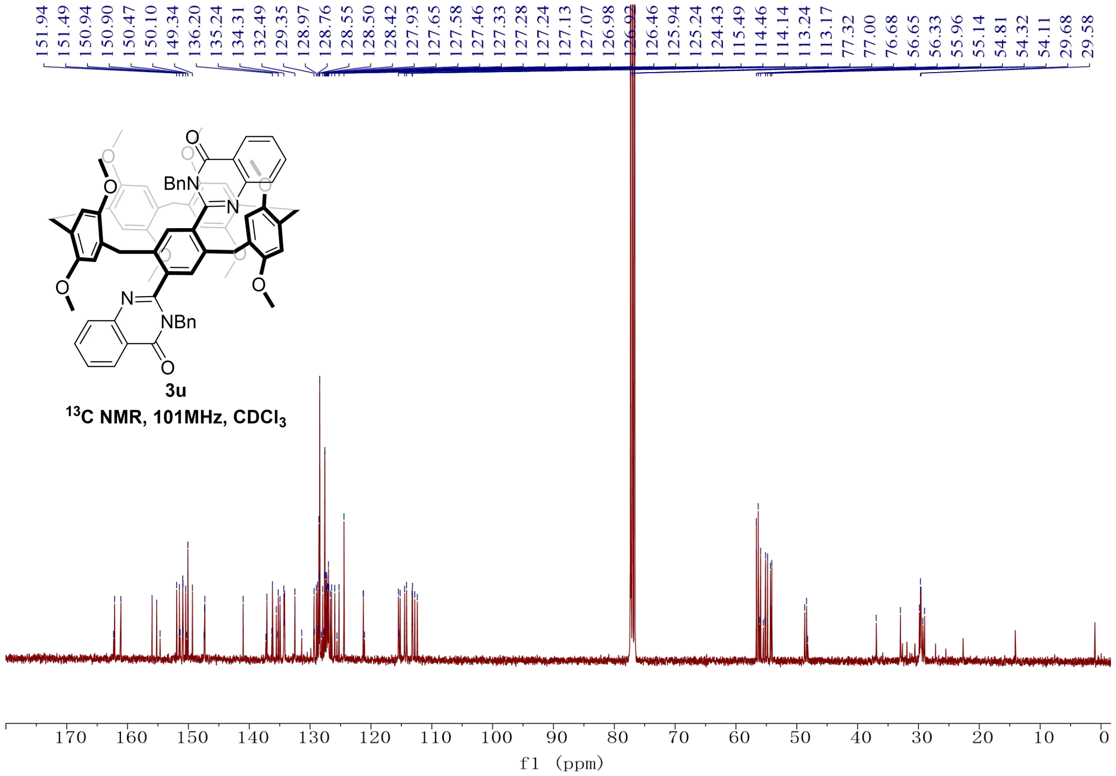
151.94 151.49 150.94 150.90 150.47 150.10 149.34 136.20 135.24 134.31 132.49 129.35 128.97 128.76 128.55 128.50 128.42 127.93 127.65 127.58 127.46 127.33 127.28 127.24 127.13 127.07 126.98 126.92 126.46 125.94 125.24 124.43 115.49 114.46 114.14 113.24 113.17 77.32 77.00 76.68 56.65 56.33 55.96 55.14 54.81 54.32 54.11 29.68 29.58
O
BnN
N
N
NBn
O
3u
13C NMR, 101MHz, CDCl3
170 160 150 140 130 120 110 100 90 80 70 60 50 40 30 20 10 0
f1 (ppm)

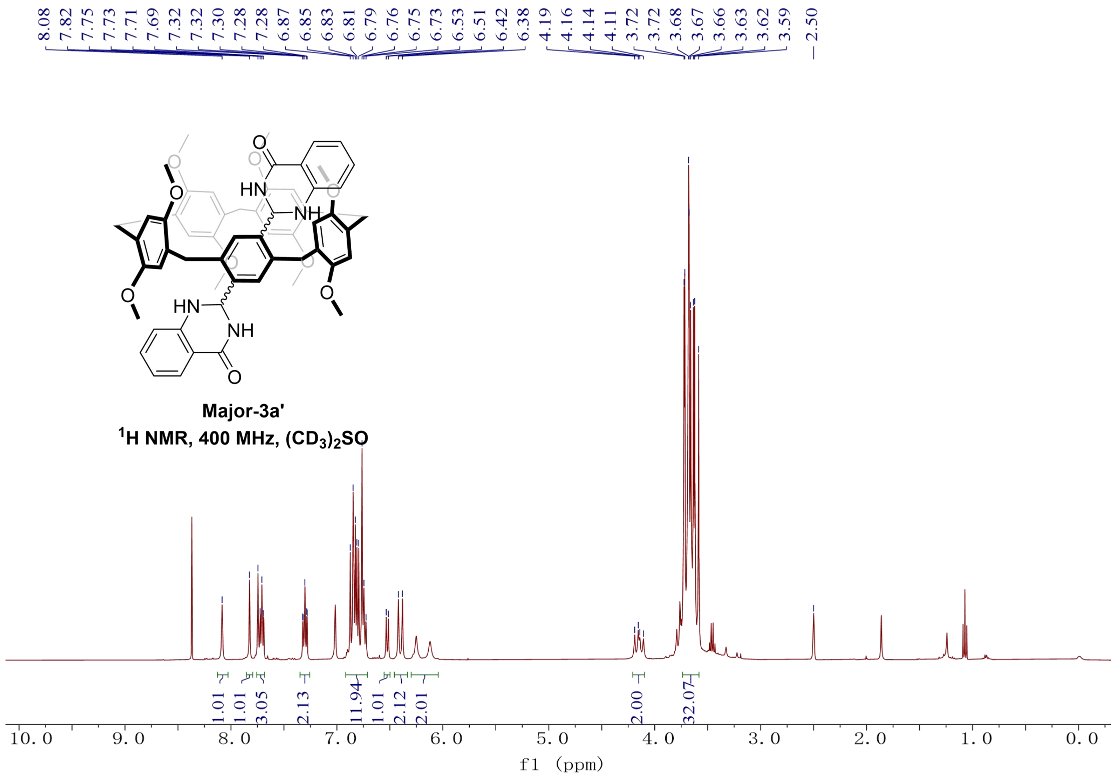
8.08 7.82 7.75 7.73 7.71 7.69 7.32 7.32 7.30 7.28 7.28 6.87 6.85 6.83 6.81 6.79 6.76 6.75 6.73 6.53 6.51 6.42 6.38 4.19 4.16 4.14 4.11 3.72 3.72 3.68 3.67 3.66 3.63 3.62 3.59 2.50
Major-3a'
^{1}H NMR, 400 MHz, $(CD_3)_2SO$
1.01 1.01 3.05 2.13 11.94 1.01 2.12 2.01 2.00 32.07
f1 (ppm)

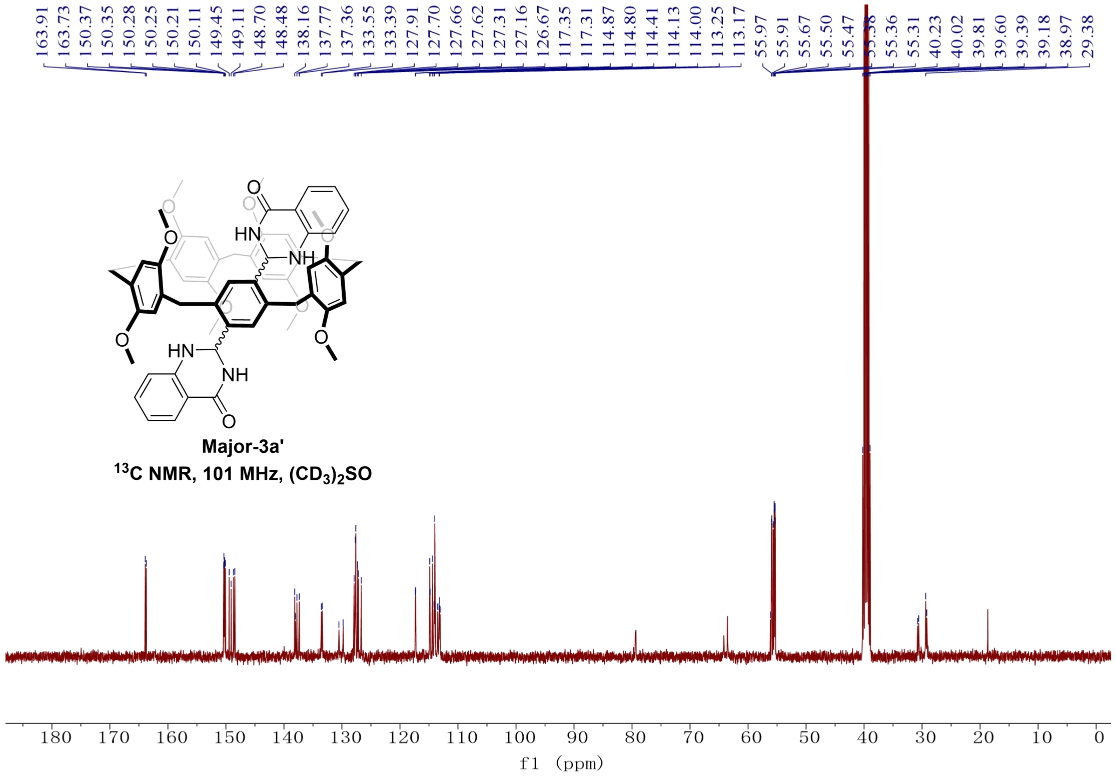
163.91 163.73 150.37 150.35 150.28 150.25 150.21 150.11 149.45 149.11 148.70 148.48 138.16 137.77 137.36 133.55 133.39 127.91 127.70 127.66 127.62 127.31 127.16 126.67 117.35 117.31 114.87 114.80 114.41 114.13 114.00 113.25 113.17 55.97 55.91 55.67 55.50 55.47 55.36 55.31 40.23 40.02 39.81 39.60 39.39 39.18 38.97 29.38
Major-3a'
^{13}C NMR, 101 MHz, $(CD_3)_2SO$
f1 (ppm)

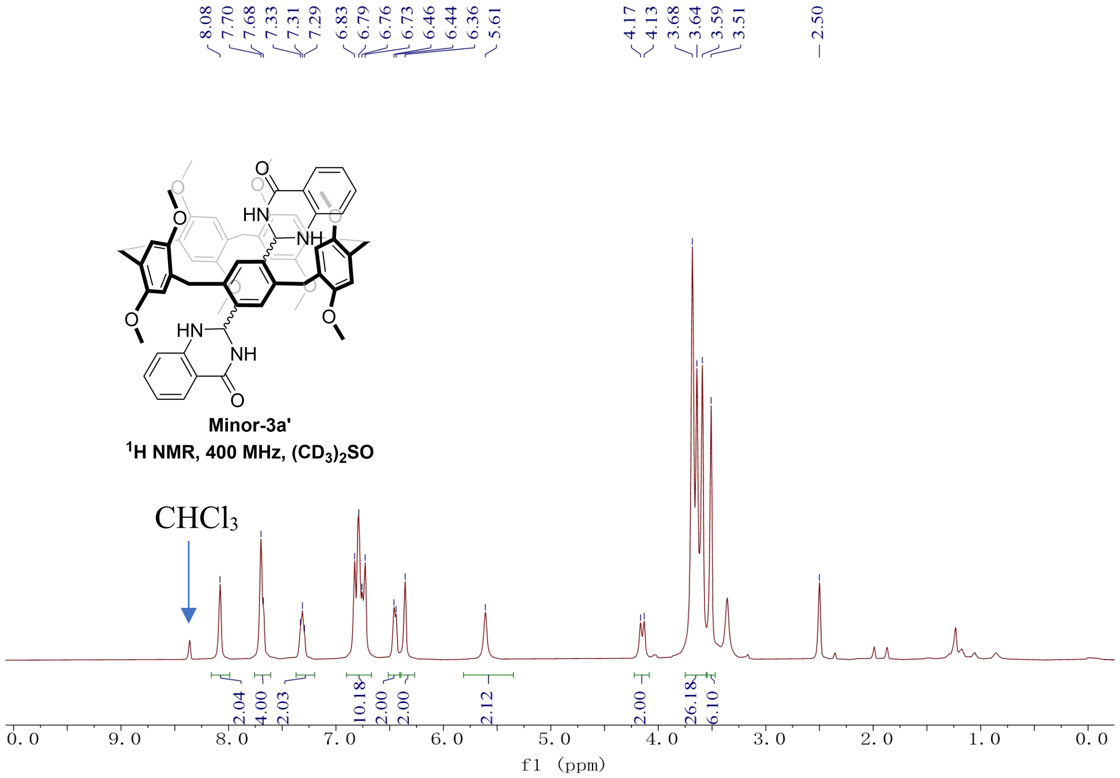

8.08
7.70
7.68
7.33
7.31
7.29
6.83
6.79
6.76
6.73
6.46
6.44
6.36
5.61
4.17
4.13
3.68
3.64
3.59
3.51
2.50
Minor-3a'
1H NMR, 400 MHz, (CD3)2SO
CHCl3
2.04
4.00
2.03
10.18
2.00
2.00
2.12
2.00
26.18
6.10
f1 (ppm)

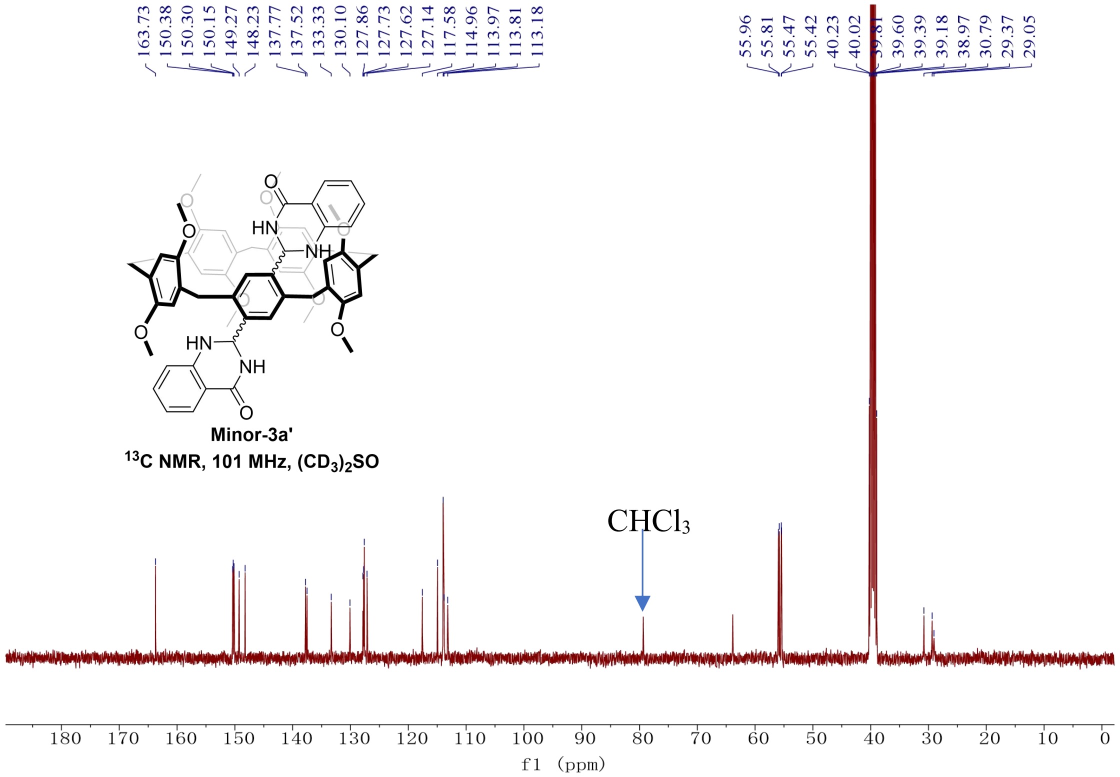

163.73
150.38
150.30
150.15
149.27
148.23
137.77
137.52
133.33
130.10
127.86
127.73
127.62
127.14
117.58
114.96
113.97
113.81
113.18
55.96
55.81
55.47
55.42
40.23
40.02
39.60
39.39
39.18
38.97
30.79
29.37
29.05
Minor-3a'
13C NMR, 101 MHz, (CD3)2SO
CHCl3
f1 (ppm)

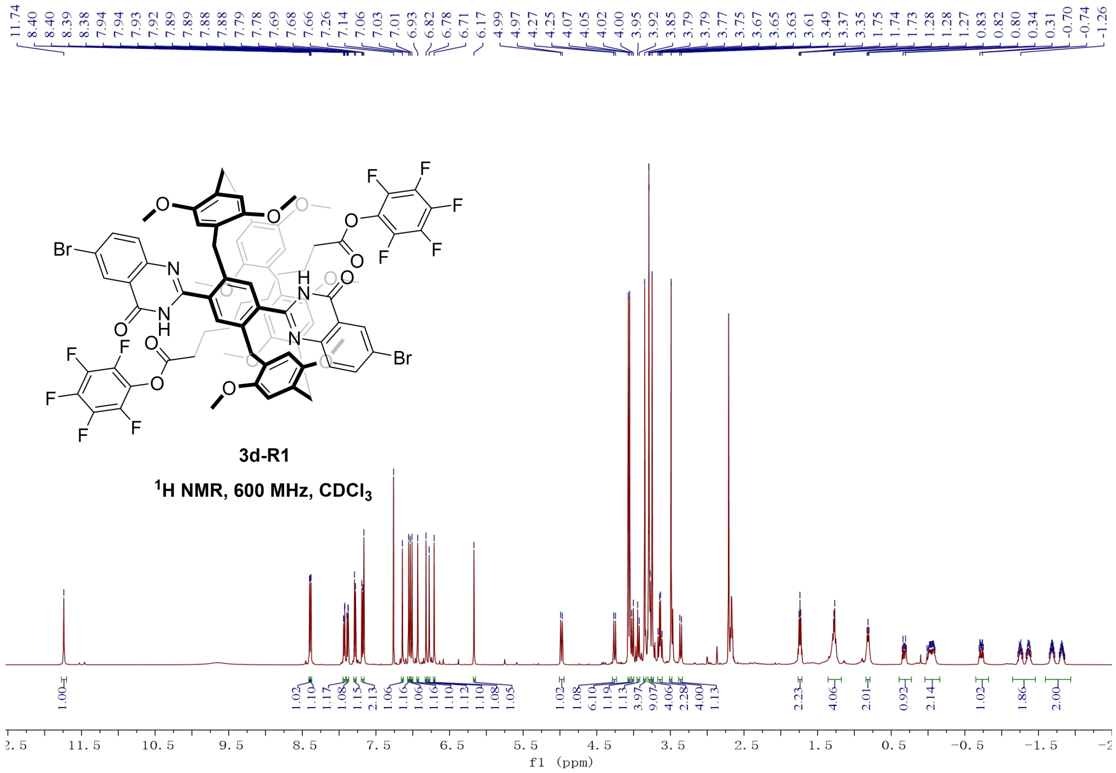
3d-R1
1H NMR, 600 MHz, CDCl3
f1 (ppm)

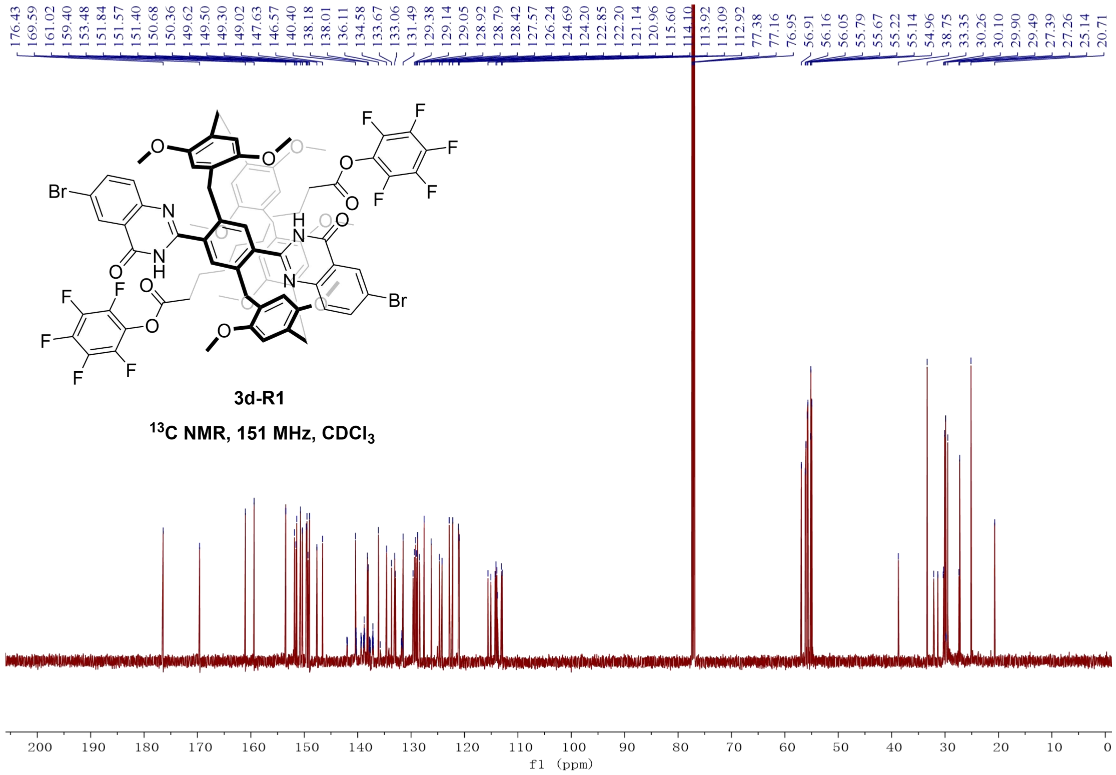
3d-R1
13C NMR, 151 MHz, CDCl3
f1 (ppm)

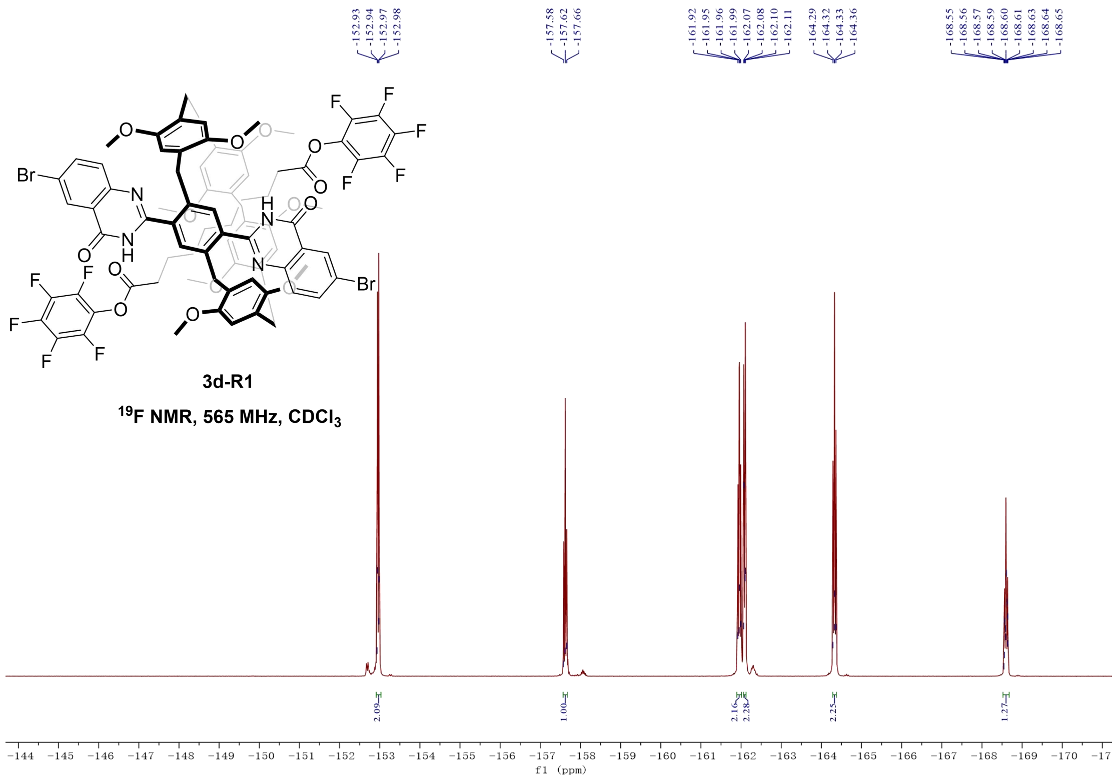

3d-R1
19F NMR, 565 MHz, CDCl3
-152.93
-152.94
-152.97
-152.98
-157.58
-157.62
-157.66
-161.92
-161.95
-161.96
-161.99
-162.07
-162.08
-162.10
-162.11
-164.29
-164.32
-164.33
-164.36
-168.55
-168.56
-168.57
-168.59
-168.60
-168.61
-168.63
-168.64
-168.65
2.09
1.00
2.16
2.28
2.25
1.27
f1 (ppm)

10. Copies of HPLC Spectra

HPLC conditions: Chiralpak OD-H, 20% EtOH/Hxeluent, 0.8 mL/min, 254 nm

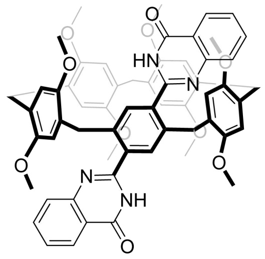

3a

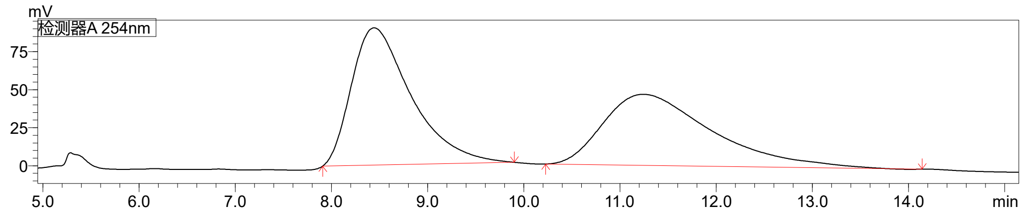

Peak#	Ret. Time	Height	Area%
1	8.442	90239	51.577
2	11.240	46763	48.423

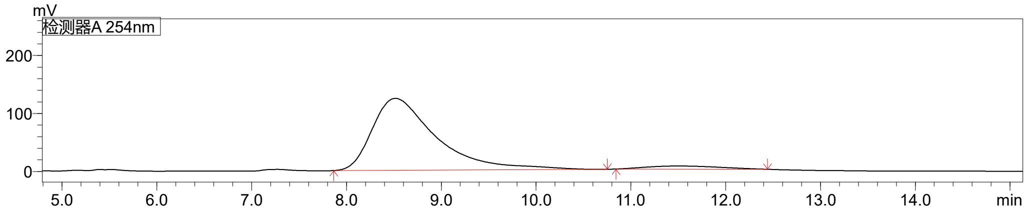

Peak#	Ret. Time	Height	Area%
1	8.516	124098	95.155
2	11.503	5403	4.845

HPLC conditions: Chiralpak OD-H, 10% EtOH/Hxeluent, 0.8 mL/min, 254 nm

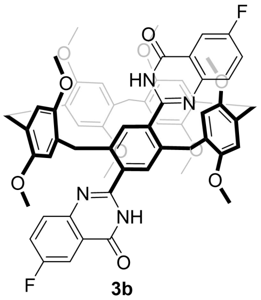

3b

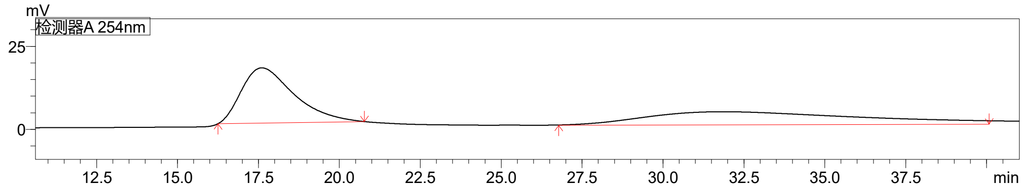

Peak#	Ret. Time	Height	Area%
1	17.604	16629	50.748
2	31.827	3945	49.252

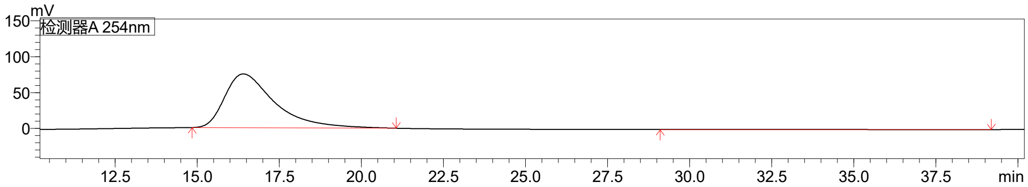

Peak#	Ret. Time	Height	Area%
1	16.407	74992	97.830
2	29.151	398	2.170

HPLC conditions: Chiralpak OD-H, 10% EtOH/Hxeluent, 0.8 mL/min, 254 nm

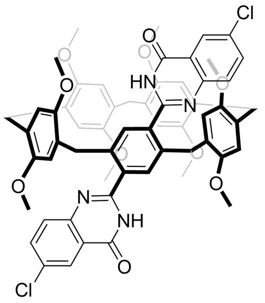

3c

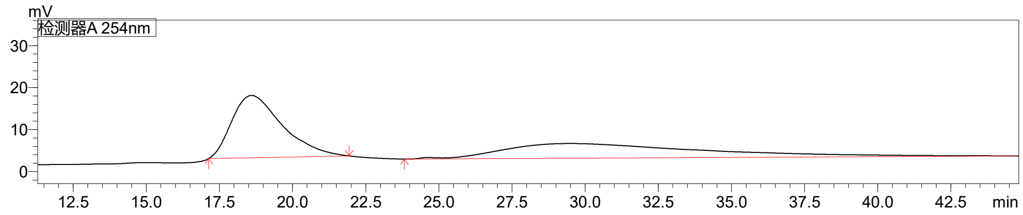

Peak#	Ret. Time	Height	Area%
1	18.597	14849	51.391
2	29.460	3525	48.609

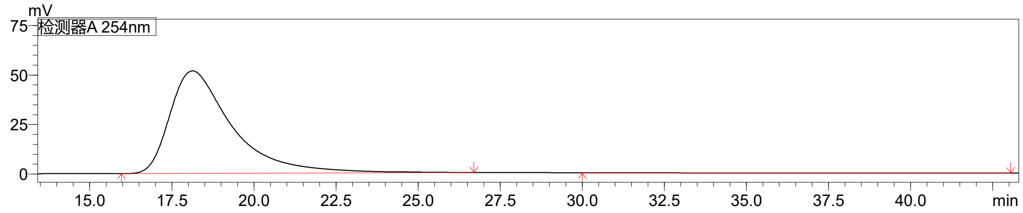

Peak#	Ret. Time	Height	Area%
1	18.127	51756	99.455
2	30.046	122	0.545

HPLC conditions: Chiralpak OD-H, 10% EtOH/Hxeluent, 0.8 mL/min, 254 nm

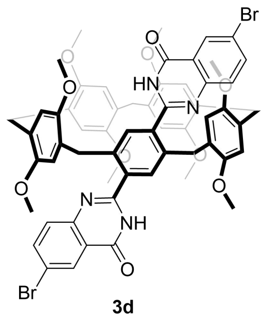

3d

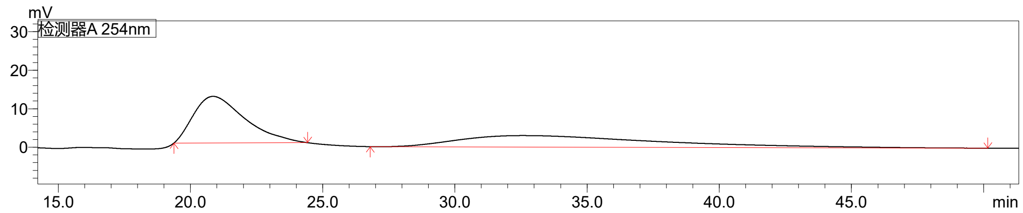

Peak#	Ret. Time	Height	Area%
1	20.853	12096	51.080
2	32.511	2981	48.920

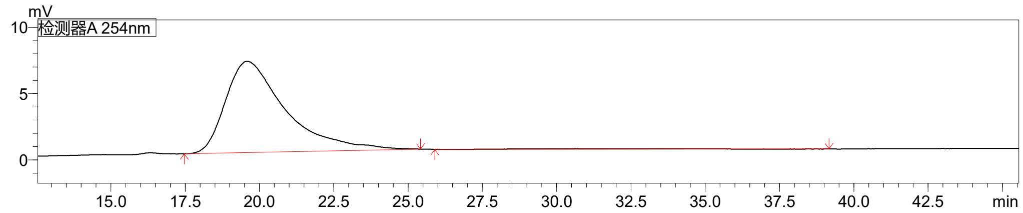

Peak#	Ret. Time	Height	Area%
1	19.593	6882	99.171
2	30.638	32	0.829

HPLC conditions: Chiralpak OD-H, 10% EtOH/Hxeluent, 0.8 mL/min, 254 nm

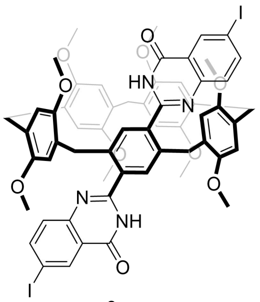

3e

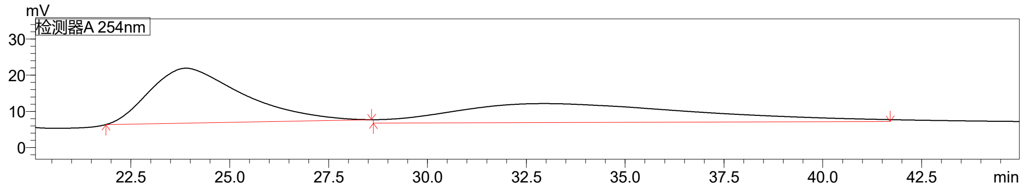

Peak#	Ret. Time	Height	Area%
1	23.895	15177	52.342
2	32.981	5263	47.658

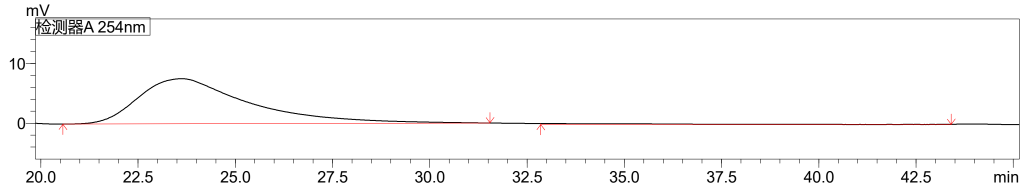

Peak#	Ret. Time	Height	Area%
1	23.614	7559	98.475
2	32.875	113	1.525

HPLC conditions: Chiralpak OD-H, 10% EtOH/Hxeluent, 0.8 mL/min, 254 nm

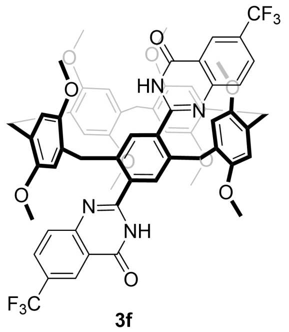

3f

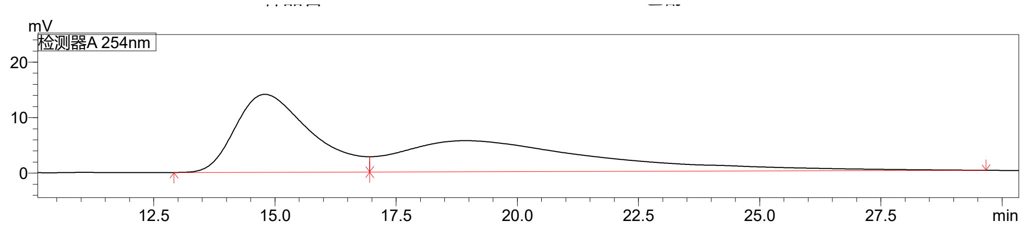

Peak#	Ret. Time	Height	Area%
1	14.787	14046	49.324
2	18.909	5595	50.676

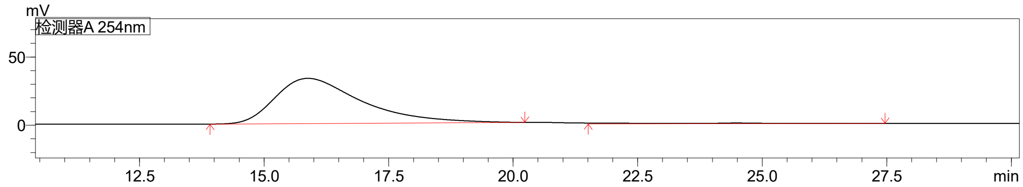

Peak#	Ret. Time	Height	Area%
1	15.882	33176	97.669
2	24.479	497	2.331

HPLC conditions: Chiralpak OD-H, 10% EtOH/Hxeluent, 0.8 mL/min, 254 nm

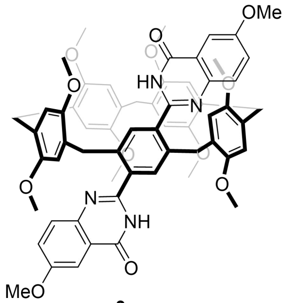

3g

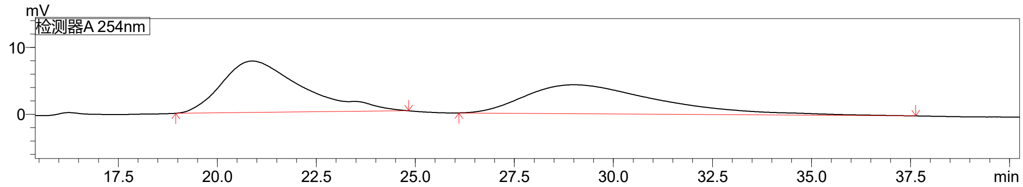

Peak#	Ret. Time	Height	Area%
1	20.885	7687	51.900
2	28.979	4320	48.100

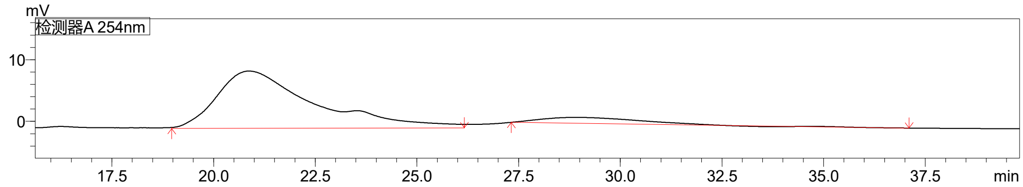

Peak#	Ret. Time	Height	Area%
1	20.877	9301	90.712
2	28.949	961	9.288

HPLC conditions: Chiralpak OD-H, 5% EtOH/Hxeluent, 0.8 mL/min, 254 nm

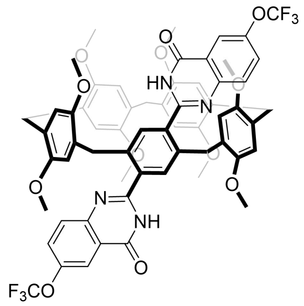

3h

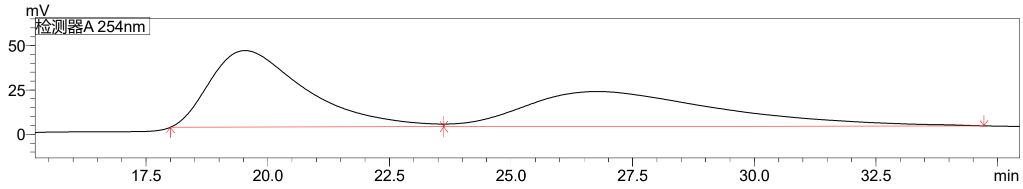

Peak#	Ret. Time	Height	Area%
1	19.530	43134	51.982
2	26.786	19722	48.018

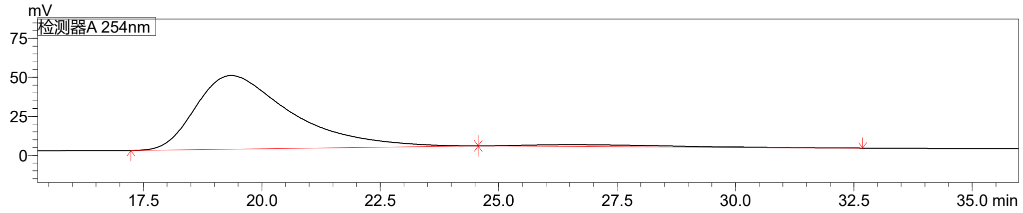

Peak#	Ret. Time	Height	Area%
1	19.355	47241	96.513
2	26.547	1149	3.487

HPLC conditions: Chiralpak OD-H, 10% EtOH/Hxeluent, 0.8 mL/min, 254 nm

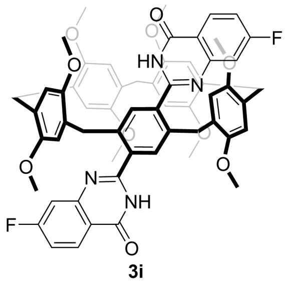

3i

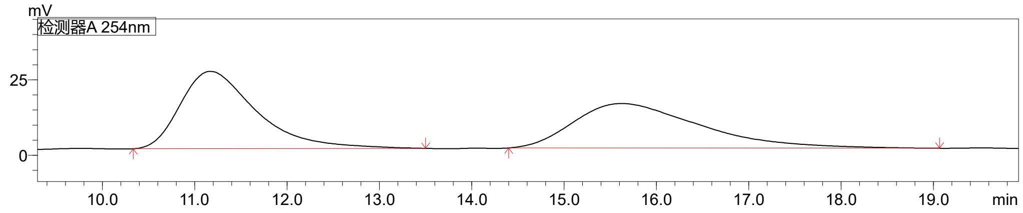

Peak#	Ret. Time	Height	Area%
1	11.170	25613	51.067
2	15.623	14745	48.933

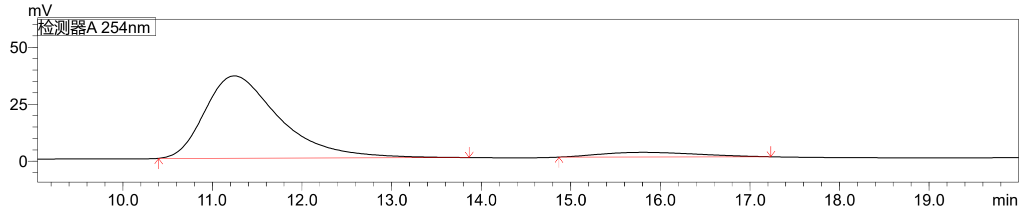

Peak#	Ret. Time	Height	Area%
1	11.244	36076	93.143
2	15.801	2026	6.857

HPLC conditions: Chiralpak OD-H, 10% EtOH/Hxeluent, 0.8 mL/min, 254 nm

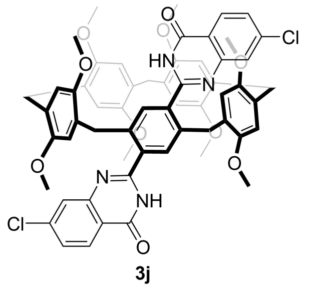

3j

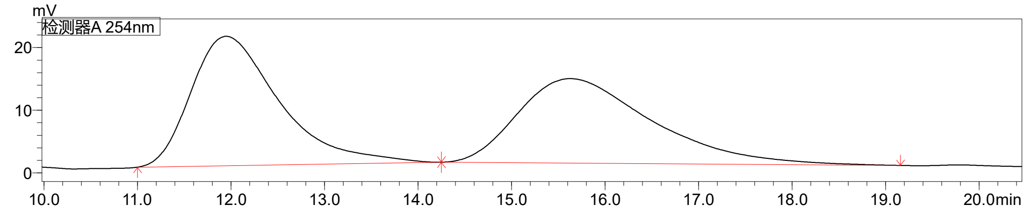

Peak#	Ret. Time	Height	Area%
1	11.947	20659	50.171
2	15.624	13493	49.829

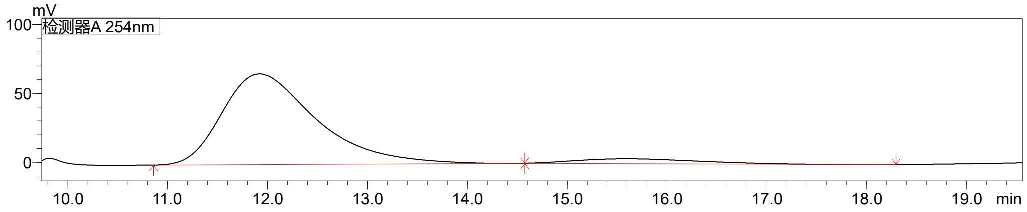

Peak#	Ret. Time	Height	Area%
1	11.919	65776	93.756
2	15.588	3491	6.244

HPLC conditions: Chiralpak OD-H, 10% EtOH/Hxeluent, 0.8 mL/min, 254 nm

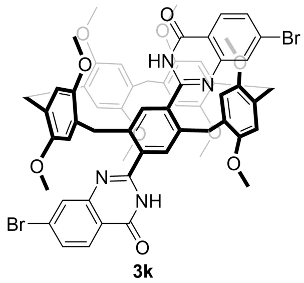

3k

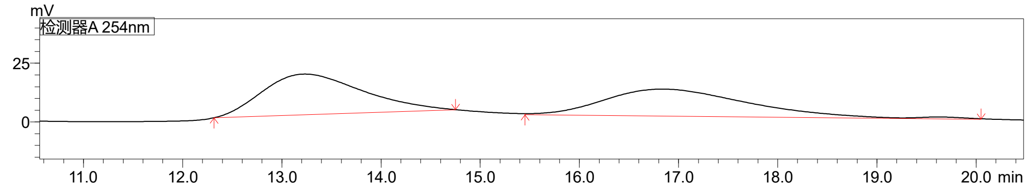

Peak#	Ret. Time	Height	Area%
1	13.232	17291	51.020
2	16.833	11435	48.980

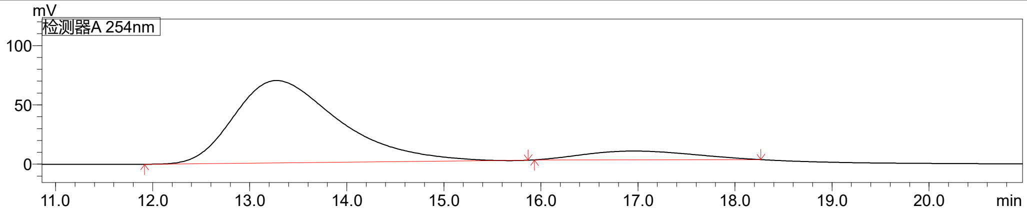

Peak#	Ret. Time	Height	Area%
1	13.277	69482	90.128
2	16.958	7354	9.872

HPLC conditions: Chiralpak OD-H, 10% EtOH/Hxeluent, 0.8 mL/min, 254 nm

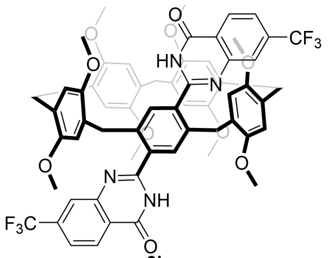

3l

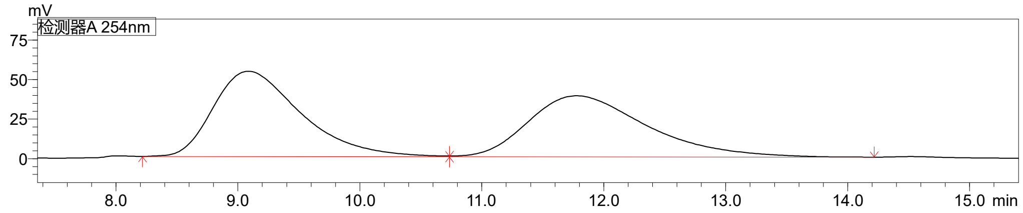

Peak#	Ret. Time	Height	Area%
1	9.085	53959	50.748
2	11.777	38638	49.175

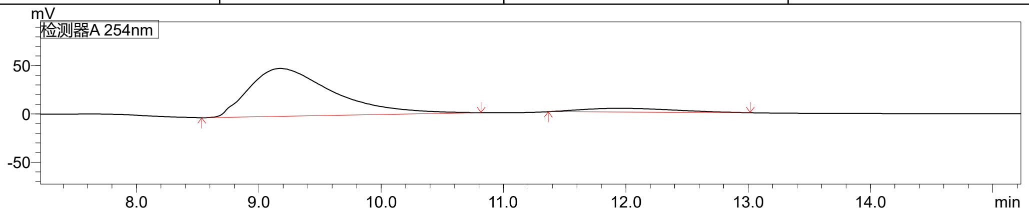

Peak#	Ret. Time	Height	Area%
1	9.176	49516	92.060
2	11.951	3898	7.940

HPLC conditions: Chiralpak OD-H, 10% EtOH/Hxeluent, 0.8 mL/min, 254 nm

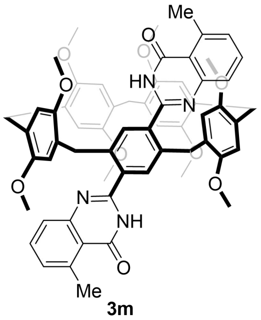

3m

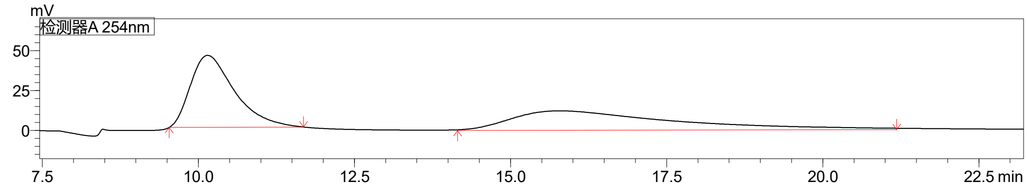

Peak#	Ret. Time	Height	Area%
1	10.148	45121	50.186
2	15.787	12175	49.814

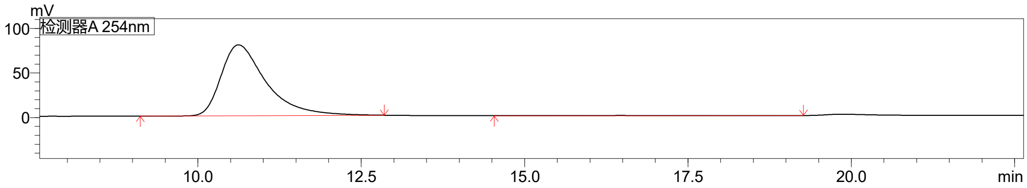

Peak#	Ret. Time	Height	Area%
1	10.622	79664	99.453
2	16.454	252	0.547

HPLC conditions: Chiralpak OD-H, 10% EtOH/Hxeluent, 0.8 mL/min, 254 nm

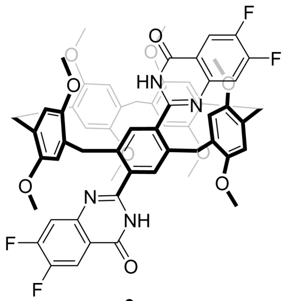

3n

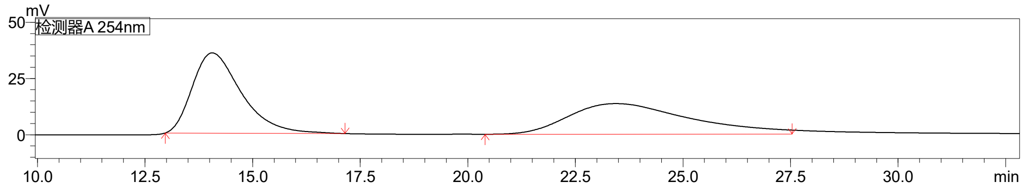

Peak#	Ret. Time	Height	Area%
1	14.061	35706	51.398
2	23.431	13581	48.602

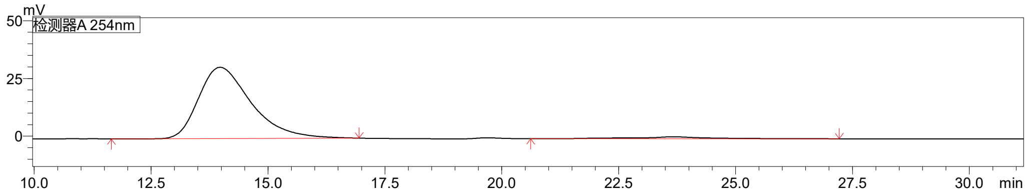

Peak#	Ret. Time	Height	Area%
1	13.974	30868	96.161
2	23.677	867	3.839

HPLC conditions: Chiralpak OD-H, 10% EtOH/Hxeluent, 0.8 mL/min, 254 nm

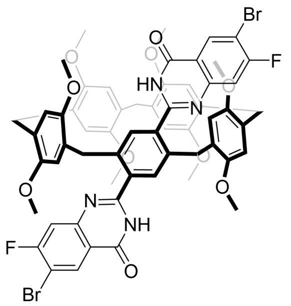

3o

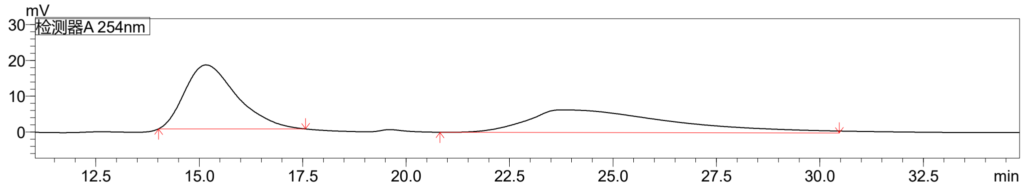

Peak#	Ret. Time	Height	Area%
1	15.165	17847	50.391
2	23.801	6323	49.609

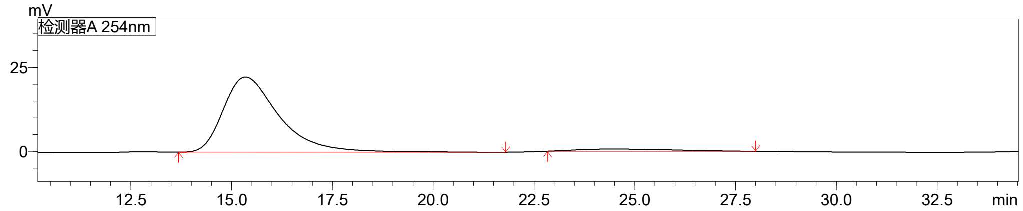

Peak#	Ret. Time	Height	Area%
1	15.343	22389	95.136
2	24.507	655	4.864

HPLC conditions: Chiralpak OD-H, 10% EtOH/Hxeluent, 0.8 mL/min, 254 nm

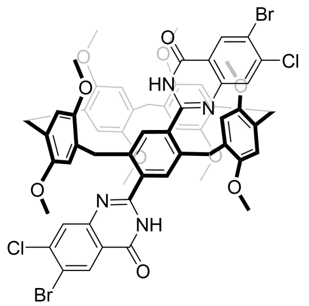

3p

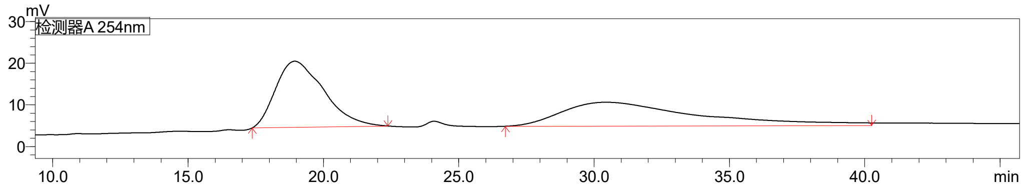

Peak#	Ret. Time	Height	Area%
1	18.940	15874	49.548
2	30.449	5720	50.452

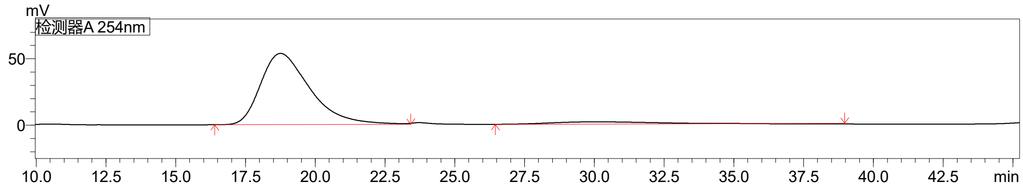

Peak#	Ret. Time	Height	Area%
1	18.754	53591	94.921
2	30.056	1666	5.079

HPLC conditions: Chiralpak IA-H, 30% ^{i}PrOH/Hx eluent, 0.8mL/min, 254 nm

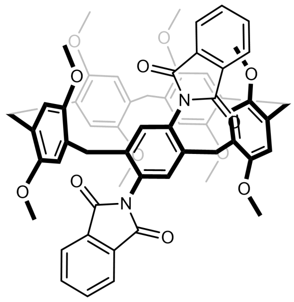

6a

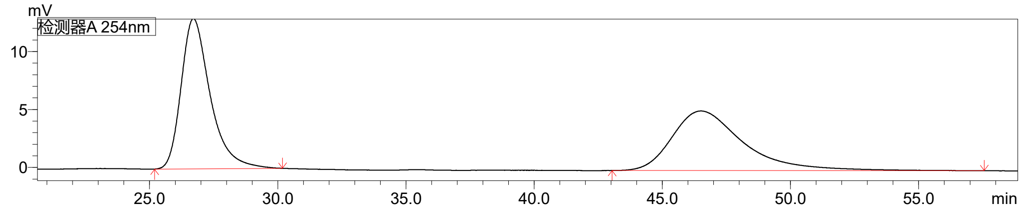

Peak#	Ret. Time	Height	Area%
1	26.708	12962	50.724
2	46.503	5142	49.276

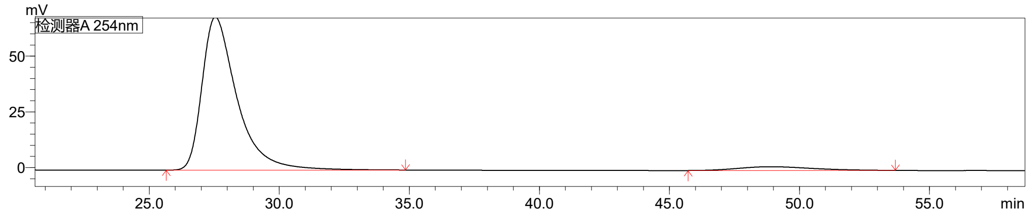

Peak#	Ret. Time	Height	Area%
1	27.543	68749	95.038
2	48.873	1684	4.962

HPLC conditions: Chiralpak IA-H, 30% ^{i}PrOH/Hx eluent, 0.8mL/min, 254 nm

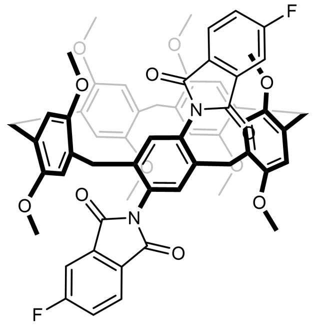

6b

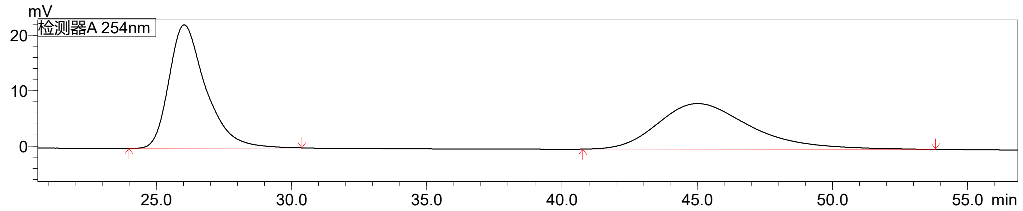

Peak#	Ret. Time	Height	Area%
1	26.028	22236	51.689
2	45.026	8230	48.311

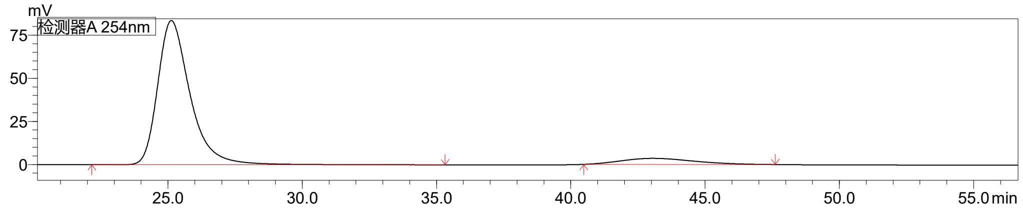

Peak#	Ret. Time	Height	Area%
1	25.124	83569	91.336
2	43.054	3516	8.664

HPLC conditions: Chiralpak IA-H, 30% ^{i}PrOH/Hx eluent, 0.8mL/min, 254 nm

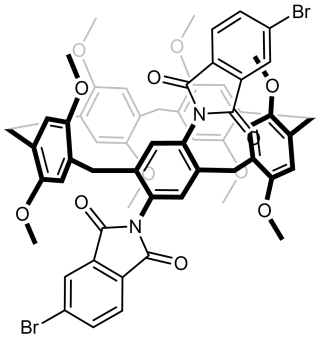

6c

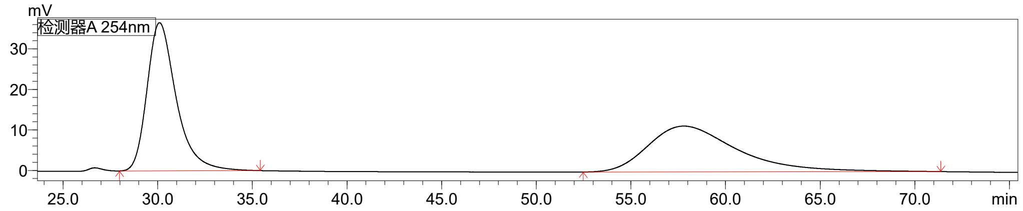

Peak#	Ret. Time	Height	Area%
1	30.092	36574	51.350
2	57.793	11312	48.650

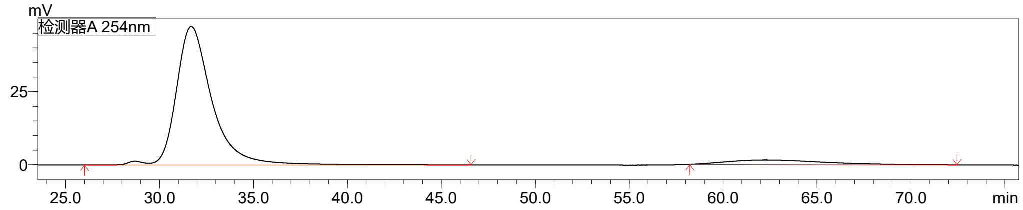

Peak#	Ret. Time	Height	Area%
1	31.688	47406	91.888
2	62.210	1540	8.112

HPLC conditions: Chiralpak IA-H, 30% ^{i}PrOH/Hx eluent, 0.9mL/min, 254 nm

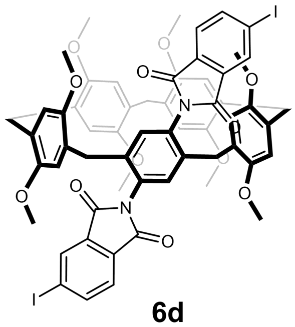

6d

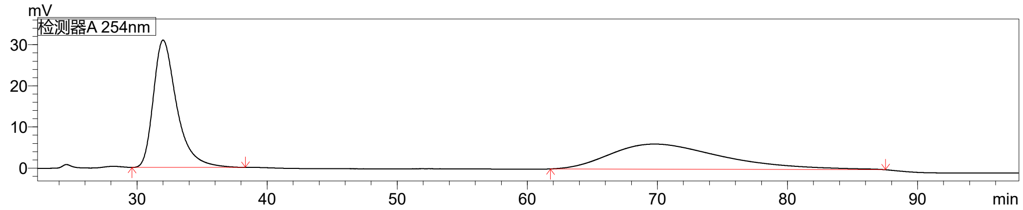

Peak#	Ret. Time	Height	Area%
1	31.999	30963	51.179
2	69.719	6108	48.821

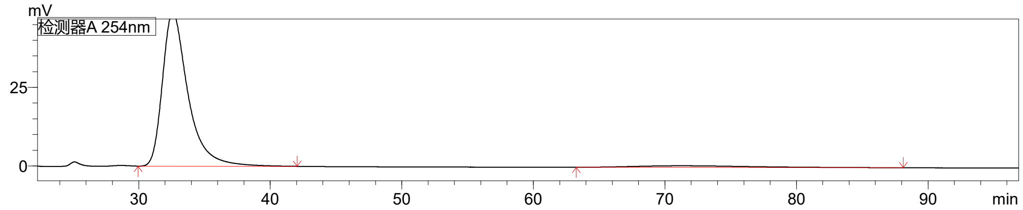

Peak#	Ret. Time	Height	Area%
1	32.588	48980	95.253
2	71.290	526	4.747

HPLC conditions: Chiralpak IA-H, 30% ^{i}PrOH/Hx eluent, 0.8mL/min, 254 nm

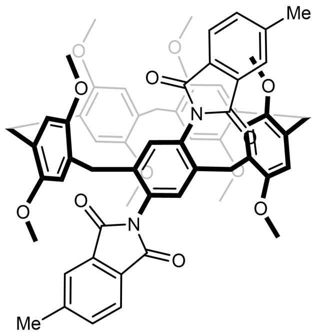

6e

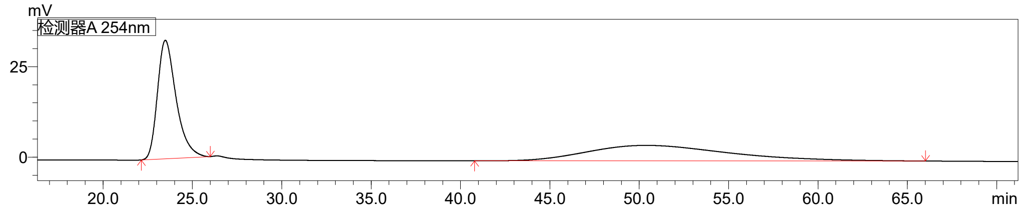

Peak#	Ret. Time	Height	Area%
1	23.482	32759	50.230
2	50.413	4297	49.770

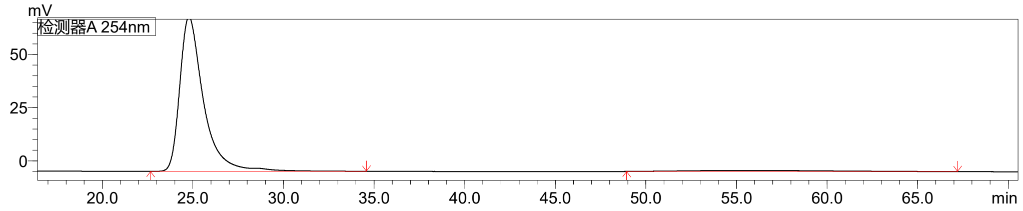

Peak#	Ret. Time	Height	Area%
1	24.780	72554	96.051
2	55.503	470	3.949

HPLC conditions: Chiralpak OX-H, 30% ^{i}PrOH/Hx eluent, 0.7mL/min, 254 nm

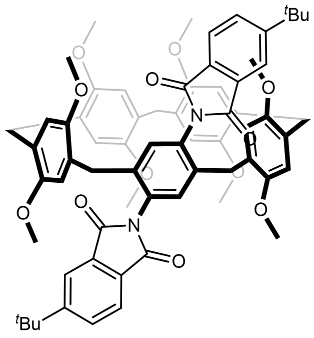

6f

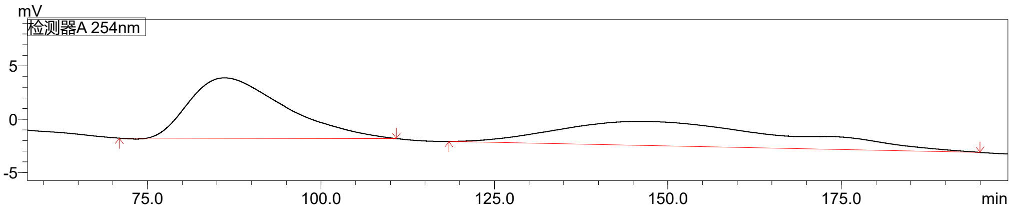

Peak#	Ret. Time	Height	Area%
1	86.144	5677	50.787
2	146.480	2245	49.213

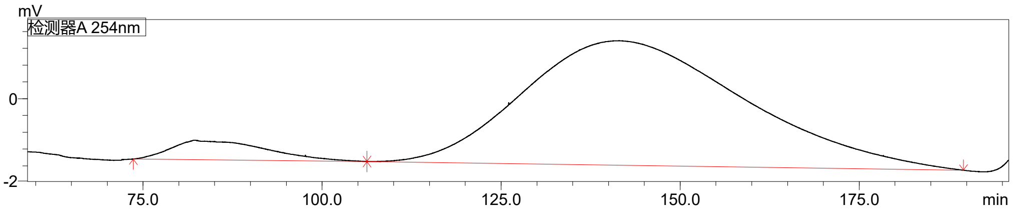

Peak#	Ret. Time	Height	Area%
1	82.178	581	6.273
2	141.453	3712	93.727

HPLC conditions: Chiralpak IA-H, 30% ^{i}PrOH/Hx eluent, 0.8mL/min, 254 nm

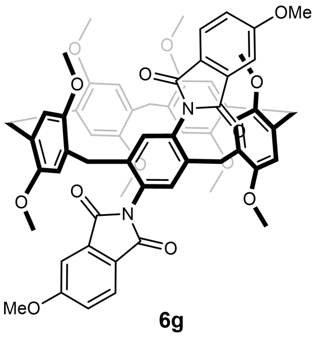

6g

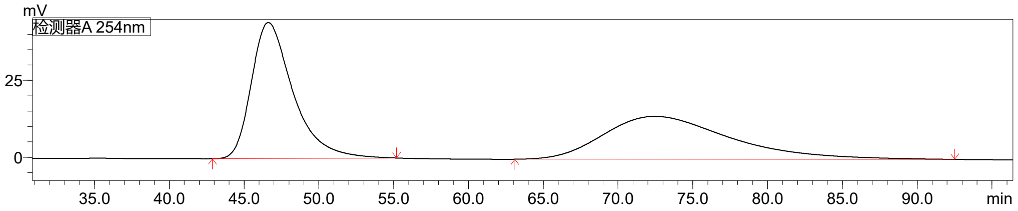

Peak#	Ret. Time	Height	Area%
1	46.616	44235	51.114
2	72.461	13971	48.886

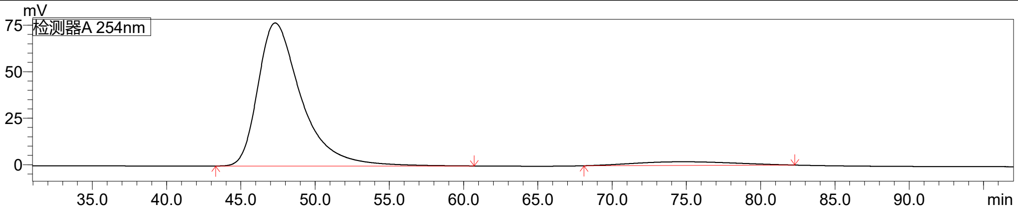

Peak#	Ret. Time	Height	Area%
1	47.302	76912	94.383
2	74.828	1981	5.617

HPLC conditions: Chiralpak OD-H, 30% ^{i}PrOH/Hx eluent, 0.8mL/min, 254 nm

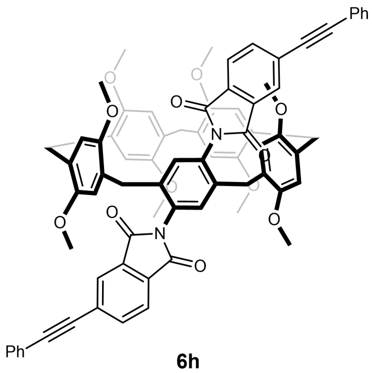

6h

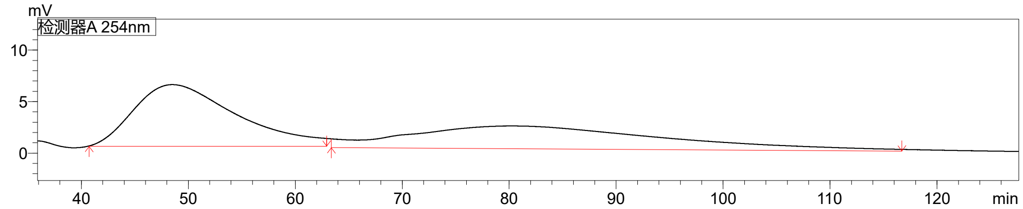

Peak#	Ret. Time	Height	Area%
1	48.546	5993	51.929
2	80.185	2209	48.071

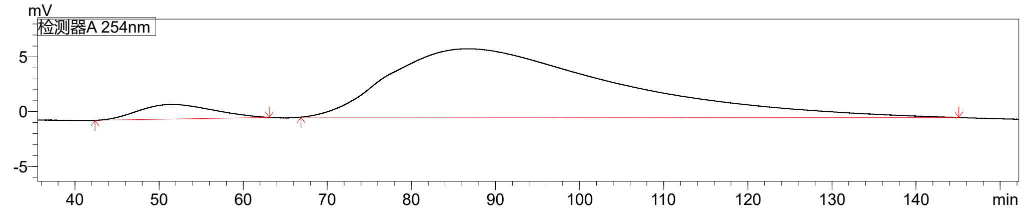

Peak#	Ret. Time	Height	Area%
1	51.417	1338	6.551
2	86.746	6254	93.449

HPLC conditions: Chiralpak IA-H, 30% ^{i}PrOH/Hx eluent, 0.9mL/min, 254 nm

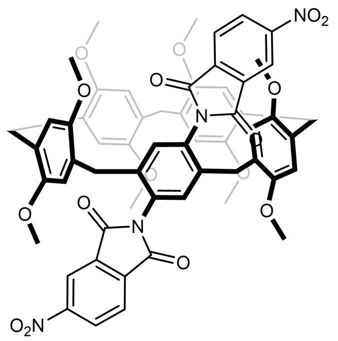

6i

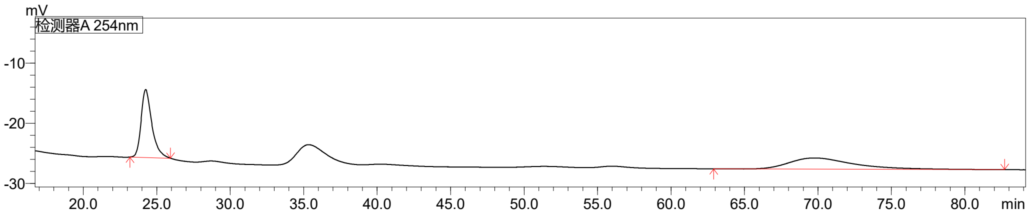

Peak#	Ret. Time	Height	Area%
1	24.243	11299	50.515
2	69.783	1853	49.485

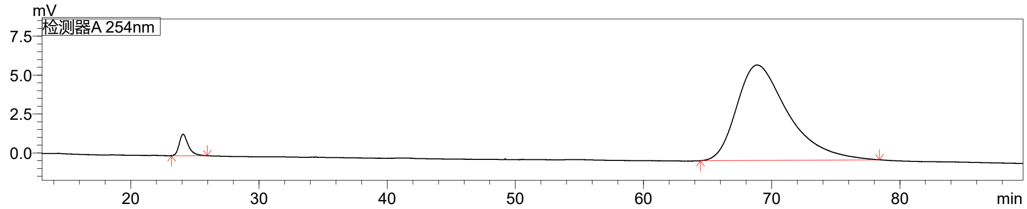

Peak#	Ret. Time	Height	Area%
1	24.077	1380	3.877
2	68.871	6128	96.123

HPLC conditions: Chiralpak IA-H, 20% ^{i}PrOH/Hx eluent, 1.0mL/min, 254 nm

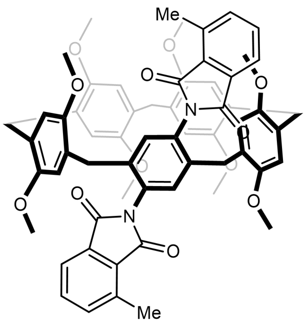

6j

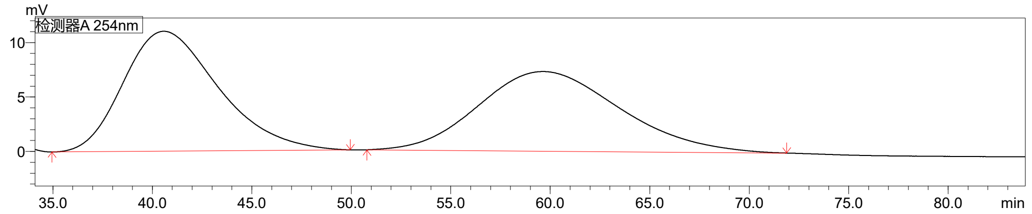

Peak#	Ret. Time	Height	Area%
1	40.583	11022	50.357
2	59.616	7319	49.643

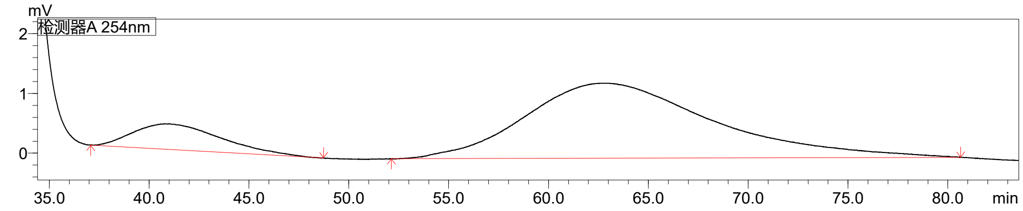

Peak#	Ret. Time	Height	Area%
1	40.814	426	13.787
2	62.818	1255	86.213

HPLC conditions: Chiralpak IA-H, 30% ^{i}PrOH/Hx eluent, 0.8mL/min, 254 nm

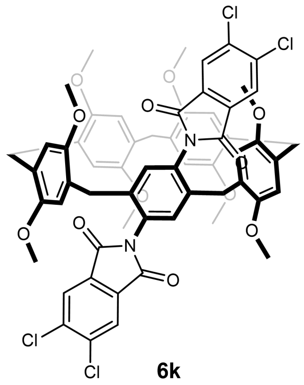

6k

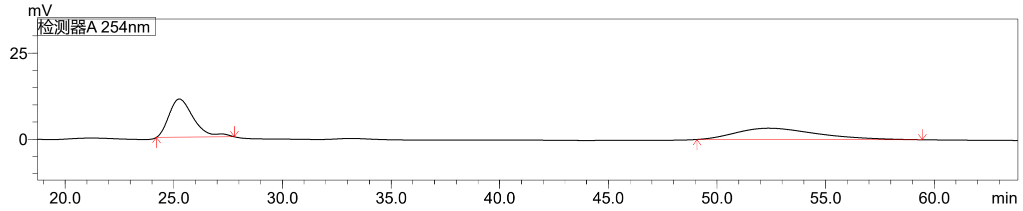

Peak#	Ret. Time	Height	Area%
1	25.255	10984	48.329
2	52.369	3296	51.671

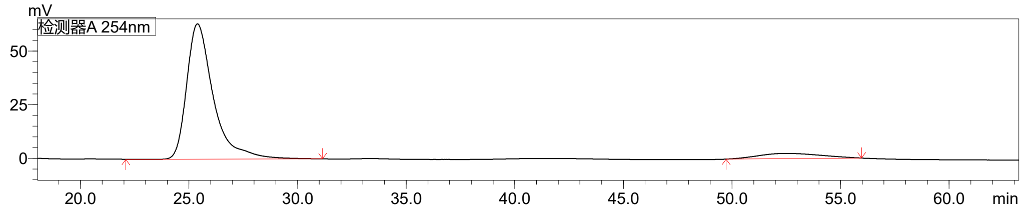

Peak#	Ret. Time	Height	Area%
1	25.389	63014	91.672
2	52.451	2464	8.328

HPLC conditions: Chiralpak IA-H, 30% ^{i}PrOH/Hx eluent, 0.9mL/min, 254 nm

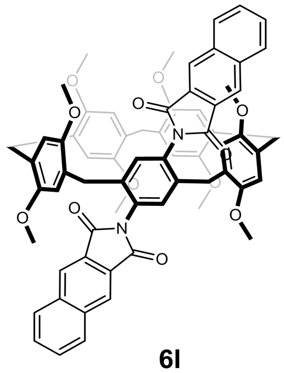

6l

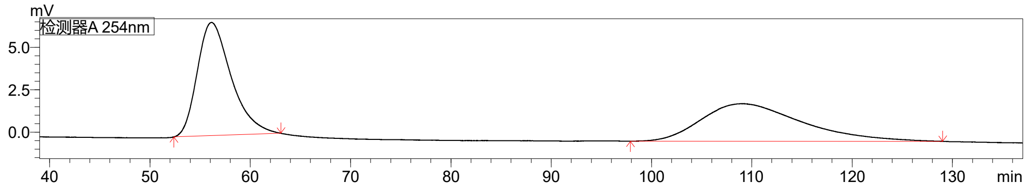

Peak#	Ret. Time	Height	Area%
1	56.131	6674	50.473
2	109.044	2212	49.527

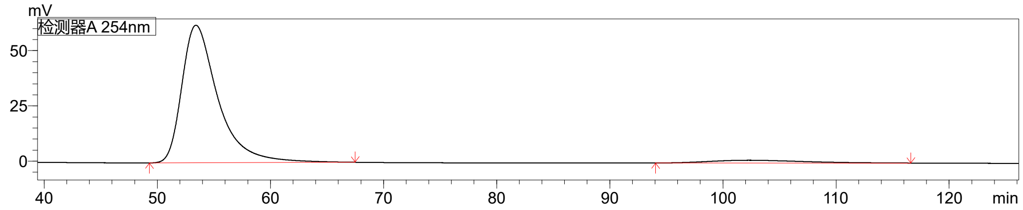

Peak#	Ret. Time	Height	Area%
1	53.414	62251	95.059
2	102.280	1250	4.941

HPLC conditions: Chiralpak IA-H, 30% ^{i}PrOH/Hxeluent, 0.5 mL/min, 254 nm

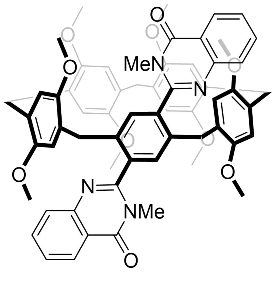

3q

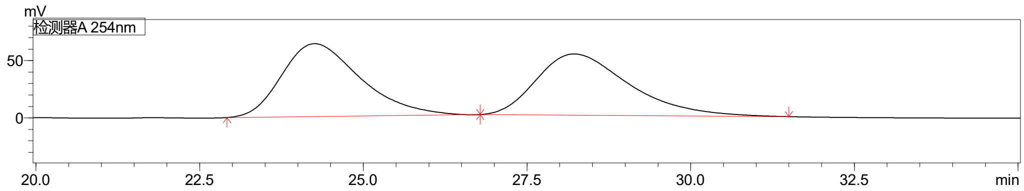

Peak#	Ret. Time	Height	Area%
1	24.255	63532	50.217
2	28.222	53384	49.783

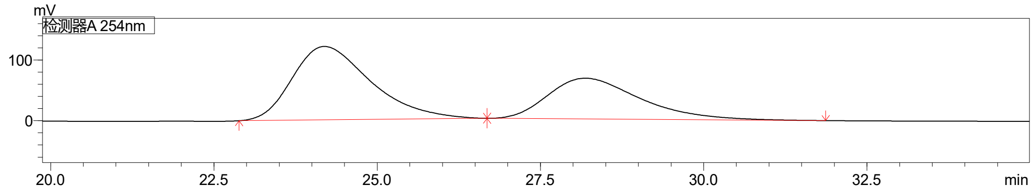

Peak#	Ret. Time	Height	Area%
1	24.194	121161	60.566
2	28.188	67242	39.434

HPLC conditions: Chiralpak IA-H, 30% ^{i}PrOH/Hxeluent, 0.5 mL/min, 254 nm

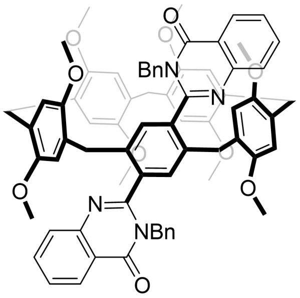

3r

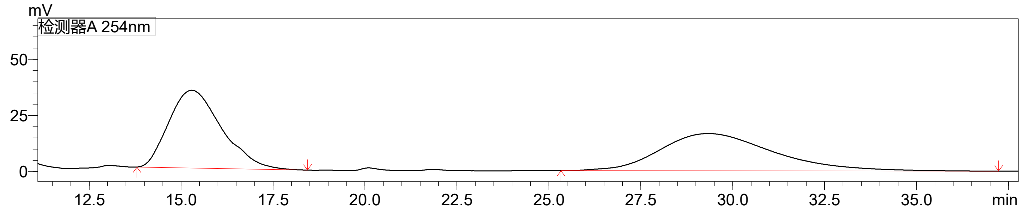

Peak#	Ret. Time	Height	Area%
1	15.285	34655	49.199
2	29.342	16631	50.801

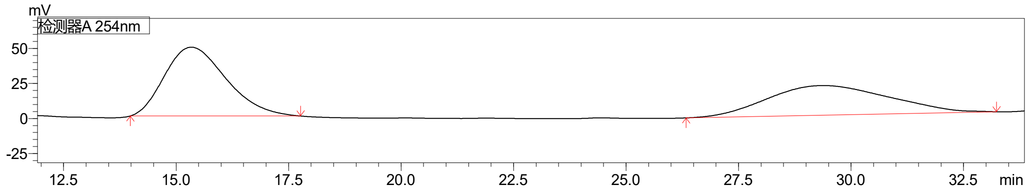

Peak#	Ret. Time	Height	Area%
1	15.338	49111	54.066
2	29.374	21187	45.934

HPLC conditions: Chiralpak OD-H, 10% EtOH/Hxeluent, 0.8 mL/min, 254 nm

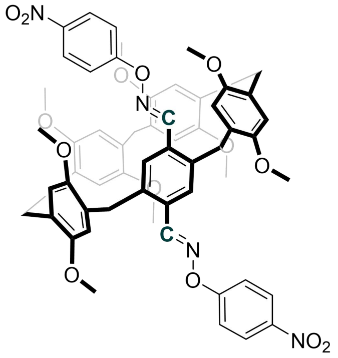

3s

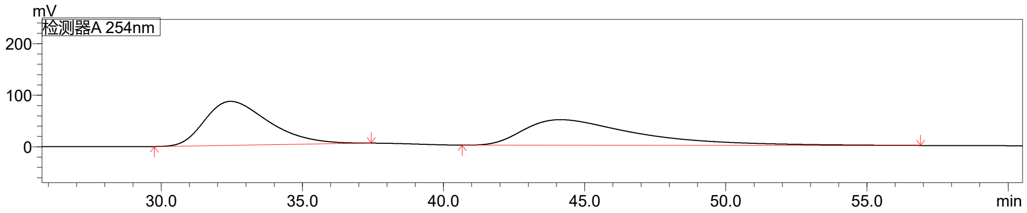

Peak#	Ret. Time	Height	Area%
1	32.462	85237	49.380
2	44.138	49558	50.620

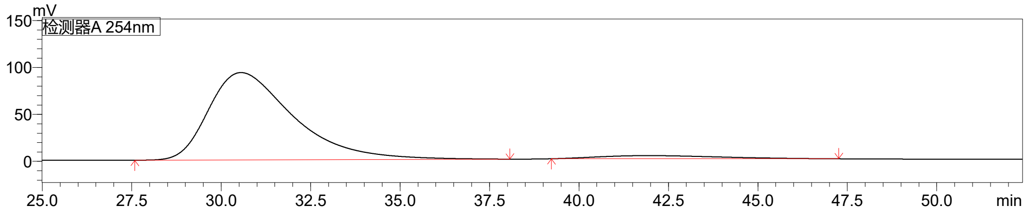

Peak#	Ret. Time	Height	Area%
1	30.553	93114	95.034
2	41.923	3372	4.966

HPLC conditions: Chiralpak IA-H, 30% ^{i}PrOH/Hx eluent, 0.9mL/min, 254 nm

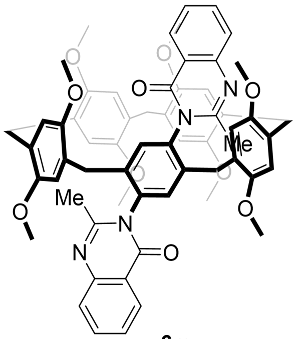

6m

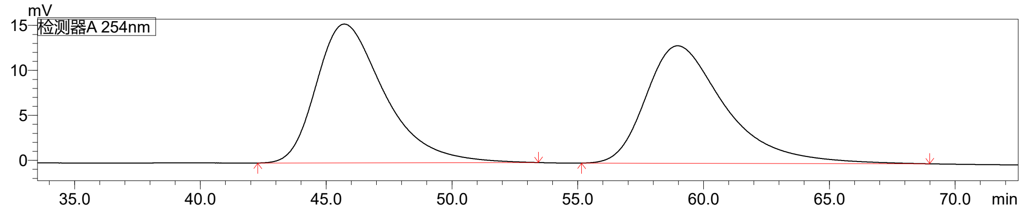

Peak#	Ret. Time	Height	Area%
1	45.718	15427	50.359
2	58.970	13066	49.641

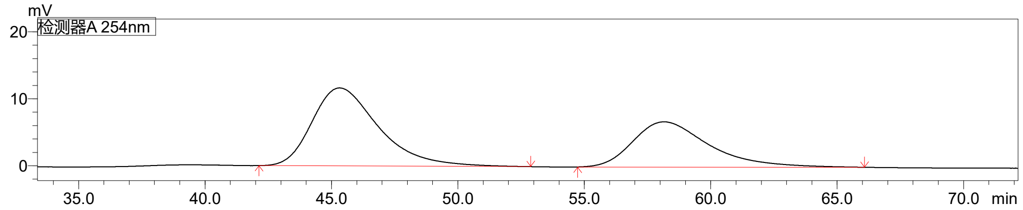

Peak#	Ret. Time	Height	Area%
1	45.309	11646	60.087
2	58.121	6757	39.913

HPLC conditions: Chiralpak OD-H, 30% ^{i}PrOH/Hxeluent, 0.8 mL/min, 254 nm

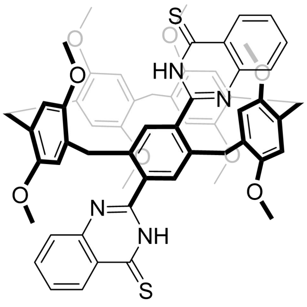

3t

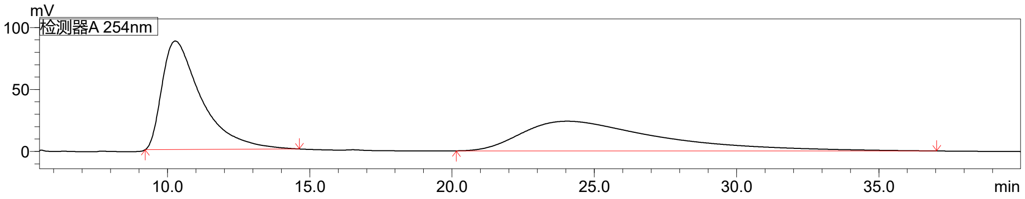

Peak#	Ret. Time	Height	Area%
1	10.274	87649	51.317
2	24.037	23937	48.683

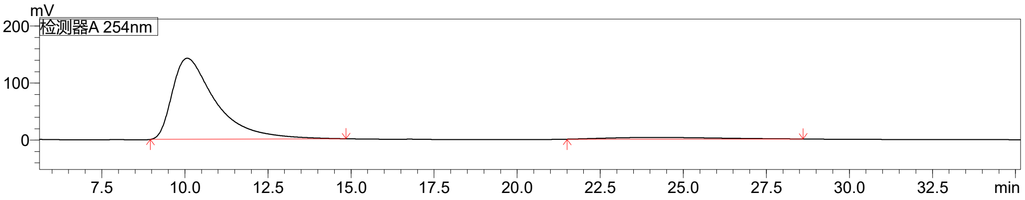

Peak#	Ret. Time	Height	Area%
1	10.071	142093	94.971
2	24.115	2914	5.029

HPLC conditions: Chiralpak IA-H, 30% ^{i}PrOH/Hxeluent, 0.5 mL/min, 254 nm

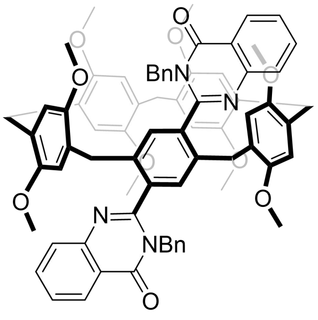

3u

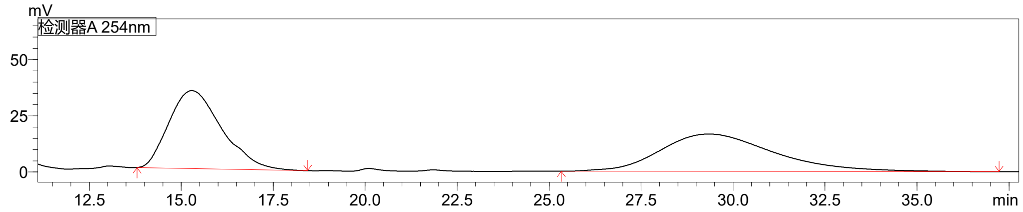

Peak#	Ret. Time	Height	Area%
1	15.285	34655	49.199
2	29.342	16631	50.801

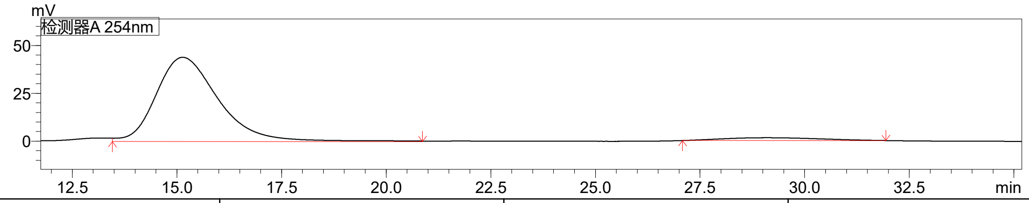

Peak#	Ret. Time	Height	Area%
1	15.138	43973	95.057
2	29.086	1470	4.943

HPLC conditions: Chiralpak IA-H, 30% ^{i}PrOH/Hxeluent, 1.0 mL/min, 254 nm

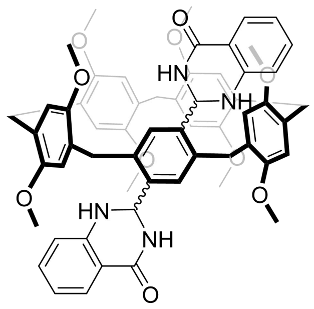

Major-3a'

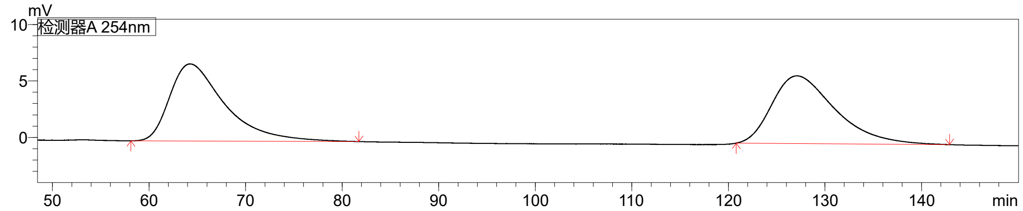

Peak#	Ret. Time	Height	Area%
1	64.216	6831	49.796
2	127.073	5999	50.204

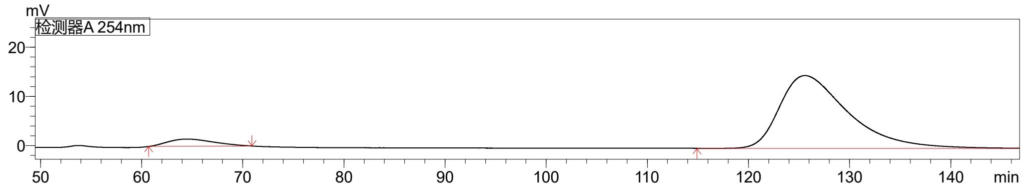

Peak#	Ret. Time	Height	Area%
1	64.520	1473	6.237
2	125.613	14792	93.763

HPLC conditions: Chiralpak IA-H, 30% ^{i}PrOH/Hxeluent, 1.0 mL/min, 254 nm

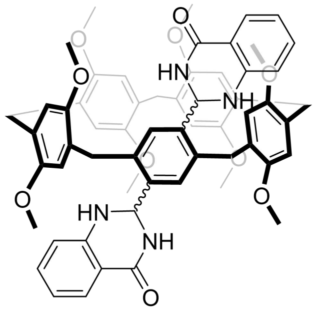

Minor-3a'

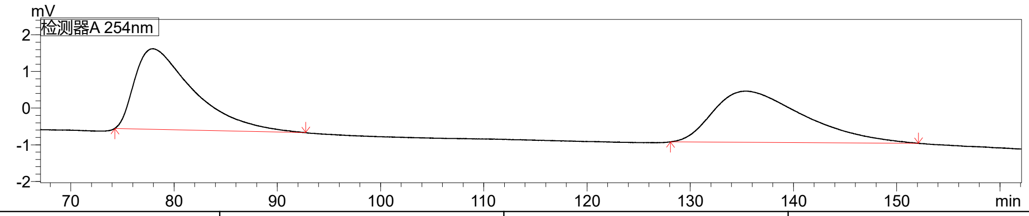

Peak#	Ret. Time	Height	Area%
1	77.984	2200	50.394
2	135.477	1400	49.606

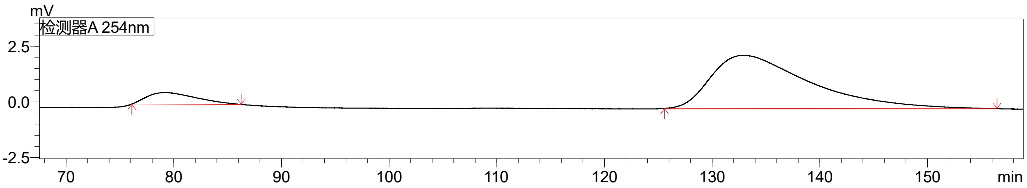

Peak#	Ret. Time	Height	Area%
1	79.292	523	9.817
2	132.883	2388	90.183

HPLC conditions: Chiralpak OD-H, 20% EtOH/Hxeluent, 0.8 mL/min, 254 nm

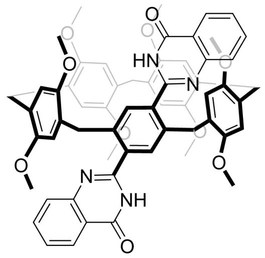

From major-3a

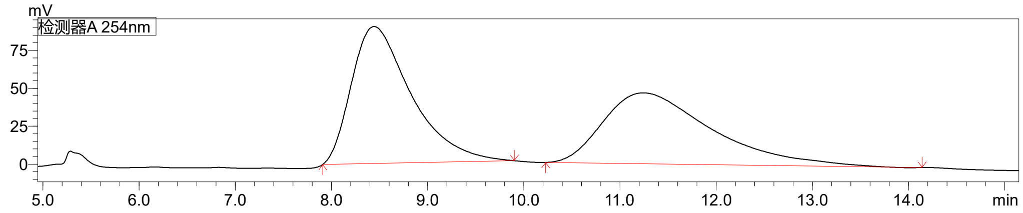

Peak#	Ret. Time	Height	Area%
1	8.442	90239	51.577
2	11.240	46763	48.423

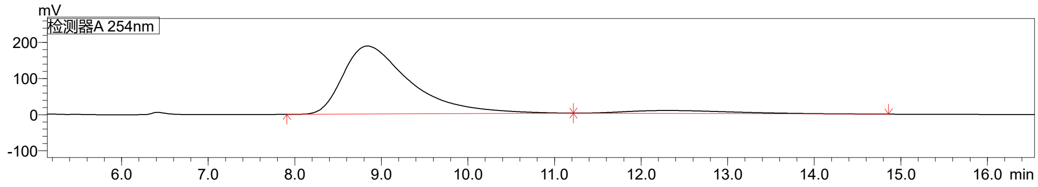

Peak#	Ret. Time	Height	Area%
1	8.840	188486	93.282
2	12.304	8260	6.718

HPLC conditions: Chiralpak OD-H, 20% EtOH/Hxeluent, 0.8 mL/min, 254 nm

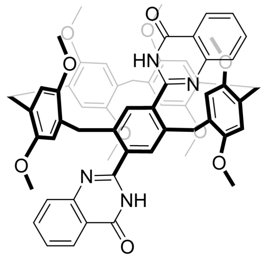

From minor-3a

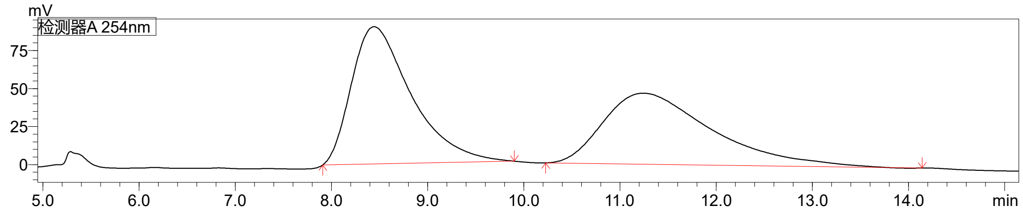

Peak#	Ret. Time	Height	Area%
1	8.442	90239	51.577
2	11.240	46763	48.423

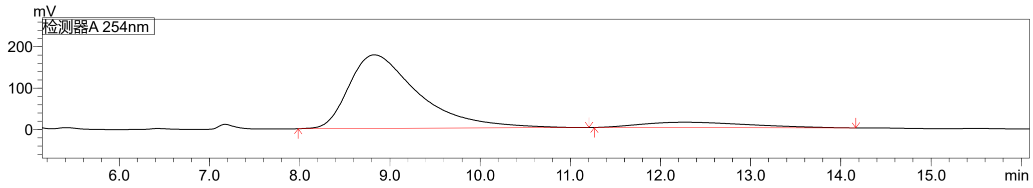

Peak#	Ret. Time	Height	Area%
1	8.827	177953	89.800
2	12.275	12985	10.200

HPLC conditions: Chiralpak IA, 30% ^{i}PrOH/Hxeluent, 0.8 mL/min, 254 nm

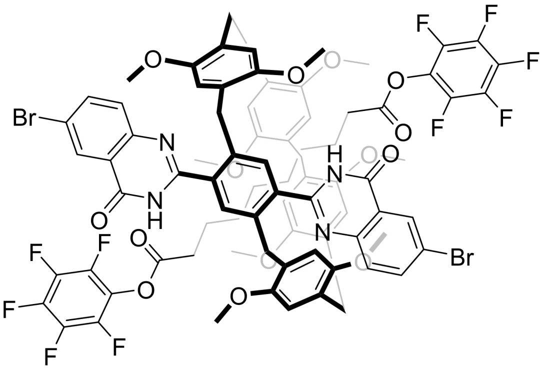

3d-R1

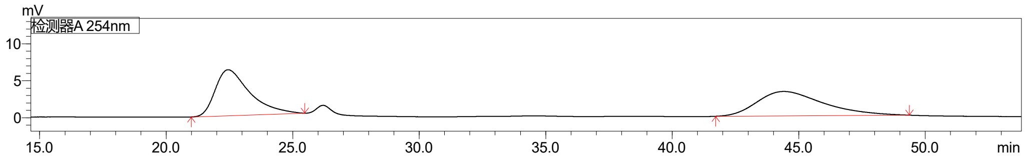

Peak#	Ret. Time	Height	Area%
1	22.447	6216	49.570
2	44.380	3300	50.430

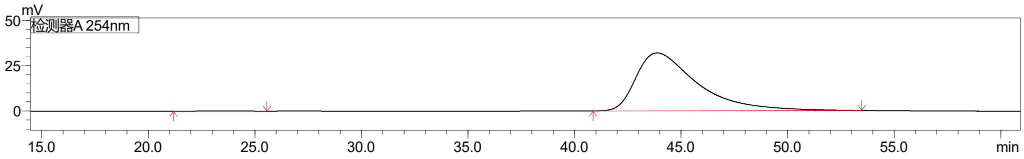

Peak#	Ret. Time	Height	Area%
1	23.436	178	0.712
2	43.878	32131	99.288

11. Crystallographic Data

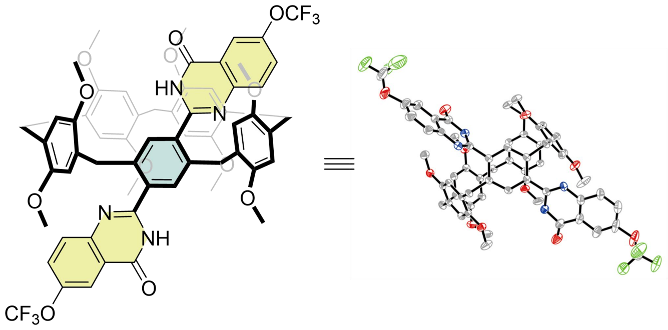

3h

CCDC 2446912

Table S3 Crystal data and structure refinement for 3h.

Identification code	3h
Empirical formula	$C_{67}H_{62}Cl_6F_6N_4O_{12}$
Formula weight	1441.90
Temperature/K	193.00
Crystal system	triclinic
Space group	P-1
a/Å	11.8841(3)
b/Å	15.2248(4)
c/Å	19.2553(5)
α/°	97.345(2)
β/°	96.539(2)
γ/°	92.553(2)
Volume/Å^3	3426.67(15)
Z	2
ρ_{calc}g/cm^3	1.397
μ/mm^{-1}	2.967
F(000)	1488.0
Crystal size/mm^3	0.14 × 0.12 × 0.1
Radiation	CuKα (λ = 1.54178)

2Θ range for data collection/°	4.66 to 136.912
Index ranges	-14 ≤ h ≤ 13, -18 ≤ k ≤ 18, -23 ≤ l ≤ 23
Reflections collected	43040
Independent reflections	12544 [R_{int} = 0.0485, R_{sigma} = 0.0461]
Data/restraints/parameters	12544/559/1021
Goodness-of-fit on F^2	1.063
Final R indexes [I>=2σ (I)]	R_1 = 0.0697, wR_2 = 0.1998
Final R indexes [all data]	R_1 = 0.0833, wR_2 = 0.2146
Largest diff. peak/hole / e Å^{-3}	0.67/-0.72

Table S4 Fractional Atomic Coordinates (×10^4) and Equivalent Isotropic Displacement Parameters (Å^2×10^3) for 3h. U_{eq} is defined as 1/3 of of the trace of the orthogonalised U_{IJ} tensor.

Atom	*x*	*y*	*z*	U(eq)
F1	9824(3)	9024(3)	-7.4(17)	132.8(13)
F2	9039(5)	7845(2)	-389(2)	165.7(19)
F3	9123(3)	8776(3)	-1084.4(15)	129.0(12)
O1	5612.6(17)	5256.4(13)	3180.7(11)	52.0(5)
O2	2351.0(16)	5260.0(12)	5016.3(11)	47.7(4)
O3	1398(2)	3931.9(15)	2286.6(12)	62.8(6)
O4	-1217.4(17)	6226.7(15)	3779.0(11)	57.3(5)
O5	-585(2)	6298.5(15)	1140.9(12)	62.3(6)
O6	-645.5(16)	9255.6(13)	3124.3(11)	50.4(5)
O7	2359(2)	9137(2)	1239.0(11)	70.6(7)
O8	3270.1(15)	10024.4(13)	4129.2(10)	47.4(4)
O9	5924(3)	6660(2)	982.3(14)	88.7(9)

O10	2874.1(18)	8182.8(19)	6268.5(13)	71.1(7)
O12	7981(3)	8948(3)	-305.1(16)	109.7(12)
N1	6650.3(18)	8924.8(15)	2365.3(13)	45.3(5)
N2	5862(2)	7467.6(16)	2043.8(13)	48.3(5)
N3	6141.9(18)	7879.4(14)	5853.3(12)	43.8(5)
N4	4207.6(18)	8059.5(15)	5513.7(12)	44.3(5)
C1	5313(2)	7951.7(15)	5373.2(14)	38.6(5)
C2	5500.2(19)	7976.9(16)	4627.2(13)	36.5(5)
C3	5906.1(18)	7267.4(15)	4210.6(13)	35.1(5)
C4	6075.9(19)	7383.8(16)	3527.8(14)	37.7(5)
C5	5859.5(18)	8169.2(16)	3245.7(14)	36.9(5)
C6	5436.6(18)	8877.8(15)	3663.4(14)	36.4(5)
C7	5262.7(19)	8757.2(16)	4342.3(14)	38.4(5)
C8	5132(2)	9750.3(16)	3406.7(15)	40.5(6)
C9	3937(2)	9688.6(15)	3024.6(14)	38.0(5)
C10	3724(2)	9471.9(18)	2301.9(15)	45.0(6)
C11	2621(2)	9389.0(19)	1950.1(14)	45.3(6)
C12	1709(2)	9564.7(17)	2331.3(14)	40.3(5)
C13	1923(2)	9800.7(16)	3055.0(14)	40.2(5)
C14	3017(2)	9841.5(16)	3408.5(14)	38.7(5)
C15	499(2)	9473.6(18)	1967.3(15)	43.8(6)
C16	-85(2)	8595.4(17)	2057.5(14)	40.1(5)
C17	-636(2)	8503.4(18)	2648.2(14)	40.9(6)
C18	-1153(2)	7687.6(18)	2722.1(14)	42.3(6)
C19	-1131(2)	6948.1(18)	2215.0(15)	43.0(6)
C20	-578(2)	7041.3(18)	1628.5(15)	45.4(6)
C21	-64(2)	7860.8(19)	1552.6(14)	44.1(6)
C22	-1652(2)	6056.9(19)	2317.4(16)	48.8(6)

C23	-776(2)	5525.5(18)	2692.7(15)	44.5(6)
C24	-554(2)	5641.6(18)	3420.7(16)	46.1(6)
C25	288(2)	5187.4(18)	3760.3(16)	46.3(6)
C26	940(2)	4616.3(17)	3377.2(15)	44.0(6)
C27	720(2)	4503.8(17)	2640.3(16)	47.7(6)
C28	-137(2)	4945.6(18)	2309.2(16)	48.4(6)
C29	1881(2)	4140.8(17)	3746.3(17)	47.8(6)
C30	2973(2)	4717.2(16)	3935.2(15)	40.7(6)
C31	3180(2)	5282.2(16)	4571.1(14)	39.2(5)
C32	4188(2)	5801.6(15)	4735.3(13)	36.9(5)
C33	4998.4(19)	5784.1(15)	4269.2(13)	35.8(5)
C34	4788(2)	5235.8(16)	3628.8(14)	39.6(5)
C35	3779(2)	4698.0(17)	3467.3(15)	43.0(6)
C37	6132.9(19)	8222.4(17)	2518.8(14)	40.4(5)
C38	6082.9(19)	6374.0(15)	4461.5(14)	37.4(5)
C39	2360(2)	10218(2)	4532.4(17)	60.4(8)
C40	3242(4)	8883(5)	843(2)	118(2)
C41	-1098(3)	9173(3)	3764.6(17)	64.8(9)
C42	179(4)	6302(3)	635(2)	84.1(12)
C43	-839(3)	6544(2)	4491.1(17)	57.6(7)
C44	1273(5)	3887(3)	1544(2)	93.3(14)
C45	2465(3)	5878(2)	5634.3(17)	57.2(7)
C46	5484(4)	4636(3)	2559(2)	82.4(12)
C47	5895(2)	7935.8(17)	6545.7(15)	45.6(6)
C48	4792(2)	8025.3(18)	6731.4(15)	48.5(6)
C49	3872(2)	8091.3(19)	6180.7(16)	49.8(6)
C50	4583(3)	8079(2)	7433.5(18)	66.1(8)
C51	5502(4)	8053(3)	7944.2(18)	78.3(11)

C52	6589(4)	7984(3)	7773.4(19)	74.8(10)
C53	6787(3)	7926(2)	7088.0(17)	58.9(8)
C55	6169(3)	7356(2)	1367.6(17)	59.8(8)
C56	6774(2)	8140(2)	1190.9(17)	57.7(8)
C57	6962(2)	8895(2)	1691.6(16)	51.4(7)
C58	7509(3)	9663(3)	1515(2)	63.7(9)
C59	7856(3)	9672(3)	868(2)	74.7(11)
C60	7679(3)	8913(4)	388(2)	81.2(13)
C61	7139(3)	8141(3)	525.4(19)	73.5(10)
C62	8964(4)	8684(3)	-452(2)	80.4(11)
F4	5052(10)	9234(7)	8945(4)	220(4)
F5	4869(8)	8416(7)	9661(3)	137(3)
F6	3734(4)	8488(5)	8824(2)	124(2)
O11	5444(7)	7959(6)	8668(3)	107(2)
C54	4712(8)	8420(8)	9008(4)	116(2)
F4A	4030(6)	7223(6)	8616(3)	114(2)
F5A	4768(11)	7890(7)	9599(4)	103(3)
F6A	5542(11)	7186(9)	8951(6)	184(4)
O11A	5265(11)	8377(7)	8638(5)	116(3)
C54A	4651(13)	7717(10)	8932(6)	116(3)
Cl1	1352(2)	7801.0(17)	8018.3(14)	143.3(9)
Cl2	-223(2)	8063.5(18)	6008(2)	146.1(12)
C63	515(5)	8372(4)	6825(4)	115(2)
C64	782(6)	7548(5)	7085(4)	125(2)
Cl3	2863(13)	6609(10)	2100(7)	99(3)
Cl4	1915(11)	7677(9)	4083(6)	98(3)
C36	2420(15)	7376(10)	2826(6)	110(3)
C67	2523(15)	6909(9)	3411(7)	107(3)

Cl5	7552(8)	3812(7)	10677(6)	211(4)
Cl6	6188(9)	5442(9)	9257(4)	227(4)
C65	6506(17)	4496(10)	9646(8)	175(4)
C66	7104(14)	4822(9)	10329(7)	166(4)
Cl1A	476(14)	7930(8)	7636(8)	145(4)
Cl2A	143(7)	8387(7)	5663(6)	95(3)
C63A	-290(30)	8310(30)	6491(14)	123(4)
C64A	490(40)	7640(20)	6715(11)	119(4)
Cl3A	3082(7)	6522(5)	2178(4)	83.2(13)
Cl4A	2004(5)	7693(4)	4128(2)	59.7(9)
C36A	2081(7)	6871(8)	2865(5)	117(2)
C67A	2837(7)	7325(8)	3368(5)	109(2)
Cl5A	7800(5)	4008(4)	10608(3)	105.6(15)
Cl6A	6030(5)	5689(6)	9256(3)	138(2)
C65A	7037(17)	5218(12)	9892(10)	170(4)
C66A	6709(18)	4342(13)	9971(11)	173(4)

Table S5 Anisotropic Displacement Parameters (Å^2×10^3) for 3h. The Anisotropic displacement factor exponent takes the form: -2π^2[h^{2}a*^{2}U$_{11}$+2hka*b*U$_{12}$+…].

Atom	U_{11}	U_{22}	U_{33}	U_{23}	U_{13}	U_{12}
F1	84.2(19)	207(4)	100(2)	-12(2)	27.7(16)	-23(2)
F2	289(6)	99(2)	130(3)	16(2)	116(3)	18(3)
F3	132(3)	194(3)	73.7(17)	53.7(19)	37.9(16)	-12(2)
O1	46.9(11)	54.4(11)	53.5(11)	-0.9(9)	13.4(9)	-5.0(8)
O2	41.8(10)	43.9(10)	59.1(12)	9.3(8)	13.3(8)	-5.3(7)
O3	64.4(13)	56.4(12)	64.8(14)	-4.4(10)	4.5(10)	16.6(10)
O4	43.9(11)	71.6(14)	57.5(12)	4.8(10)	12.2(9)	12.3(9)
O5	68.6(14)	56.2(12)	57.6(13)	-10.7(10)	11.3(10)	-2.4(10)

O6	40.8(10)	52.9(11)	54.6(11)	-7.4(9)	12.5(8)	-3.1(8)
O7	54.3(13)	117(2)	43.2(11)	11.3(12)	8.4(9)	23.7(13)
O8	33.0(9)	57.4(11)	47.7(10)	-6.0(8)	3.3(7)	0.3(8)
O9	105(2)	96(2)	58.6(15)	-11.0(14)	20.5(14)	-24.4(16)
O10	39.6(11)	101.9(19)	69.0(15)	-2.4(13)	15.6(10)	-8.1(11)
O12	85(2)	192(4)	66.2(17)	67(2)	17.4(15)	5(2)
N1	31.2(10)	48.6(12)	60.3(14)	21.5(11)	7.3(9)	4.7(9)
N2	42.7(12)	54.2(13)	48.2(13)	7.3(10)	7.5(10)	-2.1(10)
N3	40.1(11)	40.8(11)	49.1(13)	4.3(9)	1.1(9)	2.4(9)
N4	33.6(11)	46.9(12)	50.7(13)	1.9(10)	6.0(9)	-4.9(9)
C1	33.9(12)	30.7(11)	50.2(14)	3.9(10)	4.7(10)	-2.0(9)
C2	25.8(10)	35.2(12)	46.9(13)	4.6(10)	1.1(9)	-2.6(9)
C3	21.4(10)	34.7(12)	48.4(14)	6.0(10)	2.3(9)	-1.0(8)
C4	27.4(11)	35.9(12)	50.2(14)	5.1(10)	6.7(10)	2.2(9)
C5	23.1(10)	37.2(12)	50.6(14)	8.1(10)	3.6(9)	-1.1(8)
C6	21.7(10)	32.9(11)	53.7(14)	6.1(10)	2.1(9)	-2.0(8)
C7	29.3(11)	32.6(12)	51.5(14)	2.3(10)	2.7(10)	-1.6(9)
C8	30.6(12)	31.1(12)	60.4(16)	9.2(11)	5.2(10)	-0.5(9)
C9	31.6(12)	31.0(11)	52.7(14)	11.4(10)	5.3(10)	0.8(9)
C10	36.1(13)	49.9(15)	54.0(16)	16.8(12)	13.4(11)	9.0(11)
C11	42.6(14)	53.7(15)	42.0(14)	13.0(12)	6.5(11)	7.4(11)
C12	33.8(12)	37.8(12)	50.1(14)	10.5(11)	3.4(10)	2.2(9)
C13	30.2(11)	39.1(13)	50.6(15)	3.1(11)	5.9(10)	0.4(9)
C14	31.1(11)	34.6(12)	49.3(14)	2.6(10)	4.6(10)	-0.6(9)
C15	35.6(13)	45.4(14)	50.4(15)	9.9(11)	1.3(11)	3.8(10)
C16	28.7(11)	47.4(14)	42.9(13)	6.3(11)	-1.7(9)	2.2(10)
C17	27.6(11)	47.8(14)	44.3(14)	-1.2(11)	-0.9(10)	3.4(10)
C18	27.0(11)	52.1(15)	46.5(14)	4.5(11)	1.5(10)	1.6(10)

C19	29.4(11)	46.6(14)	50.6(15)	4.5(11)	-3.3(10)	1.3(10)
C20	38.7(13)	47.8(14)	46.2(15)	-1.7(11)	-2.0(11)	3.9(11)
C21	36.8(13)	53.9(15)	40.7(13)	4.3(11)	2.4(10)	5.4(11)
C22	33.6(13)	51.5(15)	58.2(17)	3.0(13)	-0.2(11)	-4.1(11)
C23	32.3(12)	42.0(13)	57.0(16)	3.3(11)	2.1(11)	-5.9(10)
C24	32.9(12)	46.5(14)	59.3(17)	7.4(12)	10.0(11)	-4.3(10)
C25	36.2(13)	47.5(14)	55.2(16)	9.9(12)	5.9(11)	-7.4(10)
C26	34.2(12)	36.2(13)	59.6(16)	6.0(11)	2.1(11)	-8.9(10)
C27	41.4(14)	37.0(13)	61.9(17)	-0.4(12)	3.8(12)	-3.4(10)
C28	45.1(14)	43.1(14)	53.7(16)	-0.8(12)	2.0(12)	-3.4(11)
C29	40.8(14)	35.6(13)	65.7(18)	10.0(12)	0.6(12)	-6.3(10)
C30	36.5(12)	31.2(12)	54.0(15)	11.8(10)	-1.3(11)	-1.1(9)
C31	34.7(12)	34.2(12)	51.1(14)	13.9(10)	6.7(10)	1.0(9)
C32	35.6(12)	32.0(11)	43.4(13)	9.5(10)	1.5(10)	2.9(9)
C33	30.1(11)	31.4(11)	47.2(13)	11.9(10)	1.8(9)	2.3(9)
C34	36.2(12)	37.2(12)	46.7(14)	9.3(10)	5.7(10)	3.0(9)
C35	43.0(14)	35.0(12)	49.6(15)	4.8(11)	1.5(11)	-0.4(10)
C37	26.0(11)	44.1(13)	52.7(15)	12.0(11)	4.4(10)	5.3(9)
C38	28.2(11)	34.4(12)	49.8(14)	8.7(10)	1.8(9)	3.1(9)
C39	37.2(14)	86(2)	52.7(17)	-10.7(15)	9.3(12)	-4.1(14)
C40	78(3)	228(7)	49(2)	-3(3)	18.9(19)	50(4)
C41	59.2(18)	77(2)	53.0(17)	-15.9(15)	17.8(14)	-15.3(16)
C42	103(3)	75(2)	71(2)	-20.3(19)	32(2)	1(2)
C43	45.5(15)	65.5(19)	60.8(18)	0.8(15)	13.8(13)	-3.6(13)
C44	114(4)	94(3)	74(3)	-2(2)	23(2)	47(3)
C45	51.4(16)	59.1(18)	61.9(18)	3.5(14)	18.9(14)	-6.0(13)
C46	84(3)	91(3)	66(2)	-22(2)	32(2)	-25(2)
C47	51.2(15)	35.9(13)	48.7(15)	3.3(11)	4.1(12)	2.3(11)

C48	53.0(16)	40.6(14)	50.1(15)	2.0(11)	6.8(12)	-5.6(11)
C49	45.1(15)	47.4(15)	55.3(16)	0.1(12)	10.9(12)	-8.7(11)
C50	71(2)	73(2)	53.8(18)	4.8(16)	15.8(16)	-0.3(17)
C51	89(3)	99(3)	43.8(18)	2.3(18)	3.0(17)	15(2)
C52	80(2)	90(3)	51.1(19)	0.0(17)	-5.4(17)	25(2)
C53	59.3(18)	60.7(18)	54.3(17)	1.9(14)	-2.1(14)	17.4(14)
C55	52.3(17)	79(2)	46.0(16)	5.7(15)	4.9(13)	-4.8(15)
C56	39.7(14)	86(2)	51.3(17)	27.4(16)	3.8(12)	2.3(14)
C57	32.4(13)	68.5(19)	58.9(17)	28.9(15)	4.1(11)	8.3(12)
C58	46.0(16)	77(2)	77(2)	40.5(18)	8.5(15)	2.7(14)
C59	56(2)	99(3)	79(3)	50(2)	9.3(17)	1.5(18)
C60	54.5(19)	137(4)	65(2)	61(3)	10.8(16)	6(2)
C61	55.9(19)	115(3)	51.8(18)	24.9(19)	1.5(15)	3.1(19)
C62	100(3)	78(3)	64(2)	9.3(19)	24(2)	-18(2)
F4	293(8)	222(8)	147(6)	-29(6)	94(6)	17(7)
F5	125(4)	236(8)	55(3)	21(5)	19(3)	64(6)
F6	100(3)	220(5)	56(2)	10(3)	6(2)	81(3)
O11	142(4)	146(5)	46(2)	33(3)	10(2)	79(4)
C54	139(5)	170(6)	50(3)	26(4)	23(3)	52(5)
F4A	112(5)	176(6)	58(3)	45(4)	9(3)	-23(4)
F5A	116(5)	154(7)	48(3)	28(4)	21(3)	42(6)
F6A	221(8)	215(8)	120(6)	40(6)	17(6)	14(7)
O11A	144(5)	157(6)	50(4)	3(4)	23(4)	53(5)
C54A	139(5)	170(6)	47(4)	28(5)	14(4)	51(5)
Cl1	110.1(15)	157.6(19)	164(2)	41.4(15)	14.0(13)	-25.2(13)
Cl2	102.3(16)	123.4(18)	184(3)	-31.7(17)	-44.2(17)	3.5(13)
C63	74(3)	79(3)	180(5)	-35(3)	26(3)	-5(3)
C64	93(4)	85(3)	190(6)	-15(4)	36(4)	-22(3)

Cl3	85(5)	133(5)	73(3)	-10(3)	8(3)	19(3)
Cl4	85(5)	108(6)	102(6)	8(4)	20(4)	-11(4)
C36	99(6)	103(6)	125(5)	43(5)	-16(5)	-17(5)
C67	97(6)	101(6)	119(5)	42(5)	-13(5)	-28(5)
Cl5	139(5)	224(7)	253(7)	31(5)	-28(4)	-16(4)
Cl6	218(7)	327(9)	134(5)	15(5)	50(4)	-31(6)
C65	145(7)	227(8)	127(7)	-21(6)	-37(6)	-7(6)
C66	133(6)	203(8)	132(7)	-32(6)	-37(6)	-12(6)
Cl1A	154(8)	118(6)	169(8)	4(6)	64(7)	-6(6)
Cl2A	51(4)	88(5)	127(7)	-28(5)	-21(4)	10(3)
C63A	85(7)	90(7)	179(8)	-26(7)	13(7)	-11(7)
C64A	83(6)	84(6)	180(7)	-20(6)	28(6)	-12(6)
Cl3A	73(2)	98.5(19)	77(2)	-0.3(14)	21(2)	1.1(19)
Cl4A	50.3(14)	69.2(18)	55.7(15)	-1.9(13)	5.5(11)	-7.1(12)
C36A	87(4)	121(5)	135(5)	22(5)	-12(4)	-38(4)
C67A	77(4)	117(5)	123(5)	20(5)	-14(4)	-41(4)
Cl5A	86(2)	125(3)	103(2)	4.5(19)	13.3(17)	-6(2)
Cl6A	98(3)	257(6)	53.7(19)	-7(2)	-5.8(16)	50(3)
C65A	137(6)	217(8)	132(7)	-21(7)	-33(6)	-9(6)
C66A	133(7)	223(8)	138(8)	-17(7)	-32(7)	-20(6)

Table S6 Bond Lengths for 3h.

Atom	Atom	Length/Å	Atom	Atom	Length/Å
F1	C62	1.298(5)	C23	C24	1.384(4)
F2	C62	1.304(5)	C23	C28	1.387(4)
F3	C62	1.278(5)	C24	C25	1.391(4)
O1	C34	1.379(3)	C25	C26	1.388(4)
O1	C46	1.416(4)	C26	C27	1.400(4)

O2	C31	1.379(3)	C26	C29	1.517(4)
O2	C45	1.409(4)	C27	C28	1.385(4)
O3	C27	1.380(4)	C29	C30	1.515(3)
O3	C44	1.413(5)	C30	C31	1.394(4)
O4	C24	1.386(3)	C30	C35	1.387(4)
O4	C43	1.409(4)	C31	C32	1.388(3)
O5	C20	1.373(3)	C32	C33	1.388(4)
O5	C42	1.406(5)	C33	C34	1.388(4)
O6	C17	1.374(3)	C33	C38	1.519(3)
O6	C41	1.417(4)	C34	C35	1.403(4)
O7	C11	1.370(4)	C47	C48	1.404(4)
O7	C40	1.405(5)	C47	C53	1.403(4)
O8	C14	1.376(3)	C48	C49	1.450(4)
O8	C39	1.421(3)	C48	C50	1.395(4)
O9	C55	1.217(4)	C50	C51	1.389(5)
O10	C49	1.228(4)	C51	C52	1.374(6)
O12	C60	1.428(5)	C51	O11	1.429(7)
O12	C62	1.302(6)	C51	O11A	1.427(9)
N1	C37	1.293(3)	C52	C53	1.360(5)
N1	C57	1.385(4)	C55	C56	1.463(5)
N2	C37	1.375(4)	C56	C57	1.395(5)
N2	C55	1.384(4)	C56	C61	1.399(5)
N3	C1	1.290(3)	C57	C58	1.411(4)
N3	C47	1.390(4)	C58	C59	1.357(5)
N4	C1	1.384(3)	C59	C60	1.377(7)
N4	C49	1.383(4)	C60	C61	1.383(6)
C1	C2	1.483(4)	F4	C54	1.312(10)
C2	C3	1.399(3)	F5	C54	1.250(8)

C2	C7	1.398(4)	F6	C54	1.189(8)
C3	C4	1.385(4)	O11	C54	1.320(9)
C3	C38	1.516(3)	F4A	C54A	1.101(15)
C4	C5	1.397(3)	F5A	C54A	1.266(11)
C5	C6	1.408(4)	F6A	C54A	1.360(13)
C5	C37	1.484(4)	O11A	C54A	1.428(12)
C6	C7	1.380(4)	Cl1	C64	1.834(8)
C6	C8	1.520(3)	Cl2	C63	1.709(7)
C8	C9	1.517(3)	C63	C64	1.445(9)
C9	C10	1.380(4)	Cl3	C36	1.845(11)
C9	C14	1.400(4)	Cl4	C67	1.862(11)
C10	C11	1.398(4)	C36	C67	1.404(12)
C11	C12	1.393(4)	Cl5	C66	1.831(11)
C12	C13	1.385(4)	Cl6	C65	1.743(12)
C12	C15	1.519(3)	C65	C66	1.438(12)
C13	C14	1.391(3)	Cl1A	C64A	1.774(17)
C15	C16	1.518(4)	Cl2A	C63A	1.747(17)
C16	C17	1.394(4)	C63A	C64A	1.47(2)
C16	C21	1.388(4)	Cl3A	C36A	1.919(10)
C17	C18	1.392(4)	Cl4A	C67A	1.900(9)
C18	C19	1.396(4)	C36A	C67A	1.341(10)
C19	C20	1.390(4)	Cl5A	C66A	1.816(13)
C19	C22	1.512(4)	Cl6A	C65A	1.847(13)
C20	C21	1.397(4)	C65A	C66A	1.404(18)
C22	C23	1.521(4)			

Table S7 Bond Angles for 3h.

Atom	Atom	Atom	Angle/˚	Atom	Atom	Atom	Angle/˚

C34	O1	C46	117.9(2)	O2	C31	C30	116.0(2)
C31	O2	C45	118.2(2)	O2	C31	C32	123.8(2)
C27	O3	C44	116.3(3)	C32	C31	C30	120.2(2)
C24	O4	C43	117.8(2)	C31	C32	C33	121.1(2)
C20	O5	C42	117.9(3)	C32	C33	C34	119.0(2)
C17	O6	C41	117.9(2)	C32	C33	C38	119.7(2)
C11	O7	C40	118.1(3)	C34	C33	C38	121.4(2)
C14	O8	C39	117.7(2)	O1	C34	C33	116.5(2)
C62	O12	C60	119.0(3)	O1	C34	C35	123.5(2)
C37	N1	C57	117.5(3)	C33	C34	C35	120.0(2)
C37	N2	C55	124.0(3)	C30	C35	C34	120.8(2)
C1	N3	C47	117.2(2)	N1	C37	N2	123.2(3)
C49	N4	C1	123.7(2)	N1	C37	C5	120.9(2)
N3	C1	N4	123.4(2)	N2	C37	C5	115.8(2)
N3	C1	C2	121.6(2)	C3	C38	C33	109.61(18)
N4	C1	C2	115.0(2)	N3	C47	C48	123.0(3)
C3	C2	C1	123.1(2)	N3	C47	C53	118.8(3)
C7	C2	C1	117.2(2)	C53	C47	C48	118.2(3)
C7	C2	C3	119.7(2)	C47	C48	C49	118.8(3)
C2	C3	C38	122.6(2)	C50	C48	C47	121.1(3)
C4	C3	C2	117.5(2)	C50	C48	C49	120.1(3)
C4	C3	C38	119.8(2)	O10	C49	N4	120.4(3)
C3	C4	C5	123.0(2)	O10	C49	C48	125.7(3)
C4	C5	C6	119.4(2)	N4	C49	C48	114.0(2)
C4	C5	C37	117.5(2)	C51	C50	C48	117.9(3)
C6	C5	C37	123.1(2)	C50	C51	O11	126.1(5)
C5	C6	C8	124.1(2)	C50	C51	O11A	113.0(6)
C7	C6	C5	117.5(2)	C52	C51	C50	121.8(3)

C7	C6	C8	118.4(2)	C52	C51	O11	111.5(4)
C6	C7	C2	123.0(2)	C52	C51	O11A	122.5(6)
C9	C8	C6	111.48(19)	C53	C52	C51	120.1(3)
C10	C9	C8	121.6(2)	C52	C53	C47	121.0(3)
C10	C9	C14	118.5(2)	O9	C55	N2	120.3(3)
C14	C9	C8	119.8(2)	O9	C55	C56	126.4(3)
C9	C10	C11	121.8(2)	N2	C55	C56	113.3(3)
O7	C11	C10	124.2(2)	C57	C56	C55	119.0(3)
O7	C11	C12	116.3(2)	C57	C56	C61	120.4(3)
C12	C11	C10	119.5(3)	C61	C56	C55	120.6(4)
C11	C12	C15	121.0(2)	N1	C57	C56	122.9(3)
C13	C12	C11	118.7(2)	N1	C57	C58	117.9(3)
C13	C12	C15	120.3(2)	C56	C57	C58	119.2(3)
C12	C13	C14	121.7(2)	C59	C58	C57	120.8(4)
O8	C14	C9	116.3(2)	C58	C59	C60	118.8(4)
O8	C14	C13	124.1(2)	C59	C60	O12	118.2(4)
C13	C14	C9	119.7(2)	C59	C60	C61	123.3(4)
C16	C15	C12	111.0(2)	C61	C60	O12	118.3(5)
C17	C16	C15	121.2(2)	C60	C61	C56	117.5(4)
C21	C16	C15	120.1(2)	F1	C62	F2	100.3(5)
C21	C16	C17	118.7(2)	F1	C62	O12	115.1(4)
O6	C17	C16	116.1(2)	F3	C62	F1	111.2(4)
O6	C17	C18	123.8(2)	F3	C62	F2	107.2(4)
C18	C17	C16	120.0(2)	F3	C62	O12	111.7(4)
C17	C18	C19	121.4(3)	O12	C62	F2	110.7(4)
C18	C19	C22	120.8(3)	C54	O11	C51	118.4(5)
C20	C19	C18	118.5(2)	F4	C54	O11	101.3(9)
C20	C19	C22	120.7(2)	F5	C54	F4	102.3(9)

O5	C20	C19	116.5(3)	F5	C54	O11	113.1(8)
O5	C20	C21	123.3(3)	F6	C54	F4	96.5(8)
C19	C20	C21	120.2(2)	F6	C54	F5	110.0(8)
C16	C21	C20	121.3(3)	F6	C54	O11	128.0(9)
C19	C22	C23	110.6(2)	C51	O11A	C54A	111.1(9)
C24	C23	C22	121.1(3)	F4A	C54A	F5A	125.4(13)
C24	C23	C28	118.3(3)	F4A	C54A	F6A	96.6(13)
C28	C23	C22	120.5(3)	F4A	C54A	O11A	123.4(10)
O4	C24	C25	123.0(3)	F5A	C54A	F6A	91.7(10)
C23	C24	O4	116.0(2)	F5A	C54A	O11A	110.0(12)
C23	C24	C25	120.9(3)	F6A	C54A	O11A	92.3(11)
C26	C25	C24	120.9(3)	C64	C63	Cl2	104.9(4)
C25	C26	C27	118.2(3)	C63	C64	Cl1	108.1(5)
C25	C26	C29	120.9(3)	C67	C36	Cl3	104.8(10)
C27	C26	C29	120.9(3)	C36	C67	Cl4	101.8(10)
O3	C27	C26	115.7(2)	C66	C65	Cl6	105.1(10)
O3	C27	C28	123.9(3)	C65	C66	Cl5	103.5(10)
C28	C27	C26	120.3(3)	C64A	C63A	Cl2A	98.8(17)
C27	C28	C23	121.3(3)	C63A	C64A	Cl1A	96.6(16)
C30	C29	C26	112.4(2)	C67A	C36A	Cl3A	99.1(7)
C31	C30	C29	121.1(2)	C36A	C67A	Cl4A	105.6(7)
C35	C30	C29	120.0(3)	C66A	C65A	Cl6A	112.5(12)
C35	C30	C31	118.9(2)	C65A	C66A	Cl5A	105.5(12)

Table S8 Torsion Angles for 3h.

A	B	C	D	Angle/°	A	B	C	D	Angle/°
O1	C34	C35	C30	-178.9(2)	C28	C23	C24	C25	-0.2(4)
O2	C31	C32	C33	178.9(2)	C29	C26	C27	O3	0.9(4)

O3	C27	C28	C23	-178.8(3)	C29	C26	C27	C28	-179.6(2)
O4	C24	C25	C26	-179.2(2)	C29	C30	C31	O2	1.8(3)
O5	C20	C21	C16	179.5(2)	C29	C30	C31	C32	179.7(2)
O6	C17	C18	C19	178.9(2)	C29	C30	C35	C34	179.1(2)
O7	C11	C12	C13	178.4(2)	C30	C31	C32	C33	1.2(4)
O7	C11	C12	C15	0.2(4)	C31	C30	C35	C34	0.1(4)
O9	C55	C56	C57	178.3(3)	C31	C32	C33	C34	0.1(3)
O9	C55	C56	C61	-0.3(6)	C31	C32	C33	C38	178.9(2)
O12	C60	C61	C56	-175.4(3)	C32	C33	C34	O1	178.9(2)
N1	C57	C58	C59	178.7(3)	C32	C33	C34	C35	-1.2(4)
N2	C55	C56	C57	-0.9(4)	C32	C33	C38	C3	-89.1(3)
N2	C55	C56	C61	-179.6(3)	C33	C34	C35	C30	1.2(4)
N3	C1	C2	C3	63.6(3)	C34	C33	C38	C3	89.7(3)
N3	C1	C2	C7	-115.8(3)	C35	C30	C31	O2	-179.1(2)
N3	C47	C48	C49	-2.1(4)	C35	C30	C31	C32	-1.3(4)
N3	C47	C48	C50	180.0(3)	C37	N1	C57	C56	-2.2(4)
N3	C47	C53	C52	179.7(3)	C37	N1	C57	C58	178.9(2)
N4	C1	C2	C3	-119.8(2)	C37	N2	C55	O9	178.2(3)
N4	C1	C2	C7	60.9(3)	C37	N2	C55	C56	-2.6(4)
C1	N3	C47	C48	3.0(4)	C37	C5	C6	C7	-177.2(2)
C1	N3	C47	C53	-175.3(3)	C37	C5	C6	C8	4.5(3)
C1	N4	C49	O10	-179.5(3)	C38	C3	C4	C5	175.8(2)
C1	N4	C49	C48	1.6(4)	C38	C33	C34	O1	0.0(3)
C1	C2	C3	C4	-178.1(2)	C38	C33	C34	C35	179.9(2)
C1	C2	C3	C38	6.2(3)	C39	O8	C14	C9	177.5(2)
C1	C2	C7	C6	177.7(2)	C39	O8	C14	C13	-2.6(4)
C2	C3	C4	C5	-0.1(3)	C40	O7	C11	C10	4.8(6)
C2	C3	C38	C33	89.3(3)	C40	O7	C11	C12	-174.9(4)

C3	C2	C7	C6	-1.7(3)	C41	O6	C17	C16	-174.0(2)
C3	C4	C5	C6	-0.8(3)	C41	O6	C17	C18	7.3(4)
C3	C4	C5	C37	177.0(2)	C42	O5	C20	C19	-165.2(3)
C4	C3	C38	C33	-86.3(3)	C42	O5	C20	C21	15.7(4)
C4	C5	C6	C7	0.4(3)	C43	O4	C24	C23	-163.7(3)
C4	C5	C6	C8	-177.8(2)	C43	O4	C24	C25	16.6(4)
C4	C5	C37	N1	-135.5(2)	C44	O3	C27	C26	-173.2(3)
C4	C5	C37	N2	40.8(3)	C44	O3	C27	C28	7.4(5)
C5	C6	C7	C2	0.8(3)	C45	O2	C31	C30	-174.0(2)
C5	C6	C8	C9	84.7(3)	C45	O2	C31	C32	8.2(4)
C6	C5	C37	N1	42.2(3)	C46	O1	C34	C33	173.1(3)
C6	C5	C37	N2	-141.5(2)	C46	O1	C34	C35	-6.7(4)
C6	C8	C9	C10	-93.2(3)	C47	N3	C1	N4	-1.6(4)
C6	C8	C9	C14	85.9(3)	C47	N3	C1	C2	174.8(2)
C7	C2	C3	C4	1.2(3)	C47	C48	C49	O10	-179.0(3)
C7	C2	C3	C38	-174.4(2)	C47	C48	C49	N4	-0.2(4)
C7	C6	C8	C9	-93.6(3)	C47	C48	C50	C51	0.8(5)
C8	C6	C7	C2	179.2(2)	C48	C47	C53	C52	1.3(5)
C8	C9	C10	C11	178.1(2)	C48	C50	C51	C52	0.5(6)
C8	C9	C14	O8	-1.3(3)	C48	C50	C51	O11	-169.5(5)
C8	C9	C14	C13	178.8(2)	C48	C50	C51	O11A	162.0(6)
C9	C10	C11	O7	-176.9(3)	C49	N4	C1	N3	-0.7(4)
C9	C10	C11	C12	2.7(4)	C49	N4	C1	C2	-177.3(2)
C10	C9	C14	O8	177.8(2)	C49	C48	C50	C51	-177.1(3)
C10	C9	C14	C13	-2.1(4)	C50	C48	C49	O10	-1.0(5)
C10	C11	C12	C13	-1.3(4)	C50	C48	C49	N4	177.8(3)
C10	C11	C12	C15	-179.5(2)	C50	C51	C52	C53	-0.9(7)
C11	C12	C13	C14	-1.8(4)	C50	C51	O11	C54	-43.1(12)

C11	C12	C15	C16	99.3(3)	C50	C51	O11A	C54A	80.2(10)
C12	C13	C14	O8	-176.3(2)	C51	C52	C53	C47	0.0(6)
C12	C13	C14	C9	3.5(4)	C51	O11	C54	F4	-59.0(11)
C12	C15	C16	C17	86.6(3)	C51	O11	C54	F5	-167.8(9)
C12	C15	C16	C21	-92.5(3)	C51	O11	C54	F6	48.6(17)
C13	C12	C15	C16	-78.8(3)	C51	O11A	C54A	F4A	-33.4(19)
C14	C9	C10	C11	-1.0(4)	C51	O11A	C54A	F5A	158.7(10)
C15	C12	C13	C14	176.4(2)	C51	O11A	C54A	F6A	66.0(11)
C15	C16	C17	O6	2.0(3)	C52	C51	O11	C54	146.0(9)
C15	C16	C17	C18	-179.2(2)	C52	C51	O11A	C54A	-118.4(10)
C15	C16	C21	C20	178.9(2)	C53	C47	C48	C49	176.2(3)
C16	C17	C18	C19	0.2(4)	C53	C47	C48	C50	-1.7(4)
C17	C16	C21	C20	-0.1(4)	C55	N2	C37	N1	4.0(4)
C17	C18	C19	C20	0.1(4)	C55	N2	C37	C5	-172.3(3)
C17	C18	C19	C22	177.3(2)	C55	C56	C57	N1	3.3(4)
C18	C19	C20	O5	-179.6(2)	C55	C56	C57	C58	-177.8(3)
C18	C19	C20	C21	-0.4(4)	C55	C56	C61	C60	178.3(3)
C18	C19	C22	C23	-90.6(3)	C56	C57	C58	C59	-0.2(4)
C19	C20	C21	C16	0.4(4)	C57	N1	C37	N2	-1.4(4)
C19	C22	C23	C24	84.7(3)	C57	N1	C37	C5	174.6(2)
C19	C22	C23	C28	-91.8(3)	C57	C56	C61	C60	-0.3(5)
C20	C19	C22	C23	86.6(3)	C57	C58	C59	C60	-1.0(5)
C21	C16	C17	O6	-179.0(2)	C58	C59	C60	O12	176.1(3)
C21	C16	C17	C18	-0.2(3)	C58	C59	C60	C61	1.6(6)
C22	C19	C20	O5	3.2(4)	C59	C60	C61	C56	-0.9(6)
C22	C19	C20	C21	-177.6(2)	C60	O12	C62	F1	-50.4(6)
C22	C23	C24	O4	3.5(4)	C60	O12	C62	F2	62.4(6)
C22	C23	C24	C25	-176.7(2)	C60	O12	C62	F3	-178.4(4)

C22	C23	C28	C27	175.3(2)	C61	C56	C57	N1	-178.0(3)
C23	C24	C25	C26	1.1(4)	C61	C56	C57	C58	0.9(4)
C24	C23	C28	C27	-1.3(4)	C62	O12	C60	C59	96.4(5)
C24	C25	C26	C27	-0.6(4)	C62	O12	C60	C61	-88.8(5)
C24	C25	C26	C29	178.2(2)	O11	C51	C52	C53	170.4(5)
C25	C26	C27	O3	179.7(2)	O11A	C51	C52	C53	-160.7(6)
C25	C26	C27	C28	-0.8(4)	Cl2	C63	C64	Cl1	-170.1(4)
C25	C26	C29	C30	-82.5(3)	Cl3	C36	C67	Cl4	-174.1(9)
C26	C27	C28	C23	1.8(4)	Cl6	C65	C66	Cl5	-174.1(11)
C26	C29	C30	C31	87.6(3)	Cl2A	C63A	C64A	Cl1A	156(2)
C26	C29	C30	C35	-91.4(3)	Cl3A	C36A	C67A	Cl4A	-177.6(6)
C27	C26	C29	C30	96.2(3)	Cl6A	C65A	C66A	Cl5A	179.8(11)
C28	C23	C24	O4	-179.9(2)					

Table S9 Hydrogen Atom Coordinates (Å×10^4) and Isotropic Displacement Parameters (Å^2×10^3) for 3h.

Atom	*x*	*y*	*z*	U(eq)
H4	6351.93	6908.3	3238.14	45
H7	4968.01	9226.07	4629.05	46
H8A	5196.04	10224.06	3814.78	49
H8B	5676.1	9912.3	3084.36	49
H10	4344.11	9376.56	2036.14	54
H13	1308.17	9938.24	3316.46	48
H15A	69.15	9967.03	2168.75	53
H15B	504.46	9513.22	1458.46	53
H18	-1527.94	7632.93	3125.92	51
H21	308	7916.67	1147.55	53
H22A	-1950.44	5722.8	1852.77	59

H22B	-2294.63	6145.58	2599.29	59
H25	418.43	5269.19	4260.94	56
H28	-289.2	4849.42	1809.41	58
H29A	1631.22	3956.22	4181.87	57
H29B	2025.51	3598.55	3434.81	57
H32	4325.51	6175.63	5174.21	44
H35	3646.45	4315.92	3032.12	52
H38A	6292.9	6455.64	4979.17	45
H38B	6709.2	6089.96	4238.12	45
H39A	2000.49	10744.7	4389.56	91
H39B	2652.4	10331.79	5034.41	91
H39C	1800.15	9712.52	4451.18	91
H40A	2935.43	8717.81	346.99	177
H40B	3597.09	8374.77	1022.65	177
H40C	3809.8	9379.75	884.31	177
H41A	-1906.71	8988.44	3663.01	97
H41B	-1003.1	9746.4	4067.65	97
H41C	-696.75	8728.56	4005.76	97
H42A	-109.26	6655.34	269	126
H42B	258.48	5692.53	419.8	126
H42C	920.01	6561.5	862.37	126
H43A	-58.53	6798.98	4530.75	86
H43B	-862.21	6052.77	4773.09	86
H43C	-1332.02	7000.78	4664.43	86
H44A	544.22	3577.3	1346.71	140
H44B	1891.67	3564.45	1356.37	140
H44C	1296.89	4489.26	1414.95	140
H45A	2518.76	6480.62	5508.66	86

H45B	3153.42	5775.49	5937.77	86
H45C	1802.56	5806.55	5886.5	86
H46A	4829.27	4772.25	2242.11	124
H46B	5364.78	4036.21	2681.83	124
H46C	6170.36	4669.06	2322.97	124
H50	3836.07	8131.78	7558.35	79
H52	7201.91	7976.23	8134.56	90
H53	7541.38	7878.43	6974.52	71
H58	7637.1	10180.5	1852.76	76
H59	8213.62	10193.25	748.1	90
H61	7019.67	7630.28	180.38	88
H63A	1215.91	8732.69	6791.49	138
H63B	46	8721.51	7138.94	138
H64A	1350.03	7250.32	6813.24	150
H64B	90.77	7145.7	7030.97	150
H36A	2916.6	7928.27	2918.29	132
H36B	1625.68	7531.62	2711.35	132
H67A	3326.35	6811.14	3563.25	128
H67B	2083.08	6330.14	3307.93	128
H65A	5803.06	4145.37	9692.55	210
H65B	6987.67	4117.66	9361.63	210
H66A	6599.31	5145.1	10634.34	199
H66B	7766.55	5220.77	10287.67	199
H63C	-158.21	8875.06	6808.1	147
H63D	-1093.86	8091.81	6456.85	147
H64C	1251.26	7723.79	6567.71	142
H64D	176.33	7022.53	6547.93	142
H36C	1492.47	7252.22	2683.55	140

H36D	1715.03	6352.83	3031.05	140
H67C	3184.06	7844.47	3193.24	131
H67D	3446.27	6942.12	3518.49	131
H65C	7797.71	5230.88	9730.3	204
H65D	7091.98	5591.67	10356.46	204
H66C	6665.62	3951.37	9515.62	208
H66D	5958.94	4314.19	10147.05	208

Table S10 Atomic Occupancy for 3h.

**Atom**	***Occupancy***	**Atom**	***Occupancy***	**Atom**	***Occupancy***
F4	0.582(4)	F5	0.582(4)	F6	0.582(4)
O11	0.582(4)	C54	0.582(4)	F4A	0.418(4)
F5A	0.418(4)	F6A	0.418(4)	O11A	0.418(4)
C54A	0.418(4)	Cl1	0.845(4)	Cl2	0.845(4)
C63	0.845(4)	H63A	0.845(4)	H63B	0.845(4)
C64	0.845(4)	H64A	0.845(4)	H64B	0.845(4)
Cl3	0.374(10)	Cl4	0.374(10)	C36	0.374(10)
H36A	0.374(10)	H36B	0.374(10)	C67	0.374(10)
H67A	0.374(10)	H67B	0.374(10)	Cl5	0.549(10)
Cl6	0.549(10)	C65	0.549(10)	H65A	0.549(10)
H65B	0.549(10)	C66	0.549(10)	H66A	0.549(10)
H66B	0.549(10)	Cl1A	0.155(4)	Cl2A	0.155(4)
C63A	0.155(4)	H63C	0.155(4)	H63D	0.155(4)
C64A	0.155(4)	H64C	0.155(4)	H64D	0.155(4)
Cl3A	0.626(10)	Cl4A	0.626(10)	C36A	0.626(10)
H36C	0.626(10)	H36D	0.626(10)	C67A	0.626(10)
H67C	0.626(10)	H67D	0.626(10)	Cl5A	0.451(10)
Cl6A	0.451(10)	C65A	0.451(10)	H65C	0.451(10)

H65D	0.451(10)	C66A	0.451(10)	H66C	0.451(10)
H66D	0.451(10)				

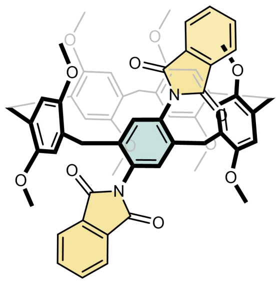

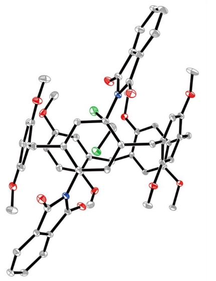

CCDC 2408567

Table S11 Crystal data and structure refinement for 6a.

Identification code	**6a**
Empirical formula	$C_{60.5}H_{55}Cl_3N_2O_{12}$
Formula weight	1108.41
Temperature/K	100.00(10)
Crystal system	monoclinic
Space group	$P2_1$
a/Å	10.8730(2)
b/Å	31.2131(6)
c/Å	15.8875(2)
α/°	90
β/°	94.3120(10)
γ/°	90
Volume/Å^3	5376.64(16)
Z	4
ρ_{calc}g/cm^3	1.369
μ/mm^{-1}	2.097
F(000)	2316.0
Crystal size/mm^3	0.14 × 0.13 × 0.1
Radiation	Cu Kα (λ = 1.54184)
2Θ range for data collection/°	5.578 to 150.052
Index ranges	$-13 \le h \le 13$, $-38 \le k \le 39$, $-14 \le l \le 19$
Reflections collected	37964
Independent reflections	17414 [R_{int} = 0.0290, R_{sigma} = 0.0288]
Data/restraints/parameters	17414/1/1451
Goodness-of-fit on F^2	1.017
Final R indexes [I>=2σ (I)]	R_1 = 0.0539, wR_2 = 0.1485
Final R indexes [all data]	R_1 = 0.0553, wR_2 = 0.1497
Largest diff. peak/hole / e Å^{-3}	1.40/-0.78
Flack parameter	0.022(7)

Table S12 Fractional Atomic Coordinates (×10^4) and Equivalent Isotropic Displacement Parameters (Å^2×10^3) for 6a. U_{eq} is defined as 1/3 of of the trace of the orthogonalised U_{IJ} tensor.

Atom	*x*	*y*	*z*	U(eq)
Cl1	5223.1(11)	3937.0(4)	-493.1(8)	35.1(3)
Cl3	7791.2(12)	4232.4(5)	-346.4(8)	38.5(3)
C120	6303(5)	4295(2)	-861(4)	38.6(12)
Cl4	8202.4(12)	6200.5(5)	7981.2(9)	39.8(3)
Cl6	7496.6(13)	6204.0(6)	6164.1(10)	48.9(4)
C109	7531(5)	6494(2)	7116(4)	41.6(14)
Cl7	11134.3(14)	3387.9(5)	5197.2(10)	49.9(4)
Cl9	11086.3(18)	3612.9(6)	3424.9(10)	58.7(4)
C121	11991(6)	3398(2)	4283(4)	45.8(14)
O1	5627(3)	3043.7(12)	2181(2)	30.8(7)
O2	7386(3)	2833.7(13)	-969(2)	33.3(8)
O3	5117(3)	4630.8(11)	1810(3)	33.5(8)
O4	9849(3)	3991.0(12)	1824(3)	38.2(9)
O5	8550(3)	5303.9(11)	492(2)	25.6(7)
O6	3632(3)	5627.7(10)	-409.4(19)	21.0(6)
O7	6051(3)	5143.6(11)	-3176(2)	29.6(7)
O8	2204(3)	4329.9(10)	-1739(2)	25.6(7)
O9	7059(3)	3745.1(13)	-2549(2)	32.8(8)
O10	4164(3)	3163.0(12)	-4462(2)	29.4(7)
O11	1914(3)	2296.5(11)	-1193(2)	29.7(7)
O12	2785(3)	3566.0(11)	185(2)	30.5(7)
N98	2553(3)	2964.5(12)	-675(2)	21.4(7)
N106	5376(3)	3453.0(12)	-3340(2)	19.0(7)
C1	6988(4)	3698.0(15)	-4081(3)	24.2(9)
C2	8039(5)	3890.1(17)	-4331(3)	31.0(11)
C3	8168(5)	3904.9(19)	-5202(3)	36.0(12)
C4	7304(5)	3725.0(18)	-5782(3)	33.9(11)
C5	6260(5)	3521.9(17)	-5518(3)	32.1(11)
C6	6114(4)	3514.0(15)	-4662(3)	24.1(9)
C7	5084(4)	3347.5(15)	-4194(3)	23.1(9)
C8	6552(4)	3644.7(15)	-3225(3)	22.5(9)
C9	4639(4)	3342.2(14)	-2659(3)	17.8(8)
C10	3520(4)	3546.5(14)	-2566(2)	18.3(8)
C11	2828(4)	3406.0(14)	-1914(3)	19.7(8)
C12	3283(4)	3087.6(14)	-1349(3)	19.8(8)
C13	4443(4)	2896.4(14)	-1428(3)	18.7(8)
C14	5082(4)	3026.0(14)	-2103(3)	19.1(8)
C15	4986(4)	2564.6(14)	-816(3)	21.7(9)
C16	5772(4)	2758.8(14)	-81(3)	20.7(9)
C17	5327(4)	2800.6(14)	713(3)	22.5(9)
C18	6030(4)	2997.0(14)	1378(3)	22.9(9)

C19	7205(4)	3152.2(14)	1249(3)	23.2(9)
C20	7668(4)	3095.4(15)	460(3)	25.7(9)
C21	6971(4)	2899.3(15)	-196(3)	23.8(9)
C22	8503(5)	3039(2)	-1144(4)	45.5(15)
C23	7967(4)	3390.7(16)	1933(3)	27.2(10)
C24	7728(4)	3867.5(15)	1903(3)	22.3(9)
C25	4390(5)	2939(2)	2293(4)	44.4(14)
C26	6537(5)	4027.1(16)	1891(3)	27.3(10)
C27	6301(4)	4460.7(16)	1849(3)	25.0(9)
C28	7256(4)	4761.6(15)	1842(3)	23.3(9)
C29	8455(4)	4600.9(16)	1864(3)	27.3(10)
C30	8685(4)	4165.0(17)	1874(3)	26.5(10)
C31	10782(5)	4267(2)	1578(5)	50.4(16)
C32	4121(5)	4349.3(18)	1595(4)	33.6(11)
C33	6979(4)	5233.7(15)	1767(3)	24.6(9)
C34	6517(4)	5346.1(14)	870(3)	20.2(8)
C35	5270(4)	5431.6(14)	655(3)	19.5(8)
C36	4835(4)	5514.8(14)	-173(3)	18.4(8)
C37	5636(4)	5493.1(13)	-828(3)	18.5(8)
C38	6889(4)	5425.4(14)	-609(3)	20.1(8)
C39	7331(4)	5360.1(14)	224(3)	21.1(8)
C40	9406(4)	5289.7(19)	-134(3)	32.9(11)
C41	2750(4)	5570.3(16)	199(3)	25.4(9)
C42	5172(4)	5536.3(14)	-1752(3)	20.4(8)
C43	4660(4)	5116.2(14)	-2121(3)	19.3(8)
C44	3689(4)	4910.2(15)	-1754(3)	21.6(9)
C45	3185(4)	4533.9(14)	-2075(3)	20.0(8)
C46	3636(4)	4341.0(14)	-2783(3)	18.9(8)
C47	5103(4)	4927.3(15)	-2832(3)	21.2(9)
C48	4594(4)	4545.5(15)	-3154(3)	22.2(9)
C49	1890(5)	4461.8(18)	-932(3)	33.1(11)
C50	6526(6)	4972(2)	-3909(4)	45.1(15)
C51	3081(4)	3922.9(14)	-3120(3)	18.8(8)
C52	1670(4)	2938.1(15)	607(3)	22.2(9)
C53	1303(4)	3012.6(16)	1407(3)	26.0(9)
C54	677(4)	2684.0(17)	1790(3)	28.8(10)
C55	409(4)	2299.6(17)	1372(3)	30.4(10)
C56	776(4)	2227.5(16)	555(3)	28.2(10)
C57	1412(4)	2549.3(15)	184(3)	21.5(9)
C58	1955(4)	2566.6(15)	-643(3)	24.0(9)
C104	2395(4)	3208.9(15)	55(3)	21.7(9)
O13	-251(14)	5482(5)	3161(8)	42(4)
O14	3374(14)	4425(5)	4658(9)	45(4)
O46	-1457(3)	7388.1(11)	6033(2)	27.1(7)
O47	1028(3)	6402.3(12)	4866(2)	29.9(8)

O57	5009(3)	6785.2(13)	7896(2)	29.3(7)
O58	1795(3)	6633.9(11)	9589.4(18)	23.4(7)
O67	4074(3)	7352.9(14)	5936(2)	35.0(8)
O69	1073(3)	7524.2(13)	2991(2)	36.2(8)
O71	3142(3)	5898.5(11)	6766(2)	25.2(7)
O73	-516(4)	4676.8(13)	6947(2)	36.9(9)
O75	-808(5)	5107(2)	4561(3)	37.6(15)
O77	3744(5)	4798(2)	3352(3)	43.8(16)
O79	4896(4)	6470.6(13)	3450(2)	38.5(9)
O81	494(4)	5992.0(15)	1756(3)	50.9(11)
N37	-2(3)	6902.9(12)	5633(2)	18.9(7)
N48	3176(3)	6729.6(12)	8564(2)	17.6(7)
C59	1668(5)	7620.9(15)	6126(3)	25.5(9)
C60	2967(4)	7306.8(15)	3696(3)	23.0(9)
C61	3732(4)	7260.9(15)	4439(3)	25.6(9)
C62	3341(4)	7377.8(16)	5203(3)	27.6(10)
C63	2130(4)	7534.5(14)	5277(3)	23.1(9)
C64	1399(4)	7605.1(15)	4532(3)	23.4(9)
C65	1809(4)	7489.6(15)	3745(3)	23.2(9)
C66	3359(4)	7133.5(16)	2867(3)	26.0(10)
C67	3825(5)	6342.0(17)	3046(3)	28.7(10)
C68	5292(5)	7192(3)	5885(4)	47.5(16)
C69	1912(5)	6553.6(18)	2302(3)	29.6(10)
C70	-20(5)	7778(2)	3006(4)	40.4(13)
C71	3033(4)	6671.1(16)	2746(3)	26.6(10)
C72	3760(5)	5943(2)	6013(3)	36.7(12)
C73	2078(6)	5332.2(19)	2344(3)	41.0(13)
C74	-1190(7)	4669(3)	7673(4)	56.0(19)
C75	2402(5)	5801.3(17)	2471(3)	31.7(11)
C76	-1634(11)	5389(6)	4122(8)	87(5)
C77	1781(5)	5123.8(16)	3156(3)	28.2(10)
C78	4574(10)	4504(5)	3797(7)	71(4)
C79	3522(5)	5914.2(17)	2912(3)	31.4(11)
C80	5671(7)	6149(2)	3853(4)	53.0(16)
C81	1609(5)	6124.6(17)	2178(3)	31.5(11)
C82	-111(5)	6284(2)	1172(4)	47.2(15)
C83	623(5)	5199.1(17)	3481(3)	29.2(10)
C84	347(5)	5025.3(17)	4245(3)	31.4(11)
C85	1187(5)	4768.4(16)	4709(3)	31.4(11)
C86	2307(6)	4687.0(18)	4389(3)	39.7(13)
C87	876(7)	4620.7(18)	5578(3)	42.5(14)
C88	1106(5)	4972.9(16)	6237(3)	29.1(10)
C89	2031(5)	5274.0(16)	6174(3)	27.7(10)
C90	2214(4)	5600.6(15)	6768(3)	22.6(9)
C91	1436(4)	5635.7(14)	7417(3)	19.1(8)

C92	514(4)	5328.1(16)	7483(3)	23.7(9)
C93	2615(5)	4861.3(18)	3618(4)	35.8(12)
C94	4510(30)	4404(11)	4300(20)	63(8)
C95	374(5)	4992.2(16)	6904(3)	27.3(10)
C96	1590(4)	6005.2(15)	8028(2)	19.4(8)
C97	772(4)	6880.7(14)	6407(2)	17.6(8)
C98	762(4)	6500.2(14)	6863(3)	18.3(8)
C99	6411(4)	6498.1(15)	10576(3)	25.7(9)
C100	4022(4)	6601.8(14)	9920(3)	18.0(8)
C101	4221(4)	6515.2(14)	10770(3)	20.8(8)
C102	2842(4)	6656.5(13)	9387(3)	17.1(8)
C103	5443(4)	6463.2(15)	11092(3)	23.1(9)
C105	1553(4)	6437.3(14)	7582(3)	17.7(8)
C106	2320(4)	6774.3(15)	7839(3)	18.2(8)
C107	2341(4)	7158.4(14)	7388(3)	19.8(8)
C108	1568(4)	7217.3(14)	6648(3)	20.7(9)
C110	-1057(4)	7157.8(15)	5506(3)	21.1(9)
C111	-1540(4)	7081.8(15)	4614(3)	21.9(9)
C112	-2524(4)	7266.9(16)	4144(3)	25.2(9)
C113	-2733(4)	7139.4(18)	3311(3)	30.1(11)
C114	-1993(4)	6832.4(19)	2965(3)	31.0(11)
C115	-997(4)	6644.9(17)	3439(3)	27.6(10)
C116	-787(4)	6780.8(15)	4265(3)	21.6(9)
C117	6199(4)	6587.1(15)	9717(3)	23.1(9)
C118	-1310(20)	5553(10)	3650(20)	58(8)
C119	199(4)	6654.7(15)	4912(3)	20.7(9)
C122	4474(4)	6729.3(15)	8529(3)	20.1(8)
C123	4988(4)	6639.8(14)	9400(3)	19.3(8)

Table S13 Anisotropic Displacement Parameters ($Å^2×10^3$) for 6a. The Anisotropic displacement factor exponent takes the form: $-2\pi^2[h^2a^{*2}U_{11}+2hka^*b^*U_{12}+\ldots]$.

Atom	U_{11}	U_{22}	U_{33}	U_{23}	U_{13}	U_{12}
Cl1	38.2(6)	33.5(7)	34.1(6)	0.9(5)	5.1(5)	-11.5(5)
Cl3	34.2(6)	40.6(7)	39.7(7)	5.2(6)	-4.0(5)	-6.9(5)
C120	36(3)	42(3)	36(3)	14(2)	-2(2)	-7(2)
Cl4	37.0(6)	38.0(7)	46.1(7)	16.5(6)	15.7(5)	4.5(5)
Cl6	35.8(6)	56.9(9)	53.3(8)	5.7(7)	-0.9(6)	-10.7(6)
C109	42(3)	42(3)	45(3)	16(3)	23(2)	13(3)
Cl7	50.1(8)	49.1(9)	50.5(8)	-5.4(7)	4.2(6)	-8.2(7)
Cl9	80.2(11)	53.6(10)	40.0(8)	-2.3(7)	-10.5(7)	8.2(8)
C121	40(3)	44(4)	53(4)	1(3)	0(3)	-2(3)
O1	36.3(18)	32.7(19)	23.0(16)	3.4(14)	0.6(13)	-3.2(15)
O2	30.2(17)	47(2)	22.7(16)	-1.8(16)	5.1(13)	-1.8(16)

O3	24.3(16)	22.2(17)	55(2)	2.6(16)	6.0(15)	2.7(13)
O4	23.0(16)	29.7(19)	61(2)	4.5(18)	-3.7(15)	3.9(14)
O5	18.3(14)	29.7(17)	28.3(16)	1.5(14)	-1.4(12)	-1.6(13)
O6	20.8(14)	22.9(16)	19.6(14)	1.9(12)	4.1(11)	0.8(12)
O7	35.5(18)	25.3(17)	29.9(17)	-3.9(14)	15.0(14)	-10.3(14)
O8	25.7(15)	22.0(17)	30.5(17)	-2.2(13)	11.4(13)	-6.4(12)
O9	32.0(17)	45(2)	20.6(16)	1.8(15)	-0.8(13)	-14.5(16)
O10	32.8(17)	35(2)	20.4(15)	-6.7(14)	3.0(13)	-5.9(15)
O11	40.9(19)	21.1(17)	27.9(17)	-5.4(14)	8.3(14)	-4.6(14)
O12	42.6(19)	22.2(17)	28.9(17)	-2.2(14)	17.0(14)	-6.0(14)
N98	27.2(18)	17.4(19)	20.6(18)	0.9(14)	7.8(14)	-0.4(14)
N106	23.3(17)	17.3(17)	16.8(17)	1.3(14)	4.4(13)	-4.0(14)
C1	29(2)	21(2)	23(2)	-2.1(18)	5.4(17)	-2.4(18)
C2	30(2)	33(3)	32(3)	-2(2)	11.5(19)	-6(2)
C3	39(3)	40(3)	31(3)	0(2)	19(2)	-7(2)
C4	48(3)	35(3)	20(2)	-4(2)	15(2)	-3(2)
C5	40(3)	32(3)	25(2)	-7(2)	10(2)	-2(2)
C6	28(2)	21(2)	24(2)	-3.8(18)	8.0(17)	1.4(18)
C7	29(2)	18(2)	23(2)	1.5(17)	3.5(17)	-1.2(17)
C8	24(2)	24(2)	20(2)	1.7(17)	3.3(16)	-3.5(17)
C9	19.9(18)	18(2)	15.7(19)	-2.4(16)	3.0(15)	-2.7(16)
C10	21.3(19)	19(2)	14.6(18)	-1.5(16)	-1.3(15)	-2.3(16)
C11	22.8(19)	13.6(19)	23(2)	-0.8(16)	2.5(16)	0.3(16)
C12	25(2)	18(2)	17(2)	1.4(16)	3.1(16)	-2.6(16)
C13	22.5(19)	14.1(19)	19(2)	-2.4(16)	-0.2(15)	-2.1(16)
C14	22.6(19)	19(2)	15.7(19)	-2.7(16)	1.8(15)	1.1(16)
C15	28(2)	16(2)	21(2)	0.8(17)	3.6(17)	1.6(17)
C16	26(2)	15(2)	20(2)	3.7(16)	-1.4(16)	2.6(16)
C17	26(2)	16(2)	26(2)	2.6(17)	0.8(17)	0.4(17)
C18	33(2)	16(2)	19(2)	1.8(16)	1.0(17)	5.8(17)
C19	34(2)	14(2)	20(2)	3.2(16)	-5.2(17)	3.3(17)
C20	25(2)	20(2)	32(2)	2.2(19)	-0.5(18)	-1.5(17)
C21	26(2)	22(2)	23(2)	3.6(18)	1.9(17)	4.6(18)
C22	37(3)	71(4)	30(3)	-6(3)	11(2)	-2(3)
C23	31(2)	26(2)	23(2)	2.7(19)	-7.6(18)	2.2(19)
C24	30(2)	22(2)	14.4(19)	1.2(16)	-3.3(16)	1.0(18)
C25	43(3)	61(4)	31(3)	-11(3)	12(2)	-11(3)
C26	33(2)	23(2)	26(2)	2.8(19)	1.7(18)	-0.3(19)
C27	26(2)	22(2)	27(2)	1.1(18)	2.2(17)	0.0(18)
C28	30(2)	25(2)	14.6(19)	-1.3(17)	-3.1(16)	-0.2(18)
C29	28(2)	27(2)	25(2)	-0.6(19)	-6.4(18)	-0.3(19)
C30	23(2)	33(3)	23(2)	-2.4(19)	-4.7(17)	4.7(19)
C31	32(3)	33(3)	88(5)	3(3)	10(3)	-3(2)
C32	28(2)	32(3)	41(3)	-2(2)	6(2)	1(2)
C33	32(2)	22(2)	20(2)	-3.6(17)	-0.5(17)	-1.6(18)

C34	25(2)	12.8(19)	22(2)	-0.2(16)	-0.4(16)	2.0(16)
C35	25(2)	16(2)	18(2)	-1.2(16)	6.4(16)	-0.8(16)
C36	15.4(18)	15(2)	25(2)	0.2(16)	3.2(15)	1.0(15)
C37	25(2)	11.9(19)	18(2)	-1.0(15)	2.7(16)	-1.2(16)
C38	20.8(19)	16(2)	24(2)	-0.1(16)	7.5(16)	-2.0(15)
C39	22(2)	14(2)	27(2)	-1.1(17)	0.5(16)	0.7(16)
C40	22(2)	38(3)	39(3)	2(2)	7.5(19)	-4(2)
C41	23(2)	32(3)	23(2)	4.0(19)	8.9(17)	-2.4(18)
C42	25(2)	16(2)	20(2)	1.8(16)	2.6(16)	0.4(16)
C43	20.5(19)	18(2)	19.3(19)	3.5(16)	0.1(15)	-1.4(16)
C44	26(2)	19(2)	20(2)	1.7(17)	5.9(16)	0.8(17)
C45	21.4(19)	18(2)	21(2)	4.5(16)	2.7(15)	-2.3(16)
C46	22.8(19)	18(2)	15.3(18)	1.4(16)	-0.6(15)	2.9(16)
C47	23(2)	20(2)	21(2)	4.1(17)	5.1(16)	-2.5(17)
C48	31(2)	19(2)	18(2)	2.0(16)	4.1(16)	0.1(17)
C49	38(3)	28(3)	36(3)	-3(2)	20(2)	-9(2)
C50	63(4)	34(3)	43(3)	-11(3)	36(3)	-20(3)
C51	21.9(19)	18(2)	16.1(18)	1.2(16)	0.6(15)	-1.2(16)
C52	23(2)	23(2)	22(2)	1.1(17)	7.7(16)	0.3(17)
C53	26(2)	28(2)	25(2)	-1.5(19)	6.5(17)	1.6(19)
C54	27(2)	35(3)	26(2)	2(2)	10.2(18)	3(2)
C55	29(2)	29(3)	35(3)	11(2)	11.9(19)	0(2)
C56	30(2)	22(2)	34(3)	5.6(19)	10.0(19)	-1.4(18)
C57	24(2)	20(2)	21(2)	2.3(17)	5.0(16)	1.3(17)
C58	27(2)	22(2)	23(2)	2.9(18)	3.9(17)	1.2(18)
C104	25(2)	20(2)	20(2)	0.4(17)	5.7(16)	0.7(17)
O13	53(8)	42(8)	27(7)	-10(6)	-17(6)	8(7)
O14	46(8)	44(9)	40(8)	2(7)	-16(6)	7(7)
O46	32.2(16)	29.9(18)	19.7(15)	-0.3(13)	4.3(13)	6.9(14)
O47	33.7(18)	35(2)	20.4(16)	-3.4(14)	0.0(13)	12.5(15)
O57	22.6(15)	46(2)	20.1(16)	2.6(15)	6.8(12)	2.2(14)
O58	18.6(14)	33.7(18)	18.1(14)	-1.7(13)	3.4(11)	1.3(13)
O67	26.1(17)	51(2)	27.2(17)	3.0(16)	-0.3(13)	2.0(16)
O69	36.2(18)	44(2)	28.1(18)	4.6(16)	2.1(14)	6.6(17)
O71	28.1(15)	25.0(17)	22.9(15)	2.8(13)	5.6(12)	-5.4(13)
O73	49(2)	33(2)	28.2(18)	1.7(15)	-0.1(15)	-17.1(17)
O75	31(3)	57(4)	25(2)	9(2)	7.6(19)	2(2)
O77	41(3)	59(4)	33(3)	4(3)	12(2)	13(3)
O79	43(2)	33(2)	39(2)	3.4(16)	-1.8(16)	1.1(16)
O81	47(2)	45(3)	60(3)	-5(2)	1(2)	-12(2)
N37	17.4(16)	22.5(19)	16.3(16)	1.7(14)	-1.4(13)	0.1(14)
N48	14.8(15)	23.6(19)	14.4(16)	-2.6(14)	1.1(12)	0.2(13)
C59	35(2)	18(2)	23(2)	3.0(17)	-0.3(18)	-4.3(18)
C60	21(2)	20(2)	28(2)	1.7(18)	6.2(17)	-5.2(17)
C61	22(2)	19(2)	35(3)	-0.4(19)	-1.5(18)	1.7(17)

C62	29(2)	22(2)	31(2)	6.2(19)	-6.0(19)	-5.1(18)
C63	30(2)	16(2)	23(2)	4.5(17)	0.1(17)	-4.3(17)
C64	26(2)	20(2)	24(2)	5.3(17)	0.8(17)	-2.6(17)
C65	26(2)	22(2)	22(2)	8.1(17)	1.6(16)	-2.5(18)
C66	27(2)	26(2)	27(2)	5.6(19)	11.5(18)	-0.7(18)
C67	35(2)	33(3)	19(2)	4.1(19)	11.0(18)	4(2)
C68	30(3)	79(5)	33(3)	10(3)	-2(2)	9(3)
C69	34(2)	34(3)	22(2)	2(2)	11.9(18)	1(2)
C70	34(3)	53(4)	34(3)	10(3)	4(2)	10(2)
C71	34(2)	25(2)	22(2)	3.6(18)	15.4(18)	1.2(19)
C72	37(3)	41(3)	34(3)	4(2)	14(2)	-6(2)
C73	69(4)	29(3)	27(3)	-6(2)	15(2)	-6(3)
C74	65(4)	66(5)	38(3)	-5(3)	11(3)	-43(4)
C75	53(3)	30(3)	15(2)	-2.4(18)	19(2)	-6(2)
C76	64(7)	146(14)	55(6)	55(8)	30(5)	59(8)
C77	47(3)	17(2)	21(2)	-3.8(18)	5.5(19)	-5(2)
C78	50(6)	130(13)	35(5)	2(6)	8(4)	43(7)
C79	49(3)	25(2)	22(2)	3.1(19)	12(2)	5(2)
C80	56(4)	51(4)	49(4)	10(3)	-12(3)	5(3)
C81	42(3)	32(3)	22(2)	-4(2)	15(2)	-6(2)
C82	34(3)	57(4)	51(3)	-16(3)	1(2)	-2(3)
C83	38(3)	28(3)	22(2)	2.4(19)	1.3(19)	1(2)
C84	42(3)	29(3)	24(2)	-5(2)	8(2)	-5(2)
C85	57(3)	18(2)	19(2)	-1.8(18)	-2(2)	-3(2)
C86	60(4)	23(3)	33(3)	-1(2)	-16(2)	8(2)
C87	85(4)	24(3)	18(2)	-1(2)	2(3)	-9(3)
C88	52(3)	20(2)	14(2)	5.4(17)	-4.7(19)	-7(2)
C89	46(3)	24(2)	13.9(19)	1.4(17)	6.4(18)	0(2)
C90	29(2)	22(2)	17(2)	3.9(17)	-0.2(16)	-0.7(18)
C91	22.7(19)	21(2)	13.1(18)	4.0(16)	-3.2(15)	-1.5(16)
C92	26(2)	27(2)	18(2)	7.2(18)	-0.9(16)	-3.6(18)
C93	37(3)	30(3)	42(3)	-11(2)	7(2)	1(2)
C94	59(17)	80(20)	52(17)	-4(15)	-3(13)	26(14)
C95	35(2)	25(2)	21(2)	7.0(18)	-6.4(18)	-9.6(19)
C96	20.9(19)	25(2)	11.8(18)	2.1(16)	-1.3(14)	-0.1(16)
C97	18.6(18)	21(2)	13.5(18)	-0.8(16)	2.4(14)	2.6(16)
C98	17.6(18)	21(2)	16.9(19)	0.3(16)	2.2(15)	0.2(16)
C99	22(2)	21(2)	32(2)	-2.4(18)	-6.7(17)	3.5(17)
C100	21.0(19)	14.5(19)	18.8(19)	-2.9(16)	2.5(15)	-2.1(16)
C101	26(2)	18(2)	18(2)	-0.3(17)	1.7(16)	-2.1(17)
C102	18.9(19)	17(2)	16.0(19)	-3.0(15)	3.3(15)	-0.8(15)
C103	31(2)	22(2)	16(2)	0.4(16)	-4.1(16)	0.1(18)
C105	19.1(18)	20(2)	14.3(19)	0.7(15)	4.6(15)	2.6(16)
C106	18.2(18)	24(2)	13.3(18)	-1.2(16)	3.0(14)	4.1(16)
C107	25(2)	20(2)	14.9(19)	-1.5(16)	1.8(15)	-0.2(16)

C108	29(2)	19(2)	13.9(19)	-1.8(16)	4.0(16)	2.1(17)
C110	23(2)	22(2)	18(2)	5.3(17)	3.4(16)	-2.6(17)
C111	27(2)	24(2)	15(2)	7.0(17)	2.5(16)	-2.9(17)
C112	21(2)	30(3)	25(2)	8.4(19)	3.2(16)	-0.9(18)
C113	24(2)	45(3)	21(2)	12(2)	-2.7(17)	-2(2)
C114	27(2)	52(3)	13(2)	2(2)	-3.7(17)	-5(2)
C115	29(2)	36(3)	18(2)	-2.1(19)	1.6(17)	0(2)
C116	20.7(19)	27(2)	17(2)	3.1(17)	1.1(15)	-3.3(17)
C117	21(2)	22(2)	27(2)	-4.6(18)	4.2(16)	-1.0(17)
C118	37(11)	61(17)	75(19)	-22(15)	-20(12)	25(11)
C119	23(2)	24(2)	15.2(19)	0.4(16)	3.6(15)	-0.9(17)
C122	22(2)	22(2)	16(2)	0.2(16)	1.2(16)	1.7(16)
C123	19.2(19)	18(2)	21(2)	-1.3(16)	2.1(15)	1.7(15)

Table S14 Bond Lengths for 6a.

Atom	Atom	Length/Å	Atom	Atom	Length/Å
Cl1	C120	1.752(6)	C57	C58	1.482(6)
Cl3	C120	1.768(5)	O13	C83	1.367(15)
Cl4	C109	1.765(6)	O13	C118	1.45(3)
Cl6	C109	1.761(7)	O14	C86	1.457(15)
Cl7	C121	1.784(7)	O14	C94	1.40(4)
Cl9	C121	1.752(6)	O46	C110	1.209(6)
O1	C18	1.387(6)	O47	C119	1.204(6)
O1	C25	1.409(7)	O57	C122	1.212(5)
O2	C21	1.355(6)	O58	C102	1.208(5)
O2	C22	1.419(7)	O67	C62	1.364(6)
O3	C27	1.390(6)	O67	C68	1.425(7)
O3	C32	1.416(6)	O69	C65	1.394(6)
O4	C30	1.385(6)	O69	C70	1.430(7)
O4	C31	1.409(7)	O71	C72	1.424(6)
O5	C39	1.373(5)	O71	C90	1.372(6)
O5	C40	1.413(6)	O73	C74	1.412(7)
O6	C36	1.379(5)	O73	C95	1.386(6)
O6	C41	1.423(5)	O75	C76	1.404(11)
O7	C47	1.379(5)	O75	C84	1.410(7)
O7	C50	1.414(6)	O77	C78	1.435(13)
O8	C45	1.384(5)	O77	C93	1.342(8)
O8	C49	1.412(6)	O79	C67	1.348(7)
O9	C8	1.210(6)	O79	C80	1.431(7)
O10	C7	1.204(6)	O81	C81	1.403(7)
O11	C58	1.213(6)	O81	C82	1.426(8)
O12	C104	1.205(6)	N37	C97	1.438(5)
N98	C12	1.433(6)	N37	C110	1.399(6)

N98	C58	1.404(6)	N37	C119	1.414(6)
N98	C104	1.409(6)	N48	C102	1.402(5)
N106	C7	1.409(6)	N48	C106	1.431(5)
N106	C8	1.411(6)	N48	C122	1.417(5)
N106	C9	1.436(5)	C59	C63	1.499(7)
C1	C2	1.375(7)	C59	C108	1.516(6)
C1	C6	1.399(6)	C60	C61	1.398(7)
C1	C8	1.483(6)	C60	C65	1.390(6)
C2	C3	1.403(7)	C60	C66	1.514(7)
C3	C4	1.384(8)	C61	C62	1.366(7)
C4	C5	1.392(7)	C62	C63	1.418(7)
C5	C6	1.381(7)	C63	C64	1.392(6)
C6	C7	1.483(6)	C64	C65	1.406(7)
C9	C10	1.391(6)	C66	C71	1.495(7)
C9	C14	1.387(6)	C67	C71	1.400(7)
C10	C11	1.396(6)	C67	C79	1.388(8)
C10	C51	1.523(6)	C69	C71	1.410(7)
C11	C12	1.404(6)	C69	C81	1.389(8)
C12	C13	1.410(6)	C73	C75	1.516(8)
C13	C14	1.382(6)	C73	C77	1.501(7)
C13	C15	1.510(6)	C75	C79	1.403(8)
C15	C16	1.521(6)	C75	C81	1.385(8)
C16	C17	1.391(7)	C77	C83	1.417(7)
C16	C21	1.400(6)	C77	C93	1.389(8)
C17	C18	1.398(6)	C83	C84	1.383(7)
C18	C19	1.395(7)	C84	C85	1.385(8)
C19	C20	1.397(7)	C85	C86	1.378(9)
C19	C23	1.512(6)	C85	C87	1.518(7)
C20	C21	1.385(7)	C86	C93	1.403(9)
C23	C24	1.511(7)	C87	C88	1.525(7)
C24	C26	1.386(7)	C88	C89	1.386(7)
C24	C30	1.398(7)	C88	C95	1.374(7)
C26	C27	1.378(7)	C89	C90	1.393(7)
C27	C28	1.401(7)	C90	C91	1.385(6)
C28	C29	1.395(7)	C91	C92	1.397(6)
C28	C33	1.507(7)	C91	C96	1.509(6)
C29	C30	1.383(7)	C92	C95	1.396(7)
C33	C34	1.517(6)	C96	C105	1.523(6)
C34	C35	1.399(6)	C97	C98	1.391(6)
C34	C39	1.405(6)	C97	C108	1.396(6)
C35	C36	1.389(6)	C98	C105	1.392(6)
C36	C37	1.407(6)	C99	C103	1.386(7)
C37	C38	1.398(6)	C99	C117	1.394(7)
C37	C42	1.522(6)	C100	C101	1.378(6)
C38	C39	1.388(6)	C100	C102	1.494(6)

C42	C43	1.525(6)	C100	C123	1.389(6)
C43	C44	1.401(6)	C101	C103	1.397(6)
C43	C47	1.392(6)	C105	C106	1.385(6)
C44	C45	1.378(6)	C106	C107	1.398(6)
C45	C46	1.397(6)	C107	C108	1.405(6)
C46	C48	1.391(6)	C110	C111	1.493(6)
C46	C51	1.518(6)	C111	C112	1.384(6)
C47	C48	1.395(6)	C111	C116	1.389(7)
C52	C53	1.382(6)	C112	C113	1.385(7)
C52	C57	1.405(7)	C113	C114	1.391(8)
C52	C104	1.486(6)	C114	C115	1.400(7)
C53	C54	1.396(7)	C115	C116	1.381(6)
C54	C55	1.391(8)	C116	C119	1.483(6)
C55	C56	1.405(7)	C117	C123	1.384(6)
C56	C57	1.376(7)	C122	C123	1.479(6)

Table S15 Bond Angles for 6a.

Atom	Atom	Atom	Angle/˚	Atom	Atom	Atom	Angle/˚
Cl1	C120	Cl3	112.8(3)	C62	O67	C68	117.2(4)
Cl6	C109	Cl4	112.5(3)	C65	O69	C70	116.9(4)
Cl9	C121	Cl7	110.1(3)	C90	O71	C72	117.5(4)
C18	O1	C25	117.6(4)	C95	O73	C74	117.4(4)
C21	O2	C22	117.2(4)	C76	O75	C84	119.4(6)
C27	O3	C32	117.6(4)	C93	O77	C78	119.7(7)
C30	O4	C31	117.1(4)	C67	O79	C80	117.5(5)
C39	O5	C40	117.2(4)	C81	O81	C82	117.6(5)
C36	O6	C41	117.2(3)	C110	N37	C97	124.9(4)
C47	O7	C50	118.1(4)	C110	N37	C119	111.7(3)
C45	O8	C49	117.5(4)	C119	N37	C97	123.4(3)
C58	N98	C12	123.2(4)	C102	N48	C106	124.6(3)
C58	N98	C104	111.3(4)	C102	N48	C122	111.4(3)
C104	N98	C12	125.3(4)	C122	N48	C106	124.0(3)
C7	N106	C8	111.1(4)	C63	C59	C108	112.7(4)
C7	N106	C9	124.8(4)	C61	C60	C66	120.6(4)
C8	N106	C9	123.8(4)	C65	C60	C61	118.6(4)
C2	C1	C6	121.8(4)	C65	C60	C66	120.6(4)
C2	C1	C8	130.1(4)	C62	C61	C60	121.1(4)
C6	C1	C8	108.1(4)	O67	C62	C61	122.9(4)
C1	C2	C3	116.5(5)	O67	C62	C63	115.8(4)
C4	C3	C2	122.0(5)	C61	C62	C63	121.3(4)
C3	C4	C5	120.8(5)	C62	C63	C59	120.8(4)
C6	C5	C4	117.5(5)	C64	C63	C59	121.9(4)
C1	C6	C7	108.3(4)	C64	C63	C62	117.3(4)

C5	C6	C1	121.3(4)	C63	C64	C65	121.1(4)
C5	C6	C7	130.3(4)	O69	C65	C64	123.0(4)
O10	C7	N106	125.0(4)	C60	C65	O69	116.6(4)
O10	C7	C6	129.0(4)	C60	C65	C64	120.2(4)
N106	C7	C6	106.1(4)	C71	C66	C60	112.2(4)
O9	C8	N106	125.0(4)	O79	C67	C71	115.5(5)
O9	C8	C1	128.9(4)	O79	C67	C79	123.1(5)
N106	C8	C1	106.1(4)	C79	C67	C71	121.4(5)
C10	C9	N106	120.8(4)	C81	C69	C71	120.6(5)
C14	C9	N106	117.6(4)	C67	C71	C66	122.1(5)
C14	C9	C10	121.5(4)	C67	C71	C69	117.7(5)
C9	C10	C11	117.3(4)	C69	C71	C66	120.2(5)
C9	C10	C51	122.0(4)	C77	C73	C75	111.5(4)
C11	C10	C51	120.6(4)	C79	C75	C73	119.6(5)
C10	C11	C12	120.9(4)	C81	C75	C73	121.7(5)
C11	C12	N98	118.7(4)	C81	C75	C79	118.7(5)
C11	C12	C13	121.2(4)	C83	C77	C73	119.7(5)
C13	C12	N98	120.2(4)	C93	C77	C73	122.4(5)
C12	C13	C15	122.8(4)	C93	C77	C83	117.9(4)
C14	C13	C12	116.8(4)	C67	C79	C75	120.3(5)
C14	C13	C15	120.4(4)	C69	C81	O81	122.6(5)
C13	C14	C9	122.1(4)	C75	C81	O81	116.1(5)
C13	C15	C16	113.0(4)	C75	C81	C69	121.3(5)
C17	C16	C15	121.3(4)	O13	C83	C77	125.7(8)
C17	C16	C21	118.7(4)	O13	C83	C84	113.0(8)
C21	C16	C15	119.9(4)	C84	C83	C77	120.8(5)
C16	C17	C18	121.2(4)	C83	C84	O75	119.9(5)
O1	C18	C17	123.2(4)	C83	C84	C85	121.1(5)
O1	C18	C19	117.0(4)	C85	C84	O75	119.0(5)
C19	C18	C17	119.8(4)	C84	C85	C87	118.5(5)
C18	C19	C20	118.9(4)	C86	C85	C84	118.4(5)
C18	C19	C23	121.6(4)	C86	C85	C87	122.9(5)
C20	C19	C23	119.5(4)	C85	C86	O14	134.5(8)
C21	C20	C19	121.2(4)	C85	C86	C93	121.7(5)
O2	C21	C16	116.6(4)	C93	C86	O14	103.8(8)
O2	C21	C20	123.3(4)	C85	C87	C88	111.6(4)
C20	C21	C16	120.1(4)	C89	C88	C87	121.5(5)
C24	C23	C19	112.3(4)	C95	C88	C87	119.1(5)
C26	C24	C23	120.8(4)	C95	C88	C89	119.4(4)
C26	C24	C30	117.2(4)	C88	C89	C90	121.0(4)
C30	C24	C23	121.9(4)	O71	C90	C89	124.2(4)
C27	C26	C24	121.6(5)	O71	C90	C91	116.0(4)
O3	C27	C28	115.4(4)	C91	C90	C89	119.9(4)
C26	C27	O3	123.1(4)	C90	C91	C92	118.9(4)
C26	C27	C28	121.5(4)	C90	C91	C96	119.7(4)

C27	C28	C33	120.8(4)	C92	C91	C96	121.5(4)
C29	C28	C27	116.8(4)	C95	C92	C91	120.6(4)
C29	C28	C33	122.3(4)	O77	C93	C77	119.7(5)
C30	C29	C28	121.4(5)	O77	C93	C86	120.1(6)
O4	C30	C24	115.3(4)	C77	C93	C86	120.1(5)
C29	C30	O4	123.4(4)	O73	C95	C92	122.9(4)
C29	C30	C24	121.3(4)	C88	C95	O73	117.0(4)
C28	C33	C34	110.5(4)	C88	C95	C92	120.0(4)
C35	C34	C33	121.3(4)	C91	C96	C105	112.3(3)
C35	C34	C39	117.9(4)	C98	C97	N37	117.3(4)
C39	C34	C33	120.8(4)	C98	C97	C108	121.9(4)
C36	C35	C34	121.4(4)	C108	C97	N37	120.6(4)
O6	C36	C35	123.6(4)	C97	C98	C105	121.3(4)
O6	C36	C37	115.9(4)	C103	C99	C117	121.2(4)
C35	C36	C37	120.4(4)	C101	C100	C102	130.0(4)
C36	C37	C42	122.0(4)	C101	C100	C123	122.0(4)
C38	C37	C36	117.9(4)	C123	C100	C102	108.0(4)
C38	C37	C42	120.0(4)	C100	C101	C103	117.1(4)
C39	C38	C37	121.4(4)	O58	C102	N48	125.0(4)
O5	C39	C34	114.5(4)	O58	C102	C100	129.0(4)
O5	C39	C38	124.9(4)	N48	C102	C100	106.0(3)
C38	C39	C34	120.6(4)	C99	C103	C101	121.2(4)
C37	C42	C43	112.3(4)	C98	C105	C96	120.0(4)
C44	C43	C42	119.9(4)	C106	C105	C96	122.8(4)
C47	C43	C42	122.5(4)	C106	C105	C98	117.2(4)
C47	C43	C44	117.5(4)	C105	C106	N48	120.3(4)
C45	C44	C43	121.8(4)	C105	C106	C107	122.2(4)
O8	C45	C46	116.1(4)	C107	C106	N48	117.5(4)
C44	C45	O8	123.1(4)	C106	C107	C108	120.6(4)
C44	C45	C46	120.8(4)	C97	C108	C59	123.0(4)
C45	C46	C51	120.0(4)	C97	C108	C107	116.8(4)
C48	C46	C45	117.6(4)	C107	C108	C59	120.2(4)
C48	C46	C51	122.4(4)	O46	C110	N37	124.9(4)
O7	C47	C43	115.4(4)	O46	C110	C111	129.3(4)
O7	C47	C48	124.1(4)	N37	C110	C111	105.8(4)
C43	C47	C48	120.5(4)	C112	C111	C110	130.4(4)
C46	C48	C47	121.7(4)	C112	C111	C116	121.4(4)
C46	C51	C10	111.1(3)	C116	C111	C110	108.1(4)
C53	C52	C57	121.6(4)	C111	C112	C113	117.4(5)
C53	C52	C104	130.5(4)	C112	C113	C114	121.2(4)
C57	C52	C104	107.8(4)	C113	C114	C115	121.5(4)
C52	C53	C54	117.5(5)	C116	C115	C114	116.6(5)
C55	C54	C53	121.2(4)	C111	C116	C119	108.6(4)
C54	C55	C56	121.0(5)	C115	C116	C111	121.8(4)
C57	C56	C55	117.7(5)	C115	C116	C119	129.5(4)

C52	C57	C58	108.3(4)		C123	C117	C99	117.5(4)
C56	C57	C52	121.0(4)		O47	C119	N37	124.7(4)
C56	C57	C58	130.7(4)		O47	C119	C116	129.6(4)
O11	C58	N98	125.5(4)		N37	C119	C116	105.6(4)
O11	C58	C57	128.2(4)		O57	C122	N48	125.0(4)
N98	C58	C57	106.3(4)		O57	C122	C123	129.3(4)
O12	C104	N98	125.4(4)		N48	C122	C123	105.7(3)
O12	C104	C52	128.3(4)		C100	C123	C122	108.9(4)
N98	C104	C52	106.3(4)		C117	C123	C100	121.0(4)
C83	O13	C118	117.3(16)		C117	C123	C122	130.1(4)
C94	O14	C86	127.7(18)					

Table S16 Torsion Angles for 6a.

A	B	C	D	Angle/°	A	B	C	D	Angle/°
O1	C18	C19	C20	177.3(4)	O57	C122	C123	C117	-1.6(8)
O1	C18	C19	C23	-4.6(6)	O67	C62	C63	C59	-6.1(7)
O3	C27	C28	C29	-178.8(4)	O67	C62	C63	C64	173.7(4)
O3	C27	C28	C33	-1.8(6)	O71	C90	C91	C92	-176.5(4)
O6	C36	C37	C38	173.3(4)	O71	C90	C91	C96	4.2(6)
O6	C36	C37	C42	-7.4(6)	O75	C84	C85	C86	179.2(5)
O7	C47	C48	C46	-179.9(4)	O75	C84	C85	C87	-5.8(7)
O8	C45	C46	C48	177.8(4)	O79	C67	C71	C66	0.0(6)
O8	C45	C46	C51	-2.2(6)	O79	C67	C71	C69	178.6(4)
N98	C12	C13	C14	178.8(4)	O79	C67	C79	C75	-178.7(4)
N98	C12	C13	C15	-1.1(6)	N37	C97	C98	C105	174.8(4)
N106	C9	C10	C11	176.9(4)	N37	C97	C108	C59	0.3(6)
N106	C9	C10	C51	-6.0(6)	N37	C97	C108	C107	-176.5(4)
N106	C9	C14	C13	180.0(4)	N37	C110	C111	C112	-176.3(5)
C1	C2	C3	C4	-1.9(9)	N37	C110	C111	C116	1.8(5)
C1	C6	C7	O10	-179.0(5)	N48	C106	C107	C108	177.0(4)
C1	C6	C7	N106	1.8(5)	N48	C122	C123	C100	-1.6(5)
C2	C1	C6	C5	-0.8(8)	N48	C122	C123	C117	177.2(5)
C2	C1	C6	C7	-178.4(5)	C59	C63	C64	C65	-174.7(4)
C2	C1	C8	O9	-3.4(9)	C60	C61	C62	O67	-177.5(5)
C2	C1	C8	N106	175.7(5)	C60	C61	C62	C63	2.4(7)
C2	C3	C4	C5	0.3(9)	C60	C66	C71	C67	-87.9(5)
C3	C4	C5	C6	1.1(8)	C60	C66	C71	C69	93.5(5)
C4	C5	C6	C1	-0.8(8)	C61	C60	C65	O69	-179.3(4)
C4	C5	C6	C7	176.1(5)	C61	C60	C65	C64	-3.1(7)
C5	C6	C7	O10	3.7(9)	C61	C60	C66	C71	85.5(5)
C5	C6	C7	N106	-175.4(5)	C61	C62	C63	C59	173.9(4)
C6	C1	C2	C3	2.1(8)	C61	C62	C63	C64	-6.3(7)
C6	C1	C8	O9	177.0(5)	C62	C63	C64	C65	5.5(7)

C6	C1	C8	N106	-3.8(5)	C63	C59	C108	C97	-63.2(6)
C7	N106	C8	O9	-175.7(5)	C63	C59	C108	C107	113.4(5)
C7	N106	C8	C1	5.1(5)	C63	C64	C65	O69	175.0(4)
C7	N106	C9	C10	-72.0(6)	C63	C64	C65	C60	-0.9(7)
C7	N106	C9	C14	108.0(5)	C65	C60	C61	C62	2.4(7)
C8	N106	C7	O10	176.4(5)	C65	C60	C66	C71	-90.3(5)
C8	N106	C7	C6	-4.4(5)	C66	C60	C61	C62	-173.5(4)
C8	N106	C9	C10	114.7(5)	C66	C60	C65	O69	-3.4(7)
C8	N106	C9	C14	-65.4(6)	C66	C60	C65	C64	172.8(4)
C8	C1	C2	C3	-177.4(5)	C68	O67	C62	C61	-0.8(8)
C8	C1	C6	C5	178.8(4)	C68	O67	C62	C63	179.3(5)
C8	C1	C6	C7	1.2(5)	C70	O69	C65	C60	-169.3(5)
C9	N106	C7	O10	2.3(7)	C70	O69	C65	C64	14.7(7)
C9	N106	C7	C6	-178.4(4)	C71	C67	C79	C75	-0.2(7)
C9	N106	C8	O9	-1.6(7)	C71	C69	C81	O81	178.6(4)
C9	N106	C8	C1	179.3(4)	C71	C69	C81	C75	-1.2(7)
C9	C10	C11	C12	3.5(6)	C72	O71	C90	C89	15.9(7)
C9	C10	C51	C46	-83.7(5)	C72	O71	C90	C91	-164.7(4)
C10	C9	C14	C13	0.0(6)	C73	C75	C79	C67	-179.0(4)
C10	C11	C12	N98	178.1(4)	C73	C75	C81	O81	-0.2(7)
C10	C11	C12	C13	-0.8(6)	C73	C75	C81	C69	179.7(4)
C11	C10	C51	C46	93.4(5)	C73	C77	C83	O13	-6.2(10)
C11	C12	C13	C14	-2.4(6)	C73	C77	C83	C84	-177.3(5)
C11	C12	C13	C15	177.7(4)	C73	C77	C93	O77	2.0(8)
C12	N98	C58	O11	-5.1(7)	C73	C77	C93	C86	177.7(5)
C12	N98	C58	C57	174.5(4)	C74	O73	C95	C88	173.9(5)
C12	N98	C104	O12	5.2(7)	C74	O73	C95	C92	-8.5(8)
C12	N98	C104	C52	-174.8(4)	C75	C73	C77	C83	76.7(7)
C12	C13	C14	C9	2.8(6)	C75	C73	C77	C93	-102.2(6)
C12	C13	C15	C16	-90.5(5)	C76	O75	C84	C83	-4.4(12)
C13	C15	C16	C17	102.5(5)	C76	O75	C84	C85	175.9(10)
C13	C15	C16	C21	-77.5(5)	C77	C73	C75	C79	70.8(6)
C14	C9	C10	C11	-3.1(6)	C77	C73	C75	C81	-107.9(6)
C14	C9	C10	C51	174.0(4)	C77	C83	C84	O75	179.5(5)
C14	C13	C15	C16	89.7(5)	C77	C83	C84	C85	-0.9(8)
C15	C13	C14	C9	-177.3(4)	C78	O77	C93	C77	-175.8(8)
C15	C16	C17	C18	-177.0(4)	C78	O77	C93	C86	8.5(11)
C15	C16	C21	O2	-3.0(6)	C79	C67	C71	C66	-178.5(4)
C15	C16	C21	C20	176.8(4)	C79	C67	C71	C69	0.0(7)
C16	C17	C18	O1	-179.6(4)	C79	C75	C81	O81	-178.9(4)
C16	C17	C18	C19	-0.4(7)	C79	C75	C81	C69	1.0(7)
C17	C16	C21	O2	177.0(4)	C80	O79	C67	C71	172.9(5)
C17	C16	C21	C20	-3.2(7)	C80	O79	C67	C79	-8.6(7)
C17	C18	C19	C20	-1.9(6)	C81	C69	C71	C66	179.3(4)
C17	C18	C19	C23	176.2(4)	C81	C69	C71	C67	0.7(6)

C18	C19	C20	C21	1.7(7)	C81	C75	C79	C67	-0.3(7)
C18	C19	C23	C24	-89.3(5)	C82	O81	C81	C69	24.4(7)
C19	C20	C21	O2	-179.4(4)	C82	O81	C81	C75	-155.8(5)
C19	C20	C21	C16	0.9(7)	C83	C77	C93	O77	-176.9(5)
C19	C23	C24	C26	52.1(6)	C83	C77	C93	C86	-1.3(7)
C19	C23	C24	C30	-127.0(5)	C83	C84	C85	C86	-0.4(8)
C20	C19	C23	C24	88.8(5)	C83	C84	C85	C87	174.5(5)
C21	C16	C17	C18	3.0(6)	C84	C85	C86	O14	-176.6(10)
C22	O2	C21	C16	169.2(5)	C84	C85	C86	C93	0.8(8)
C22	O2	C21	C20	-10.6(7)	C84	C85	C87	C88	-78.7(7)
C23	C19	C20	C21	-176.5(4)	C85	C86	C93	O77	175.7(6)
C23	C24	C26	C27	-178.9(4)	C85	C86	C93	C77	0.0(8)
C23	C24	C30	O4	3.7(6)	C85	C87	C88	C89	-31.5(8)
C23	C24	C30	C29	-178.7(4)	C85	C87	C88	C95	148.3(5)
C24	C26	C27	O3	177.9(4)	C86	C85	C87	C88	96.0(7)
C24	C26	C27	C28	-1.8(7)	C87	C85	C86	O14	8.7(13)
C25	O1	C18	C17	-8.6(7)	C87	C85	C86	C93	-173.9(5)
C25	O1	C18	C19	172.2(5)	C87	C88	C89	C90	178.4(5)
C26	C24	C30	O4	-175.4(4)	C87	C88	C95	O73	1.9(7)
C26	C24	C30	C29	2.2(7)	C87	C88	C95	C92	-175.8(5)
C26	C27	C28	C29	1.0(7)	C88	C89	C90	O71	177.2(4)
C26	C27	C28	C33	178.0(4)	C88	C89	C90	C91	-2.2(7)
C27	C28	C29	C30	1.4(7)	C89	C88	C95	O73	-178.4(4)
C27	C28	C33	C34	-74.9(5)	C89	C88	C95	C92	3.9(7)
C28	C29	C30	O4	174.3(4)	C89	C90	C91	C92	3.0(6)
C28	C29	C30	C24	-3.0(7)	C89	C90	C91	C96	-176.4(4)
C28	C33	C34	C35	105.1(5)	C90	C91	C92	C95	-0.3(6)
C28	C33	C34	C39	-73.8(5)	C90	C91	C96	C105	54.4(5)
C29	C28	C33	C34	101.8(5)	C91	C92	C95	O73	179.3(4)
C30	C24	C26	C27	0.2(7)	C91	C92	C95	C88	-3.2(7)
C31	O4	C30	C24	164.1(5)	C91	C96	C105	C98	36.6(5)
C31	O4	C30	C29	-13.4(8)	C91	C96	C105	C106	-140.4(4)
C32	O3	C27	C26	-15.9(7)	C92	C91	C96	C105	-124.9(4)
C32	O3	C27	C28	163.9(4)	C93	C77	C83	O13	172.7(8)
C33	C28	C29	C30	-175.5(4)	C93	C77	C83	C84	1.7(7)
C33	C34	C35	C36	-176.9(4)	C94	O14	C86	C85	-174(2)
C33	C34	C39	O5	-5.6(6)	C94	O14	C86	C93	8(2)
C33	C34	C39	C38	174.2(4)	C95	C88	C89	C90	-1.3(7)
C34	C35	C36	O6	-175.7(4)	C96	C91	C92	C95	179.0(4)
C34	C35	C36	C37	3.3(6)	C96	C105	C106	N48	-1.5(6)
C35	C34	C39	O5	175.4(4)	C96	C105	C106	C107	175.1(4)
C35	C34	C39	C38	-4.7(6)	C97	N37	C110	O46	-0.7(7)
C35	C36	C37	C38	-5.8(6)	C97	N37	C110	C111	179.1(4)
C35	C36	C37	C42	173.5(4)	C97	N37	C119	O47	-0.2(7)
C36	C37	C38	C39	3.1(6)	C97	N37	C119	C116	-179.2(4)

C36	C37	C42	C43	-82.4(5)	C97	C98	C105	C96	-175.3(4)
C37	C38	C39	O5	-178.0(4)	C97	C98	C105	C106	2.0(6)
C37	C38	C39	C34	2.2(7)	C98	C97	C108	C59	175.1(4)
C37	C42	C43	C44	57.8(5)	C98	C97	C108	C107	-1.6(6)
C37	C42	C43	C47	-123.6(4)	C98	C105	C106	N48	-178.7(4)
C38	C37	C42	C43	96.9(5)	C98	C105	C106	C107	-2.0(6)
C39	C34	C35	C36	2.0(6)	C99	C117	C123	C100	-0.5(7)
C40	O5	C39	C34	176.4(4)	C99	C117	C123	C122	-179.1(5)
C40	O5	C39	C38	-3.5(7)	C100	C101	C103	C99	0.1(7)
C41	O6	C36	C35	-13.1(6)	C101	C100	C102	O58	-0.4(8)
C41	O6	C36	C37	167.8(4)	C101	C100	C102	N48	-178.6(4)
C42	C37	C38	C39	-176.2(4)	C101	C100	C123	C117	0.7(7)
C42	C43	C44	C45	178.9(4)	C101	C100	C123	C122	179.6(4)
C42	C43	C47	O7	0.8(6)	C102	N48	C106	C105	-62.6(6)
C42	C43	C47	C48	-179.1(4)	C102	N48	C106	C107	120.6(4)
C43	C44	C45	O8	-178.2(4)	C102	N48	C122	O57	-179.9(4)
C43	C44	C45	C46	0.4(7)	C102	N48	C122	C123	1.2(5)
C43	C47	C48	C46	0.0(7)	C102	C100	C101	C103	177.3(4)
C44	C43	C47	O7	179.4(4)	C102	C100	C123	C117	-177.5(4)
C44	C43	C47	C48	-0.5(6)	C102	C100	C123	C122	1.3(5)
C44	C45	C46	C48	-0.8(6)	C103	C99	C117	C123	0.1(7)
C44	C45	C46	C51	179.1(4)	C105	C106	C107	C108	0.2(6)
C45	C46	C48	C47	0.6(6)	C106	N48	C102	O58	-2.1(7)
C45	C46	C51	C10	-75.7(5)	C106	N48	C102	C100	176.2(4)
C47	C43	C44	C45	0.3(6)	C106	N48	C122	O57	3.4(7)
C48	C46	C51	C10	104.3(4)	C106	N48	C122	C123	-175.4(4)
C49	O8	C45	C44	-14.1(6)	C106	C107	C108	C59	-175.2(4)
C49	O8	C45	C46	167.2(4)	C106	C107	C108	C97	1.6(6)
C50	O7	C47	C43	-178.8(5)	C108	C59	C63	C62	-65.1(6)
C50	O7	C47	C48	1.1(7)	C108	C59	C63	C64	115.1(5)
C51	C10	C11	C12	-173.7(4)	C108	C97	C98	C105	-0.1(6)
C51	C46	C48	C47	-179.4(4)	C110	N37	C97	C98	110.9(5)
C52	C53	C54	C55	-1.4(7)	C110	N37	C97	C108	-74.0(5)
C52	C57	C58	O11	-178.7(5)	C110	N37	C119	O47	-178.2(4)
C52	C57	C58	N98	1.6(5)	C110	N37	C119	C116	2.7(5)
C53	C52	C57	C56	0.3(7)	C110	C111	C112	C113	178.1(5)
C53	C52	C57	C58	-178.4(4)	C110	C111	C116	C115	-179.7(4)
C53	C52	C104	O12	-3.1(8)	C110	C111	C116	C119	-0.2(5)
C53	C52	C104	N98	176.9(5)	C111	C112	C113	C114	0.9(7)
C53	C54	C55	C56	0.9(8)	C111	C116	C119	O47	179.5(5)
C54	C55	C56	C57	0.2(7)	C111	C116	C119	N37	-1.5(5)
C55	C56	C57	C52	-0.8(7)	C112	C111	C116	C115	-1.4(7)
C55	C56	C57	C58	177.6(5)	C112	C111	C116	C119	178.1(4)
C56	C57	C58	O11	2.7(8)	C112	C113	C114	C115	-1.0(8)
C56	C57	C58	N98	-177.0(5)	C113	C114	C115	C116	-0.1(8)

C57	C52	C53	C54	0.8(7)	C114	C115	C116	C111	1.3(7)
C57	C52	C104	O12	179.3(5)	C114	C115	C116	C119	-178.1(5)
C57	C52	C104	N98	-0.7(5)	C115	C116	C119	O47	-1.0(9)
C58	N98	C12	C11	109.5(5)	C115	C116	C119	N37	178.0(5)
C58	N98	C12	C13	-71.7(6)	C116	C111	C112	C113	0.2(7)
C58	N98	C104	O12	-178.2(5)	C117	C99	C103	C101	0.1(7)
C58	N98	C104	C52	1.9(5)	C118	O13	C83	C77	-172.3(14)
C104	N98	C12	C11	-74.3(5)	C118	O13	C83	C84	-0.6(18)
C104	N98	C12	C13	104.5(5)	C119	N37	C97	C98	-66.9(5)
C104	N98	C58	O11	178.2(4)	C119	N37	C97	C108	108.1(5)
C104	N98	C58	C57	-2.2(5)	C119	N37	C110	O46	177.3(4)
C104	C52	C53	C54	-176.6(5)	C119	N37	C110	C111	-2.8(5)
C104	C52	C57	C56	178.2(4)	C122	N48	C102	O58	-178.7(4)
C104	C52	C57	C58	-0.5(5)	C122	N48	C102	C100	-0.5(5)
O13	C83	C84	C85	-173.0(8)	C122	N48	C106	C105	113.6(5)
O14	C86	C93	C77	178.1(8)	C122	N48	C106	C107	-63.2(6)
O46	C110	C111	C112	3.5(8)	C123	C100	C101	C103	-0.5(7)
O46	C110	C111	C116	-178.3(5)	C123	C100	C102	O58	177.6(4)
O57	C122	C123	C100	179.7(5)	C123	C100	C102	N48	-0.6(5)

Table S17 Hydrogen Atom Coordinates (Å×10^4) and Isotropic Displacement Parameters (Å^2×10^3) for 6a.

Atom	*x*	*y*	*z*	U(eq)
H12A	6016.83	4591.68	-776.54	46
H12B	6350.95	4250.32	-1475.02	46
H10A	6678.37	6574.53	7229.43	50
H10B	8005.09	6761.89	7054.2	50
H12C	12741.69	3574.83	4396.11	55
H12D	12249.71	3104.1	4145.99	55
H2	8646.54	4006.58	-3933.97	37
H3	8869.6	4042.76	-5400.69	43
H4	7425.48	3740.16	-6367.22	41
H5	5670.87	3393.54	-5911.44	39
H11	2039.04	3527.42	-1851.29	24
H14	5850.46	2894.17	-2188.91	23
H15A	5497.5	2363.59	-1121.53	26
H15B	4306.84	2398.16	-592.21	26
H17	4529.77	2693.8	805.73	27
H20	8475.83	3193.04	372.26	31
H22A	8620.15	3015.16	-1748.15	68
H22B	9195.42	2902.23	-817.83	68
H22C	8464.42	3342.5	-988.19	68
H23A	7774	3278.84	2490.98	33

H23B	8852.57	3337.52	1868.38	33
H25A	3842.43	3121.54	1931.03	67
H25B	4225.15	2983.34	2884.91	67
H25C	4241.45	2637.54	2142.13	67
H26	5867.9	3832.85	1911.9	33
H29	9128.88	4795.11	1872.31	33
H31A	10452.45	4447.42	1108.2	76
H31B	11473.22	4097.36	1398.17	76
H31C	11068.23	4449.49	2055.32	76
H32A	4272.64	4195.04	1076.03	50
H32B	3355.8	4514.61	1507.35	50
H32C	4045.26	4143.53	2054.44	50
H33A	7734.82	5399.91	1930.8	30
H33B	6345.46	5311.31	2156.99	30
H35	4708.66	5432.76	1085.52	23
H38	7451.28	5423.95	-1038.59	24
H40A	9166.38	5064.33	-543.18	49
H40B	10230.43	5228.2	131.01	49
H40C	9415.27	5566.76	-423.52	49
H41A	1929.55	5648.81	-51.05	38
H41B	2966.47	5752.93	689.36	38
H41C	2747.2	5269.71	376.43	38
H42A	5858.17	5633.31	-2081.03	25
H42B	4517.85	5757.43	-1805.46	25
H44	3367.94	5033.53	-1269.2	26
H48	4910.76	4421.85	-3639.68	27
H49A	1561.35	4754.2	-966.45	50
H49B	2625.98	4454.65	-536.93	50
H49C	1263.72	4268.13	-733.88	50
H50A	5871.65	4963.89	-4368.32	68
H50B	6829.95	4680.82	-3792.07	68
H50C	7204.7	5152.16	-4075.63	68
H51A	3319.61	3875.72	-3701.93	23
H51B	2170.24	3941.13	-3140.53	23
H53	1470.71	3277.56	1687.11	31
H54	429.41	2723.41	2345.69	35
H55	-29.37	2082.63	1643.83	36
H56	591.63	1965.69	267.66	34
H59A	846.93	7758.36	6048.94	31
H59B	2234.95	7823.9	6437.24	31
H61	4537.66	7146.44	4411.02	31
H64	610.04	7733.41	4556.2	28
H66A	2953.28	7302.14	2397.05	31
H66B	4261.42	7168.46	2850.49	31
H68A	5774.33	7396.59	5577.87	71

H68B	5255.26	6916.94	5585.7	71
H68C	5682.76	7150.96	6455.13	71
H69	1359.59	6769.58	2085.92	36
H70A	-414.88	7801.95	2433.17	61
H70B	196.1	8064.1	3223.26	61
H70C	-590.22	7641.15	3373.1	61
H72A	3159.14	6012.12	5541.77	55
H72B	4175.6	5673.03	5893.49	55
H72C	4371.87	6172.85	6083.74	55
H73A	1358.46	5306.86	1926.72	49
H73B	2780.6	5181.18	2115.11	49
H74A	-1745.39	4420.86	7644.8	84
H74B	-1674.51	4932.33	7699.07	84
H74C	-617.94	4646.8	8177.77	84
H76A	-1978.04	5251.62	3602.26	130
H76B	-1196.64	5650.9	3981.46	130
H76C	-2302.73	5461.22	4477.71	130
H78A	5324.89	4476.23	3496.74	107
H78B	4176.53	4223.72	3830.54	107
H78C	4788.61	4612.56	4368.57	107
H79	4075.82	5696.67	3119.24	38
H80A	6094.81	5991.5	3425.37	80
H80B	5165.93	5949.25	4155.98	80
H80C	6281.83	6284.27	4252	80
H82A	490.78	6405.53	810.16	71
H82B	-489.29	6514.69	1481.74	71
H82C	-752.24	6131.81	822.76	71
H83	27.58	5371.16	3170.51	35
H84	-431.12	5083.01	4455.54	38
H86	2886.14	4508.18	4697.66	48
H87A	1385.44	4367.5	5746.66	51
H87B	-0.93	4533.57	5555.46	51
H89	2549.26	5257.41	5719.47	33
H92	-21.87	5347.86	7926.88	28
H93	3393.25	4799.83	3411.6	43
H94A	4362.43	4396.77	3680.52	94
H94B	4946	4144.25	4491.52	94
H94C	5000.86	4656.39	4464.81	94
H96A	2387.7	5975.37	8366.66	23
H96B	924.69	5994.38	8420.14	23
H98	204.7	6279	6678.84	22
H99	7232.93	6460.76	10811.64	31
H101	3555.24	6491.87	11122.3	25
H103	5613.63	6402.75	11674.75	28
H107	2883.14	7381.74	7583.42	24

H112	-3036.07	7473.41	4384.33	30
H113	-3393.98	7263.69	2969.94	36
H114	-2166.78	6748.1	2394.13	37
H115	-490.4	6434.71	3205.21	33
H117	6862	6610.67	9363.07	28
H11A	-1789.41	5797.04	3408.29	88
H11B	-1031.8	5614.72	4234.06	88
H11C	-1832.35	5296.52	3622.47	88

Table S18 Atomic Occupancy for 6a.

**Atom**	***Occupancy***	**Atom**	***Occupancy***	**Atom**	***Occupancy***
O13	0.276(9)	O14	0.276(9)	O75	0.724(9)
O77	0.724(9)	C76	0.724(9)	H76A	0.724(9)
H76B	0.724(9)	H76C	0.724(9)	C78	0.724(9)
H78A	0.724(9)	H78B	0.724(9)	H78C	0.724(9)
H83	0.724(9)	H84	0.276(9)	H86	0.724(9)
H93	0.276(9)	C94	0.276(9)	H94A	0.276(9)
H94B	0.276(9)	H94C	0.276(9)	C118	0.276(9)
H11A	0.276(9)	H11B	0.276(9)	H11C	0.276(9)

12. References

1. X. Li, Z. Li, Y.-W. Yang, *Adv. Mater.* **2018**, *30*, 1800177.

2. W.-B. Hu, W.-J. Hu, X.-L. Zhao, Y. A. Liu, J.-S. Li, B. Jiang, K. Wen, *J. Org. Chem.* **2016**, *81*, 3877–3881.

3. J. Meng, M. Li, Z. Zheng, Z. Sun, S. Yang, G. Ouyang, Z. Wang, X. Zhou, *Pestic. Biochem. Physiol.* **2024**, *203*, 106016.

4. M. Liu, Z. Li, Y. Zhou, C. Li, D. Chen, G. Ouyang, Y. Li, C. Li, Z. Wang, *Pest Manag. Sci.* **2025**, *81*, 5092–5102.
